# Caesium fluoride-mediated hydrocarboxylation of alkenes and allenes: scope and mechanistic insights†
†Electronic supplementary information (ESI) available. See DOI: 10.1039/c9sc02467k


**DOI:** 10.1039/c9sc02467k

**Published:** 2019-09-11

**Authors:** Ashot Gevorgyan, Marc F. Obst, Yngve Guttormsen, Feliu Maseras, Kathrin H. Hopmann, Annette Bayer

**Affiliations:** a Department of Chemistry , UiT The Arctic University of Norway , Norway . Email: annette.bayer@uit.no; b Hylleraas Centre for Quantum Molecular Sciences , Department of Chemistry , UiT The Arctic University of Norway , Norway . Email: kathrin.hopmann@uit.no; c Institute of Chemical Research of Catalonia (ICIQ) , Spain

## Abstract

A caesium fluoride-mediated hydrocarboxylation of olefins is disclosed that does not rely on precious transition metal catalysts and ligands. The reaction occurs at atmospheric pressures of CO_2_ in the presence of 9-BBN as a stoichiometric reductant. Stilbenes, β-substituted styrenes and allenes could be carboxylated in good yields. The developed methodology can be used for preparation of commercial drugs as well as for gram scale hydrocarboxylation. Computational studies indicate that the reaction occurs *via* formation of an organocaesium intermediate.

## Abstract 2

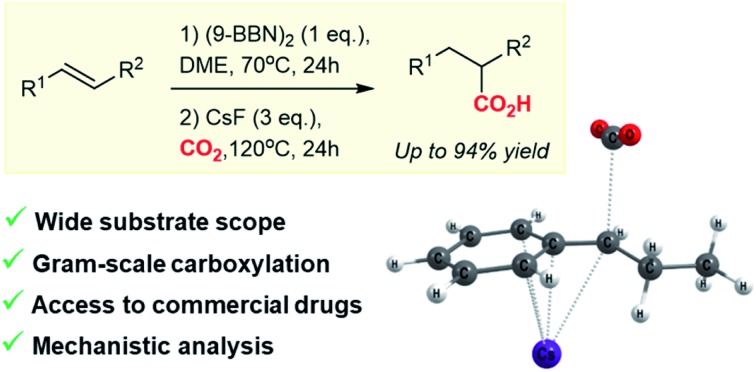
A caesium fluoride-mediated regioselective hydrocarboxylation of alkenes and allenes is described. Computational studies reveal that the stability of an organocaesium intermediate accounts for the observed reactivity.

## Introduction

CO_2_ provides a sustainable source of carbon that increasingly is being used in chemical synthesis.^1^ Construction of anthropogenic chemical carbon cycles^2^ by valorisation of CO_2_ into chemicals, materials, and fuels, is a promising strategy for replacing fossil carbon in the chemical industry.^1^,^3^ Various studies have shown that transition metal-based catalysts are able to selectively reduce CO_2_ into simple chemicals, such as formic acid, methanol, alkanes, and CO.3*a* CO_2_ can also be incorporated into carbonates, which are valuable starting materials for polymer science.3*b* Use of CO_2_ in C–C bond forming reactions opens new pathways towards value-added products and pharmaceuticals from CO_2_.^4^

**Scheme 1:**
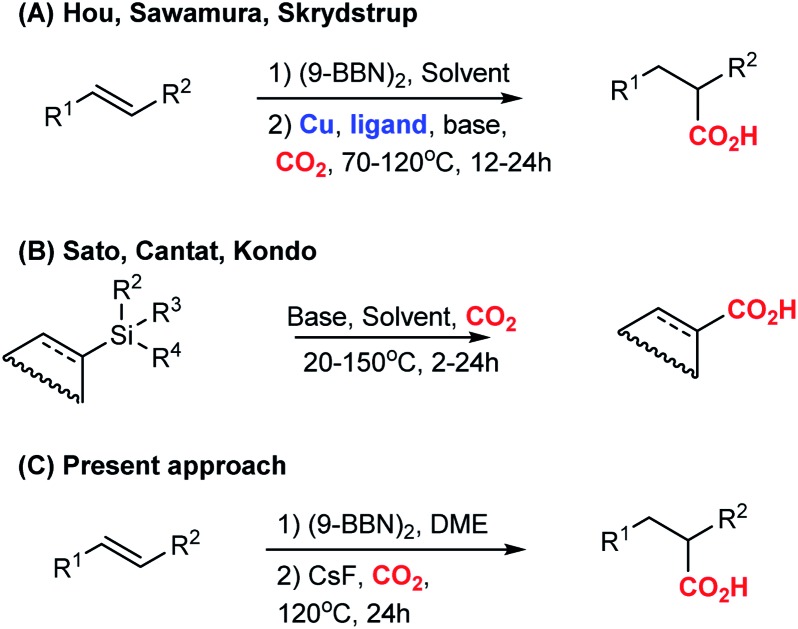
Previous works (A^6^ and B^7^) and present study (C).

As part of our research interest to develop C–CO_2_ bond forming reactions,^5^ we became interested in the copper-catalysed hydrocarboxylation reactions reported by Hou,6*a* Sawamura6*b* and Skrydstrup6*c* (Scheme 1A). In these formal hydrocarboxylations, an initial hydroboration with 9-borabicyclo[3.3.1]nonane (9-BBN) transforms an alkene to an organoborane, which in a subsequent copper-catalysed step is carboxylated with CO_2_. In order to elucidate the mechanistic details of the carboxylation step, we embarked on a computational study of the reaction. Surprisingly, our computational analysis indicated the existence of a feasible carboxylation pathway that does not involve the copper complex. Our subsequent experiments confirmed that it is possible to carboxylate *in situ* formed organoboranes in absence of copper. Related reports of carboxylations with CO_2_ in absence of transition metals include fluoride-mediated carboxylations of organosilanes7b–f and KO*t*Bu-mediated carboxylations of benzylboronic esters (Scheme 1B).7*a* However, none of these reports addressed a possible role of the counterion for the observed reactivity. To the best of our knowledge, a CsF-mediated hydrocarboxylation with *in situ* generated organoboranes has not been reported. In the following, we detail our findings of the CsF-mediated hydrocarboxylation of alkenes with CO_2_ (Scheme 1C). A detailed computational analysis indicates that the reaction proceeds *via* formation of organocaesium intermediates. The described transformation expands the repertoire of carboxylation reactions that can be performed without the use of transition metal catalysts.

## Results and discussion

On basis of a preliminary computational investigation of the hydrocarboxylation of alkenes, we speculated that *trans*-stilbene **1a** can be hydrocarboxylated *via* an organoborane intermediate in the absence of a transition metal catalyst, which is in contrast to previous reports.^6^ To test our hypothesis, we used 9-BBN in dioxane to convert **1a** into an organoborane intermediate, which we attempted to carboxylate with CO_2_ in a CsF-mediated transformation (Table 1). Gratifyingly, the corresponding carboxylic acid **2a** was obtained in 83% yield (Table 1, entry 2). In comparison, the previously reported copper-catalysed reaction6*c* gave the carboxylation product **2a** in 78% yield (Table 1, entry 1). The higher yield in absence of copper was observed for several substrates (ESI, Scheme S1 and S2†). This phenomenon may be explained by a copper-promoted decarboxylation reaction slowly consuming the product **2a**.^8^ To support this hypothesis, we mixed 2-phenylpropionic acid with the Cu complex under reaction conditions, which lead us to recover only 95% of the starting acid, while in the absence of Cu, the recovery of acid was 99% (ESI, Scheme S1†).

**Table 1:** Optimization of reaction conditions^*a*^

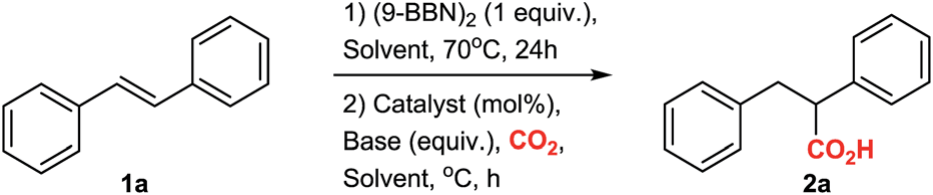					
Entry	Catalyst (mol%)	Base (equiv.)	Solvent	°C/h	^*b*^Yield %
1	IPrCuI(5)^*c*^	CsF(3)	Dioxane	120/24	78
2	—	CsF(3)	Dioxane	120/24	83
3	—	CsF(3)	THF	120/24	61
4	—	CsF(3)	Diglyme	120/24	67
5	—	CsF(3)	DME	120/24	87
6	—	CsF(3)	DMA	120/24	0
7	—	CsF(3)	Toluene	120/24	70
8	—	CsF(3)	MeCN	120/24	0
9	—	KF(3)	DME	120/24	50
10	—	NaF(3)	DME	120/24	0
11	—	Cs_2_CO_3_(3)	DME	120/24	71
12	—	K_2_CO_3_(3)	DME	120/24	67
13	—	KO*t*Bu(3)^*d*^	DME	120/24	47
14	—	CsF(2)	DME	120/24	57
15	—	CsF(3)	DME	80/24	59
16	—	CsF(3)	DME	120/28	85

^*a*^Reaction conditions: (1) **1a** (0.444 mmol), (9-BBN)_2_ (1 equiv.), solvent (3 mL), 70 °C, 24 h. (2) (IPrCuI (5 mol%)), base (2–3 equiv.), CO_2_ 120 mL, 80–120 °C, 24–28 h.

^*b*^Isolated yields.

^*c*^The active catalyst was prepared *in situ* (IPr = 1,3-bis(2,6-diisopropylphenyl)imidazol-2-ylidene).

^*d*^The reaction mixture was run at 20 °C for 30 min before addition of CO_2_.

We proceeded to establish the optimum reaction conditions of the base-mediated carboxylation reaction. Screening of different solvents revealed that the reaction works well in ethers. The best yield was observed in dimethoxyethane (DME, 87%, Table 1, entry 5). The screening of different bases indicated that the optimal base is CsF (87% yield; Table 1, entry 5), while other fluoride containing bases like KF and NaF gave inferior results (50% and 0% yield, entry 9 and 10). Interestingly, also Cs_2_CO_3_ and K_2_CO_3_ gave good results (71% and 67% yield, entry 11 and 12), showing that not only the fluoride anion is important for the outcome of the reaction. On basis of the reports by Hou,6*a* Sawamura6*b* and Schomaker,7*a* we also attempted to employ alkoxides as base, but observed difficulties in our system. If mixed simultaneously, the reaction between alkoxide and CO_2_ lead to the corresponding carbonates, and no carboxylation product was formed. Alkoxide bases were effective only if the second reaction step was run without CO_2_ for minimum 30 minutes at 20 °C, followed by addition of CO_2_, which provided a yield of 47% (Table 1, entry 13). Further screening related to the stoichiometry of reagents, duration of the reaction, and temperature showed that the best conditions are 1 equiv. olefin and (9-BBN)_2_ and 3 equiv. CsF in DME at 120 °C for 24 h (Table 1, entry 5; for further details see ESI, Table S1†).

**Scheme 2:**
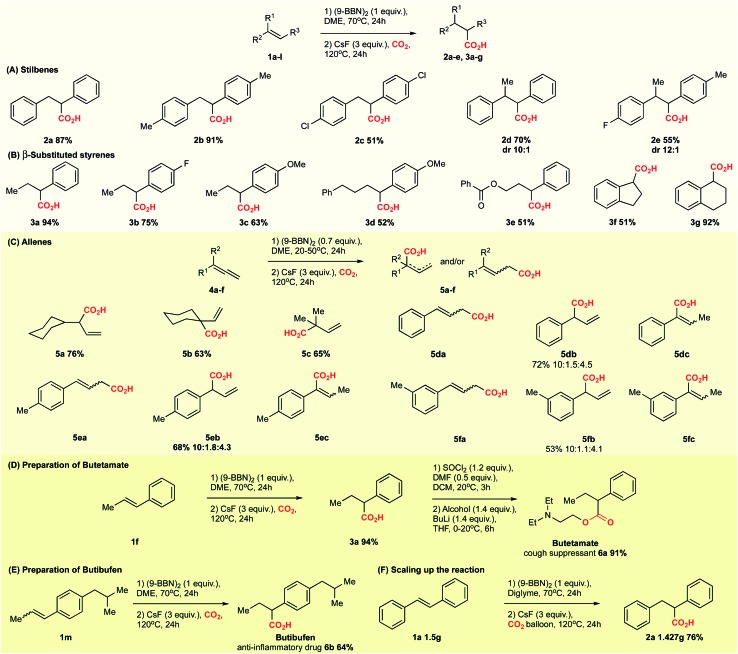
Substrate scope of the CsF-mediated hydrocarboxylation.

With the optimized conditions at hand, we explored the substrate scope of the reaction (Scheme 2; ESI Scheme S3†). Screening of different substrates showed that the CsF-mediated hydrocarboxylation works only on systems where the *in situ* hydroboration step (mediated by 9-BBN) generates benzylic or allylic borane intermediates. Indeed, styrene and cyclohexene were not reactive under optimal conditions (ESI Table S1†). On the other hand, stilbenes, β-substituted styrenes and allenes were successful substrates. Neither the pinacol ester of benzylboronic acid nor *in situ*-generated benzylic catechol esters (instead of the organoborane intermediate) were reactive in the CsF-mediated carboxylation (ESI Scheme S4†).

The CsF-mediated hydrocarboxylation of stilbene derivatives (**1a–e**) produced the corresponding carboxylic acids **2a–e** with moderate to excellent yields (Scheme 2A). The conversion of (*E*)-α-methyl stilbenes (**1d**, **1e**) was regioselective, providing exclusive carboxylation at the sterically less hindered β-position and resulting in formation of **2d** and **2e**, each as a mixture of diastereomers. The observed regioselectivity is assumed to be controlled by steric effects.^6^,^9^,^10^

The CsF-mediated hydrocarboxylation of β-substituted styrenes (**1f–l**) gave the α-carboxylated products **3a–g** as the sole product in moderate to good yields (Scheme 2B). Interestingly, whereas the selectivity of the 9-BBN-initiated hydroboration of β-substituted styrenes is substrate-dependent and generally gives a non-regioselective mixture of boranes,^6^,^9^,^10^ our base-initiated carboxylation appears to convert only the benzylic boranes, providing a single carboxylation product with excellent regioselectivity for **3a–g** (Scheme 2B). In contrast, the Cu-catalysed hydrocarboxylation does not differentiate between the regioisomeric borane intermediates, giving a mixture of carboxylic acids.6*c* For example, in the copper-catalysed hydrocarboxylation of indene (**1k**), we observed a mixture of α- and β-regioisomers with a ratio of 4 : 1 (ESI, Scheme S2†).

Allenes also proved to be suitable substrates for CsF-mediated hydrocarboxylation (Scheme 2C). Both aliphatic and aromatic allenes could be transformed to carboxylic acids **5a–f** with good yields. The regioselectivity of the reaction was strongly dependent on the nature of the allene substituents. Allenes with aliphatic substituents gave the internal allylic carboxylic acid as a single product **5a–c** (Scheme 2C). In contrast, allenes possessing aromatic substituents yielded the carboxylic acids **5d–f** as isomeric mixtures, with the terminal carboxylic acids as the major product (Scheme 2C). Although borane-mediated hydroboration of allenes has been described,^11^ the selectivity is not well understood, and equilibria of internal and terminal allylic boranes have been proposed. Recently, Chida and Sato showed that the hydroboration of allenes in deuterated THF occurs predominantly at the terminal double bond.11*f* Carboxylation of alkyl allenes may then proceed from the terminal allylic borane with an allyl shift, or involve the internal allylic borane generated through equilibration. For aryl allenes, the direct carboxylation of the terminal allylic borane is preferred as the system is less likely to rearrange due to conjugation.

We further tested the possibility of asymmetric hydrocarboxylation using the (–)-isopinocampheylborane TMEDA complex – a chiral analogue of 9-BBN – in the initial hydroboration step (ESI Scheme S5†).^12^ Even though the hydroboration–oxidation of *trans*-β-methylstyrene gave the corresponding alcohol with 36% *ee* (ESI, Scheme S5A†), the hydroboration–carboxylation using our conditions led to racemic product (ESI, Scheme S5B†). The observed racemisation may be explained by the structural instability of intermediate organometallic compounds, such as the organoborane or an organocaesium (*vide infra*) at elevated temperatures.^13^

In order to show the versatility of the developed CsF-mediated hydrocarboxylation reaction, we applied our strategy in the synthesis of the commercial drugs butetamate **6a** and butibufen **6b** from β-substituted styrenes (Scheme 2D and E). Although in case of butetamate, four steps are required (hydroboration, carboxylation, preparation of acid anhydride, and esterification), only two isolations were needed, providing almost quantitative yields. Similarly, butibufen was obtained in 64% yield using the direct hydrocarboxylation of β-substituted styrene **1m** (Scheme 2E).

Importantly, the hydrocarboxylation reaction can be scaled up (Scheme 2F). For this we changed the solvent from DME to diglyme (2-methoxyethyl ether), which has a higher boiling point, allowing the reaction to be performed in simple flasks using a CO_2_ balloon. Starting from 1.5 g of stilbene, we could prepare 1.427 g of the corresponding acid **2a** (Scheme 2F). The yield at gram scale (76%, Scheme 2) is slightly larger compared to the small scale (67%, Table 1, entry 4), probably due to better recovery of material during work-up at larger scale.

**Fig. 1:**
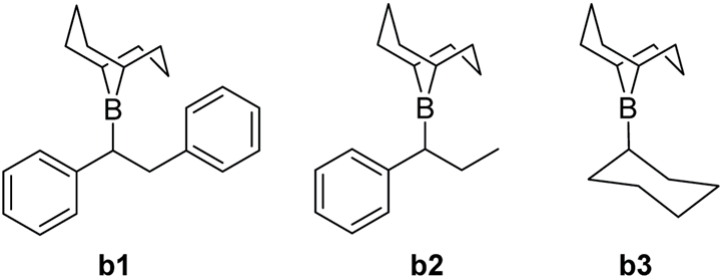
Computationally investigated boranes.

The computational analysis of the CsF-mediated carboxylation of *in situ* generated organoboranes provided insights into the mechanistic steps. Three boranes were included in the theoretical study (Fig. 1): **b1** and **b2**, derived from the experimentally reactive alkenes *trans*-stilbene (**1a**) and *trans*-β-methylstyrene (**1f**), and **b3**, corresponding to the non-reactive alkene cyclohexene (**1o**). Three possible reaction mechanisms (referred to as **A**, **B** and **C**) were found by an automated search of the potential energy surface with the AFIR method.^14^ Mechanism **A** (ESI, Fig. S6†) is characterized by a nucleophilic attack of the reactive carbon of the borane on a CO_2_ molecule, followed by a transmetalation with CsF. This mechanism is considered not viable, as all the evaluated boranes show a computed Gibbs free activation energy of >50 kcal mol^–1^ for the first step (ESI, Table S2†).

**Scheme 3:**
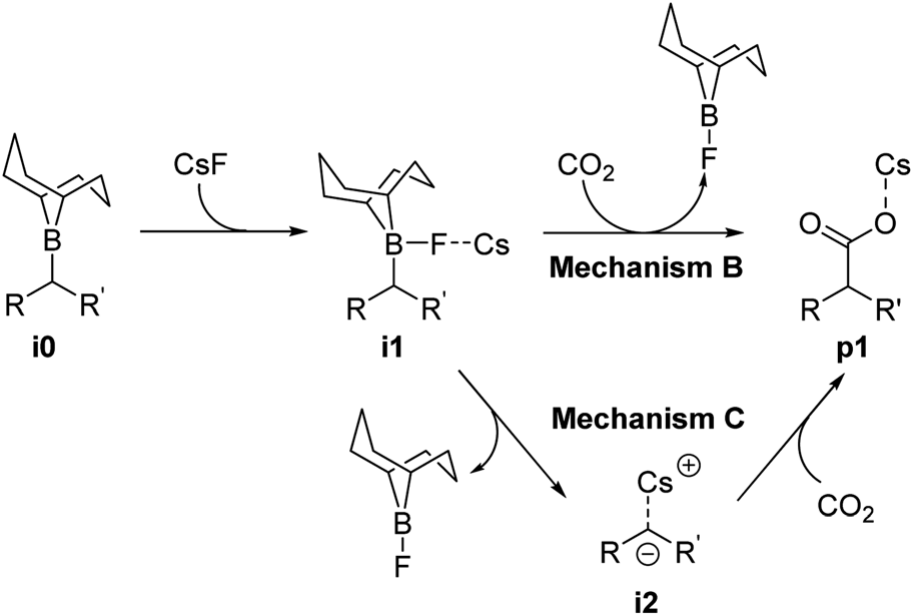
Computed reaction mechanisms **B** and **C**.

Reaction mechanism **B** (Scheme 3) occurs through two steps: First, the formation of a B–F bond between the borane **i0** and a CsF molecule yielding intermediate **i1**, and second, the nucleophilic attack of intermediate **i1** on CO_2_. The latter step is characterized by a concerted formation of the C–CO_2_ bond and the cleavage of the B–C bond, releasing F-(9-BBN) and forming the product **p1**. The overall barrier computed for the different boranes with mechanism **B** (ESI, Table S3†) is significantly lower than with mechanism **A** (Table S2†). However, with values of 44.4 kcal mol^–1^ (cyclohexane-derived borane **b3**) to 52.3 kcal mol^–1^ (*trans*-β-methylstyrene-derived borane **b2**), the barriers are too high to be overcome at the reaction temperature of 120 °C.^15^

**Fig. 2:**
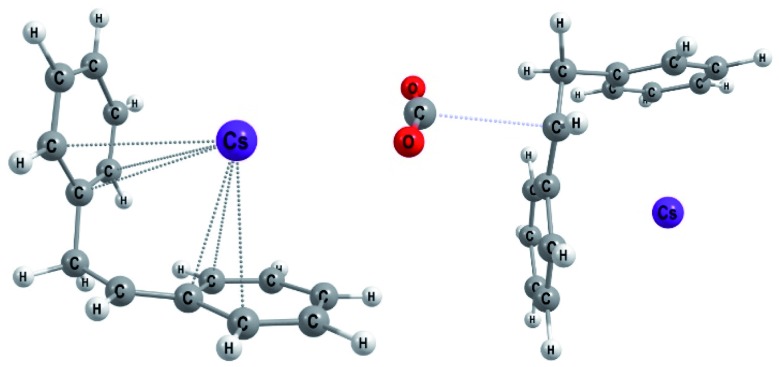
Optimized geometries for **b1** (Mechanism **C**): the organocaesium intermediate **i2** (left) and the C–CO_2_ bond formation TS (TS**_i2–p1_**, right).

The first step of mechanism **C** (Scheme 3) is the same as for **B**, the formation of intermediate **i1**. In the next step, the boron–carbon bond is cleaved, releasing a F-(9-BBN) molecule and forming the organocaesium intermediate **i2** (Fig. 2). In the final step, **i2** undergoes a nucleophilic attack on a CO_2_ molecule. Interestingly, at the insertion TS for substrate **b1**, CO_2_ shows no clear preference to interact with the cesium centre (Fig. 2; see also ESI, Fig. S9†), in contrast to other computational studies predicting CO_2_–Cs interactions.^16^ However, for **b2**, a preference for a weak CO_2_–Cs interaction is seen (ESI, Fig. S10†). The reason may be that the Cs atom experiences stronger interactions with the two phenyl rings of **b1** than with the single aromatic ring in **b2**, making additional CO_2_–Cs interactions preferable for **b2**.

**Fig. 3:**
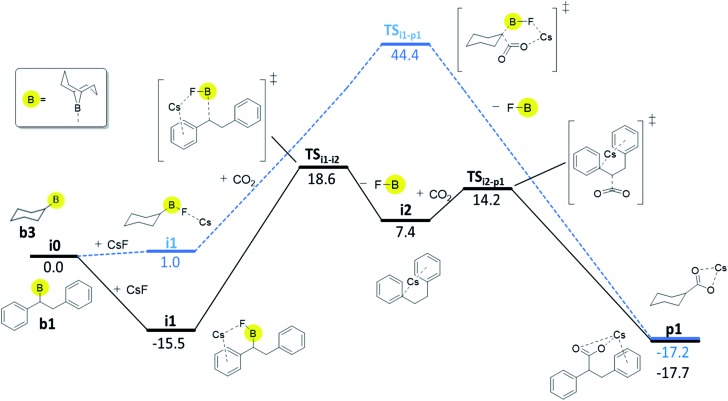
Computed Gibbs free energy profile (kcal/mol; DLPNO-CCSD(T)//ωB97XD) of the preferred reaction pathways, mechanism **C** for **b1** (black solid line) and mechanism **B** for **b3** (blue dashed line).

For boranes **b1** and **b2**, the rate-limiting step of mechanism C is the cleavage of the boron–carbon bond with overall barriers of 34.0 kcal mol^–1^ for borane **b1** (derived from *trans*-stilbene) and 36.7 kcal mol^–1^ for **b2** (derived from *trans*-β-methylstyrene). Mechanism **C** is thus the preferred pathway for boranes **b1** and **b2**. The full energy profile for carboxylation of **b1***via* mechanism C is shown in Fig. 3.

For borane **b3** (derived from cyclohexene), the rate-limiting step of mechanism **C** is the C–CO_2_ bond formation with an overall barrier of 51.5 kcal mol^–1^, which is not feasible. The lowest computed barrier for borane **b3** is thus observed with mechanism **B** (Fig. 3), which at 44.4 kcal mol^–1^ is not feasible at the experimental temperature, in line with the experimentally observed lack of reactivity of cyclohexene.

Our computational and experimental results are in good agreement, indicating that the carboxylation of benzylic boranes occurs *via* reaction mechanism **C**, which features an organocaesium intermediate **i2**. The benzylic boranes **b1** and **b2** are able to stabilize the organocaesium intermediate **i2***via* delocalization of the negative charge, and *via* cation–π interactions between caesium and the aromatic substituents on the organoborane. Similar Cs–π interactions have been observed in related computational studies.^17^ The cost of forming **i2** is only 7.4 kcal mol^–1^ for **b1** and 12.7 kcal mol^–1^ for **b2**. The cyclohexyl borane **b3** lacks these stabilizing effects, resulting in a relative energy of 37.2 kcal mol^–1^ for the **i2** intermediate. We therefore suggest that the stability of the organocaesium intermediate **i2** is the factor determining the reactivity of olefins in the CsF-mediated hydrocarboxylation.

## Conclusions

We report a CsF-mediated hydrocarboxylation of alkenes and allenes proceeding *via* a hydroboration with 9-BBN followed by a CsF-mediated carboxylation of the resulting organoboranes. The caesium fluoride-mediated carboxylation was effective for *in situ* generated benzylic and allylic organoboranes derived from stilbenes, β-substituted styrenes and allenes, providing the corresponding carboxylic acids with good yields and excellent regioselectivities. The developed methodology was demonstrated at gram-scale and was used for the production of commercial drugs. Computational studies indicate that benzylic organoboranes are transformed to organocaesium intermediates, which then undergo a nucleophilic attack on CO_2_. Stabilisation of the organocaesium intermediate by the aromatic substituent account for the observed selectivity towards benzylic organoboranes.

## Methods

Experimental and computational details are given in the ESI.† The ESI includes experimental procedures and analytical data, an example input for DLPNO-CCSD(T) calculations, computed energies for the full reaction pathways for **b1**, **b2** and **b3**, and a comparison of computed C–CO_2_ TS structures. A separate xyz file contains all optimized coordinates in a format that allows easy visualization with Mercury.

### General procedure for metal–free hydrocarboxylation of stilbenes, β-substituted styrenes and allenes

Inside the glove box, a 45 mL pressure tube was charged with the corresponding olefin or allene (1.5 mmol), (9-BBN)_2_ (1 equiv. for olefins or 0.7 equiv. for allenes) and dry DME (7 mL). The flask was closed with a suitable cap, removed from the glove box and heated to 70 °C (olefin) or 50 °C (allene) for 24 h. Afterwards, the pressure tube was transferred back to the glove box. To the reaction mixture at 20 °C was added CsF (3 equiv.). The pressure tube was closed with the cap and removed from the glove box. Afterwards CO_2_ (120 mL) was added *via* a syringe, which was followed by stirring of the reaction mixture at 120 °C for 24 h. Next, the reaction mixture was diluted with 30 mL Et_2_O and transferred into a 500 mL separating funnel. The resulting mixture was extracted with 30 mL saturated basic (NaHCO_3_, 1 M KOH) solution (3 times). The resulting basic solution was washed with 15 mL Et_2_O (once), acidified (50–55 mL 6 M HCl) and extracted with 30 mL Et_2_O (3 times). The resulting solution of Et_2_O was distilled to dryness to give the corresponding acid (in case of **5c** the final solution of Et_2_O was dried using Na_2_SO_4_, which was followed by careful evaporation of solvents).

### Computational methods

Density functional theory (DFT) calculations were performed with the ωB97XD hybrid functional,^18^ as implemented in Gaussian 16, Revision B.01.^19^ Geometries were optimized with the SDD ECP and basis set for Cs and the 6-31+G* basis set for all other elements. Initial guess structures for the transition states were obtained through linear transit calculations and through artificial force induced reaction modelling (AFIR) as implemented in GRRM.^14^ Solvation effects were included in the final geometry optimizations *via* the IEFPCM model (1,4-dioxane). Explicit solvent molecules may bind to specific points in the system, but we do not expect them to affect the overall mechanistic picture,^17^ and because of this they were omitted from the calculation. Vibrational, entropic, and temperature corrections were computed at 393.15 Kelvin, with the same level of theory as geometry optimizations. Electronic energies were obtained with DLPNO-CCSD(T)^20^ using ORCA 4.1.1.^21^ The ZORA operator as well as the basis sets SARC-ZORA-TZVPP (for Cs) and ZORA-def2-QZVPP (all other elements) were employed. The final Gibbs free energies (Δ*G*_DPLNO-CCSD(T)//ωB97XD_) in the main text correspond to the DLPNO-CCSD(T) electronic energies combined with the DFT-based vibrational, entropic and temperature corrections, and the standard state (SS, 393.15 K) conversion in case of a change in the number of moles:^22^ Δ*G*_DPLNO-CCSD(T)//ωB97XD_ = Δ*G*_ωB97XD/IEFPCM_ – Δ*E*_ωB97XD/IEFPCM_ + Δ*E*_DPLNO-CCSD(T)_ + SS. All ORCA and Gaussian calculations were performed on the Norwegian supercomputer Stallo at UiT, whereas GRRM calculations were performed on the computer cluster at ICIQ. More information on the computational details and example inputs as well as additional DFT energies are given in the ESI.†

## Conflicts of interest

There are no conflicts to declare.

## Supplementary Material

Supplementary information

Supplementary information
