# Supplementary material for: Caesium fluoride-mediated hydrocarboxylation of alkenes and allenes: scope and mechanistic insights
Source: Chem Sci. 2019 Sep 11;10(43):10072–8. doi: 10.1039/c9sc02467k (PMC6991174; doi:10.1039/c9sc02467k)
Supplement: Supplementary file 1 [file SC-010-C9SC02467K-s001.pdf]

Electronic Supplementary Material (ESI) for Chemical Science.
This journal is © The Royal Society of Chemistry 2019

Supplementary Information
Caesium Fluoride-Mediated Hydrocarboxylation of Alkenes and Allenes: Scope and Mechanistic Insights

Ashot Gevorgyan,[a,†] Marc F. Obst,[b,†] Yngve Guttormsen,[a] Feliu Maseras,[c] Kathrin H. Hopmann,*[b] Annette Bayer*[a]

[a] Department of Chemistry, UiT - The Arctic University of Norway, 9037 Tromsø, Norway.
[b] Hylleraas Centre for Quantum Molecular Sciences, Department of Chemistry, UiT - The Arctic University of Norway, 9037 Tromsø, Norway.
[c] Institute of Chemical Research of Catalonia (ICIQ), The Barcelona Institute of Science and Technology, Avgda. Països Catalans, 16, 43007 Tarragona, Spain.
[†] These authors contributed equally to this work.
E-mail: kathrin.hopmann@uit.no, annette.bayer@uit.no

Table of content:

General considerations 2
Optimization of reaction 2
Control experiments 5
Starting material used in the study 6
Control experiments on benzylboronic esters 7
Carboxylation of enantiomerically enriched organoborane 8
Setup of the reaction 9
General procedures 14
Characterization of products 22
Copies of spectra 31
Computational details 112

General considerations

Commercially available starting materials, reagents, catalysts and anhydrous and degassed solvents were used without further purification. Flash column chromatography was performed with Merck silica gel 60 (230-400 mesh). The solvents for column chromatography were distilled before use. Thin layer chromatography was carried out using Merck TLC Silica gel 60 F_{254} and visualized by short-wavelength ultraviolet light or by treatment with potassium permanganate ($KMnO_4$) stain. ^{1}H, ^{13}C and ^{19}F NMR spectra were recorded on a Bruker Avance 400 MHz at 20°C. All ^{1}H NMR spectra are reported in parts per million (ppm) downfield of TMS and were measured relative to the signals for $CHCl_3$ (7.26 ppm), methanol (4.87ppm, 3.31ppm) and DMSO (2.50 ppm). All ^{13}C NMR spectra were reported in ppm relative to residual $CDCl_3$ (77.20 ppm), methanol (49.1 ppm) or DMSO (39.70 ppm) and were obtained with ^{1}H decoupling. Coupling constants, *J*, are reported in Hertz (Hz). High-resolution mass spectra (HRMS) were recorded from methanol solutions on an LTQ Orbitrap XL (Thermo Scientific) either in negative or in positive electrospray ionization (ESI) mode. Chromatographic analysis was performed on a Waters Acquity UPC^2 system, equipped with a Torus DIOL column (2.1 mm x 50 mm, 130 Å, 1.7 μmeter) and coupled to QDa Detector with electrospray ionisation (ESI) and single-quadropole mass detector. The mobile phase for SFC analysis was a gradient of MeOH + 0.5% Ammonia solution (25%) in supercritical CO_2 over 2.0 min (gradient 0-20%). Melting points were measured using Stuart SMP50 automatic melting point detector.

Warning!

Most of the reactions were performed in specialized glassware under pressure. The glassware should always be examined for damages before any manipulation. All laboratory safety procedures must be followed strictly and the work with pressure tubes must be conducted behind a shield.

Optimization of reaction

General experimental procedure. For general setup, see Figure S1-S4.

Inside of glove box 45 mL pressure tube was charged with *trans*-stilbene (0.444 mmol), $(9\text{-BBN})_2$ (1 equiv.) and corresponding dry solvent (3 mL). The flask was closed with suitable cap, removed from the glove box and heated to 70°C for 24h. Afterwards, the pressure tube was transferred back to the glove box. To the reaction mixture at 20°C was added the base (0-3 equiv.) and (in case of Cu-catalysed experiments) previously prepared solution of catalyst (the mixture of CuI (5 mol%), IPrHCl (6 mol%) and NaO*t*Bu (6 mol%) in appropriate dry solvent (1 mL) was stirred at 20°C for 30 min). The pressure tube was closed with the cap and removed from the glove box. Afterwards CO_2 (120 mL) was added *via* a syringe, which was followed by stirring of reaction mixture at 120°C for 24h. Next, the reaction mixture was diluted with 15 mL Et_2O and transferred into 250 mL separating funnel. The resulting mixture was extracted with 15 mL saturated basic ($NaHCO_3$, 1M KOH) solution (3 times). Resulting basic solution was washed with 10 mL Et_2O (once), acidified (25-30 mL 6M HCl) and extracted with 15 mL Et_2O (3 times). The resulting solution of Et_2O was distilled to dryness to give corresponding acid.

In cases of diglyme, triglyme and PEG400 the final Et_2O solution was washed with 10 mL distilled water (3 times) before evaporation.

Table S1. Reaction Optimization.[a]

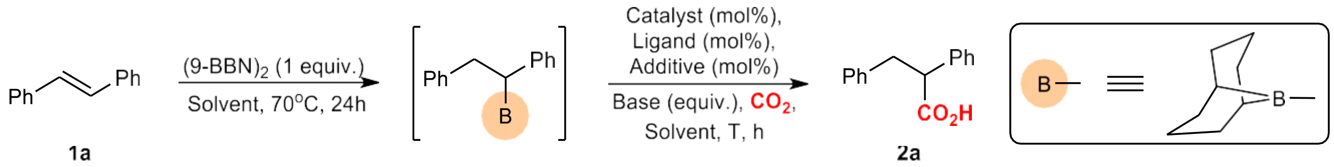

Entry	Catalyst (mol%)	Ligand (mol%)	Additive (mol%)	Base (equiv.)	Solvent	T, °C	h	Yield %[b]
1	CuI	IPrHCl	NaO*t*Bu	CsF (3)	Dioxane	120	24	78
2	-	-	-	CsF (3)	Dioxane	120	24	83
3	-	-	-	CsF (3)	THF	120	24	61
4	-	-	-	CsF (3)	Diglyme	120	24	67
5	-	-	-	CsF (3)	Triglyme	120	24	64
6	-	-	-	CsF (3)	PEG400	120	24	0
**7**	-	-	-	**CsF (3)**	**DME**	**120**	**24**	**87**
8	-	-	-	CsF (3)	DMA	120	24	0
9	-	-	-	CsF (3)	MeCN	120	24	0
10	-	-	-	CsF (3)	Toluene	120	24	70
11	-	-	-	CsF (3)	Cyclohexane	120	24	12
12	-	-	-	CsF (3)	ODCB	120	24	65
13	-	-	-	CsF (3)	DCE	120	24	0
14	-	-	-	CsF (3)	CCl_4	120	24	0
15	-	-	-	Cs_2CO_3 (3)	DME	120	24	71
16	-	-	-	CsOAc (3)	DME	120	24	34
17	-	-	-	Rb_2CO_3 (3)	DME	120	24	65
18	-	-	-	KF (3)	DME	120	24	50

19	-	-	-	KO*t*Bu (3)[c]	DME	120	24	47
20	-	-	-	K_2CO_3 (3)	DME	120	24	67
21	-	-	-	K_3PO_4 (3)	DME	120	24	0
22	-	-	-	NaO*t*Bu (3)[c]	DME	120	24	33
23	-	-	-	NaF (3)	DME	120	24	0
24	-	-	-	CsF (2)	DME	120	24	57
25	-	-	-	-	DME	120	24	0
26	-	-	-	CsF (3)	DME	80	24	59
27	-	-	-	CsF (3)	DME	80	72	74
28	-	-	-	CsF (3)	DME	120	16	78
29	-	-	-	CsF (3)	DME	120	28	85
30	CuI (5)	IPrHCl (6)	NaO*t*Bu (6)	CsF (3)	DME	120	24	86

[a] Reaction conditions: 1) **1a** (0.444 mmol), $(9\text{-BBN})_2$ (1 equiv.), Solvent (3 mL), 70°C, 24h. 2) (CuI (5 mol%), IPrHCl (1,3-bis(2,6-diisopropylphenyl)imidazolium chloride, 6 mol%), NaO*t*Bu (6 mol%)), base (0-3 equiv.), CO_2 120 mL, 80-120°C, 16-72h. [b] Isolated yields. [c] Before addition of CO_2 the reaction mixture, containing the base, was mixed at 20°C for 30 min.

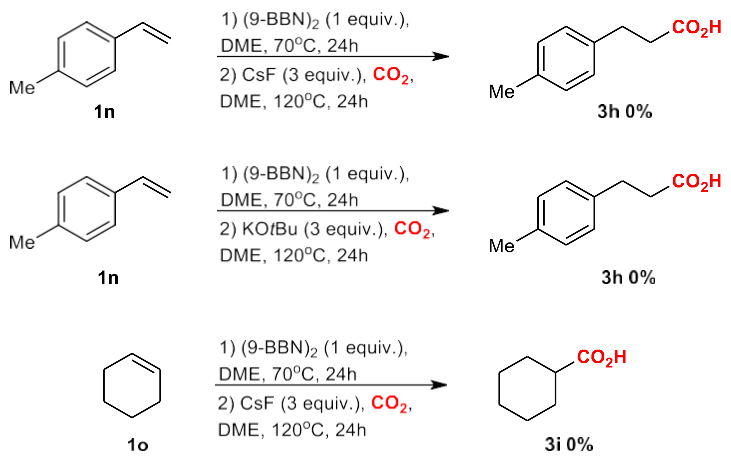

Control experiments for comparison with Cu-containing conditions

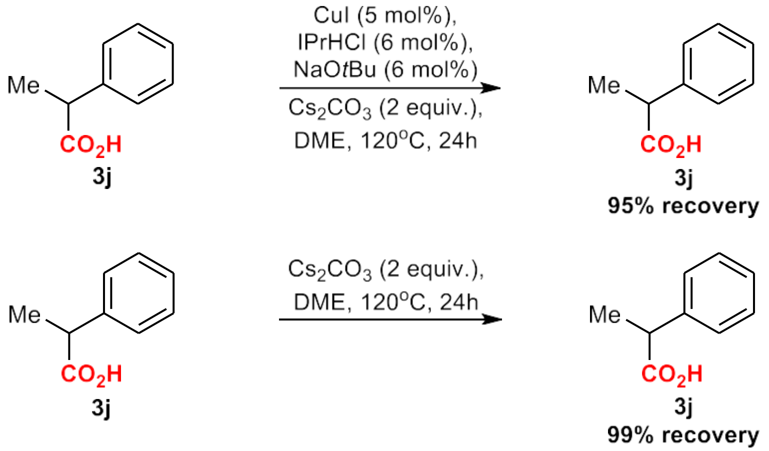

Scheme S1. Control experiments on Cu-catalysed decarboxylation.

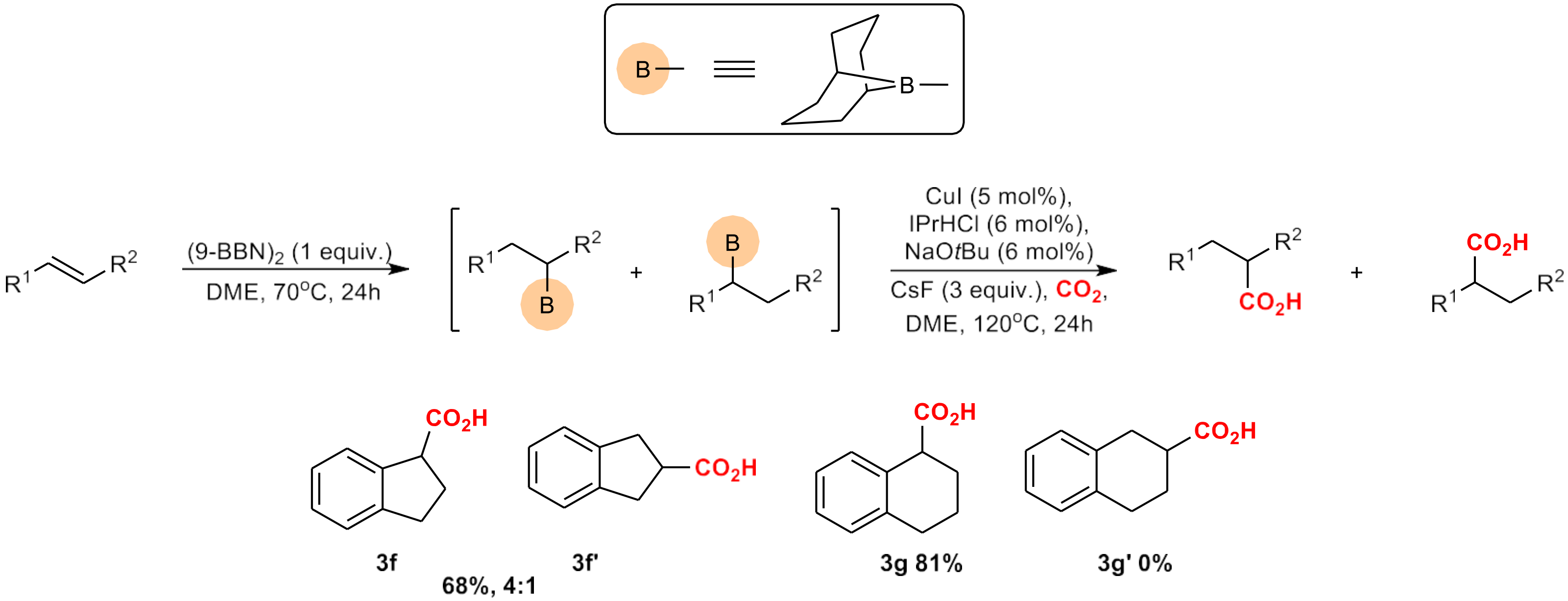

Scheme S2. Cu-catalysed hydrocarboxylation of indene and 1,2-dihydronaphthalene.

Starting material used in the study

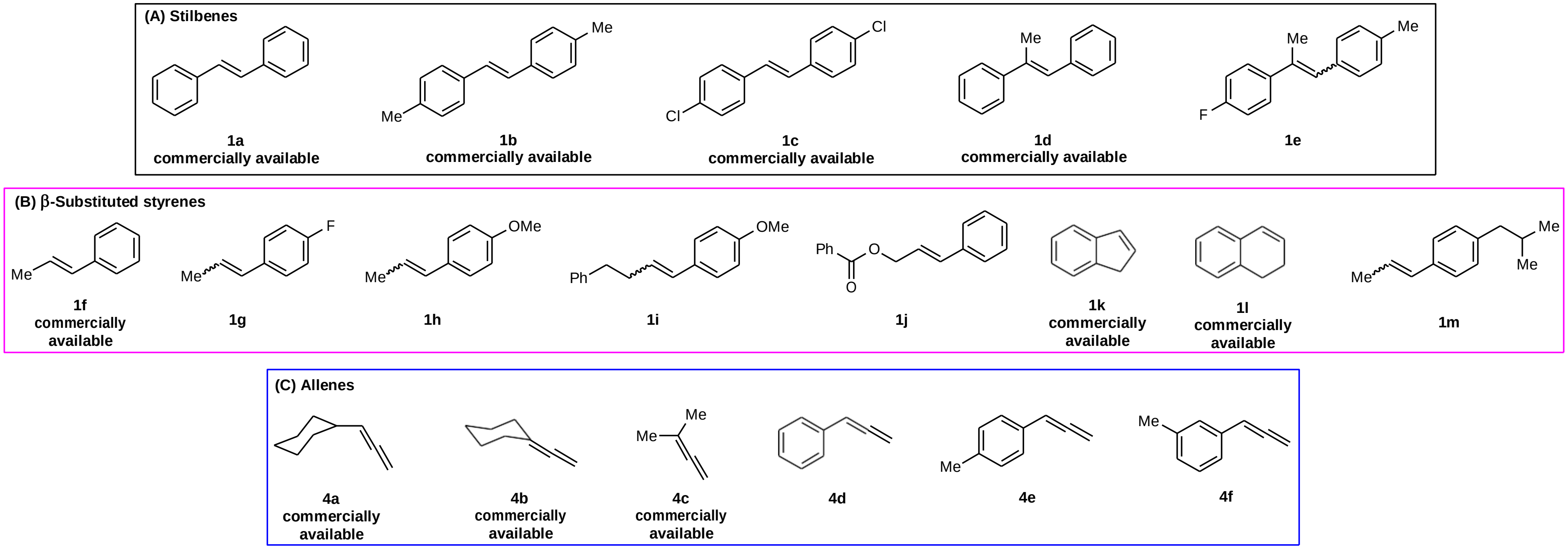

Scheme S3. List of starting materials used in the work.

Control experiments on benzylboronic esters

(A) CsF-mediated carboxylation of benzylboronic acid pinacol ester

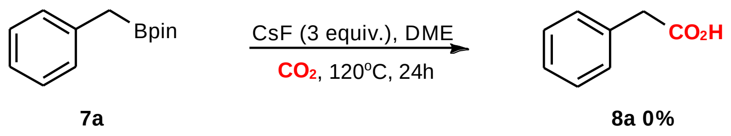

(B) Control experiment on $LiBH_4$-catalysed hydroboration using catecholborane

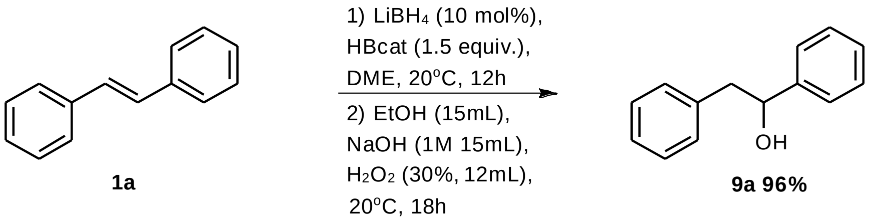

(C) CsF-mediated hydrocarboxylation using catecholborane

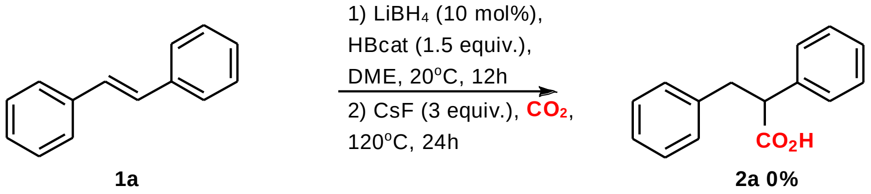

Scheme S4. Control experiments on benzylboronic esters.

Carboxylation of enantiomerically enriched organoborane

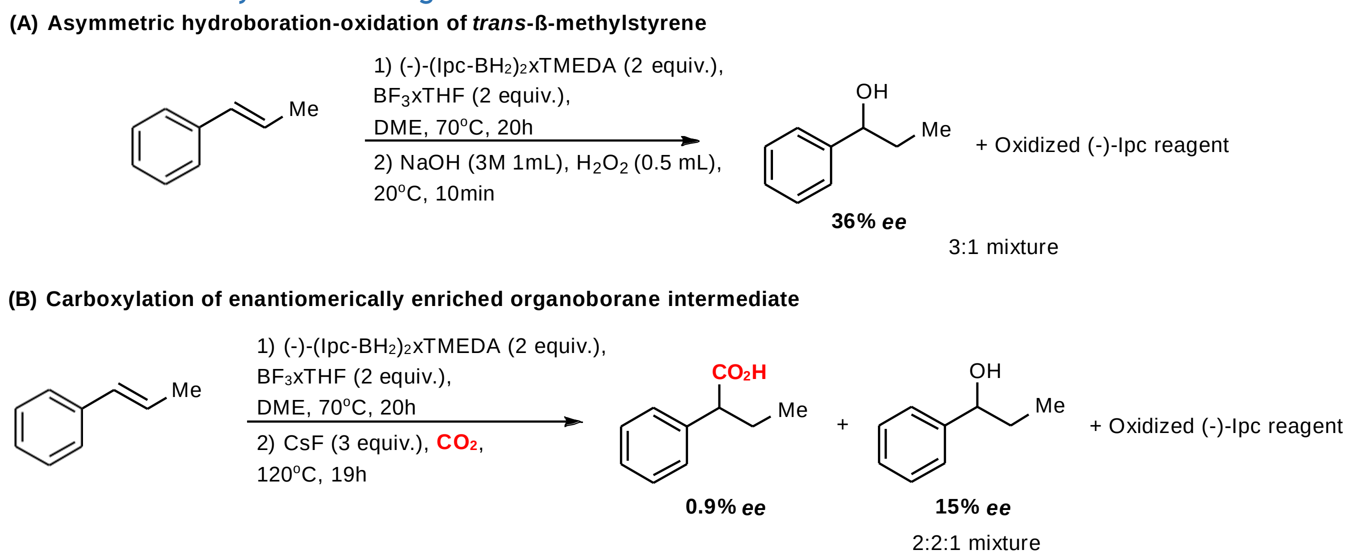

Scheme S5. Asymmetric hydroboration-oxidation of *trans*-β-methylstyrene (A) and carboxylation of enantiomerically enriched organoborane intermediate (B).

Setup of the reaction

Figure S1. 45mL pressure tube with suitable stabilizer and septa.

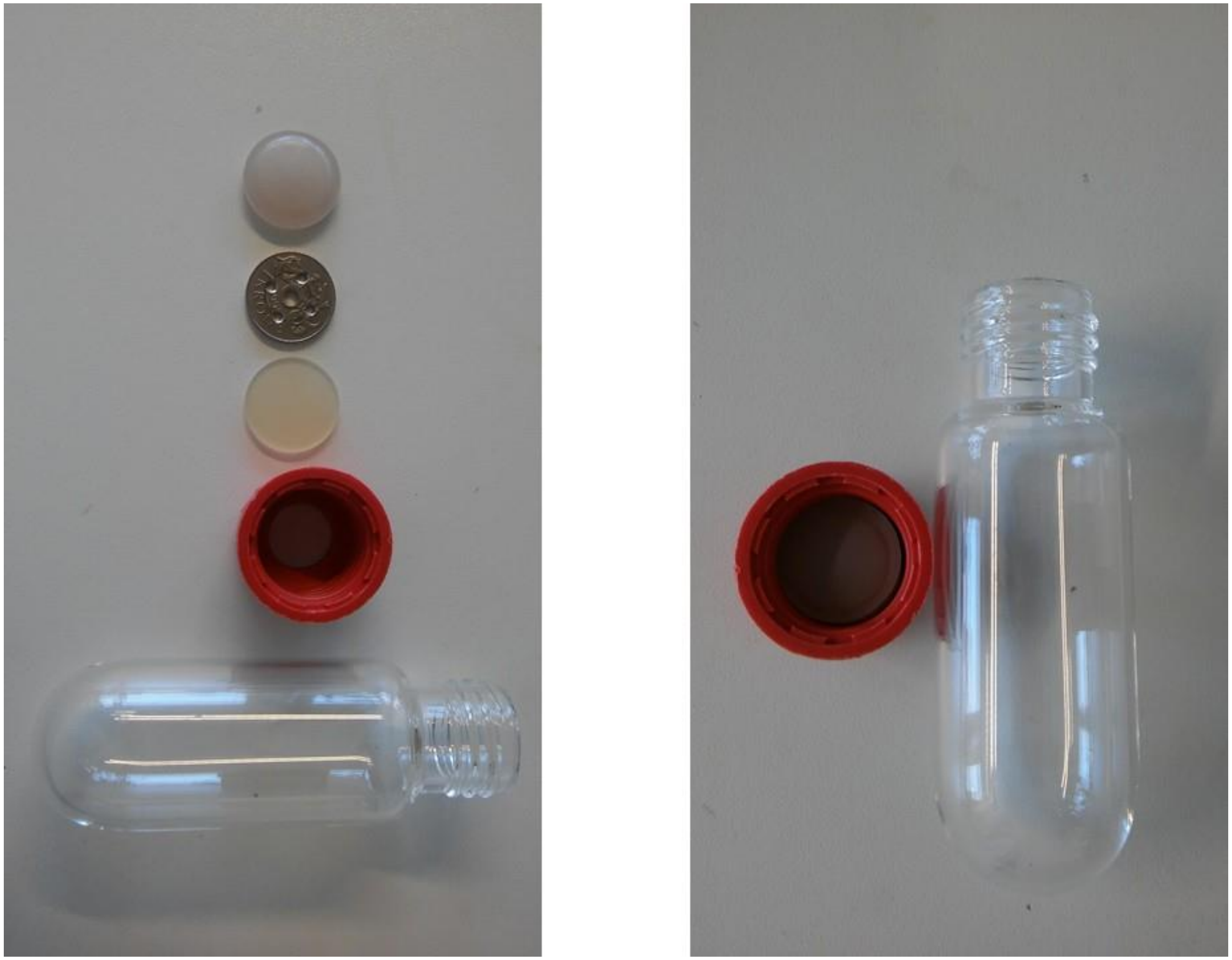

Figure S2. 45mL pressure tube with suitable stabilizer and septa.

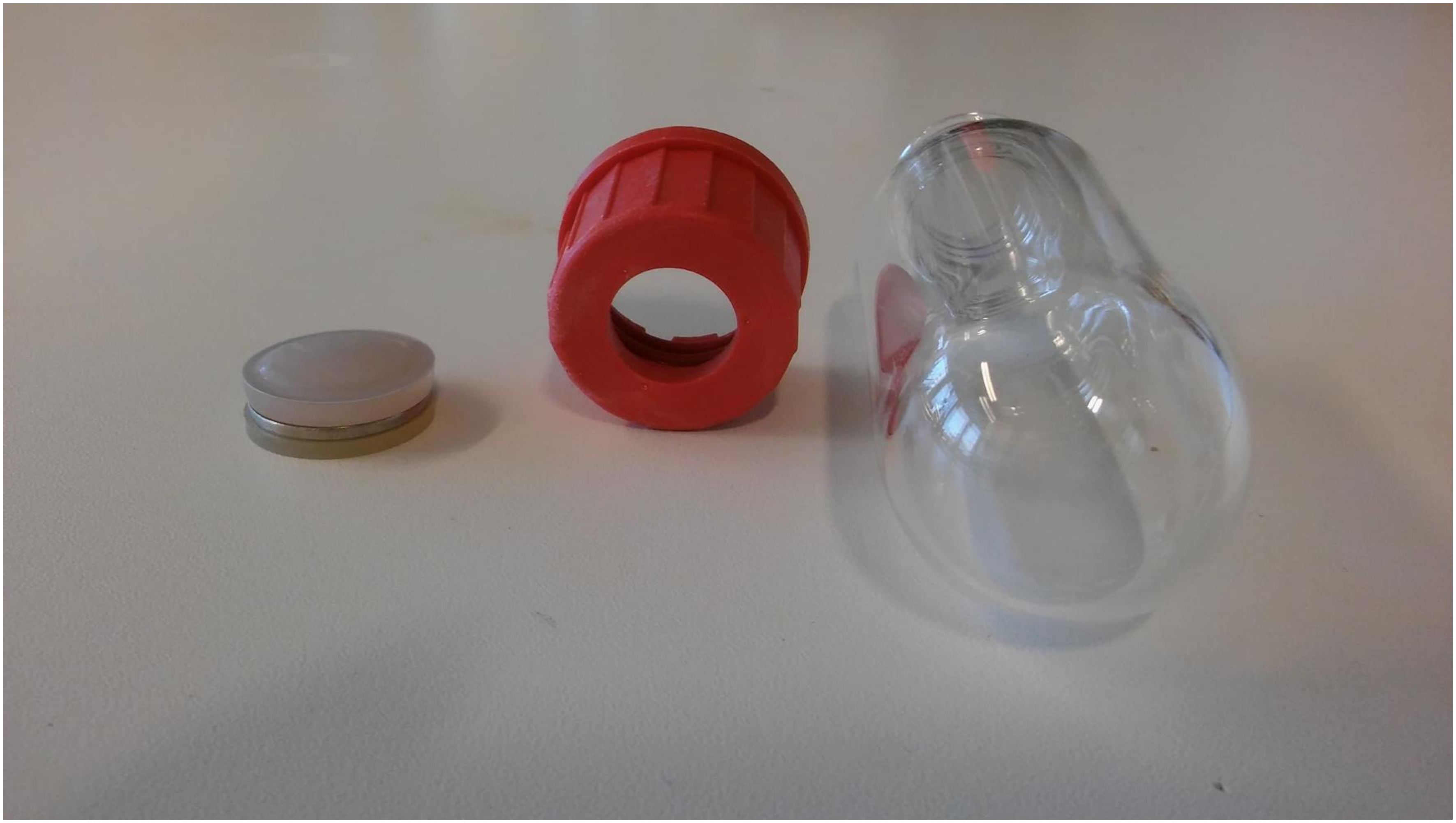

Figure S3. Syringe with CO_2 ready for injection.

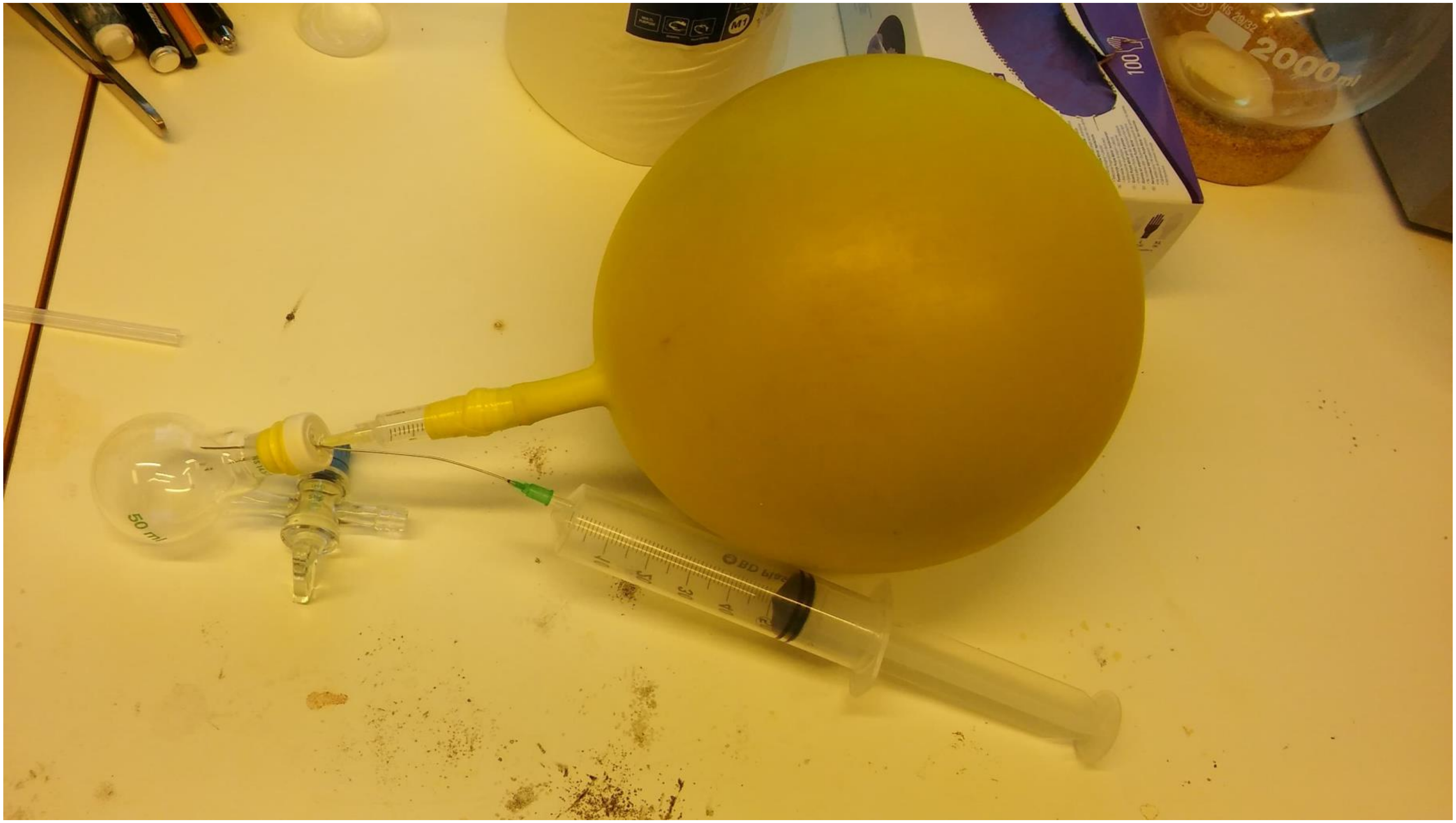

Figure S4. First injection of CO_2.

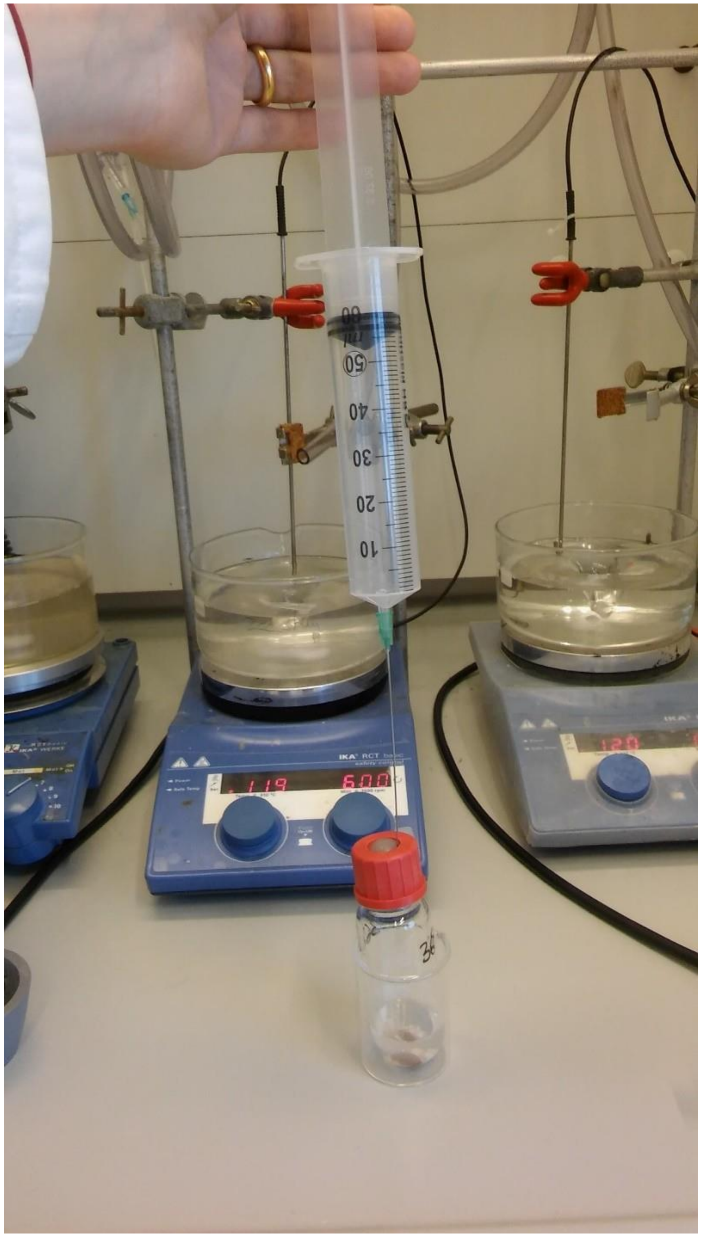

Figure S5. Gram scale hydrocarboxylation in diglyme.

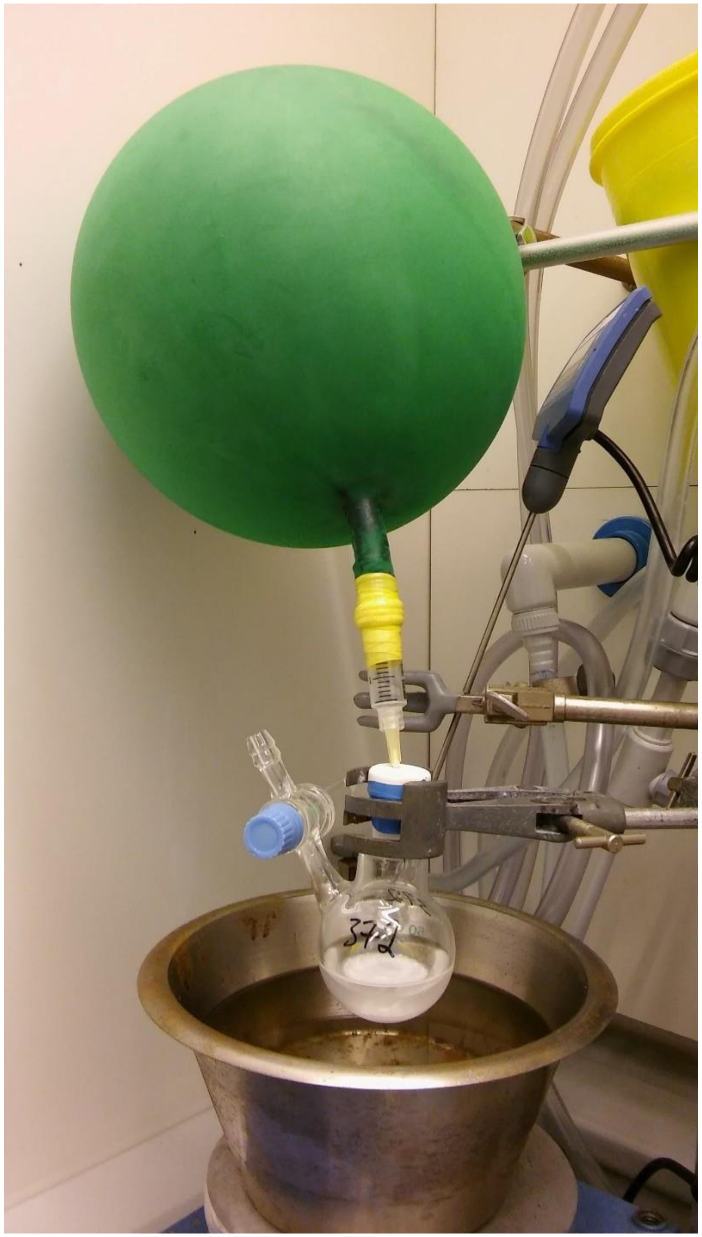

General procedures

Preparation of starting materials for 2e, 3b, 3c, 3d and 6b by Wittig reaction.

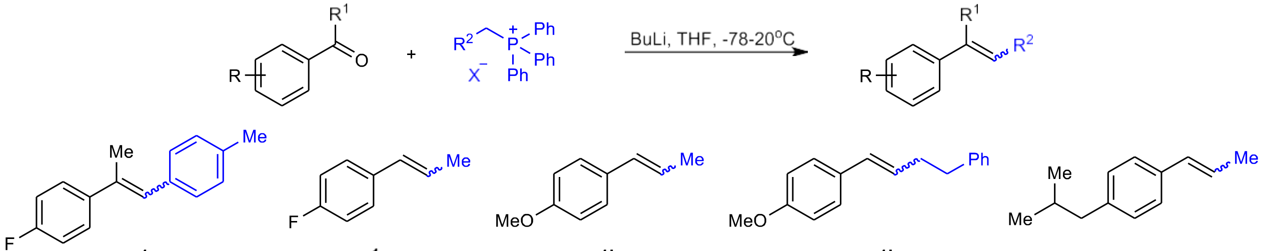

1e 1g 1h 1i 1m

50mL round bottom flask was charged with phosphonium salt (1.2 equiv.), sealed with a rubber septa, evacuated and back filled with Ar. Afterwards, an Ar balloon was added to the system followed by addition of dry THF (15 mL). The reaction mixture was transferred into isopropanol bath (-78°C), which was followed by addition of BuLi (2.5M in hexanes, 1.2 equiv., dropped). The reaction mixture was allowed to reach 20°C. Next, it was transferred back to the isopropanol bath (-78°C), which was followed by addition of carbonyl compound (6 mmol, solid carbonyl compounds were dissolved in 4-6 mL dry THF before addition). Further, the reaction mixture was allowed to reach 20°C where it was stirred for 12h. The formed mixture was evaporated to dryness and purified by column chromatography.

Preparation of starting material for 3e.

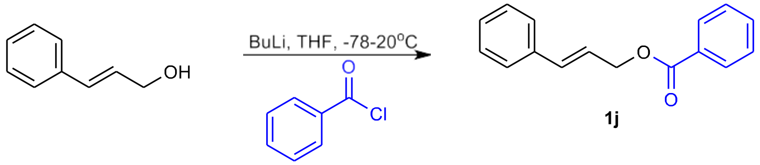

1j

50mL round bottom flask was charged with cinnamyl alcohol (7.5 mmol), sealed with a rubber septa, evacuated and beck filled with Ar. An Ar balloon was added to the system, which was followed by addition of dry THF (15 mL). The reaction mixture was transferred into isopropanol bath (-78°C), which was followed by addition of BuLi (2.5M in hexanes, 1 equiv., dropped). The reaction mixture was allowed to reach 20°C. Afterwards, the reaction mixture was transferred back to the isopropanol bath (-78°C) that was followed by addition of benzoyl chloride (1.2 equiv., dropped). The reaction mixture was allowed to reach 20°C and was stirred at 20°C for 6h. Afterwards, the reaction mixture was evaporated to dryness and purified by column chromatography.

Preparation of starting materials for 5d-f.[1]

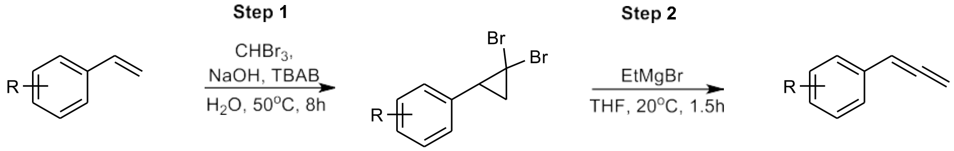

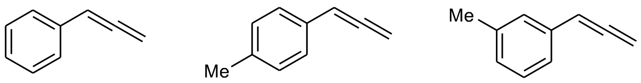

4d **4e** **4f**

Step 1: To the stirring mixture of corresponding styrene (50 mmol), bromoform (2 equiv.), and TBAB (0.1 equiv.) in 50 mL round bottom flask was added sodium hydroxide (2 equiv.) in water (7.5 mL, dropped). The resulting mixture was stired at 50°C for 8h. Next, the reaction was quenched with brine (50 mL) and extracted with DCM (3 × 50 mL). Combined organic fractions were washed with brine (50 mL), evaporated to dryness and used for the next step without purification.
Step 2: To a stirred solution of gem-dibromocyclopropane in dry THF (40 mL), was added EtMgBr (1.1 equiv., 1M in THF). The reaction mixture was stirred at 20° C for 1.5h. Next, the reaction was quenched with brine (10 mL) that was followed by addition of 6M HCl (50 mL) and extraction using Et_2O (3 × 50 mL). The resulting solution of Et_2O was dried (Na_2SO_4) and evaporated. The resulting mixture was purified by column chromatography.

General procedure for metal-free hydrocarboxylation of stilbenes and β-substituted styrenes. For general setup, see Figure S1-S4.

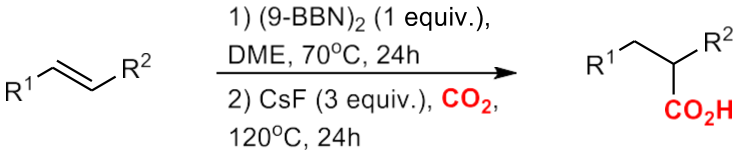

Inside of glove box 45 mL pressure tube was charged with corresponding olefin (1.5 mmol), $(9\text{-BBN})_2$ (1 equiv.) and dry DME (7 mL). The flask was closed with suitable cap, removed from the glove box and heated to 70°C for 24h. Afterwards, the pressure tube was transferred back to the glove box. To the reaction mixture at 20°C was added CsF (3 equiv.). The pressure tube was closed with the cap and removed from the glove box. Afterwards CO_2 (120 mL) was added *via* a syringe, which was followed by stirring of reaction mixture at 120°C for 24h. Next, the reaction mixture was diluted with 30 mL Et_2O and transferred into 500 mL separating funnel. The resulting mixture was extracted with 30 mL saturated basic ($NaHCO_3$, 1M KOH) solution (3 times). Resulting basic solution was washed with 15 mL Et_2O (once), acidified (50-55 mL 6M HCl) and extracted with 30 mL Et_2O (3 times). The resulting solution of Et_2O was distilled to dryness to give corresponding acid.

---

[1] K. Semba, M. Shinomiya, T. Fujihara, J. Terao and Y. Tsuji, *Chem. Eur. J.*, 2013, **19**, 7125-7132.

General procedure for metal-free hydrocarboxylation of allenes. For general setup, see Figure S1-S4.

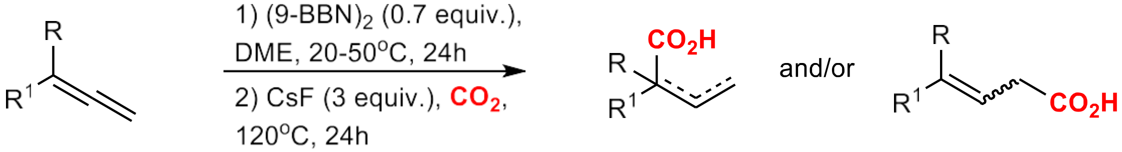

Inside of glove box 45 mL pressure tube was charged with corresponding allene (1.5 mmol), $(9\text{-BBN})_2$ (0.7 equiv.) and dry DME (7 mL). The flask was closed with suitable cap, removed from the glove box and heated to 50ºC for 24h (in case of **5c** this step was runned at 20ºC). Afterwards, the pressure tube was transferred back to the glove box. To the reaction mixture at 20ºC was added CsF (3 equiv.). The pressure tube was closed with the cap and removed from the glove box. Afterwards CO_2 (120 mL) was added *via* a syringe, which was followed by stirring of reaction mixture at 120ºC for 24h. Next, the reaction mixture was diluted with 30 mL Et_2O and transferred into 500 mL separating funnel. The resulting mixture was extracted with 30 mL saturated basic ($NaHCO_3$, 1M KOH) solution (3 times). Resulting basic solution was washed with 15 mL Et_2O (once), acidified (50-55 mL 6M HCl) and extracted with 30 mL Et_2O (3 times). The resulting solution of Et_2O was distilled to dryness to give corresponding acid (in case of **5c** the final solution of Et_2O was dried using Na_2SO_4, which was followed by careful evaporation of solvents).

General procedure for preparation of Butetamate.

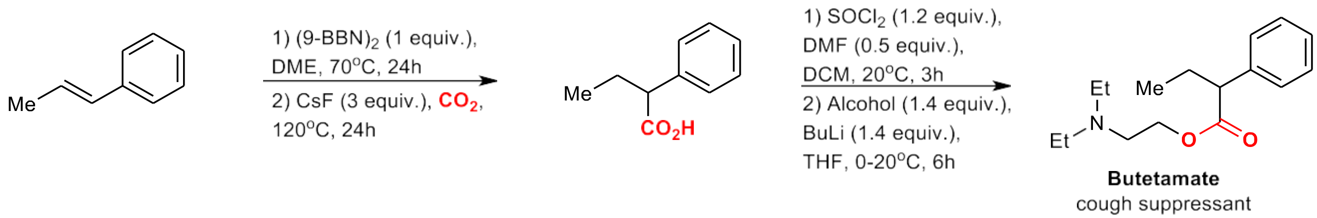

Inside of glove box 45 mL pressure tube was charged with *trans*-β-methylstyrene (1.5 mmol), $(9\text{-BBN})_2$ (1 equiv.) and dry DME (7 mL). The flask was closed with suitable cap, removed from the glove box and heated to 70ºC for 24h. Afterwards, the pressure tube was transferred back to the glove box. To the reaction mixture at 20ºC was added CsF (3 equiv.). The pressure tube was closed with the cap and removed from the glove box. Afterwards CO_2 (120 mL) was added *via* a syringe, which was followed by stirring of reaction mixture at 120ºC for 24h. Next, the reaction mixture was diluted with 30 mL Et_2O and transferred into 500 mL separating funnel. The resulting mixture was extracted with 30 mL saturated basic ($NaHCO_3$, 1M KOH) solution (3 times). Resulting basic solution was washed with 15 mL Et_2O (once), acidified (50-55 mL 6M HCl) and extracted with 30 mL Et_2O (3 times). The resulting solution of Et_2O was distilled to dryness to give corresponding acid.

An oven dried 25mL Schlenk flask was charged with the acid, sealed with rubber septa, evacuated and beck filled with Ar (3 times). Next, an Ar balloon was added to the system, which was followed by addition of dry DCM (4 mL), dry DMF (0.5 equiv) and $SOCl_2$ (1.2 equiv., dropwise). The reaction mixture was stirred at 20°C for 3h that was followed by removal of volatiles and addition of dry THF (4 mL). To the resulting mixture at 0°C was added (dropwise) previously prepared solution of corresponding alkoxide (at 0°C, under Ar atmosphere to the solution of 2-(diethylamino)ethanol (1.4 equiv.) in dry THF (4 mL) was added BuLi (2.5M in hexanes, 1.4 equiv., dropped), which was followed by stirring of the reaction mixture at 20°C for 30 min). The resulting mixture was stirred at 20°C for 6h, evaporated to dryness and purified using column chromatography.

General procedure for preparation of Butibufen.

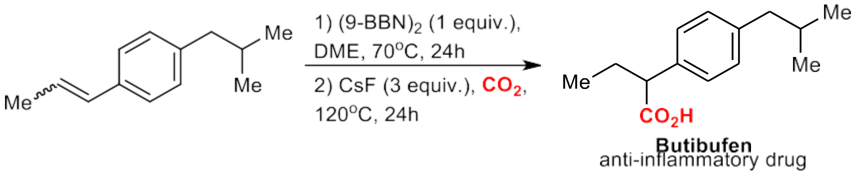

Inside of glove box 45 mL pressure tube was charged with corresponding olefin (1.148 mmol), $(9\text{-BBN})_2$ (1 equiv.) and dry DME (5 mL). The flask was closed with suitable cap, removed from the glove box and heated to 70°C for 24h. Afterwards, the pressure tube was transferred back to the glove box. To the reaction mixture at 20°C was added CsF (3 equiv.). The pressure tube was closed with the cap and removed from the glove box. Afterwards CO_2 (120 mL) was added *via* a syringe, which was followed by stirring of reaction mixture at 120°C for 24h. Next, the reaction mixture was diluted with 30 mL Et_2O and transferred into 500 mL separating funnel. The resulting mixture was extracted with 30 mL saturated basic ($NaHCO_3$, 1M KOH) solution (3 times). Resulting basic solution was washed with 15 mL Et_2O (once), acidified (50-55 mL 6M HCl) and extracted with 30 mL Et_2O (3 times). The resulting solution of Et_2O was distilled to dryness to give corresponding acid.

General procedure for gram scale hydrocarboxylation of *trans*-stilbene. For general setup, see Figure S5.

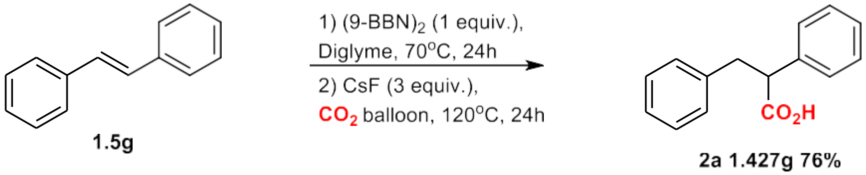

Inside of glove box 50 mL Schlenk flask was charged with *trans*-stilbene (1.5 g, 8.322 mmol), $(9\text{-BBN})_2$ (1 equiv.) and dry Diglyme (18 mL). The flask was closed with rubber septa, removed from the glove box and heated to 70° C for 24h. Afterwards, the flask was transferred back to the glove box. To the reaction mixture at 20°C was added CsF (3.792 g.). The flask was closed with rubber septa and removed from the glove box. Next, the flask was carefully evacuated (once), which was followed by addition of CO_2 balloon. The reaction mixture was stirred

at 120ºC for 24h. Further, the reaction mixture was diluted with 90 mL Et_2O and transferred into 1000 mL separating funnel. The resulting mixture was extracted with 90 mL saturated basic ($NaHCO_3$, 1M KOH) solution (3 times). Resulting basic solution was washed with 45 mL Et_2O (once), acidified (160 mL 6M HCl) and extracted with 90 mL Et_2O (3 times). The resulting solution of Et_2O was washed with 30 mL distilled water (3 times) and evaporated to dryness to give corresponding acid.

General procedure for Cu-catalysed hydrocarboxylation of indene and 1,2-dihydronaphthalene. For general setup, see Figure S1-S4.

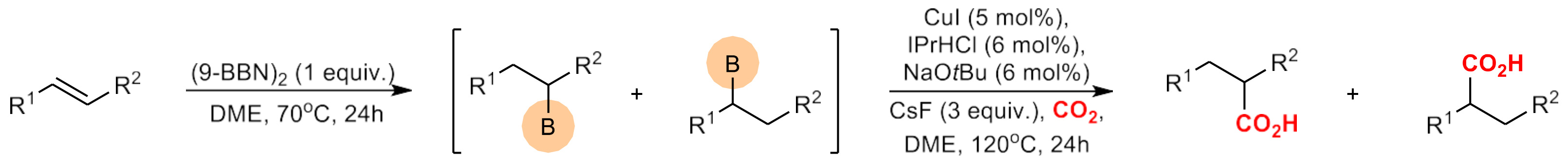

Inside of glove box 45 mL pressure tube was charged with corresponding olefin (1.5 mmol), $(9\text{-BBN})_2$ (1 equiv.) and dry DME (5 mL). The flask was closed with suitable cap, removed from the glove box and heated to 70ºC for 24h. Afterwards, the pressure tube was transferred back to the glove box. To the reaction mixture at 20ºC was added CsF (3 equiv.) and previously prepared solution of catalyst (the mixture of CuI (5 mol%), IPrHCl (6 mol%) and NaO*t*Bu (6 mol%) in dry DME (2 mL) was stirred at 20ºC for 30 min). The pressure tube was closed with the cap and removed from the glove box. Afterwards CO_2 (120 mL) was added *via* a syringe, which was followed by stirring of reaction mixture at 120ºC for 24h. Next, the reaction mixture was diluted with 30 mL Et_2O and transferred into 500 mL separating funnel. The resulting mixture was extracted with 30 mL saturated basic ($NaHCO_3$, 1M KOH) solution (3 times). Resulting basic solution was washed with 15 mL Et_2O (once), acidified (50-55 mL 6M HCl) and extracted with 30 mL Et_2O (3 times). The resulting solution of Et_2O was distilled to dryness to give corresponding acid.

Control experiment on Cu-catalysed decarboxylation.

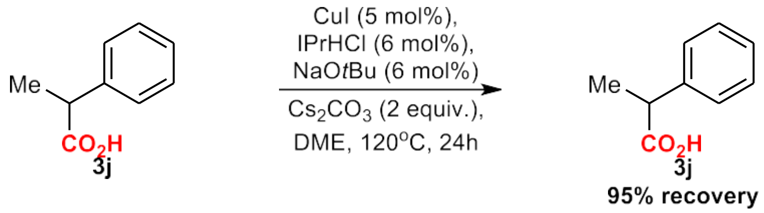

Inside of glove box 45 mL pressure tube was charged with 2-phenylpropionic acid (1.5 mmol), DME (5 mL), Cs_2CO_3 (2 equiv.) and previously prepared solution of catalyst (the mixture of CuI (5 mol%), IPrHCl (6 mol%) and NaO*t*Bu (6 mol%) in dry DME (2 mL) was stirred at 20ºC for 30 min). The pressure tube was closed with the cap and removed from the glove box, which was followed by stirring of reaction mixture at 120ºC for 24h. Next, the reaction mixture was diluted with 30 mL Et_2O and transferred into 500 mL separating funnel. The resulting mixture

was extracted with 30 mL saturated basic ($NaHCO_3$, 1M KOH) solution (3 times). Resulting basic solution was washed with 15 mL Et_2O (once), acidified (50-55 mL 6M HCl) and extracted with 30 mL Et_2O (3 times). The resulting solution of Et_2O was distilled to dryness to give corresponding acid.

Control experiment on Cu-free decarboxylation.

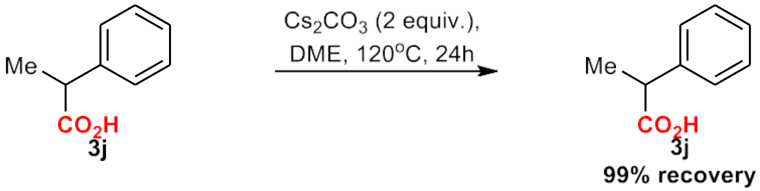

Inside of glove box 45 mL pressure tube was charged with 2-phenylpropionic acid (1.5 mmol), DME (5 mL) and Cs_2CO_3 (2 equiv.). The pressure tube was closed with the cap and removed from the glove box, which was followed by stirring of reaction mixture at 120°C for 24h. Next, the reaction mixture was diluted with 30 mL Et_2O and transferred into 500 mL separating funnel. The resulting mixture was extracted with 30 mL saturated basic ($NaHCO_3$, 1M KOH) solution (3 times). Resulting basic solution was washed with 15 mL Et_2O (once), acidified (50-55 mL 6M HCl) and extracted with 30 mL Et_2O (3 times). The resulting solution of Et_2O was distilled to dryness to give corresponding acid.

Control experiment on $LiBH_4$-catalysed hydroboration using catecholborane.

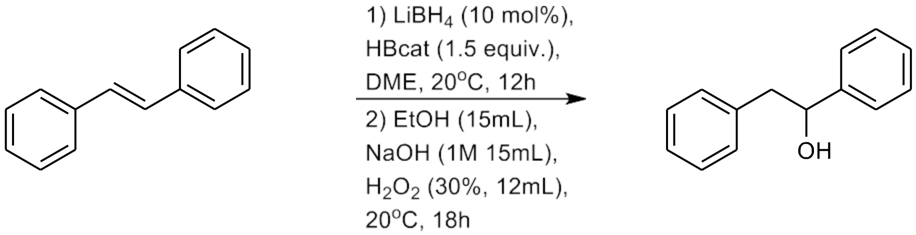

Inside of glove box 25 mL round bottom flask was charged with *trans*-stilbene (1.110 mmol), catecholborane (1.5 equiv.), DME (6 mL) and $LiBH_4$ (10 mol%). The flask was closed with a rubber septa, removed from the glove box and stirred at 20°C for 12h. Afterwards, the reaction mixture was transferred into 250 mL round bottom flask. To the mixture sequentially were added EtOH (15 mL), 1M NaOH (15 mL) and aq. H_2O_2 (30%, 12 mL). The resulting mixture was stirred at 20°C for 18h. Then it was quenched with aq. $Na_2S_2O_3$ (10%, 60 mL). This was followed by extraction using 40 mL DCM (3 times). The organic fractions were collected, evaporated to dryness and purified using column chromatography.

Asymmetric hydroboration-oxidation of *trans*-ß-methylstyrene.

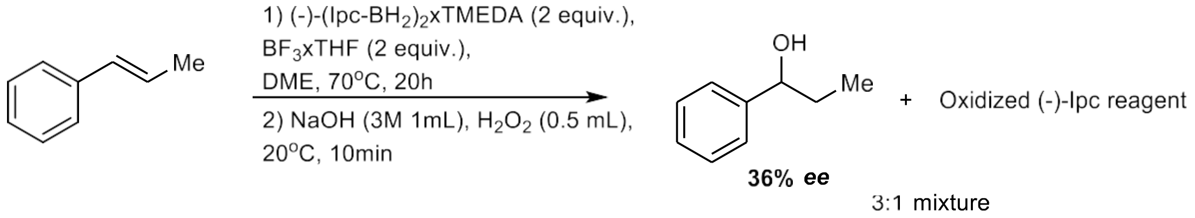

Inside of glove box, a 45 mL pressure tube was charged with (-)-isopinocampheylborane TMEDA complex ((-)-($Ipc\text{-}BH_2)_2$xTMEDA) (1.0 mmol) and BF_3xTHF (1.0 mmol) in dry DME (2 mL). The flask was closed with suitable cap, removed from the glove box and stirred for 1h. *Trans*-beta-methylstyrene (0.5 mmol) was added with syringe and heated to 70°C for 20h. To the intermediate borane compound was added 1 mL 3M NaOH and 0.5 mL H_2O_2 and reacted 10 min. The phases were allowed to settle and the organic phase was removed. The aqueous phase was extracted with 1 mL EtOAc and the organic phase was filtered through a pad of Na_2SO_4 and evaporated. The residue was purified by automated flash chromatography on a 10g Biotage SNAP column with a 0-30% EtOAc in heptane gradient. The resulting blank oil is 1-phenylpropanol in an approximate 3:1 mixture with oxidized (-)-Ipc reagent shown by NMR. Chiral SFC was performed using Waters Trefoil CEL2 (2.5 µm, 3.0 x 150 mm), backpressure: 1500 psi, column temperature 40°C, mobile phase B: 1:1:1 EtOH:*i*PrOH:MeCN with 20 mM NH_4OAc. Gradient 1-6 % 4.5 min, 6 % 1.5 min. The 1-phenylpropanol has an *ee* of 36% and the (-)-Ipc reagent is invisible by UV detection.

Carboxylation of enantiomerically enriched organoborane intermediate.

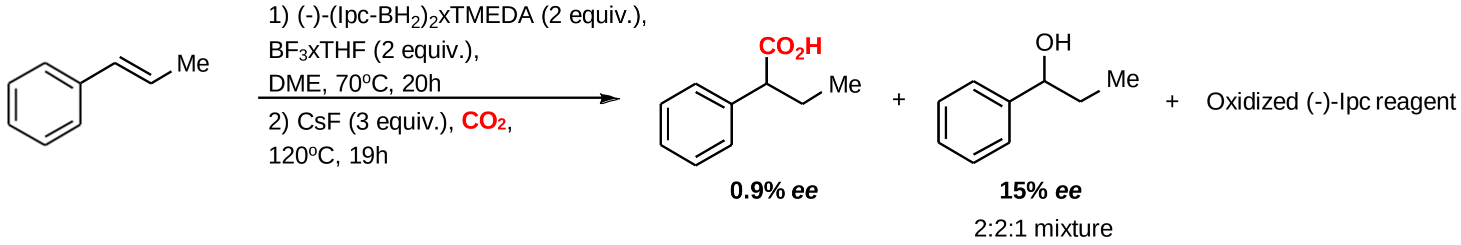

Inside of glove box, a 45 mL pressure tube was charged with (-)-isopinocampheylborane TMEDA complex ((-)-($Ipc\text{-}BH_2)_2$xTMEDA) (1.0 mmol) and BF_3xTHF (1.0 mmol) in dry DME (2 mL). The flask was closed with suitable cap, removed from the glove box and stirred for 1h. *Trans*-beta-methylstyrene (0.5 mmol) was added with syringe and heated to 70°C for 20h. The pressure tube was then transferred back to the glove box where at 20°C was added CsF (3 equiv.). The pressure tube was closed with the cap and removed from the glove box. Afterwards CO_2 (120 mL) was added *via* a syringe, which was followed by stirring of reaction mixture at 120°C for 19h. Next, the reaction mixture was diluted with 30 mL Et_2O and transferred into a 250 mL separating funnel. The resulting mixture was extracted with 15 mL 1M KOH solution (3 times). The resulting basic solution was washed with 30 mL Et_2O, acidified (30 mL 2M HCl) and extracted with 15 mL Et_2O (3 times). The resulting solution of Et_2O was dried with Na_2SO_4 and distilled to dryness.

The acid base extraction after carboxylation yields an approximate 2:2:1 ratio of 2-phenylbutyric acid, 1-phenylpropanol and oxidized (-)-Ipc reagent, determined by NMR. SFC was performed with Waters Trefoil CEL1 (2.5 µm, 3.0 x 150 mm), backpressure: 1500 psi, column temperature 40°C, mobile phase B: 1:1 MeOH:*i*PrOH with 20 mM TFA. Gradient 1-10 % 4.5 min, 10 % 1.5 min. The 2-phenylbutyric acid has 0.9% *ee* and the 1-phenylpropanol has 15% *ee* (favoring the same enantiomer as for the oxidized intermediate). Note that the retention of 2-phenylbutyric acid varies between runs with up to 0.5 min.

Characterization of products

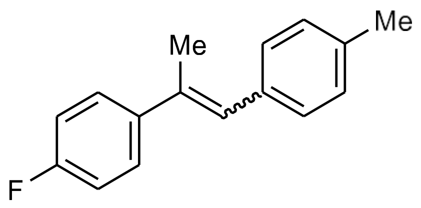

***1-Fluoro-4-(1-(p-tolyl)prop-1-en-2-yl)benzene*, mixture of stereoisomers, 1e.** Starting from 10.86 mmol of corresponding carbonyl compound the product was obtained as a colourless viscous oil as a 1:1 mixture of isomers, yield 99% (2.444 g). **^{1}H NMR** (400 MHz, $CDCl_3$): δ = 2.18 (d, 1.5H, *J* = 1.6 Hz, Me), 2.26-2.27 (m, 3H, Me), 2.38 (s, 1.5H, Me), 6.45-6.46 (m, 0.45H, olefin), 6.76 (s, 0.49H, olefin), 6.84 (d, *J* = 8.1 Hz, 1H, Ar), 6.92-7.00 (m, 2H, Ar), 7.03-7.09 (m, 1H, Ar), 7.13-7.21 (m, 2H, Ar), 7.24-7.28 (m, 1H, Ar), 7.46-7.51 (m, 1H, Ar).
^{13}C NMR (101 MHz, $CDCl_3$): δ = 17.8, 21.3, 21.4, 27.2, 115.1, 115.3, 115.4, 115.6, 127.0, 127.6, 127.7, 127.8, 128.9, 129.0, 129.1, 129.2, 130.0, 130.1, 134.7, 135.5, 135.9, 136.1, 136.5, 136.8.

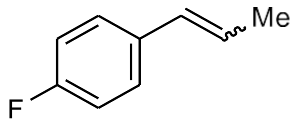

***1-Fluoro-4-(prop-1-en-1-yl)benzene*, mixture of stereoisomers, 1g.**[2] Starting from 12.09 mmol of corresponding carbonyl compound the product was obtained as a colorless liquid as a 1:3 mixture of isomers, yield 90% (1.487 g). **^{1}H NMR** (400 MHz, $CDCl_3$): δ = 1.88-1.91 (m, 3H, Me), 5.80 (dq, *J* = 11.6, 7.2 Hz, 0.27H, olefin), 6.17 (dq, *J* = 15.7, 6.6 Hz, 0.74H, olefin), 6.36-6.43 (m, 1H, olefin), 6.97-7.07 (m, 2H, Ar), 7.26-7.33 (m, 2H, Ar). **^{13}C NMR** (101 MHz, $CDCl_3$): δ = 14.7, 18.6, 115.0, 115.3, 115.4, 115.6, 125.5, 125.6, 126.8, 127.3, 127.4, 129.0, 130.0, 130.5, 130.6, 134.3, 160.8, 163.3.

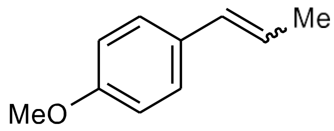

***1-Methoxy-4-(prop-1-en-1-yl)benzene*, mixture of stereoisomers, 1h.**[3] Starting from 11.02 mmol of corresponding carbonyl compound the product was obtained as a colorless liquid as a 1:9 mixture of isomers, yield 94% (1.534 g). **^{1}H NMR** (400 MHz, $CDCl_3$): δ = 1.85-1.91 (m, 3H, Me), 3.80-3.82 (m, 3H, OMe), 5.71 (dq, *J* = 11.5, 7.2 Hz, 0.11H, olefin), 6.10 (dq, *J* = 15.7, 6.6 Hz, 0.9H, olefin), 6.33-6.40 (m, 1H, olefin), 6.82-6.90 (m, 2H, Ar), 7.24-7.35 (m, 2H, Ar). **^{13}C NMR** (101 MHz, $CDCl_3$): δ = 18.6, 55.5, 113.7, 114.1, 123.7, 127.1, 130.2, 130.5, 131.0, 158.8.

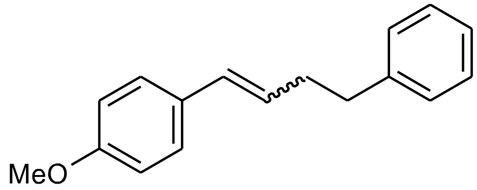

***1-Methoxy-4-(4-phenylbut-1-en-1-yl)benzene*, mixture of stereoisomers, 1i.**[4] Starting from 5.876 mmol of corresponding carbonyl compound the product was obtained as a colourless viscous oil as a 1:4 mixture of isomers, yield 83% (1.162 g). **^{1}H NMR** (400 MHz, $CDCl_3$): δ = 2.51-2.58 (m, 1.57H, CH_2), 2.66-2.73 (m, 0.46H, CH_2), 2.79-2.84 (m, 2H, CH_2), 3.82-3.83 (m, 3H, OMe), 5.65 (dddd, *J* = 11.5, 8.8, 7.0, 2.3 Hz, 0.22H, olefin), 6.11-6.19 (m, 0.78H, olefin), 6.37-6.42 (m, 1H, olefin), 6.85-

[2] L. Zheng, F. Gao, C. Yang, G.-L. Gao, Y. Zhao, Y. Gao and W. Xia, *Org. Lett.* 2017, **19**, 5086-5089.
[3] I. R. Green and N. October, *ARKIVOC* 2010, **2**, 71-96.
[4] S. Hajra, B. Maji and D. Mal, *Adv. Synth. Catal.* 2009, **351**, 859-864.

6.90 (m, 2H, Ar), 7.21-7.35 (m, 7H, Ar). ^{13}**C NMR** (101 MHz, $CDCl_3$): δ = 30.6, 35.1, 36.2, 36.3, 55.4, 113.8, 114.1, 126.0, 126.1, 127.2, 128.0, 128.5, 128.6, 129.0, 129.9, 130.1, 130.4, 130.7, 142.1, 158.9.

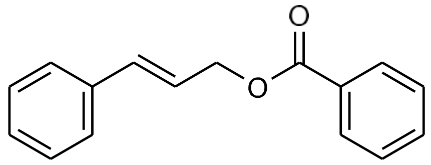

***Cinnamyl benzoate,* 1j.**[5] Starting from 7.453 mmol of cinnamyl alcohol the product was obtained as a colourless viscous oil, yield 95% (1.682 g). ^{1}**H NMR** (400 MHz, $CDCl_3$): δ = 4.78 (dd, *J* = 6.4, 1.4 Hz, 2H, CH_2), 6.20 (dt, *J* = 15.9, 6.4 Hz, 1H, olefin), 6.54 (d, *J* = 15.9 Hz, 1H, olefin), 7.04-7.08 (m, 1H, Ar), 7.11-7.14 (m, 2H, Ar), 7.20-7.26 (m, 4H, Ar), 7.33-7.38 (m, 1H, Ar), 7.87-7.89 (m, 2H, Ar). ^{13}**C NMR** (101 MHz, $CDCl_3$): δ = 65.7, 123.5, 126.8, 128.3, 128.5, 128.6, 128.8, 129.7, 129.8, 130.4, 133.2, 134.4, 136.4, 166.6.

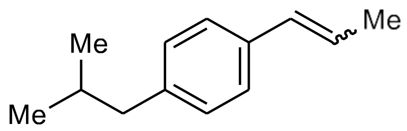

***1-Isobutyl-4-(prop-1-en-1-yl)benzene,* mixture of stereoisomers, 1m.**[6] Starting from 9.246 mmol of corresponding carbonyl compound the product was obtained as a colorless liquid, yield 94% (1.511 g). ^{1}**H NMR** (400 MHz, $CDCl_3$): δ = 0.94-0.97 (m, 6H, 2xMe), 1.84-1.97 (m, 4H, Me, CH), 2.48-2.52 (m, 2H, CH_2), 5.80 (dq, *J* = 11.5, 7.2 Hz, 0.21H, olefin), 6.24 (dq, *J* = 15.7, 6.6 Hz, 0.79H, olefin), 6.40-6.47 (m, 1H, olefin), 7.10-7.17 (m, 2H, Ar), 7.26-7.30 (m, 2H, Ar). ^{13}**C NMR** (101 MHz, $CDCl_3$): δ = 14.9, 18.6, 22.5, 22.6, 30.4, 45.3, 124.8, 125.7, 126.2, 128.8, 129.1, 129.4, 130.0, 131.1, 135.6, 140.5.

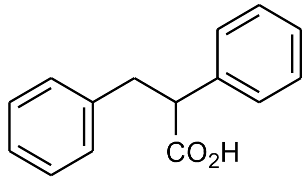

***2,3-Diphenylpropanoic acid,* 2a.**[7] Starting from 0.444 mmol of corresponding stilbene the product was obtained as a white solid, yield 87% (0.087 g), m.p. = 77-80°C. ^{1}**H NMR** (400 MHz, $CDCl_3$): δ = 3.13 (dd, *J* = 13.9, 7.0 Hz, 1H, CH_2), 3.50 (dd, *J* = 13.8, 8.4 Hz, 1H, CH_2), 3.95 (dd, *J* = 8.4, 7.0 Hz, 1H, CH), 7.17-7.21 (m, 2H, Ar), 7.24-7.33 (m, 3H, Ar), 7.34-7.41 (m, 5H, Ar), 11.45 (br s, 1H, CO_2H). ^{13}**C NMR** (101 MHz, $CDCl_3$): δ = 39.4, 53.6, 126.6, 127.8, 128.3, 128.5, 128.9, 129.1, 138.1, 138.8, 179.7. **HRMS-EI** (m/z) $[M-H]^-$ calcd. for $C_{15}H_{13}O_2$ 225.0921 found 225.0923.

[5] C.-T. Chen, J.-H. Kuo, V. D. Pawar, Y. S. Munot, S.-S. Weng, C.-H. Ku and C.-Y. Liu, *J. Org. Chem.* 2005, **70**, 1188-1197.
[6] S. Movahhed, J. Westphal, M. Dindaroglu, A. Falk and H.-G. Schmalz, *Chem. Eur. J.* 2016, **22**, 7381-7384.
[7] M. Juhl, S. L. R. Laursen, Y. Huang, D. U. Nielsen, K. Daasbjerg and T. Skrydstrup, *ACS Catal.* 2017, **7**, 1392-1396.

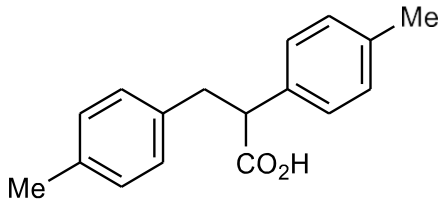

2,3-Di-p-tolylpropanoic acid, **2b.**[8] Starting from 0.336 mmol of corresponding stilbene the product was obtained as a white solid, yield 91% (0.078 g), m.p. = 138-140°C. **^{1}H NMR** (400 MHz, $CDCl_3$): δ = 2.31 (d, *J* = 14.7 Hz, 6H, 2xMe), 2.98 (dd, *J* = 13.9, 6.8 Hz, 1H, CH_2), 3.35 (dd, *J* = 13.9, 8.5 Hz, 1H, CH_2), 3.81 (dd, *J* = 8.5, 6.8 Hz, 1H, CH), 7.00-7.05 (m, 4H, Ar), 7.13 (d, *J* = 7.9 Hz, 2H, Ar), 7.21 (d, *J* = 8.1 Hz, 2H, Ar), 10.25 (br s, 1H, CO_2H). **^{13}C NMR** (101 MHz, $CDCl_3$): δ = 21.2, 21.3, 38.9, 53.2, 128.1, 128.9, 129.2, 129.6, 135.2, 135.9, 136.0, 137.5, 179.5. **HRMS-EI** (m/z) $[M-H]^-$ calcd. for $C_{17}H_{17}O_2$ 253.1229 found 253.1234.

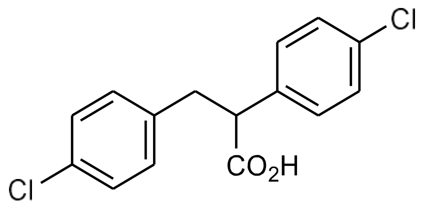

2,3-Bis(4-chlorophenyl)propanoic acid, **2c.**[8] Starting from 0.0803 mmol of corresponding stilbene the product was obtained as a white solid, yield 51% (0.012 g), m.p. > 200°C. **^{1}H NMR** (400 MHz, MeOH-d4): δ = 2.97 (dd, *J* = 13.7, 7.6 Hz, 1H, CH_2), 3.30-3.35 (m, 1H, CH_2), 3.84 (t, *J* = 7.9 Hz, 1H, CH), 7.10-7.12 (m, 2H, Ar), 7.18-7.21 (m, 2H, Ar), 7.26-7.32 (m, 4H, Ar). **^{13}C NMR** (101 MHz, MeOH-d4): δ = 40.0, 54.1, 129.4, 129.7, 130.9, 131.8, 133.3, 134.3, 139.1, 139.2. **HRMS-EI** (m/z) $[M-H]^-$ calcd. for $C_{15}H_{11}Cl_2O_2$ 293.0136 found 293.0142.

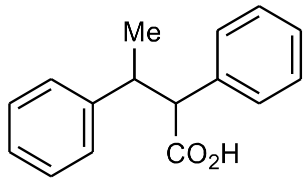

2,3-Diphenylbutanoic acid, **mixture of diastereomers, NMR-ratio 10:1, LC-MS-ratio 85:15, 2d.** Starting from 0.772 mmol of corresponding stilbene the product was obtained as a white solid, yield 70% (0.130 g), m.p. = 128-131°C. **^{1}H NMR** (400 MHz, $CDCl_3$): δ = 1.06 (d, *J* = 6.7 Hz, 0.31H, Me of second diastereomere), 1.48 (d, *J* = 7.0 Hz, 3H, Me), 3.46-3.54 (m, 1H, CH), 3.74-3.80 (m, 1H, CH), 7.00-7.21 (m, 10H, Ar), 7.30-7.51 (m, 0.99H, Ar of second diastereomere), 11.40 (br s, 1H, CO_2H). **^{13}C NMR** (101 MHz, $CDCl_3$): δ = 20.2, 21.3, 43.2, 43.6, 59.1, 59.4, 126.4, 126.9, 127.4, 127.5, 127.7, 128.0, 128.3, 128.4, 128.7, 128.8, 128.9, 129.0, 137.0, 143.4, 179.2, 180.1. **HRMS-EI** (m/z) $[M-H]^-$ calcd. for $C_{16}H_{15}O_2$ 239.1072 found 239.1076.

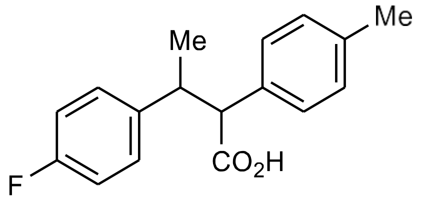

3-(4-Fluorophenyl)-2-(p-tolyl)butanoic acid, **mixture of diastereomers, NMR-ratio 12:1, LC-MS-ratio 92:8, 2e.** Starting from 0.884 mmol of corresponding stilbene the product was obtained as a white solid, yield 55% (0.133 g), m.p. = 148-151°C. **^{1}H NMR** (400 MHz, $CDCl_3$): δ = 1.04 (d, *J* = 6.9 Hz, 0.24H, Me of minor diastereomere), 1.44 (dd, *J* = 6.7, 1.1 Hz, 3H, Me), 2.24 (s, 3H, Me), 2.38 (s, 0.26H, Me of minor diastereomere), 3.45-3.52 (m, 1H, CH), 3.65-3.70 (m, 1H, CH), 6.80-6.84 (m, 2H, Ar), 6.95-6.99 (m, 4H, Ar), 7.05-7.08 (m, 2H, Ar), 7.20-7.37 (m, 0.71H, Ar of minor diastereomere), 11.52 (br s, 1H, CO_2H). **^{13}C NMR** (101 MHz, $CDCl_3$): δ = 20.2, 21.1, 21.3, 21.4, 30.5, 42.4, 42.7, 59.1, 59.3, 71.6, 115.1 (d, *J* = 21.2 Hz), 115.4, 115.6, 128.6, 128.7,

---

[8] J. Dai, W. Ren, J. Li and Y. Shi, *Org. Chem. Front.* 2018, **5**, 561-565.

128.9, 129.1 (d, J = 7.8 Hz), 129.2, 129.7, 133.8, 137.2, 137.8, 139.3 (d, J = 3.3 Hz), 161.4 (d, J = 244.0 Hz), 179.4, 180.2. **HRMS-EI** (m/z) $[M-H]^-$ calcd. for $C_{17}H_{16}FO_2$ 271.1134 found 271.1136.

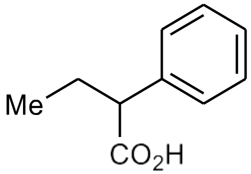

***2-Phenylbutanoic acid,* 3a.**[9] Starting from 1.690 mmol of corresponding β-substituted styrene the product was obtained as a white solid, yield 94% (0.262 g), m.p. = 39-42°C. **^{1}H NMR** (400 MHz, $CDCl_3$): δ = 0.91 (t, J = 7.4 Hz, 3H, Me), 1.76-1.87 (m, 1H, CH_2), 2.06- 2.17 (m, 1H, CH_2), 3.46 (t, J = 7.7 Hz, 1H, CH), 7.25-7.35 (m, 5H, Ar), 10.76 (br s, 1H, CO_2H). **^{13}C NMR** (101 MHz, $CDCl_3$): δ = 12.3, 26.5, 53.5, 127.6, 128.3, 128.8, 138.5. **HRMS-EI** (m/z) $[M-H]^-$ calcd. for $C_{10}H_{11}O_2$ 163.0759 found 163.0788.

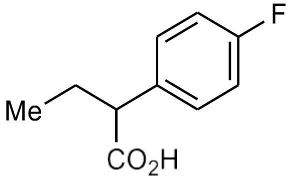

***2-(4-Fluorophenyl)butanoic acid,* 3b.** Starting from 1.470 mmol of corresponding β-substituted styrene the product was obtained as a white solid, yield 75% (0.201 g), m.p. = 54-56°C. **^{1}H NMR** (400 MHz, $CDCl_3$): δ = 0.84 (t, J = 7.4 Hz, 3H, Me), 1.73 (dt, J = 13.7, 7.5 Hz, 1H, CH_2), 2.03 (dt, J = 13.6, 7.4 Hz, 1H, CH_2), 3.39 (t, J = 7.7 Hz, 1H, CH), 6.93-6.99 (m, 2H, Ar), 7.19-7.25 (m, 2H, Ar), 10.13 (br s, 1H, CO_2H). **^{13}C NMR** (101 MHz, $CDCl_3$): δ = 12.2, 26.6, 52.7, 115.7 (d, J = 22.4 Hz), 129.8 (d, J = 7.9 Hz), 134.2 (d, J = 3.2 Hz), 162.4 (d, J = 246.0 Hz), 180.2. **HRMS-EI** (m/z) $[M-H]^-$ calcd. for $C_{10}H_{10}FO_2$ 181.0665 found 181.0672.

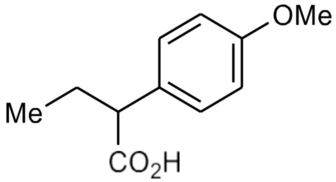

***2-(4-Methoxyphenyl)butanoic acid,* 3c.**[10] Starting from 1.350 mmol of corresponding β-substituted styrene the product was obtained as a white solid, yield 63% (0.164 g), m.p. = 57-60°C. **^{1}H NMR** (400 MHz, $CDCl_3$): δ = 0.84 (t, J = 7.4 Hz, 3H, Me), 1.72 (dt, J = 13.7, 7.4 Hz, 1H, CH_2), 2.02 (dt, J = 13.7, 7.4 Hz, 1H, CH_2), 3.35 (t, J = 7.7 Hz, 1H, CH), 3.73 (s, 3H, OMe), 6.79-6.83 (m, 2H, Ar), 7.16-7.20 (m, 2H, Ar), 11.29 (br s, 1H, CO_2H). **^{13}C NMR** (101 MHz, $CDCl_3$): δ = 12.2, 26.4, 52.6, 55.4, 114.2, 129.3, 130.6, 159.1, 180.9. **HRMS-EI** (m/z) $[M-H]^-$ calcd. for $C_{11}H_{13}O_3$ 193.0865 found 193.0872.

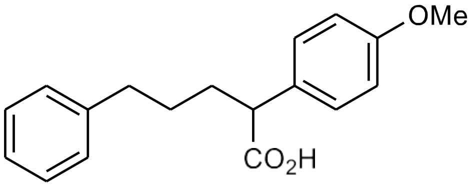

***2-(4-Methoxyphenyl)-5-phenylpentanoic acid,* 3d.** Starting from 0.839 mmol of corresponding β-substituted styrene the product was obtained as a colourless viscous oil, yield 52% (0.123 g). **^{1}H NMR** (400 MHz, $CDCl_3$): δ = 1.49-1.62 (m, 2H, CH_2), 1.73-1.81 (m, 1H, CH_2), 1.99-2.08 (m, 1H, CH_2), 2.51-2.63 (m, 2H, CH_2), 3.46 (t, J = 7.7 Hz, 1H, CH), 3.73 (s, 3H, OMe), 6.79-6.82 (m, 2H, Ar), 7.07.7.27 (m, 7H, Ar), 11.01 (br s, 1H, CO_2H). **^{13}C NMR** (101 MHz, $CDCl_3$): δ = 29.3, 32.7, 35.7,

[9] J. A. Mukhlall, B. C. Noll and W. H. Hersh, *J. Sulfur Chem.* 2011, **32**, 199-212.
[10] A. H. Mermerian and G. C. Fu, *J. Am. Chem. Soc.* 2005, **127**, 5604-5607.

50.7, 55.4, 114.2, 125.9, 128.4, 128.5, 128.8, 129.2, 130.5, 142.0, 159.1, 180.6. **HRMS-EI** (m/z) $[M-H]^-$ calcd. for $C_{18}H_{19}O_3$ 283.1334 found 283.1335.

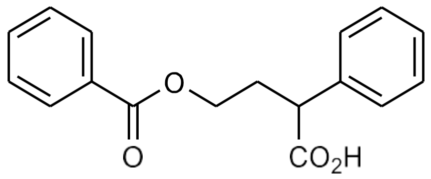

***4-(Benzoyloxy)-2-phenylbutanoic acid,* 3e.** Starting from 0.839 mmol of corresponding β-substituted styrene the product was obtained as a colourless viscous oil, yield 51% (0.122 g). **^{1}H NMR** (400 MHz, $CDCl_3$): δ = 2.44 (dddd, *J* = 12.8, 10.3, 9.2, 8.2 Hz, 1H, CH_2), 2.72 (dddd, *J* = 12.6, 9.0, 6.7, 3.4 Hz, 1H, CH_2), 3.84 (dd, *J* = 10.2, 9.0 Hz, 1H, CH), 4.36 (td, *J* = 9.2, 6.7 Hz, 1H, CH_2), 4.49 (td, *J* = 8.7, 3.4 Hz, 1H, CH_2), 7.28-7.33 (m, 2H, Ar), 7.36-7.39 (m, 2H, Ar), 7.49 (t, *J* = 7.7 Hz, 3H, Ar), 7.60-7.64 (m, 1H, Ar), 8.12-8.15 (m, 2H, Ar), 11.40 (br s, 1H, CO_2H). **^{13}C NMR** (101 MHz, $CDCl_3$): δ = 31.7, 45.7, 66.9, 127.8, 128.0, 128.6, 129.1, 129.4, 130.3, 134.0, 136.7, 172.4, 178.2. **HRMS-EI** (m/z) $[M-H]^-$ calcd. for $C_{17}H_{15}O_4$ 283.0970 found 283.0976.

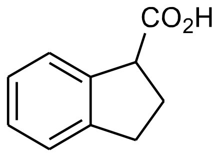

***2,3-Dihydro-1H-indene-1-carboxylic acid,* 3f.**[11] Starting from 1.722 mmol of corresponding β-substituted styrene the product was obtained as a yellowish viscous oil, yield 46% (0.142 g). **^{1}H NMR** (400 MHz, $CDCl_3$): δ = 2.31-2.49 (m, 2H, CH_2), 2.89-2.96 (m, 1H, CH_2), 3.07-3.15 (m, 1H, CH_2), 4.08 (dd, *J* = 8.5, 6.0 Hz, 1H, CH), 7.17-7.26 (m, 3H, Ar), 7.41-7.43 (m, 1H, Ar), 10.97 (br s, 1H, CO_2H). **^{13}C NMR** (101 MHz, $CDCl_3$): δ = 28.8, 31.9, 50.1, 124.9, 125.1, 126.7, 127.9, 140.1, 144.3, 180.6. **HRMS-EI** (m/z) $[M-H]^-$ calcd. for $C_{10}H_9O_2$ 161.0603 found 161.0610.

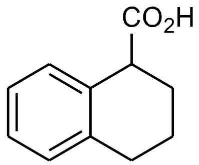

***1,2,3,4-Tetrahydronaphthalene-1-carboxylic acid,* 3g.**[12] Starting from 1.540 mmol of corresponding β-substituted styrene the product was obtained as a white solid, yield 92% (0.248 g), m.p. = 76-79°C. **^{1}H NMR** (400 MHz, $CDCl_3$): δ = 1.80-1.91 (m, 1H, CH_2), 1.99-2.13 (m, 2H, CH_2), 2.21-2.30 (m, 1H, CH_2), 2.78-2.94 (m, 2H, CH_2), 3.91 (t, *J* = 5.7 Hz, 1H, CH), 7.16-7.31 (m, 4H, Ar), 11.82 (br s, 1H, CO_2H).
^{13}C NMR (101 MHz, $CDCl_3$): δ = 20.6, 26.6, 29.2, 44.6, 126.0, 127.2, 129.6, 129.8, 132.6, 137.4, 181.9. **HRMS-EI** (m/z) $[M-H]^-$ calcd. for $C_{11}H_{11}O_2$ 175.0759 found 175.0763.

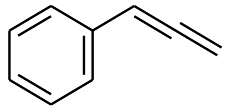

***Propa-1,2-dien-1-ylbenzene,* 4d.**[1,13] Starting from 28.800 mmol of corresponding styrene the product was obtained as a colorless liquid, yield 27% (0.917 g). **^{1}H NMR** (400 MHz, $CDCl_3$): δ = 5.16 (d, *J* = 6.8 Hz, 2H, allene), 6.18 (t, *J* = 6.8 Hz, 1H, allene), 7.19-7.24 (m, 1H, Ar), 7.30-7.34 (m, 4H, Ar).
^{13}C NMR (101 MHz, $CDCl_3$): δ = 78.9, 94.1, 126.9, 127.1, 128.8, 134.1, 210.0.

[11] J. Pietruszka, R. C. Simon, F. Kruska and M. Braun, Eur. J. Org. Chem. 2009, 6217-6224.
[12] L.-L. Liao, G.-M. Cao, J.-H. Ye, G.-Q. Sun, W.-J. Zhou, Y.-Y. Gui, S.-S. Yan, G. Shen, and D.-G. Yu, J. Am. Chem. Soc. 2018, **140**, 17338-17342.

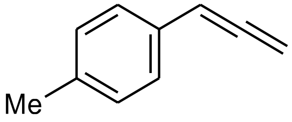

***1-Methyl-4-(propa-1,2-dien-1-yl)benzene,* 4e.**[13] Starting from 25.390 mmol of corresponding styrene the product was obtained as a colorless liquid, yield 42% (1.401 g). **^{1}H NMR** (400 MHz, $CDCl_3$): δ = 2.34 (s, 3H, Me), 5.14 (d, *J* = 6.8 Hz, 2H, allene), 6.15 (t, *J* = 6.8 Hz, 1H, allene), 7.13 (d, *J* = 7.9 Hz, 2H, Ar), 7.20 (d, *J* = 8.1 Hz, 2H, Ar). **^{13}C NMR** (101 MHz, $CDCl_3$): δ = 21.4, 78.8, 93.9, 126.3, 126.8, 129.4, 129.5, 131.1, 136.8, 209.8.

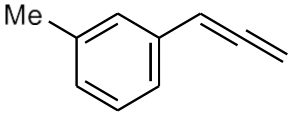

***1-Methyl-3-(propa-1,2-dien-1-yl)benzene,* 4f.**[13] Starting from 16.920 mmol of corresponding styrene the product was obtained as a colorless liquid, yield 42% (0.921 g). **^{1}H NMR** (400 MHz, $CDCl_3$): δ = 2.34 (s, 3H, Me), 5.14 (d, *J* = 6.8 Hz, 2H, allene), 6.14 (t, *J* = 6.8 Hz, 1H, allene), 7.02 (d, *J* = 7.6 Hz, 1H, Ar), 7.10-7.13 (m, 2H, Ar), 7.19-7.24 (m, 1H, Ar).
^{13}C NMR (101 MHz, $CDCl_3$): δ = 21.5, 78.8, 94.1, 124.1, 127.5, 127.9, 128.7, 133.9, 138.4, 210.0.

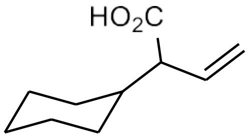

***2-Cyclohexylbut-3-enoic acid,* 5a.**[14] Starting from 1.637 mmol of corresponding allene the product was obtained as a white solid, yield 76% (0.208 g), m.p. = 67-69°C. **^{1}H NMR** (400 MHz, $CDCl_3$): δ = 0.84-0.93 (m, 1H, Cy), 1.00-1.31 (m, 4H, Cy), 1.61-1.78 (m, 6H, Cy), 2.76 (dd, *J* = 9.6, 8.1 Hz, 1H, CH), 5.10-5.18 (m, 2H, olefin), 5.78 (dt, *J* = 17.0, 9.9 Hz, 1H, olefin), 11.66 (br s, 1H, CO_2H).
^{13}C NMR (101 MHz, $CDCl_3$): δ = 26.1, 26.2, 26.3, 30.1, 31.3, 39.9, 57.4, 118.7, 134.6, 180.8.
HRMS-EI (m/z) $[M-H]^-$ calcd. for $C_{10}H_{15}O_2$ 167.1072 found 167.1081.

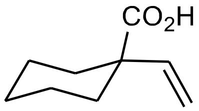

***1-Vinylcyclohexane-1-carboxylic acid,* 5b.**[15] Starting from 1.850 mmol of corresponding allene the product was obtained as a colourless viscous oil, yield 63% (0.179 g). **^{1}H NMR** (400 MHz, $CDCl_3$): δ = 1.26-1.33 (m, 1H, Cy), 1.40-1.64 (m, 6H, Cy), 1.75-1.80 (m, 1H, Cy), 2.08-2.13 (m, 2H, Cy), 5.16-5.21 (m, 2H, olefin), 5.83 (dd, *J* = 17.5, 10.7 Hz, 1H, olefin), 11.18 (br s, 1H, CO_2H).
^{13}C NMR (101 MHz, $CDCl_3$): δ = 23.2, 25.8, 33.4, 49.6, 115.3, 141.8.
HRMS-EI (m/z) $[M-H]^-$ calcd. for $C_9H_{13}O_2$ 153.0916 found 153.0927.

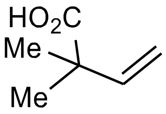

***2,2-Dimethylbut-3-enoic acid,* 5c.**[16] Starting from 2.936 mmol of corresponding allene the product was obtained as a colourless liquid, yield 65% (0.217 g). **^{1}H NMR** (400 MHz, $CDCl_3$): δ = 1.33 (s, 6H, 2xMe), 5.10-5.17 (m, 2H, olefin), 6.05

[13] J. Liu, Z. Han, X. Wang, Z. Wang and K. Ding, *J. Am. Chem. Soc.* 2015, **137**, 15346-15349.
[14] E. M. Brun, S. Gil, R. Mestres and M. Parra, *Synthesis* 2000, **8**, 1160-1165.
[15] H. A. Duong, P. B. Huleatt, Q.-W. Tan and E. L. Shuying, *Org. Lett.* 2013, **15**, 4034-4037.
[16] C. Zhu, J. Takaya and N. Iwasawa, *Org. Lett.* 2015, **17**, 1814-1817.

(dd, J = 17.4, 10.6 Hz, 1H, olefin), 10.93 (br s, 1H, CO_2H). **^{13}C NMR** (101 MHz, $CDCl_3$): δ = 24.6, 44.9, 113.6, 142.1, 183.0. **HRMS-EI** (m/z) $[M-H]^-$ calcd. for $C_6H_9O_2$ 113.0603 found 113.0612.

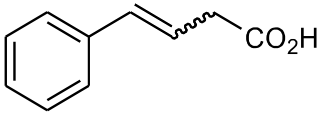
5da

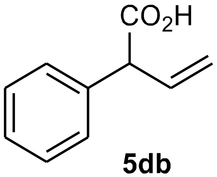
5db

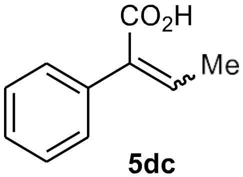
5dc

***4-Phenylbut-3-enoic acid* 5da, *2-phenylbut-3-enoic acid* 5db, *2-phenylbut-2-enoic acid* 5dc, NMR-ratio 10:1.5:4.5, LC-MS-ratio of 5 isomers 66:18:6:6:3.**[17] Starting from 1.722 mmol of corresponding allene the product was obtained as a yellowish viscous oil, yield 72% (0.202 g). **^{1}H NMR** (400 MHz, $CDCl_3$): δ = 1.80 (d, J = 7.2 Hz, 0.56H, **5dc** Me), 2.18 (d, J = 7.3 Hz, 1.02H, **5dc** Me), 3.32 (dd, J = 7.1, 1.5 Hz, 2H, **5da**, CH_2), 4.36-4.39 (m, 0.11H, **5db**, CH), 5.20-5.30 (m, 0.25H, **5db**, olefin), 5.82-5.87 (m, 0.14H, **5db**, olefin), 6.21-6.34 (m, 1.11H, **5da**/**5dc**, olefin), 6.47 (q, J = 7.3 Hz, 0.34H, **5dc**, olefin), 6.51-6.56 (m, 0.99H, **5da**, olefin), 7.18-7.43 (m, 11.34H, **5da**/**5db**/**5dc**, Ar), 11.72 (br s, 2.68H, **5da**/**5db**/**5dc**, CO_2H). **^{13}C NMR** (101 MHz, $CDCl_3$): δ = 15.9, 16.6, 30.5, 33.9, 38.2, 38.7, 55.7, 118.2, 120.9, 121.6, 122.4, 126.5, 126.9, 127.4, 127.7, 127.8, 128.1, 128.2, 128.3, 128.4, 128.5, 128.7, 128.8, 128.9, 129.6, 129.9, 134.2, 134.4, 135.1, 136.4, 136.8, 137.3, 138.3, 140.8, 143.5, 150.9, 173.8, 178.5. **HRMS-EI** (m/z) $[M-H]^-$ calcd. for $C_{10}H_9O_2$ 161.0603 found 161.0612.

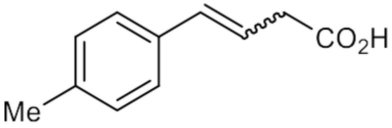
5ea

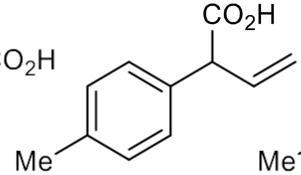
5eb

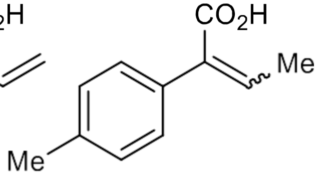
5ec

***4-(p-Tolyl)but-3-enoic acid* 5ea, *2-(p-tolyl)but-3-enoic acid* 5eb, *2-(p-tolyl)but-2-enoic acid* 5ec, NMR-ratio 10:1.8:4.3, LC-MS-ratio of 5 isomers 80:8:5:4:3.** Starting from 1.540 mmol of corresponding allene the product was obtained as a yellowish viscous oil, yield 68% (0.185 g). **^{1}H NMR** (400 MHz, $CDCl_3$): δ = 1.88 (d, J = 7.2 Hz, 0.27H, **5ec**, Me), 2.24 (d, J = 7.3 Hz, 0.56H, **5ec**, Me), 2.43-2.47 (m, 5.82H, **5ea**/**5eb**/**5ec**, Me), 3.37 (dd, J = 7.2, 1.5 Hz, 2H, **5ea**, CH_2), 4.41 (d, J = 8.0 Hz, 0.26H, **5eb**, CH), 5.26-5.33 (m, 0.40H, **5eb**, olefin), 5.88-5.96 (m, 0.18H, **5eb**, olefin), 6.27-6.36 (m, 1.25H, **5ea**/**5ec**, olefin), 6.51 (q, J = 7.3 Hz, 0.18H, **5ec**, olefin), 6.55-6.59 (m, 1H, **5ea**, olefin), 7.14-7.42 (m, 8.55H, **5ea**/**5eb**/**5ec**, Ar), 12.07 (br s, 2.33H, **5ea**/**5eb**/**5ec**, CO_2H). **^{13}C NMR** (101 MHz, $CDCl_3$): δ= 15.9, 16.5, 21.1, 21.2, 21.3, 21.4, 34.0, 35.0, 38.2, 38.3, 55.3, 118.0, 119.8, 121.4, 121.7, 126.4, 127.9, 128.1, 128.2, 128.7, 128.8, 129.0, 129.2, 129.3, 129.4, 129.5, 129.6, 129.8, 130.6, 132.6, 134.0, 134.2, 134.3, 134.5, 135.2, 135.5, 136.5, 137.0, 137.4, 137.5, 137.6, 138.3, 140.0, 143.1, 151.1, 172.4, 173.1, 174.0, 176.9, 178.7, 179.3. **HRMS-EI** (m/z) $[M-H]^-$ calcd. for $C_{11}H_{11}O_2$ 175.0759 found 175.0767.

[17] For **5da**, see: (a) K. Michigami, T. Mita and Y. Sato, *J. Am. Chem. Soc.* 2017, **139**, 6094-6097. For **5db**, see: (b) K.-J. Jiao, Z.-M. Li, X.-T. Xu, L.-P. Zhang, Y.-Q. Li, K. Zhang and T.-S. Mei, *Org. Chem. Front.* 2018, **5**, 2244-2248. For **5dc**, see: (c) F. N. Palmer, F. Lach, C. Poriel, A. G. Pepper, M. C. Bagley, A. M. Z. Slawin and C. J. Moody, *Org. Biomol. Chem.* 2005, **3**, 3805-3811. (d) V. D. Vitnik, M. D. Ivanović, Ž. J. Vitnik, J. B. Đorđević, Ž. S. Žižak, Z. D. Juranić and I. O. Juranić, *Synth. Commun.* 2009, **39:8**, 1457-1471. For similar mixture of regioisomers, see: (e) L.-L. Liao, G.-M. Cao, J.-H. Ye, G.-Q. Sun, W.-J. Zhou, Y.-Y. Gui, S.-S. Yan, G. Shen and D.-G. Yu, *J. Am. Chem. Soc.* 2018, **140**, 17338-17342.

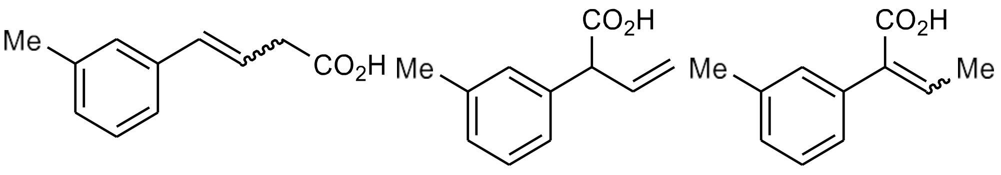

5fa **5fb** **5fc**

***4-(m-Tolyl)but-3-enoic acid* 5fa, *2-(m-tolyl)but-3-enoic acid* 5fb, *2-(m-tolyl)but-2-enoic acid* 5fc, NMR-ratio 10:1.1:4.1, LC-MS-ratio of 5 isomers 71:11:8:6:4.** Starting from 1.536 mmol of corresponding allene the product was obtained as a yellowish viscous oil, yield 53% (0.142 g). **^{1}H NMR** (400 MHz, $CDCl_3$): δ = 1.81 (d, *J* = 7.2 Hz, 0.42H, **5fc**, Me), 2.18 (d, *J* = 7.3 Hz, 0.81H, **5fc**, Me), 2.37-2.40 (m, 5.99H, **5fa**/**5fb**/**5fc**, Me), 3.32 (dd, *J* = 7.1, 1.4 Hz, 2H, **5fa**, CH_2), 4.34 (d, *J* = 8.1 Hz, 0.08H, **5fb**, CH), 5.20-5.30 (m, 0.18H, **5fb**, olefin), 5.83-5.88 (m, 0.11H, **5fb**, olefin), 6.26-6.33 (m, 1.13H, **5fa**/**5fc**, olefin), 6.46 (q, *J* = 7.3 Hz, 0.28H, **5fc**, olefin), 6.50-6.54 (m, 1H, **5fa**, olefin), 6.99-7.36 (m, 8.82H, **5fa**/**5fb**/**5fc**, Ar), 11.53 (br s, 2.41H, **5fa**/**5fb**/**5fc**, CO_2H). **^{13}C NMR** (101 MHz, $CDCl_3$): δ = 15.9, 16.5, 21.5, 21.6, 30.5, 34.0, 38.2, 38.6, 55.7, 118.1, 120.6, 121.5, 123.7, 125.1, 125.2, 125.8, 125.9, 126.9, 127.1, 127.7, 128.2, 128.5, 128.6, 128.7, 128.8, 128.9, 129.7, 130.5, 134.3, 134.6, 135.2, 136.7, 137.9, 138.2, 138.6, 140.3, 143.3, 151.0, 173.8, 178.5. **HRMS-EI** (m/z) $[M-H]^-$ calcd. for $C_{11}H_{11}O_2$ 175.0759 found 175.0769.

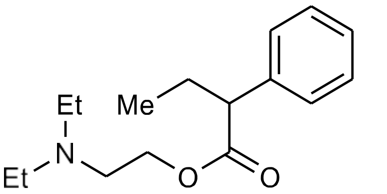

***2-(Diethylamino)ethyl 2-phenylbutanoate (Butetamate),* 6a.** Starting from 1.830 mmol of corresponding acid the product was obtained as a colourless liquid, yield 91% (0.436 g). **^{1}H NMR** (400 MHz, $CDCl_3$): δ = 0.93 (dt, *J* = 33.3, 7.3 Hz, 9H, 3xMe), 1.74-1.85 (m, 1H, CH_2), 2.04-2.15 (m, 1H, CH_2), 2.50 (q, *J* = 7.1 Hz, 4H, 2xCH_2), 2.65 (t, *J* = 6.2 Hz, 2H, CH_2), 3.45 (t, *J* = 7.7 Hz, 1H, CH), 4.09-4.19 (m, 2H, CH_2), 7.23-7.31 (m, 5H, Ar). **^{13}C NMR** (101 MHz, $CDCl_3$): δ = 12.1, 12.3, 26.9, 47.8, 51.2, 53.7, 63.2, 127.3, 128.2, 128.7, 139.3, 174.2. **HRMS-EI** (m/z) $[M+H]^+$ calcd. for $C_{16}H_{26}NO_2$ 264.1958 found 264.1958.

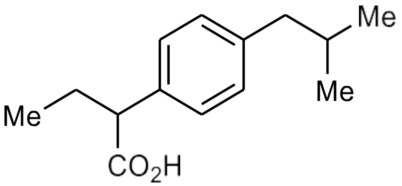

***2-(4-Isobutylphenyl)butanoic acid (Butibufen),* 6b.** Starting from 1.148 mmol of corresponding β-substituted styrene the product was obtained as a colourless viscous oil, yield 64% (0.162 g). **^{1}H NMR** (400 MHz, $CDCl_3$): δ = 0.89-0.93 (m, 9H, 3xMe), 1.76-1.88 (m, 2H, CH_2), 2.04-2.15 (m, 1H, CH), 2.45 (d, *J* = 7.1 Hz, 2H, CH_2), 3.42-3.44 (m, 1H, CH), 7.09-7.11 (m, 2H, Ar), 7.20-7.22 (m, 2H, Ar), 10.25 (s, 1H, CO_2H). **^{13}C NMR** (101 MHz, $CDCl_3$): δ = 12.3, 22.6, 26.5, 30.3, 30.5, 45.2, 53.1, 127.9, 129.5, 135.7, 141.1, 180.4. **HRMS-EI** (m/z) $[M-H]^-$ calcd. for $C_{14}H_{19}O_2$ 219.1385 found 219.1392.

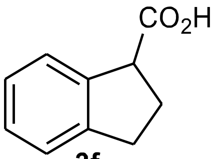

3f

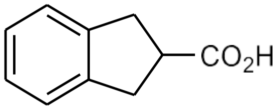

3f'

***2,3-Dihydro-1H-indene-1-carboxylic acid* 3f, *2,3-dihydro-1H-indene-2-carboxylic acid* 3f', LC-MS-ratio 4:1.** Starting from 1.720 mmol of corresponding β-substituted styrene the product was obtained as a yellowish viscous oil, yield 68% (0.190 g). ^{1}**H NMR** (400 MHz, $CDCl_3$): δ = 2.29-2.49 (m, 2H, CH_2 **3f/3f'**), 2.76 (t, *J* = 7.3 Hz, 0.29H, CH_2 from **3f'**), 2.89-2.96 (m, 1H, CH_2 **3f**), 3.05-3.15 (m, 1H, CH_2 **3f/3f'**), 3.19-3.55 (m, 1.39H, CH_2, CH **3f'**), 4.08 (dd, *J* = 8.5, 6.0 Hz, 1H, CH **3f**), 7.15-7.30 (m, 4.3H, Ar **3f/3f'**), 7.42-7.44 (m, 1H, Ar **3f/3f'**), 11.55 (br s, 1H, CO_2H **3f/3f'**). ^{13}**C NMR** (101 MHz, $CDCl_3$): δ = 28.8, 30.5, 31.0, 31.8, 34.3, 36.1, 43.5, 50.1, 59.0, 124.5, 124.8, 124.9, 125.1, 126.6, 126.7, 126.8, 126.9, 127.9, 129.2, 140.1, 141.4, 144.3, 144.6, 176.6, 180.8. **HRMS-EI** (m/z) $[M-H]^-$ calcd. for $C_{10}H_9O_2$ 161.0603 found 161.0609.

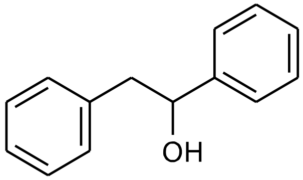

***1,2-Diphenylethan-1-ol,* 9a.**[18] Starting from 1.110 mmol of *trans*-stilbene the product was obtained as a colourless viscous oil, yield 96% (0.212 g). ^{1}**H NMR** (400 MHz, $CDCl_3$): δ = 2.18 (s, 1H, OH), 3.00-3.10 (m, 2H, CH_2), 4.90 (dd, *J* = 8.1, 5.3 Hz, 1H, CH), 7.21-7.42 (m, 10H, Ar). ^{13}**C NMR** (101 MHz, $CDCl_3$): δ = 46.2, 75.4, 126.0, 126.7, 127.7, 128.5, 128.6, 129.6, 138.2, 144.0.

[18] M. R. Hollerbach and T. J. Barker, *Organometallics* 2018, **37**, 1425-1427.

Copies of spectra

Compound 1e

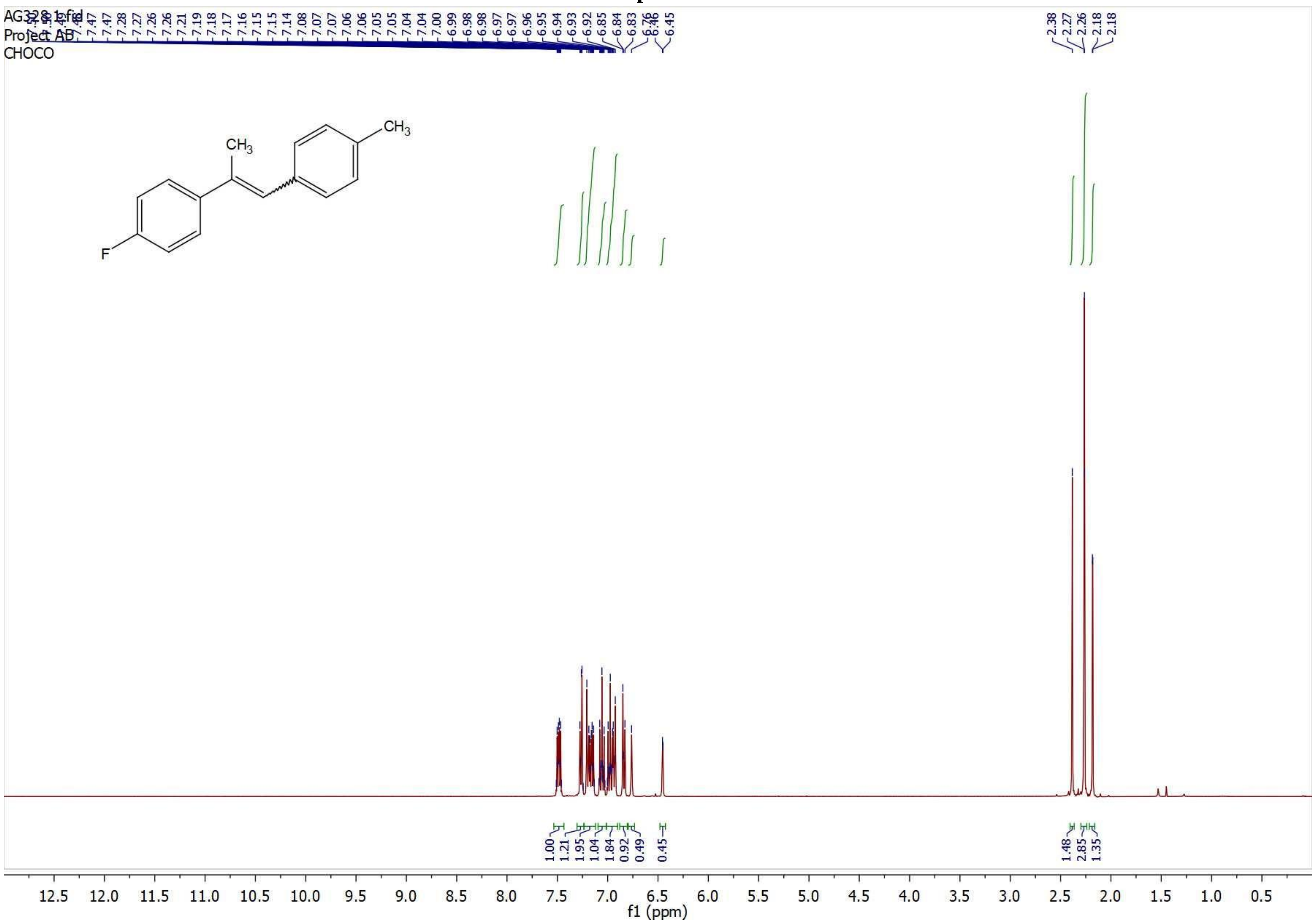

Compound 1e

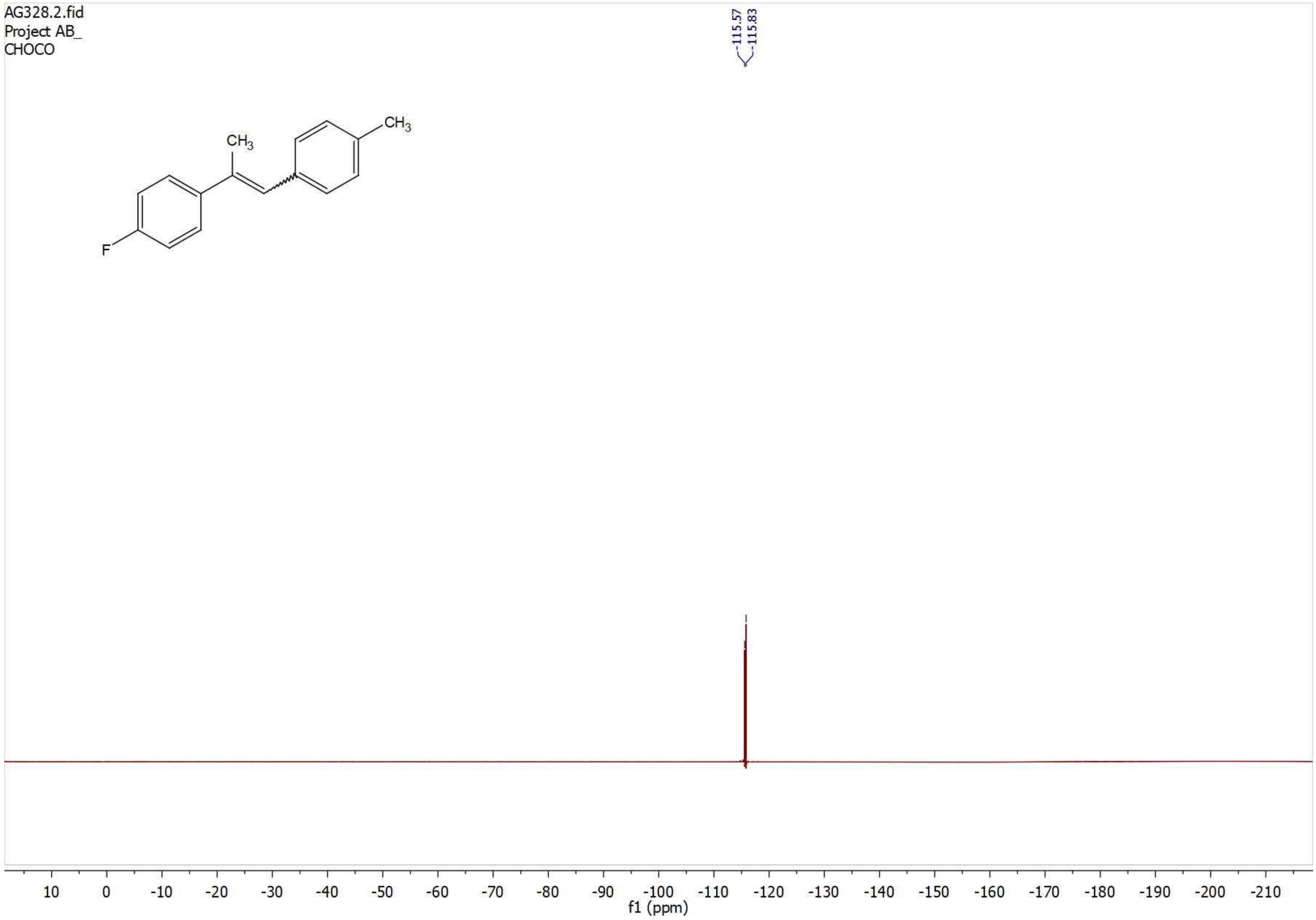

AG328.2.fid
Project AB_
CHOCO
-115.57
-115.83
CH3
CH3
F
10 0 -10 -20 -30 -40 -50 -60 -70 -80 -90 -100 -110 -120 -130 -140 -150 -160 -170 -180 -190 -200 -210
f1 (ppm)

Compound 1e

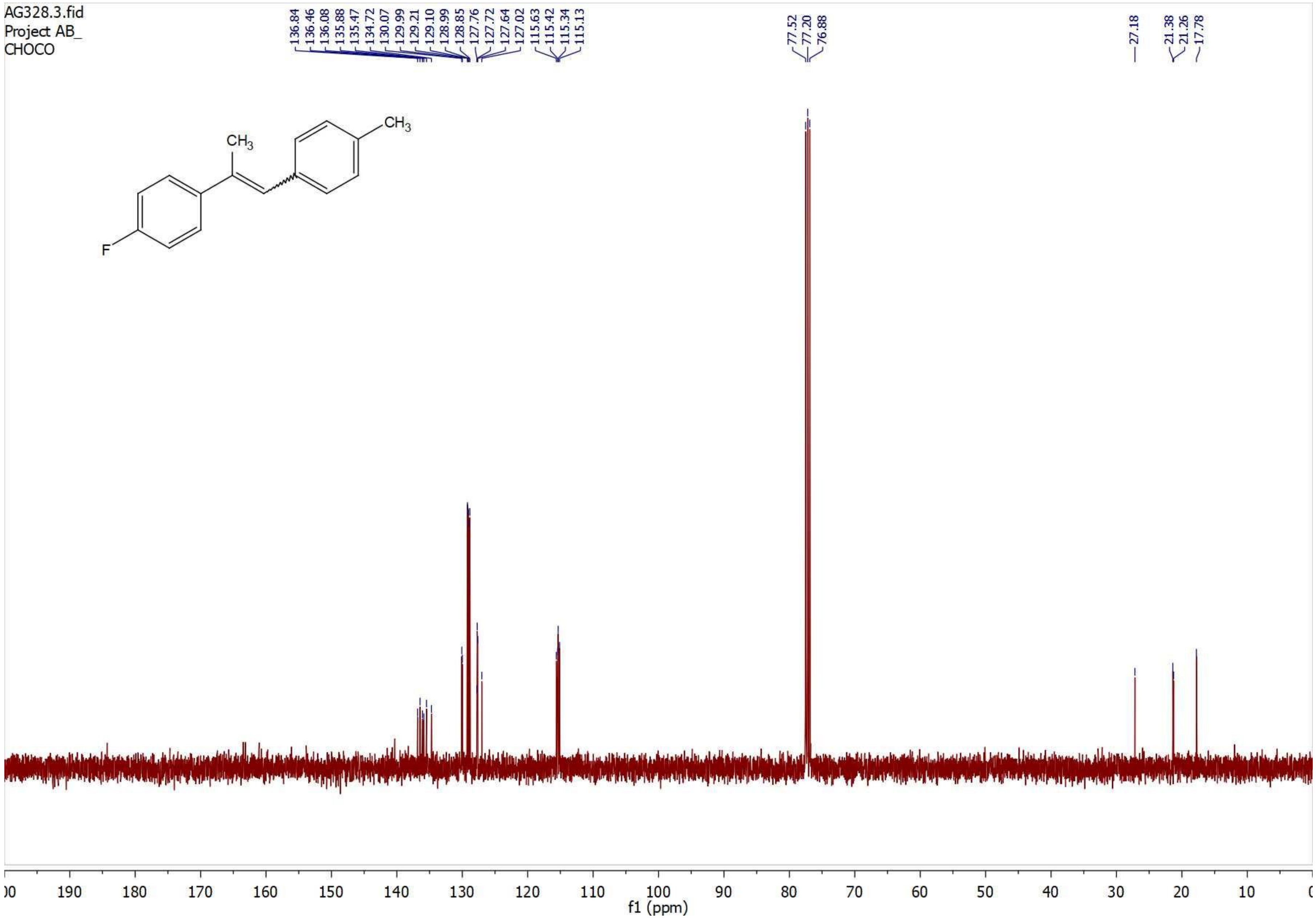

AG328.3.fid
Project AB_
CHOCO
136.84
136.46
136.08
135.88
135.47
134.72
130.07
129.99
129.21
129.10
128.99
128.85
127.76
127.72
127.64
127.02
115.63
115.42
115.34
115.13
77.52
77.20
76.88
27.18
21.38
21.26
17.78
CH3
CH3
F
190
180
170
160
150
140
130
120
110
100
90
80
70
60
50
40
30
20
10
f1 (ppm)

Compound 1g

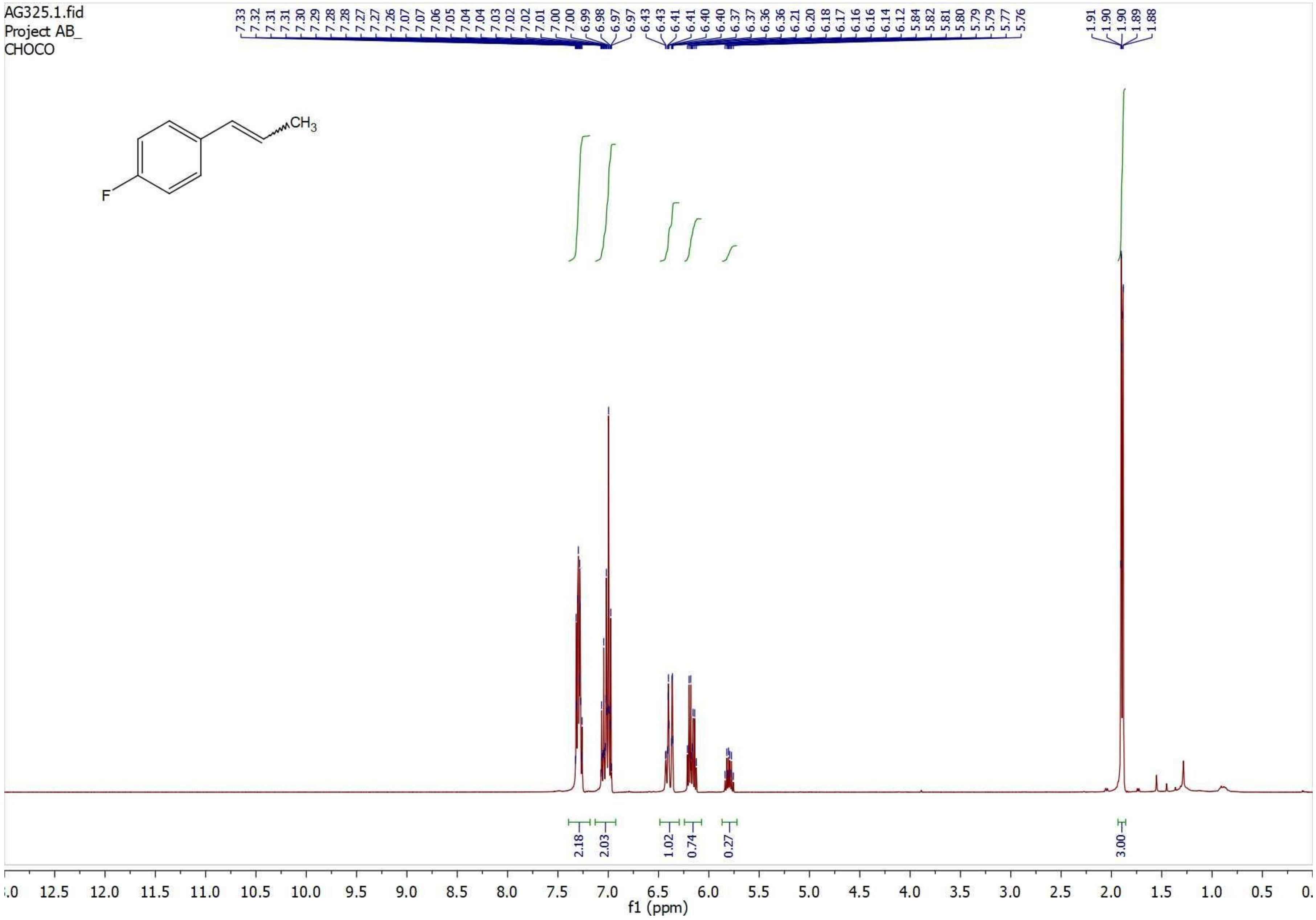
AG325.1.fid
Project AB_
CHOCO
F
CH3
f1 (ppm)
2.18
2.03
1.02
0.74
0.27
3.00

Compound 1g

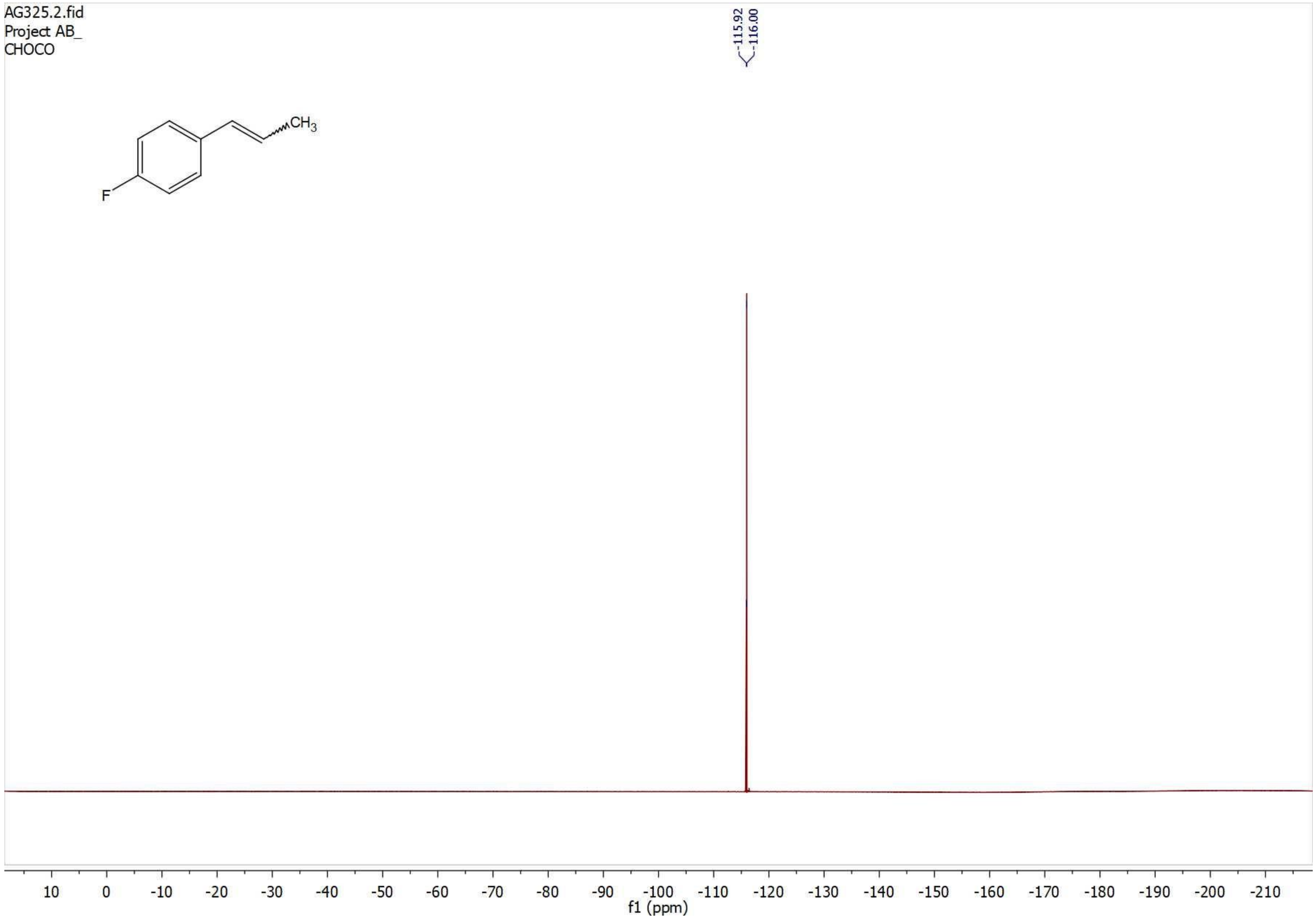
AG325.2.fid
Project AB_
CHOCO
-115.92
-116.00
CH3
F
10
0
-10
-20
-30
-40
-50
-60
-70
-80
-90
-100
-110
-120
-130
-140
-150
-160
-170
-180
-190
-200
-210
f1 (ppm)

Compound 1g

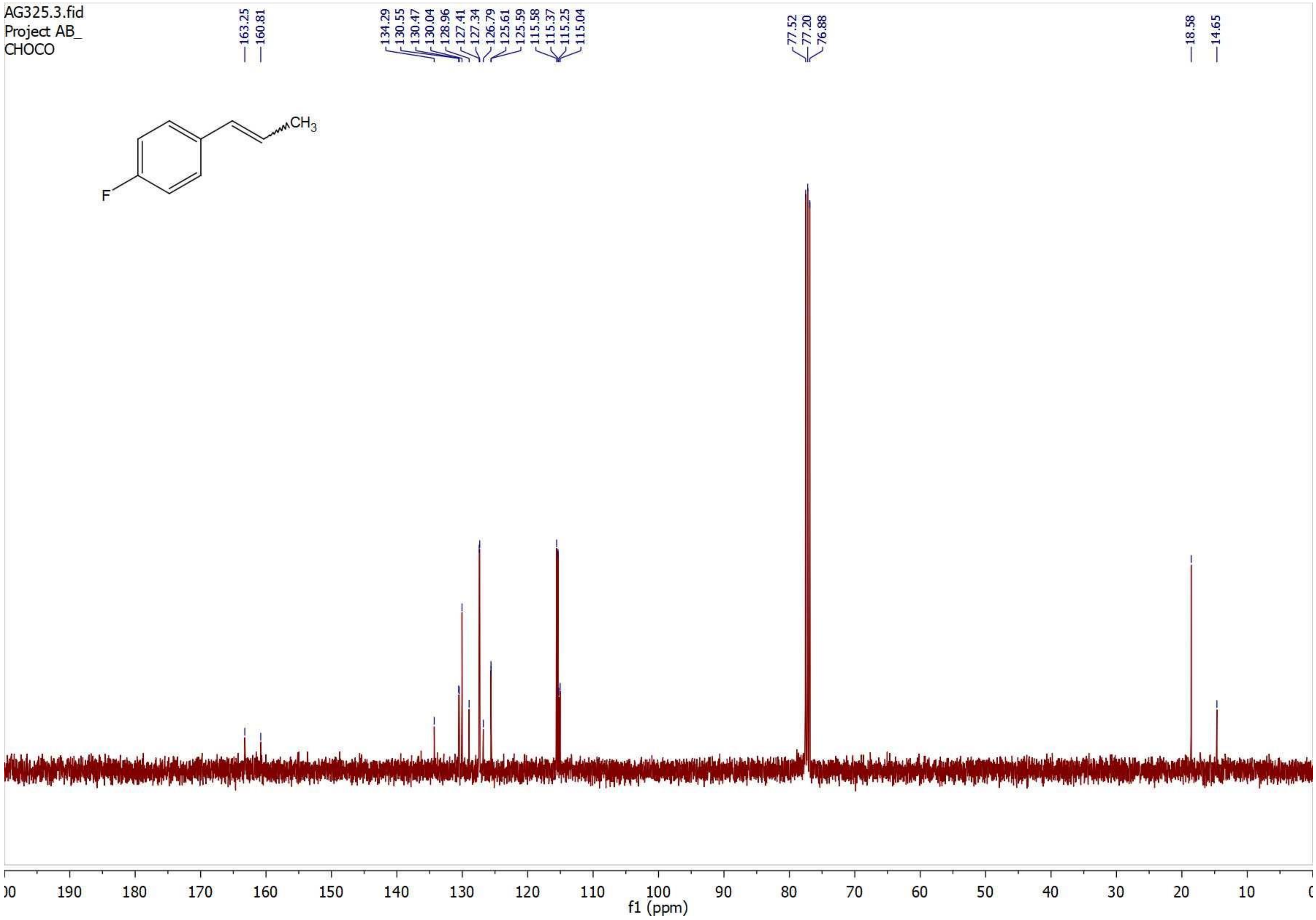
AG325.3.fid
Project AB_
CHOCO
163.25
160.81
134.29
130.55
130.47
130.04
128.96
127.41
127.34
126.79
125.61
125.59
115.58
115.37
115.25
115.04
77.52
77.20
76.88
18.58
14.65
F
CH3
f1 (ppm)

Compound 1h

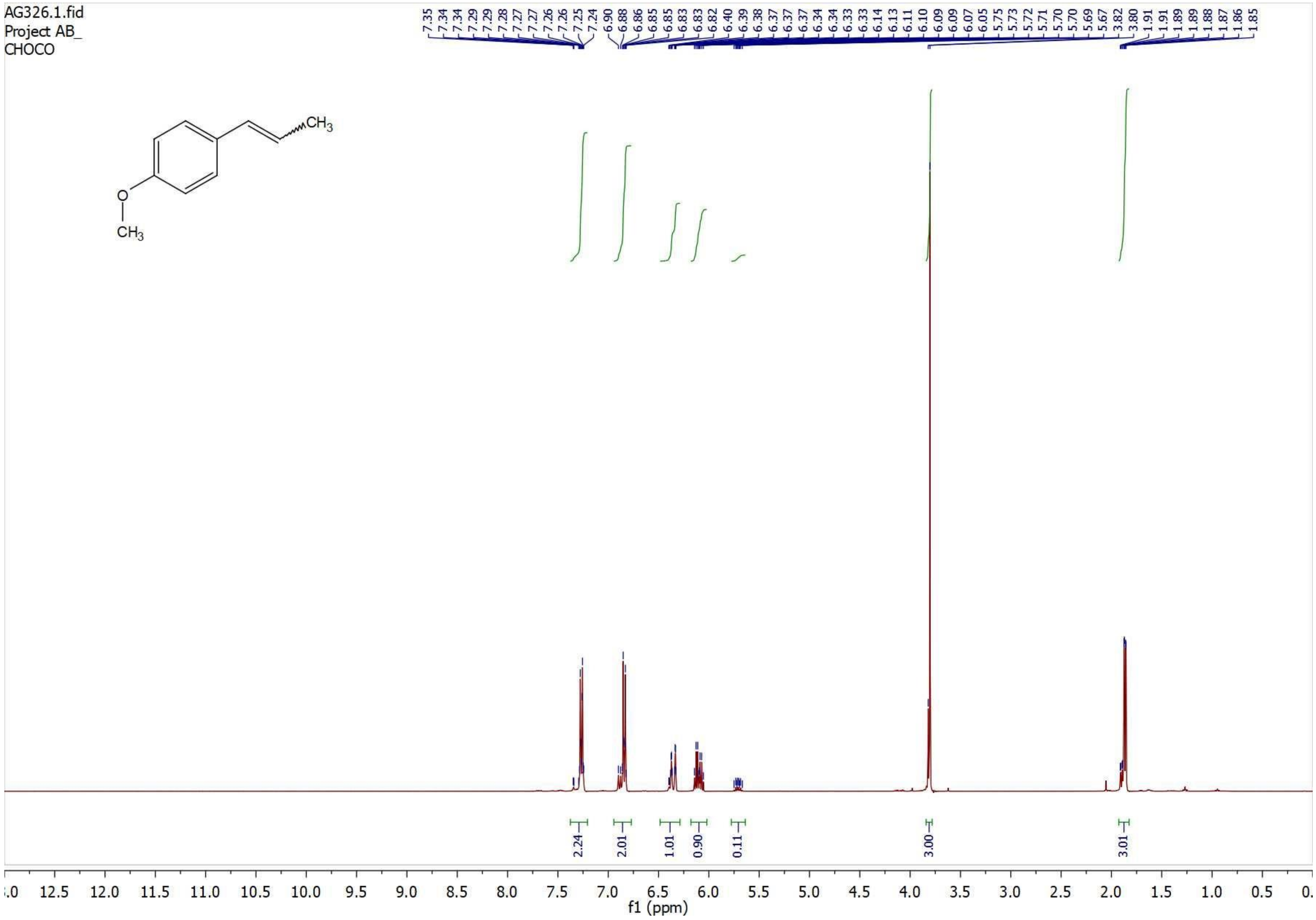

AG326.1.fid
Project AB_
CHOCO
CH3
O
CH3
f1 (ppm)
2.24
2.01
1.01
0.90
0.11
3.00
3.01

Compound 1h

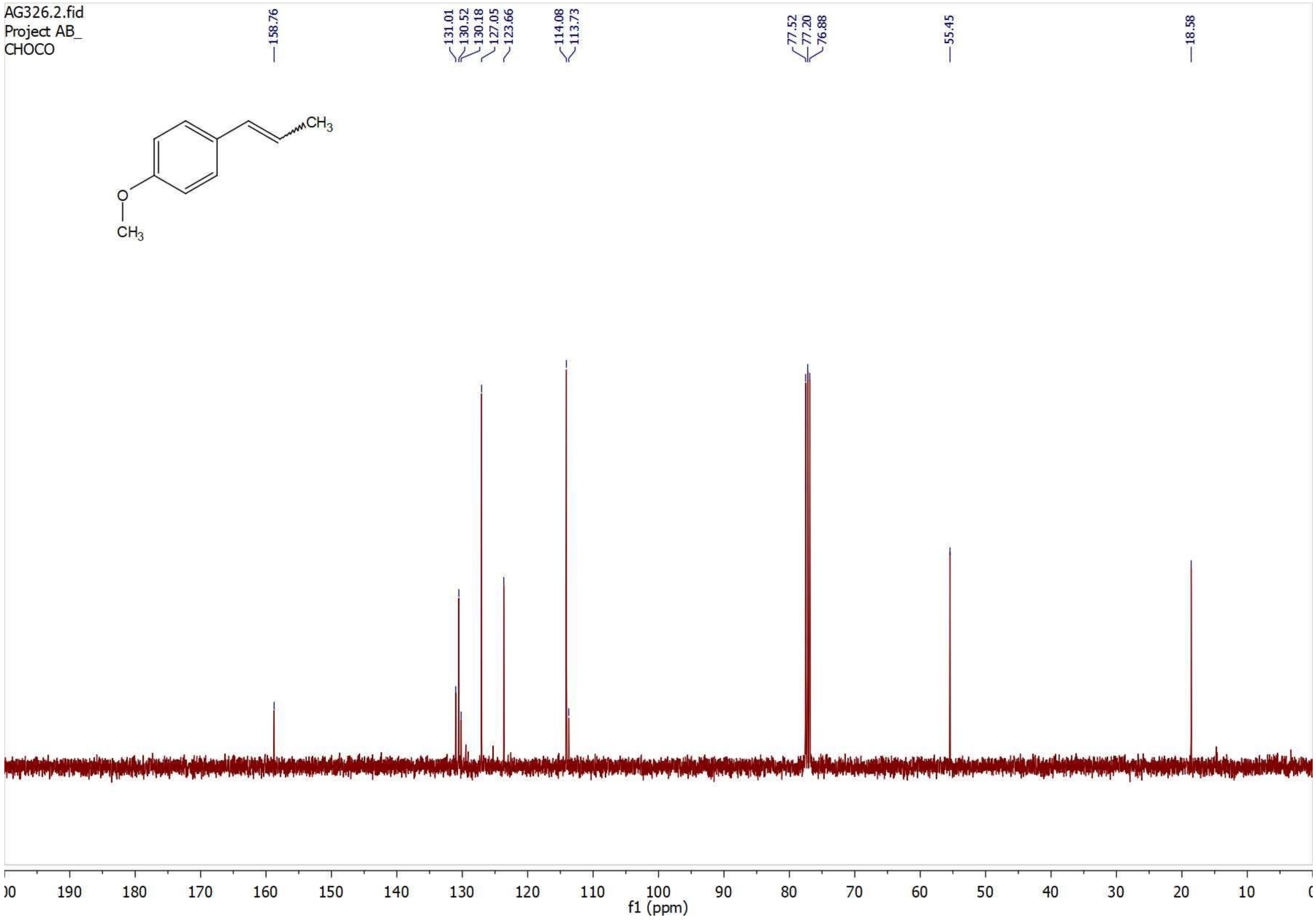
AG326.2.fid
Project AB_
CHOCO
158.76
131.01
130.52
130.18
127.05
123.66
114.08
113.73
77.52
77.20
76.88
55.45
18.58
O
CH3
CH3
190
180
170
160
150
140
130
120
110
100
90
80
70
60
50
40
30
20
10
f1 (ppm)

Compound 1i

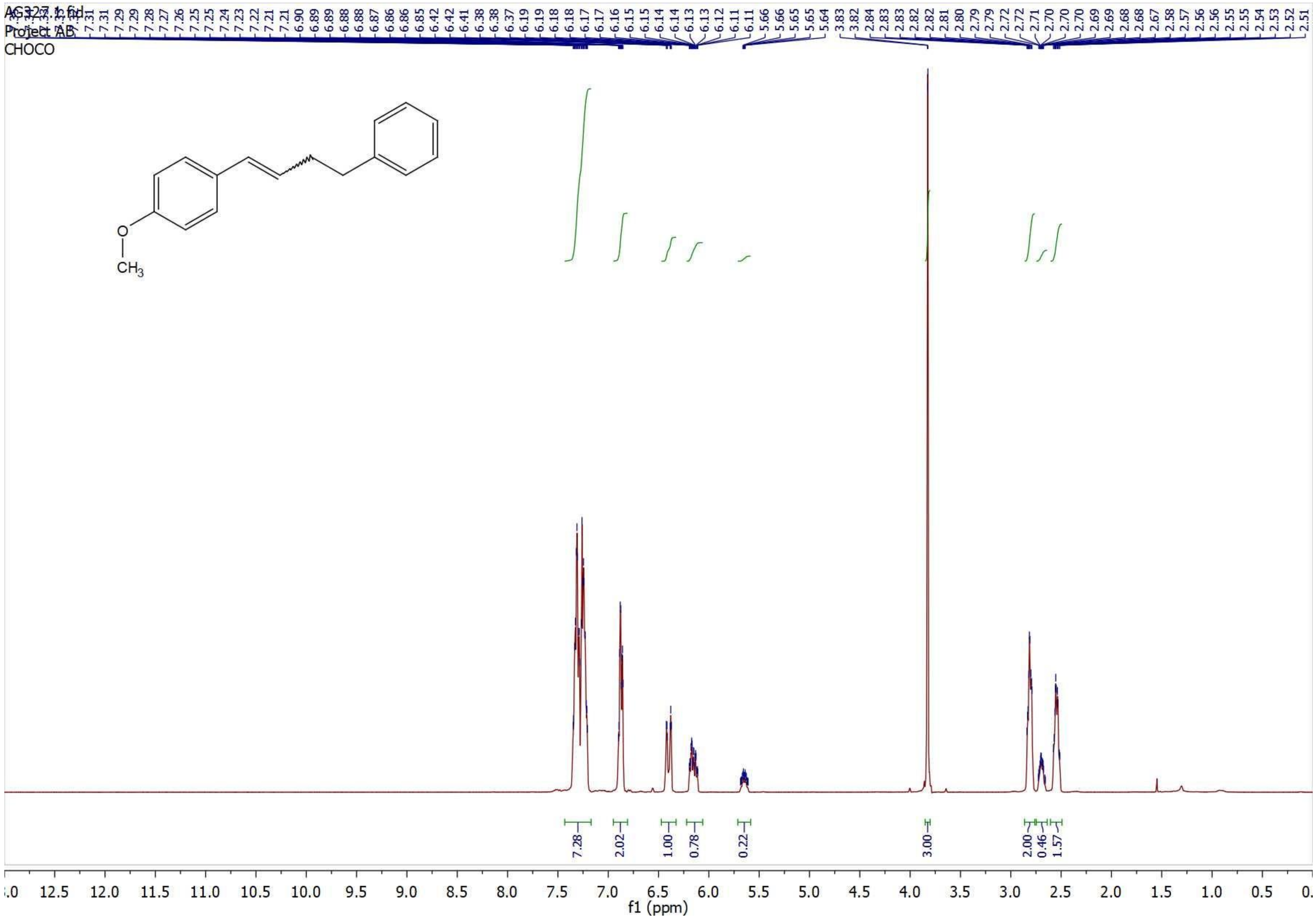
AG327.1.fid
Project AB
CHOCO
7.31 7.31 7.29 7.29 7.28 7.27 7.26 7.25 7.25 7.24 7.23 7.22 7.21 7.21 6.90 6.89 6.89 6.88 6.88 6.87 6.86 6.86 6.85 6.42 6.42 6.41 6.38 6.38 6.37 6.19 6.18 6.18 6.17 6.17 6.16 6.15 6.15 6.14 6.14 6.13 6.13 6.12 6.11 6.11 5.66 5.66 5.65 5.64 3.83 3.82 2.84 2.83 2.83 2.82 2.82 2.81 2.80 2.79 2.79 2.72 2.72 2.71 2.70 2.70 2.70 2.69 2.69 2.68 2.68 2.67 2.58 2.57 2.56 2.56 2.55 2.55 2.54 2.53 2.52 2.51
O
CH3
7.28
2.02
1.00
0.78
0.22
3.00
2.00
0.46
1.57
12.5 12.0 11.5 11.0 10.5 10.0 9.5 9.0 8.5 8.0 7.5 7.0 6.5 6.0 5.5 5.0 4.5 4.0 3.5 3.0 2.5 2.0 1.5 1.0 0.5
f1 (ppm)

Compound 1i

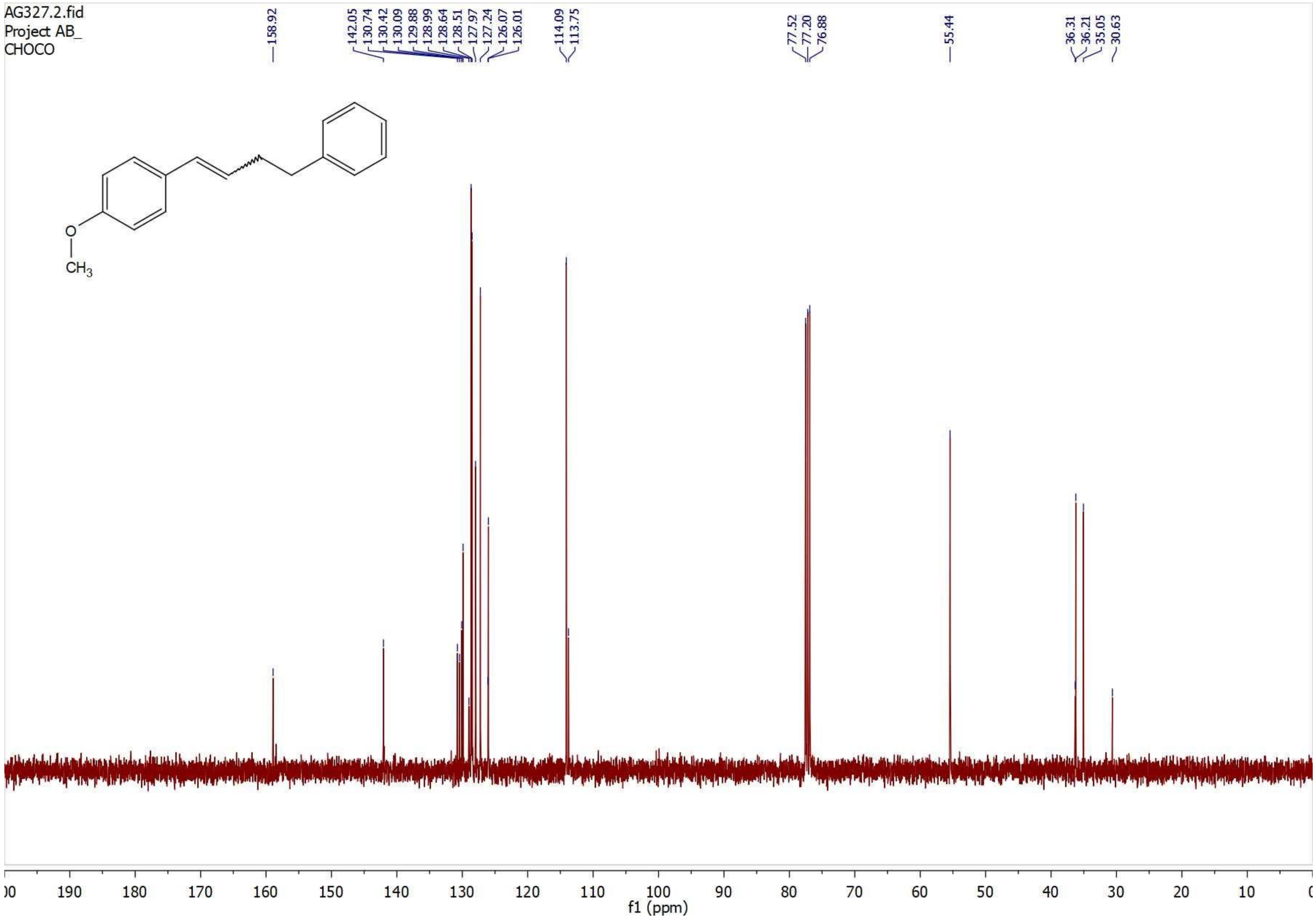

AG327.2.fid
Project AB_
CHOCO
158.92
142.05
130.74
130.42
130.09
129.88
128.99
128.64
128.51
127.97
127.24
126.07
126.01
114.09
113.75
77.52
77.20
76.88
55.44
36.31
36.21
35.05
30.63
O
CH3
190
180
170
160
150
140
130
120
110
100
90
80
70
60
50
40
30
20
10
f1 (ppm)

Compound 1j

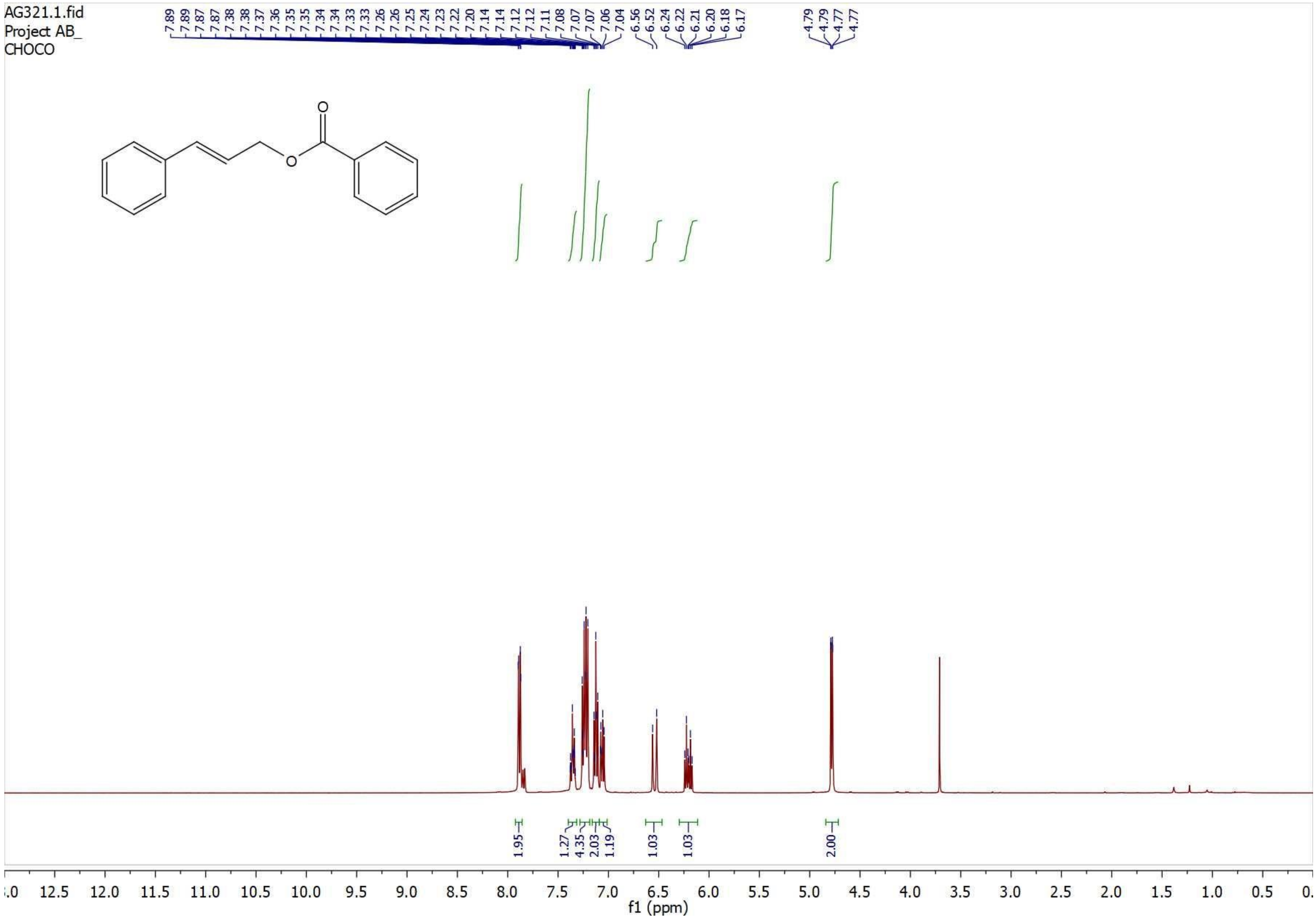
AG321.1.fid
Project AB_
CHOCO
7.89 7.89 7.87 7.87 7.38 7.38 7.37 7.36 7.35 7.35 7.34 7.34 7.33 7.33 7.26 7.26 7.25 7.24 7.23 7.22 7.20 7.14 7.14 7.12 7.12 7.11 7.08 7.07 7.07 7.06 7.04 6.56 6.52 6.24 6.22 6.21 6.20 6.18 6.17 4.79 4.79 4.77 4.77
O
O
1.95
1.27
4.35
2.03
1.19
1.03
1.03
2.00
13.0 12.5 12.0 11.5 11.0 10.5 10.0 9.5 9.0 8.5 8.0 7.5 7.0 6.5 6.0 5.5 5.0 4.5 4.0 3.5 3.0 2.5 2.0 1.5 1.0 0.5 0.
f1 (ppm)

Compound 1j

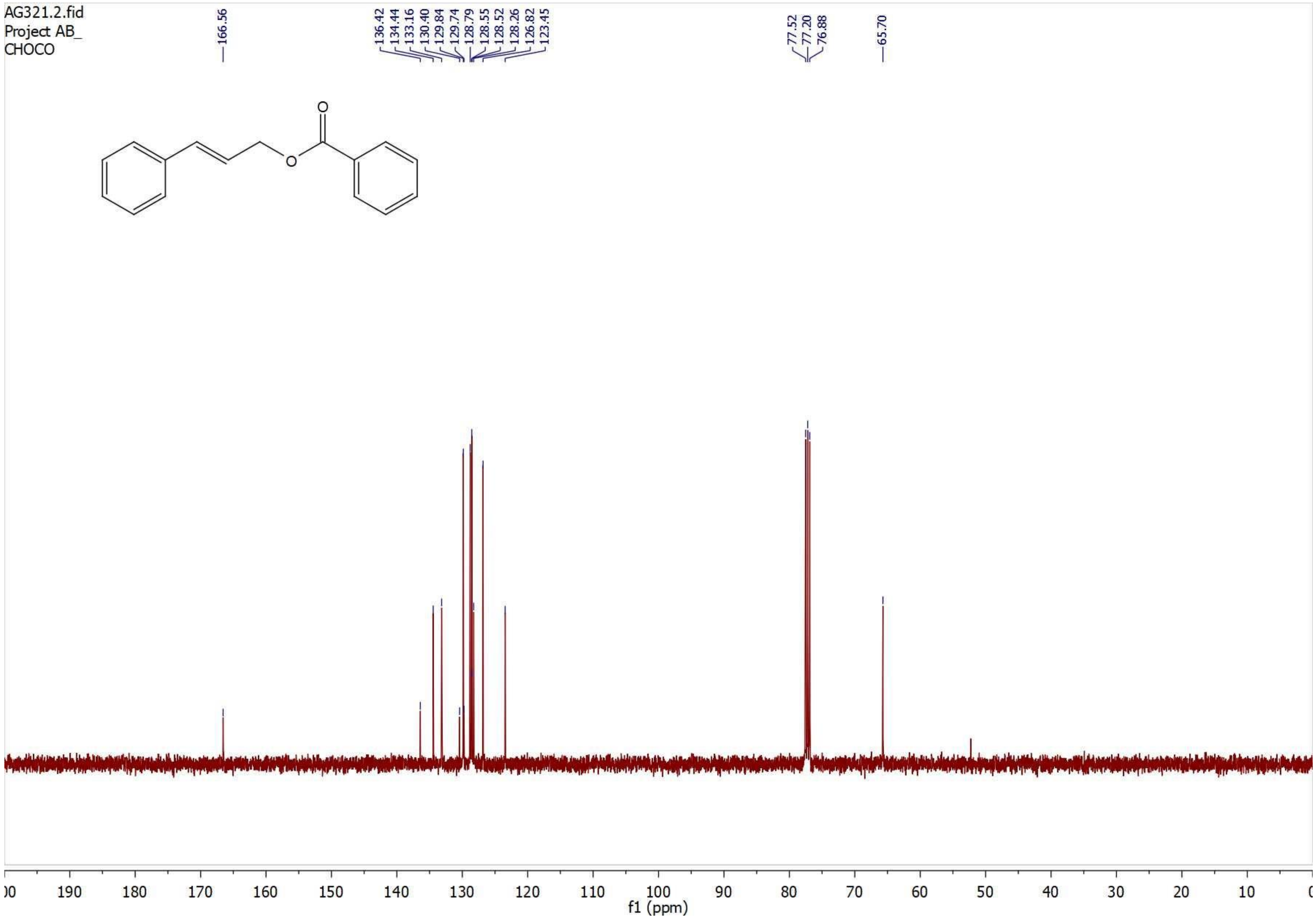
AG321.2.fid
Project AB_
CHOCO
166.56
136.42
134.44
133.16
130.40
129.84
129.74
128.79
128.55
128.52
128.26
126.82
123.45
77.52
77.20
76.88
65.70
O
O
190
180
170
160
150
140
130
120
110
100
90
80
70
60
50
40
30
20
10
f1 (ppm)

Compound 1m

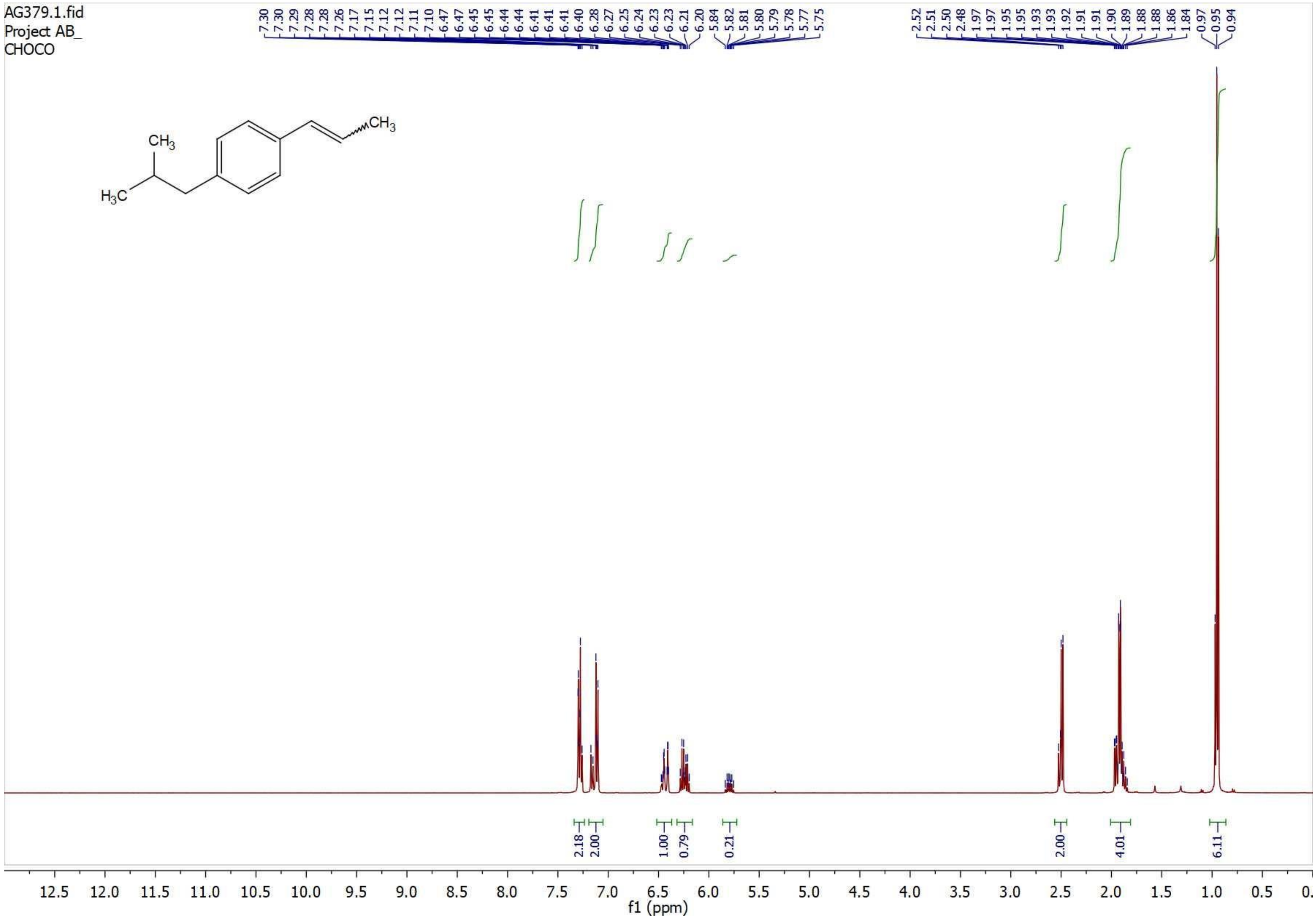

Compound 1m

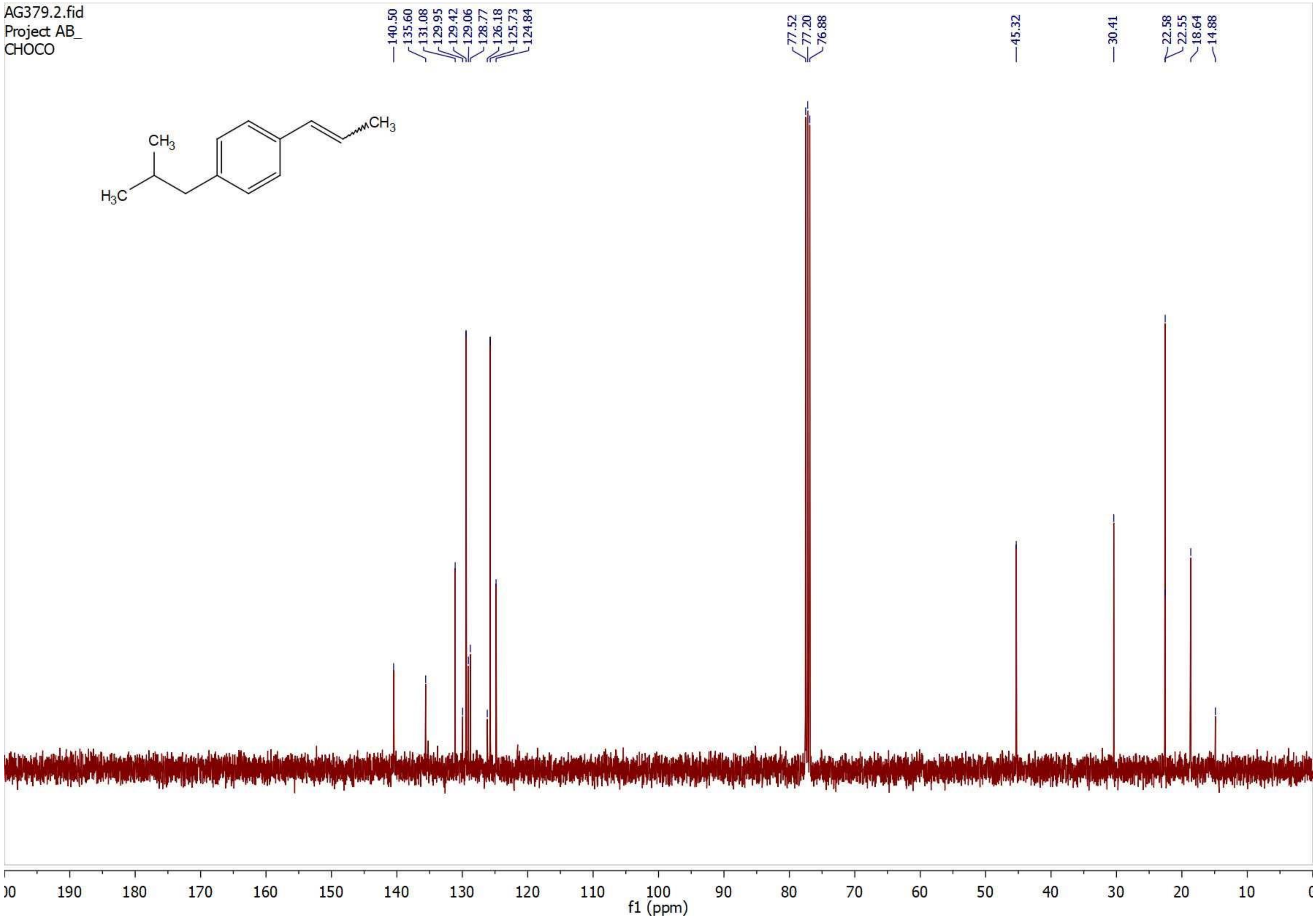
AG379.2.fid
Project AB_
CHOCO
140.50
135.60
131.08
129.95
129.42
129.06
128.77
126.18
125.73
124.84
77.52
77.20
76.88
45.32
30.41
22.58
22.55
18.64
14.88
CH3
H3C
CH3
f1 (ppm)

Compound 2a

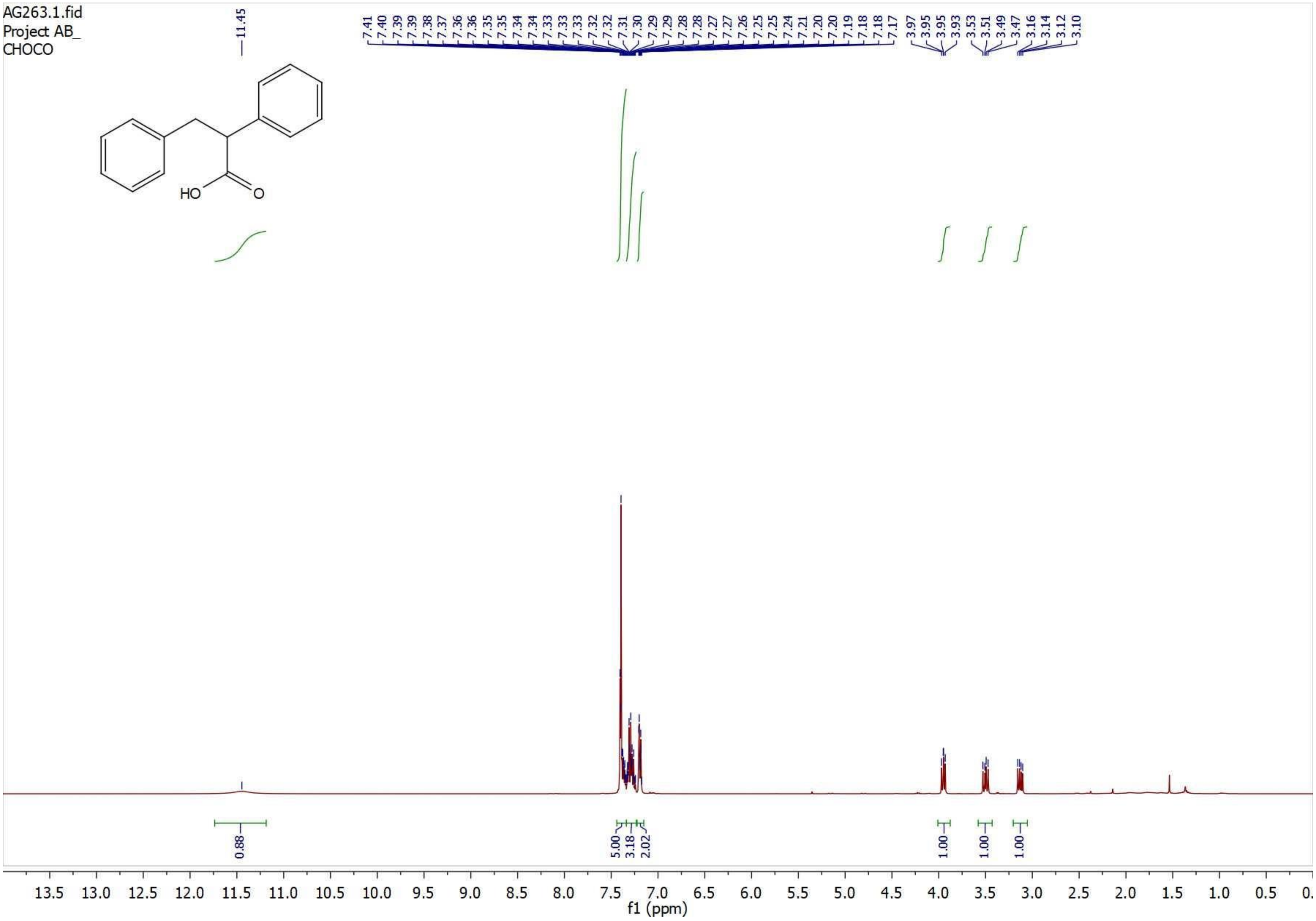
AG263.1.fid
Project AB_
CHOCO
11.45
7.41 7.40 7.39 7.39 7.38 7.37 7.36 7.36 7.35 7.35 7.34 7.34 7.33 7.33 7.33 7.32 7.32 7.31 7.30 7.29 7.29 7.28 7.28 7.27 7.27 7.26 7.25 7.25 7.24 7.21 7.20 7.20 7.19 7.18 7.18 7.17
3.97 3.95 3.95 3.93
3.53 3.51 3.49 3.47
3.16 3.14 3.12 3.10
HO
O
0.88
5.00
3.18
2.02
1.00
1.00
1.00
13.5 13.0 12.5 12.0 11.5 11.0 10.5 10.0 9.5 9.0 8.5 8.0 7.5 7.0 6.5 6.0 5.5 5.0 4.5 4.0 3.5 3.0 2.5 2.0 1.5 1.0 0.5 0.
f1 (ppm)

Compound 2a

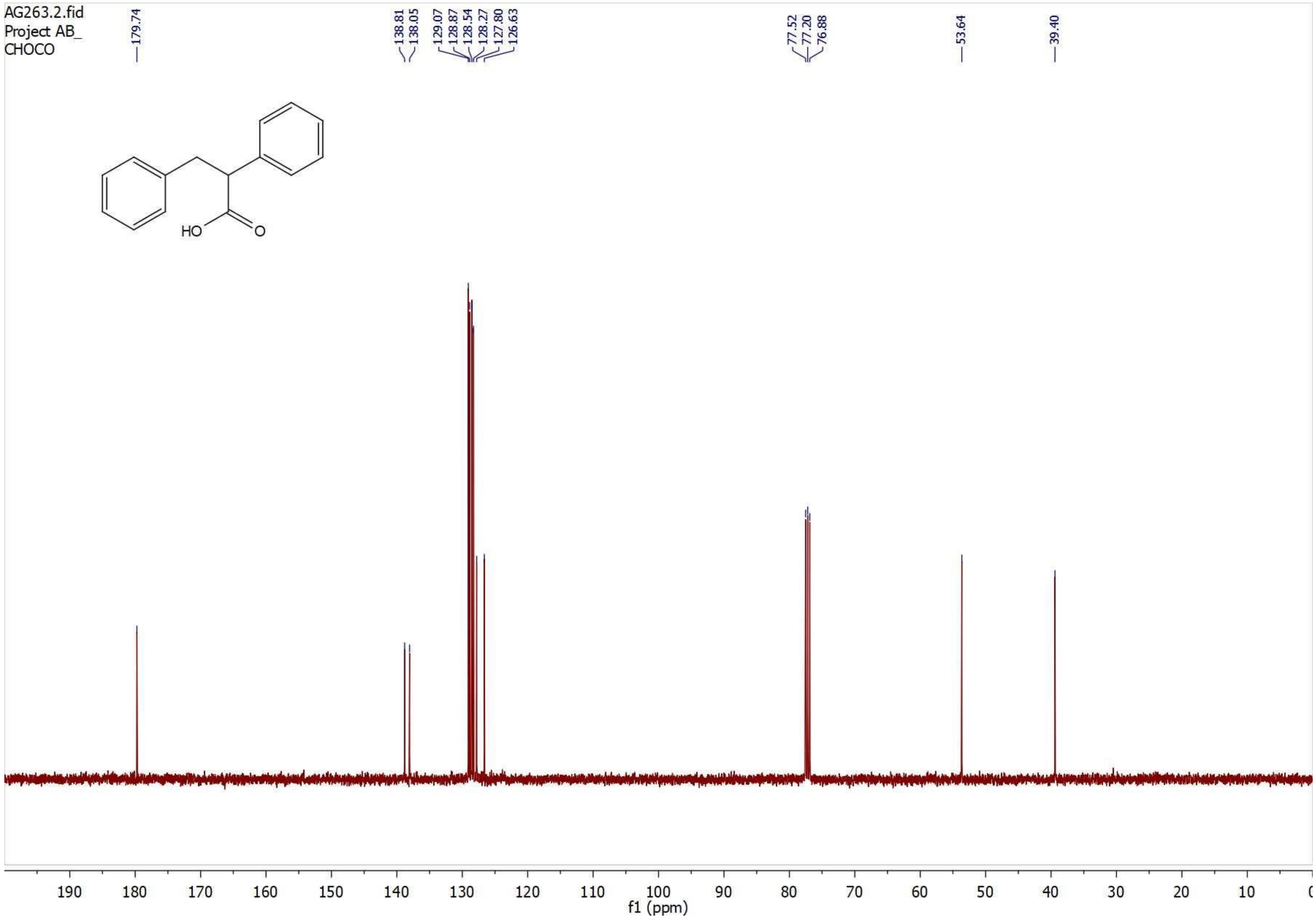

AG263.2.fid
Project AB_
CHOCO
179.74
138.81
138.05
129.07
128.87
128.54
128.27
127.80
126.63
77.52
77.20
76.88
53.64
39.40
HO
O
190
180
170
160
150
140
130
120
110
100
90
80
70
60
50
40
30
20
10
f1 (ppm)

Compound 2b

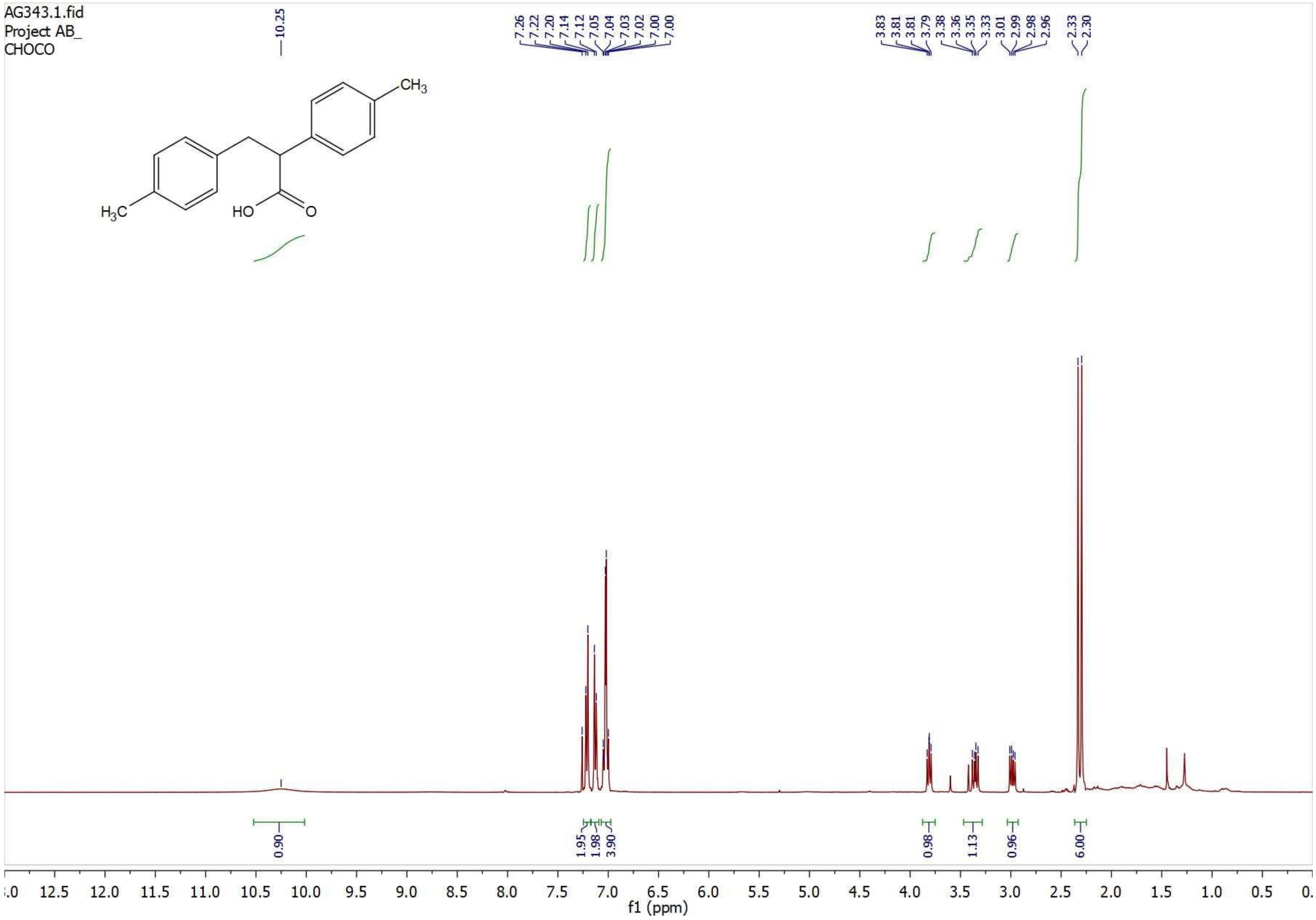
AG343.1.fid
Project AB_
CHOCO
10.25
7.26
7.22
7.20
7.14
7.12
7.05
7.04
7.03
7.02
7.00
7.00
3.83
3.81
3.81
3.79
3.38
3.36
3.35
3.33
3.01
2.99
2.98
2.96
2.33
2.30
CH3
H3C
HO
O
0.90
1.95
1.98
3.90
0.98
1.13
0.96
6.00
f1 (ppm)

Compound 2b

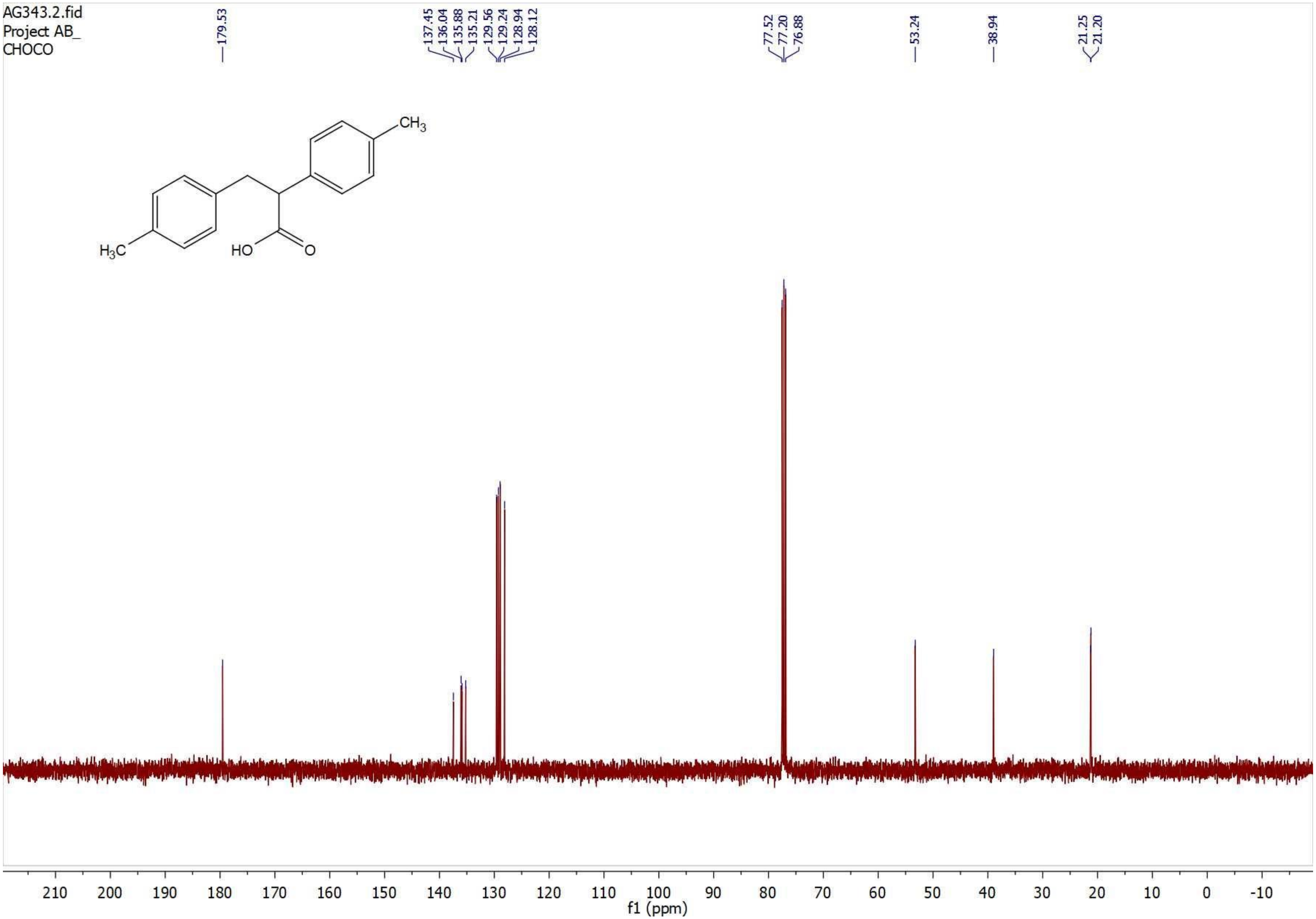
AG343.2.fid
Project AB_
CHOCO
179.53
137.45
136.04
135.88
135.21
129.56
129.24
128.94
128.12
77.52
77.20
76.88
53.24
38.94
21.25
21.20
CH3
H3C
HO
O
210 200 190 180 170 160 150 140 130 120 110 100 90 80 70 60 50 40 30 20 10 0 -10
f1 (ppm)

Compound 2c

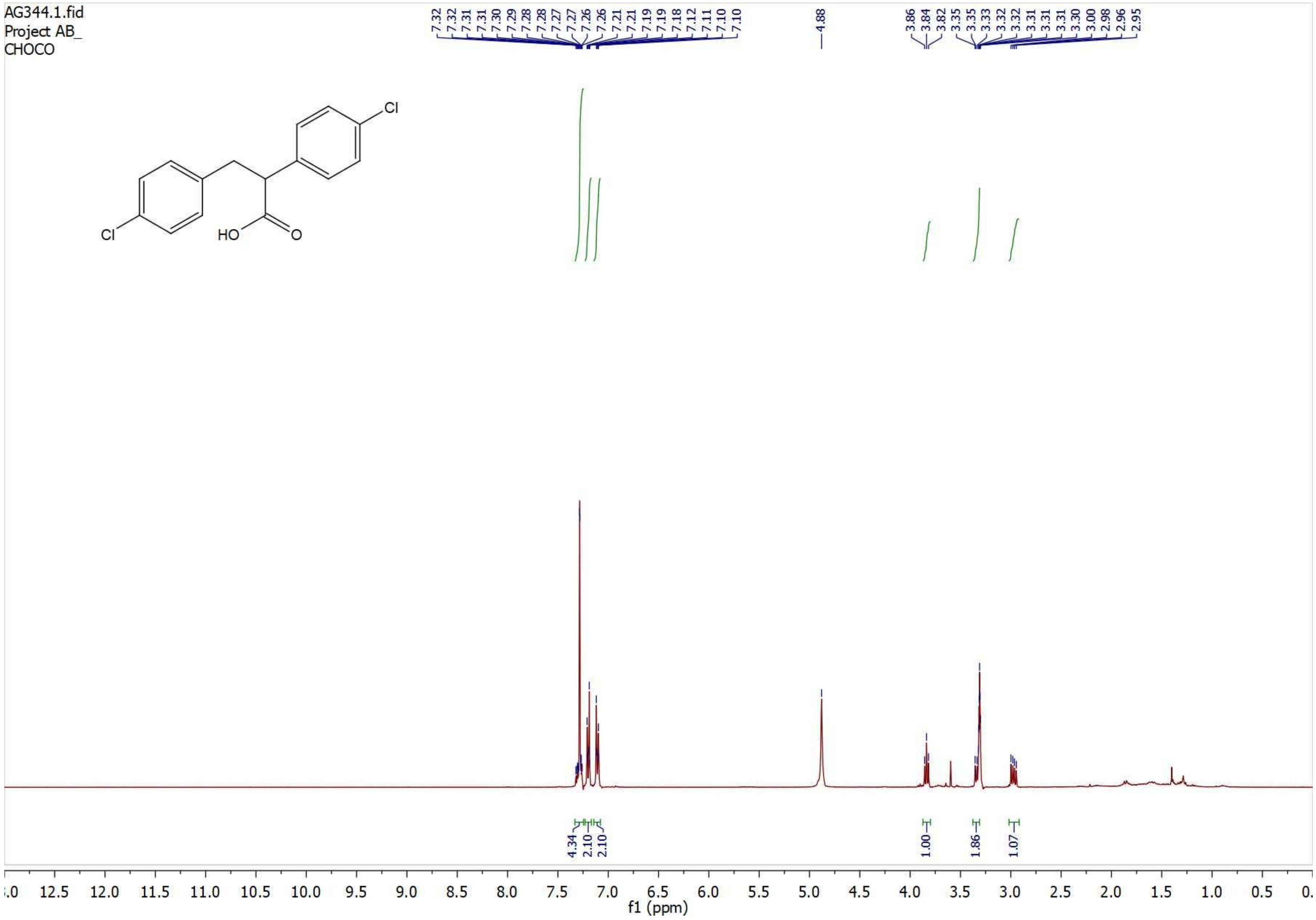
AG344.1.fid
Project AB_
CHOCO
Cl
Cl
HO
O
7.32
7.32
7.31
7.31
7.30
7.29
7.28
7.28
7.27
7.27
7.26
7.26
7.21
7.21
7.19
7.19
7.18
7.12
7.11
7.10
7.10
4.88
3.86
3.84
3.82
3.35
3.35
3.33
3.32
3.32
3.31
3.31
3.31
3.30
3.00
2.98
2.96
2.95
4.34
2.10
2.10
1.00
1.86
1.07
f1 (ppm)

Compound 2c

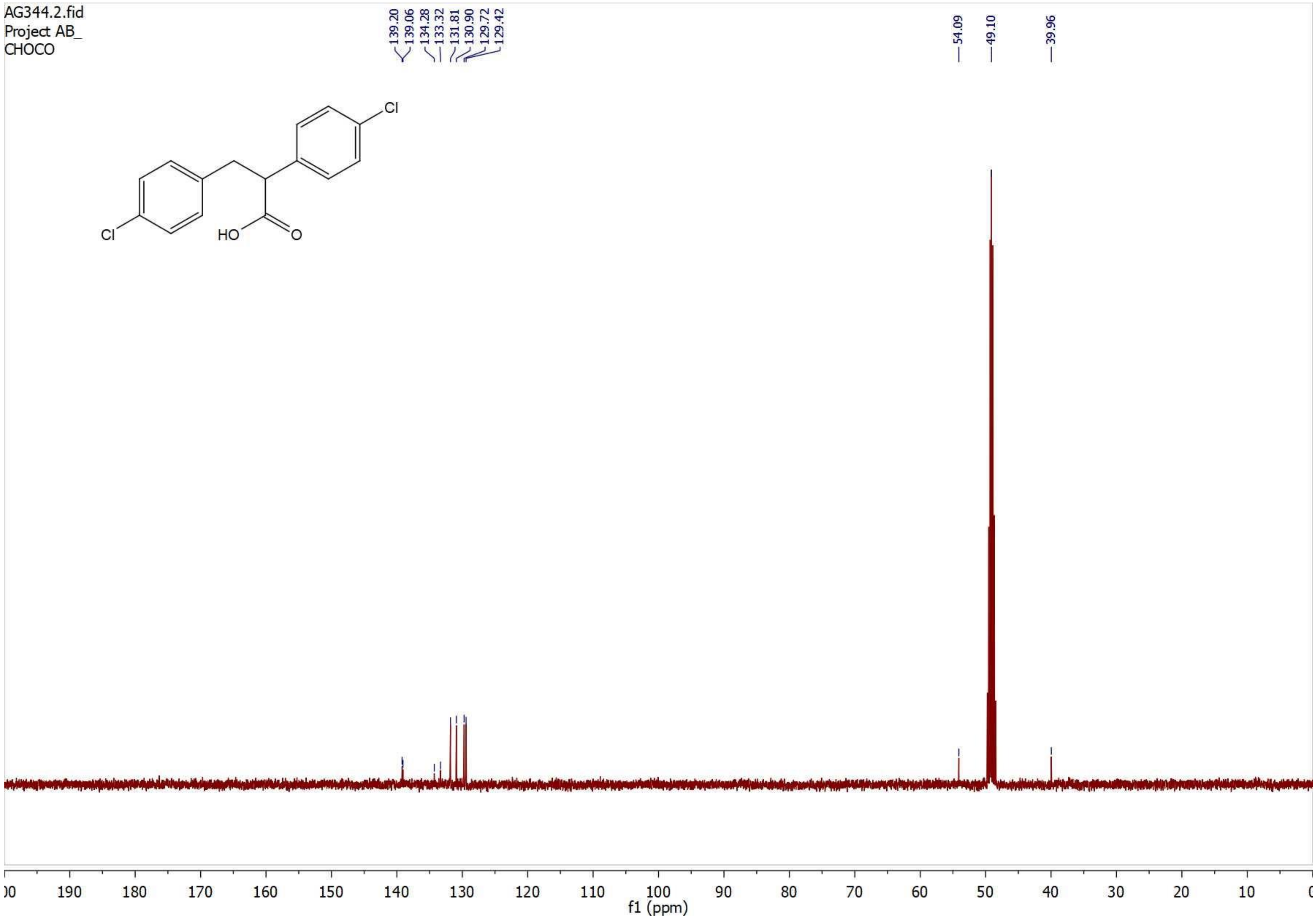

AG344.2.fid
Project AB_
CHOCO
139.20
139.06
134.28
133.32
131.81
130.90
129.72
129.42
54.09
49.10
39.96
Cl
Cl
HO
O
190
180
170
160
150
140
130
120
110
100
90
80
70
60
50
40
30
20
10
f1 (ppm)

Compound 2d

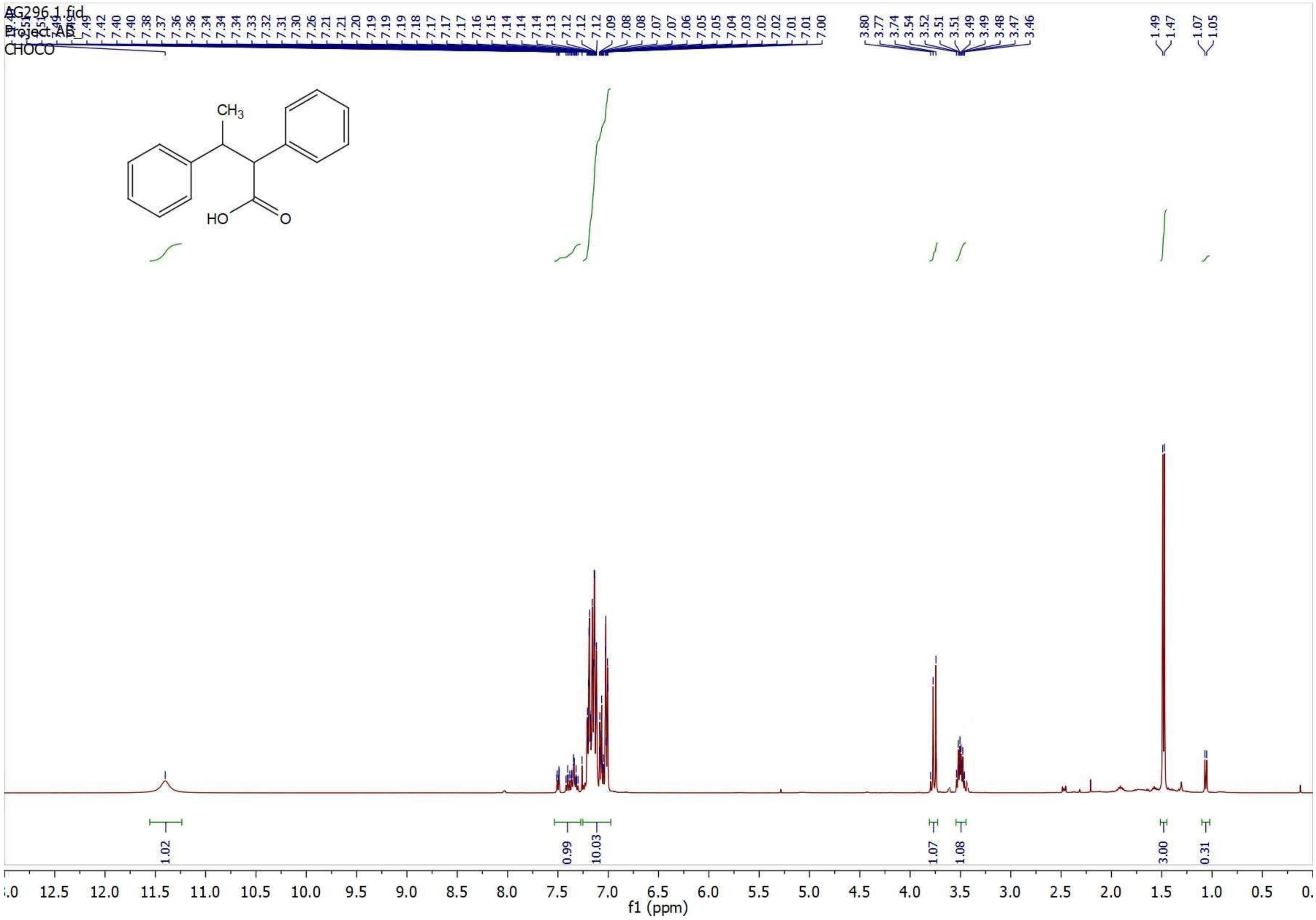

Compound 2d

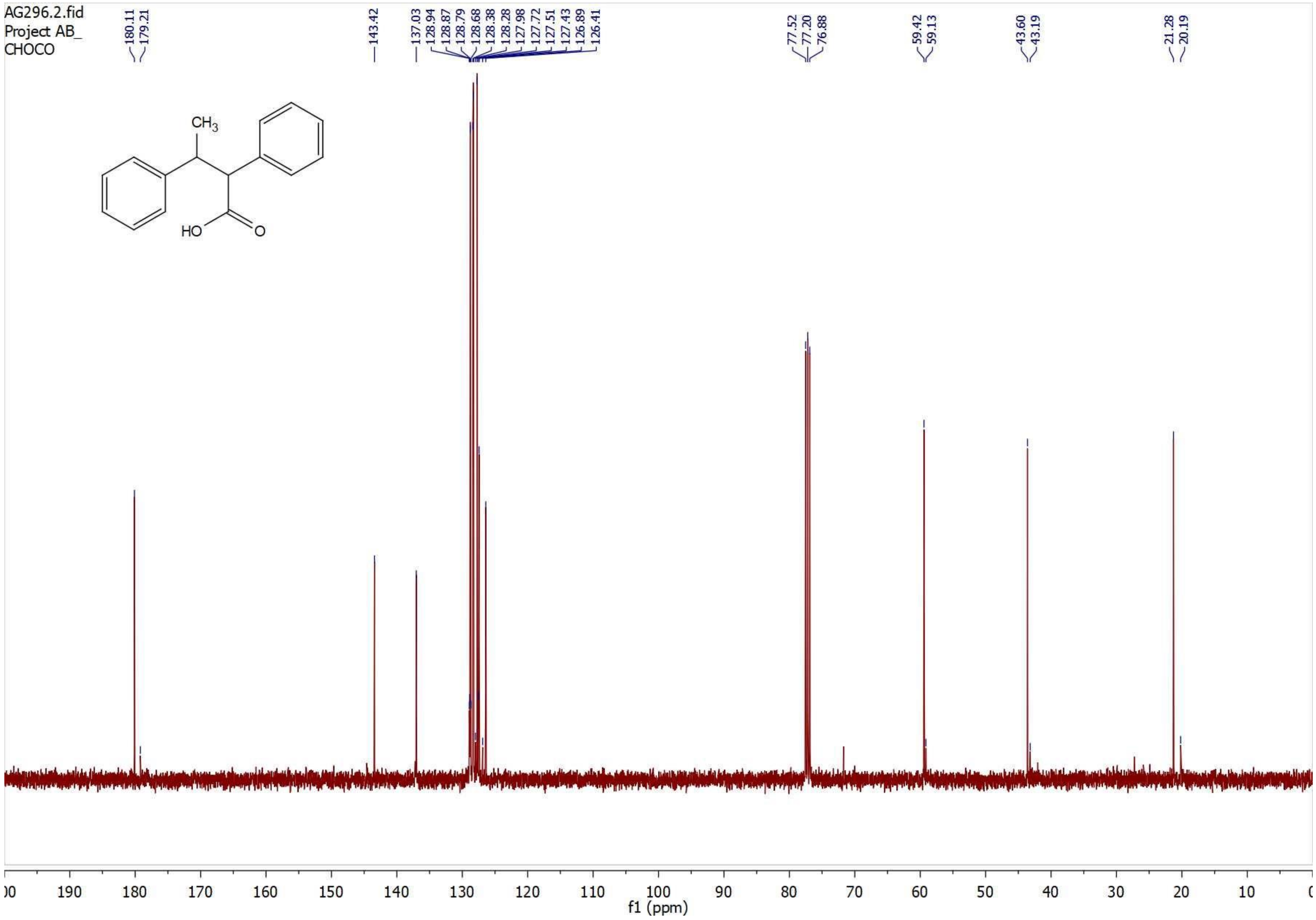
AG296.2.fid
Project AB_
CHOCO
180.11
179.21
143.42
137.03
128.94
128.87
128.79
128.68
128.38
128.28
127.98
127.72
127.51
127.43
126.89
126.41
77.52
77.20
76.88
59.42
59.13
43.60
43.19
21.28
20.19
CH3
HO
O
f1 (ppm)

Chromatogram of compound 2d

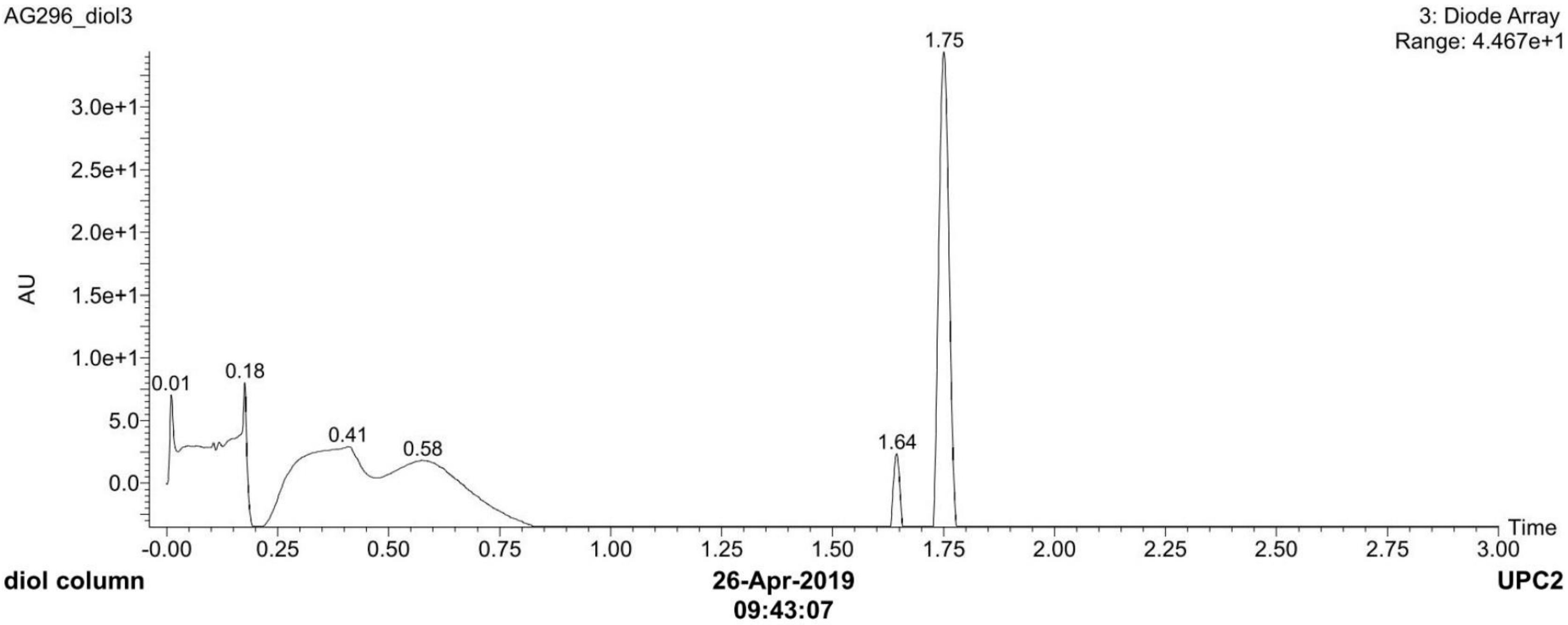

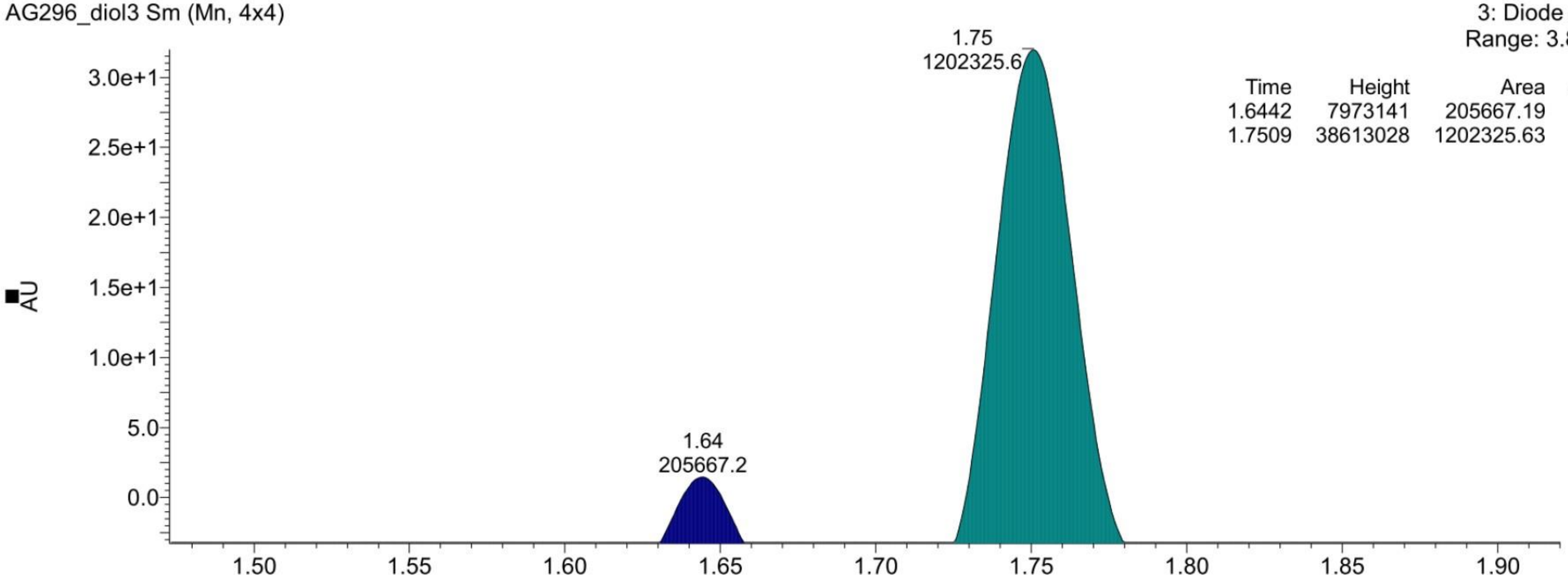

Time	Height	Area	Area Area%
1.6442	7973141	205667.19	14.61
1.7509	38613028	1202325.63	85.39

Compound 2e

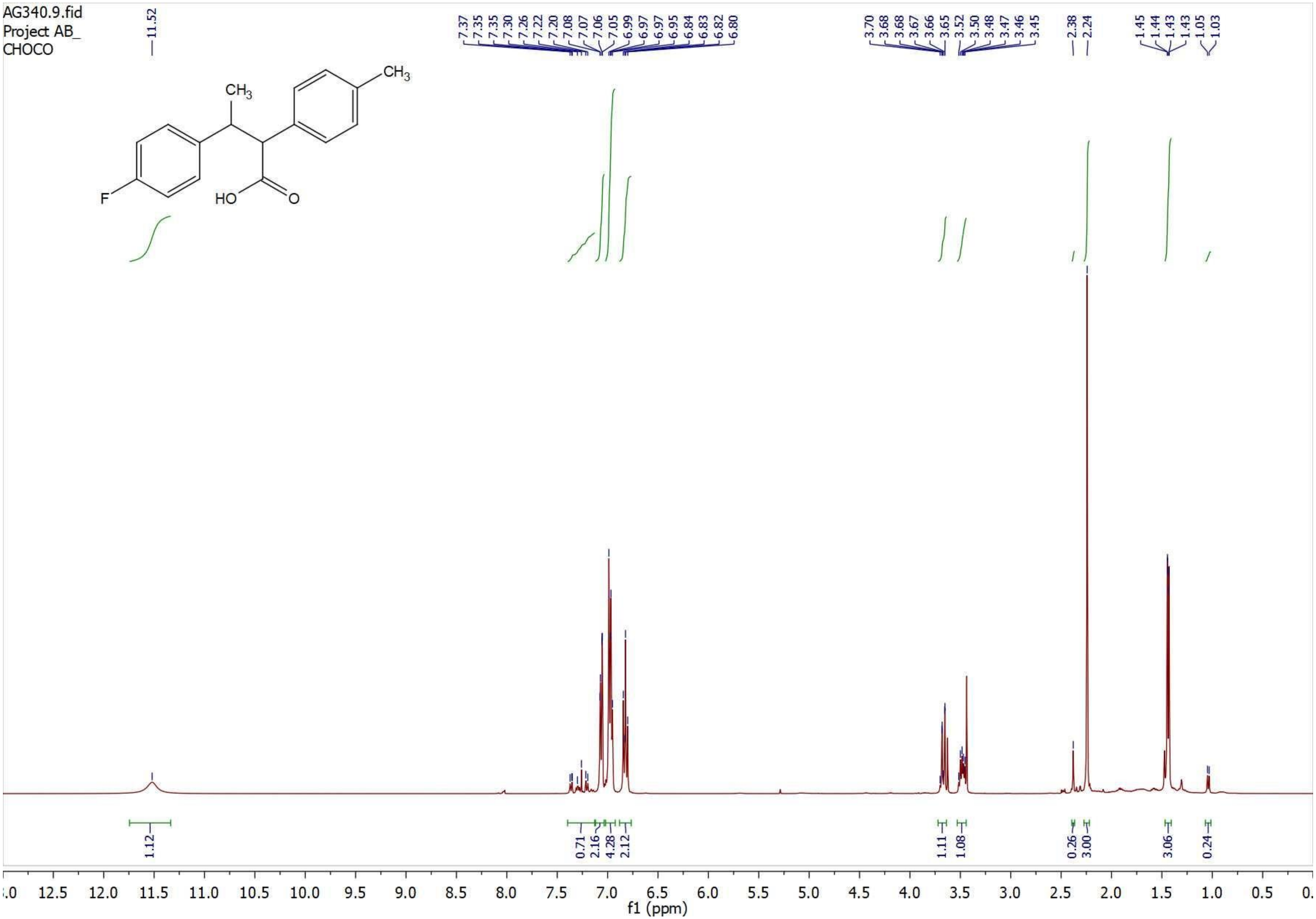
AG340.9.fid
Project AB_
CHOCO
11.52
7.37 7.35 7.35 7.30 7.26 7.22 7.20 7.08 7.07 7.06 7.05 6.99 6.97 6.97 6.95 6.84 6.83 6.82 6.80
3.70 3.68 3.68 3.67 3.66 3.65 3.52 3.50 3.48 3.47 3.46 3.45
2.38 2.24
1.45 1.44 1.43 1.43 1.05 1.03
CH3
CH3
F
HO
O
1.12
0.71 2.16 4.28 2.12
1.11 1.08
0.26 3.00
3.06
0.24
13.0 12.5 12.0 11.5 11.0 10.5 10.0 9.5 9.0 8.5 8.0 7.5 7.0 6.5 6.0 5.5 5.0 4.5 4.0 3.5 3.0 2.5 2.0 1.5 1.0 0.5 0.
f1 (ppm)

Compound 2e

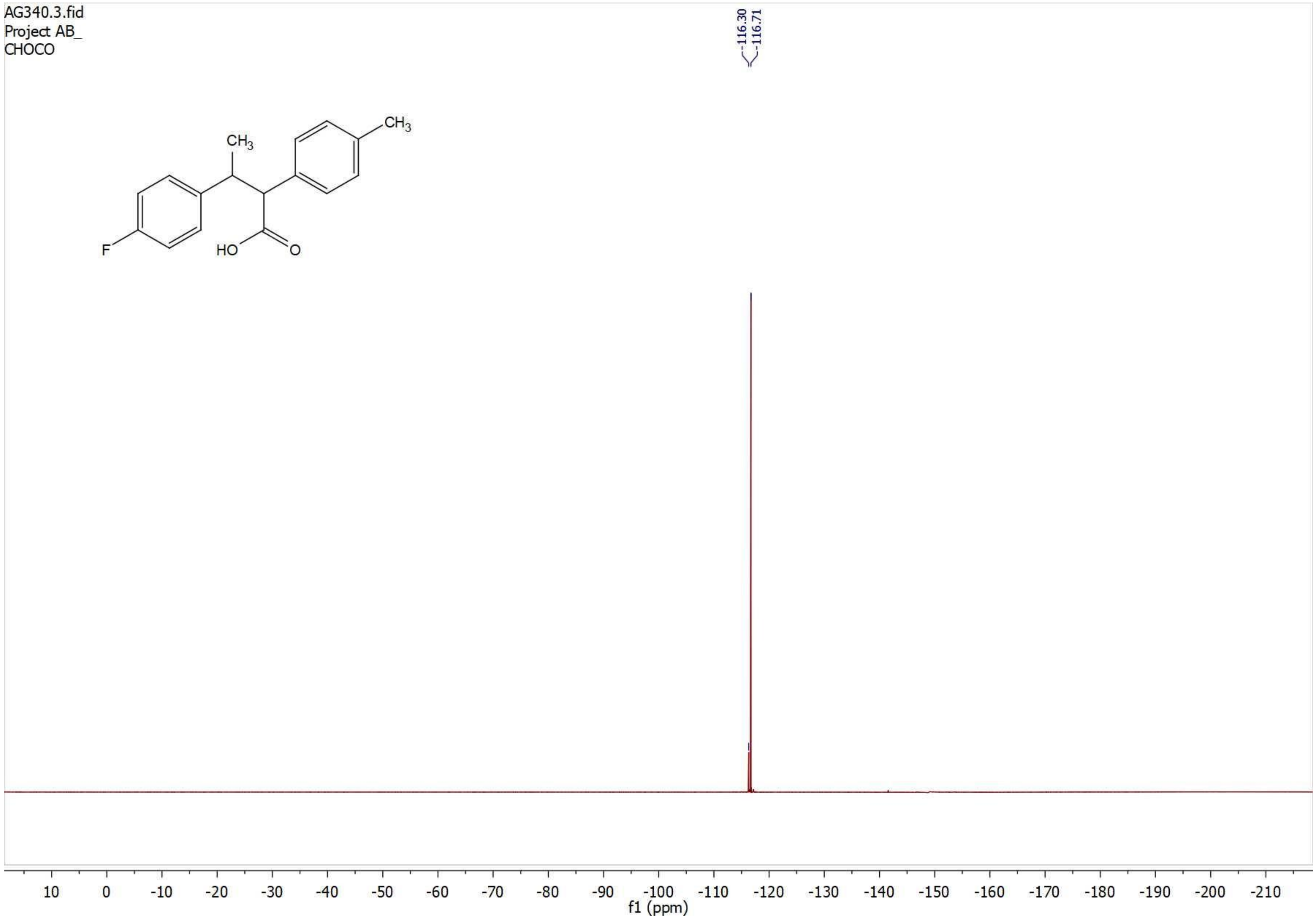
AG340.3.fid
Project AB_
CHOCO
-116.30
-116.71
CH3
CH3
F
HO
O
10
0
-10
-20
-30
-40
-50
-60
-70
-80
-90
-100
-110
-120
-130
-140
-150
-160
-170
-180
-190
-200
-210
f1 (ppm)

Compound 2e

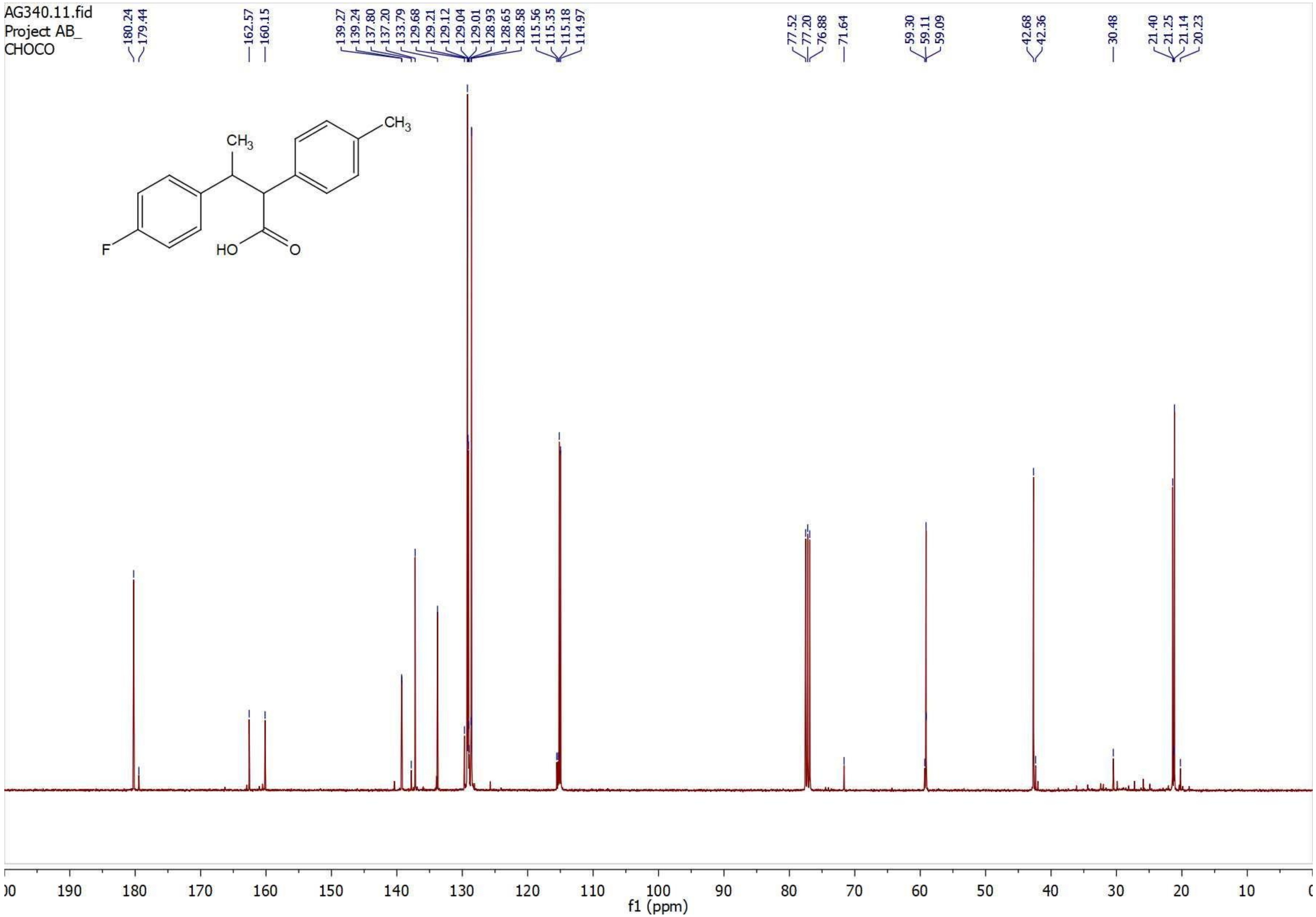
AG340.11.fid
Project AB_
CHOCO
180.24
179.44
162.57
160.15
139.27
139.24
137.80
137.20
133.79
129.68
129.21
129.12
129.04
129.01
128.93
128.65
128.58
115.56
115.35
115.18
114.97
77.52
77.20
76.88
71.64
59.30
59.11
59.09
42.68
42.36
30.48
21.40
21.25
21.14
20.23
CH3
CH3
F
HO
O
f1 (ppm)

Chromatogram of compound 2e

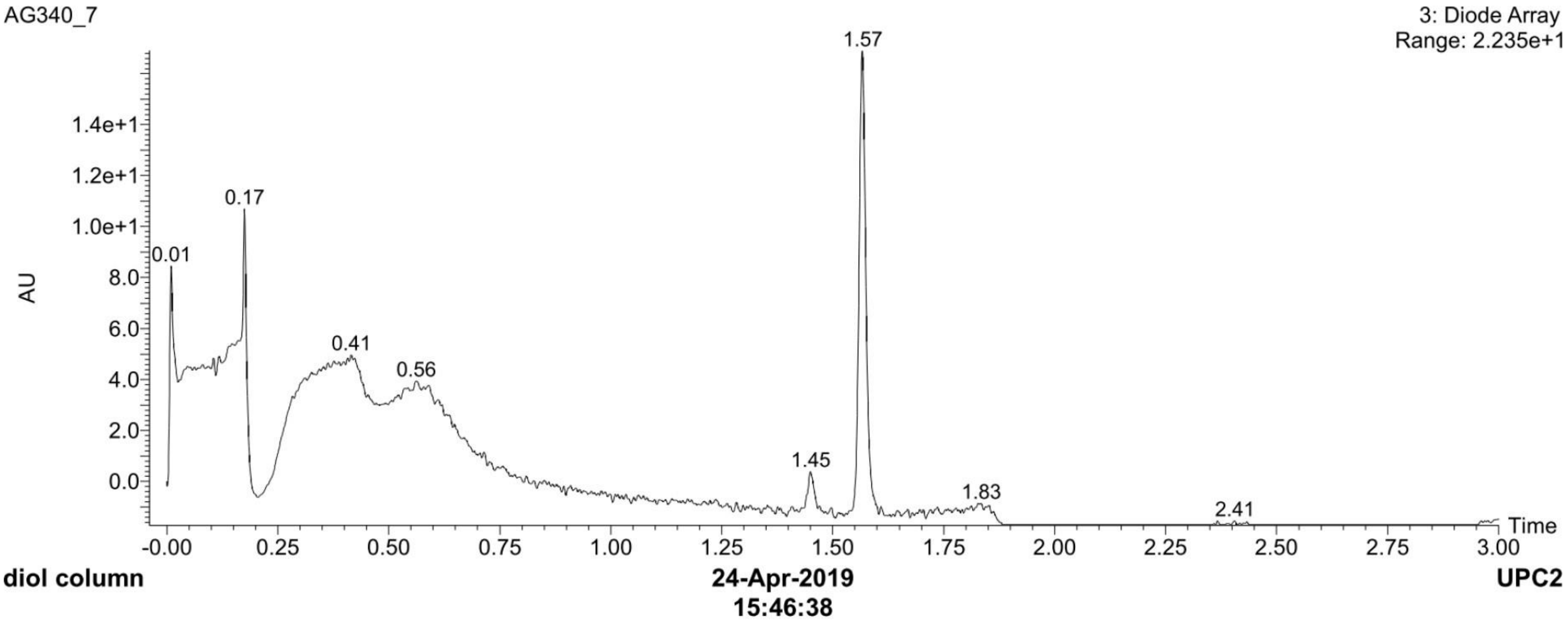

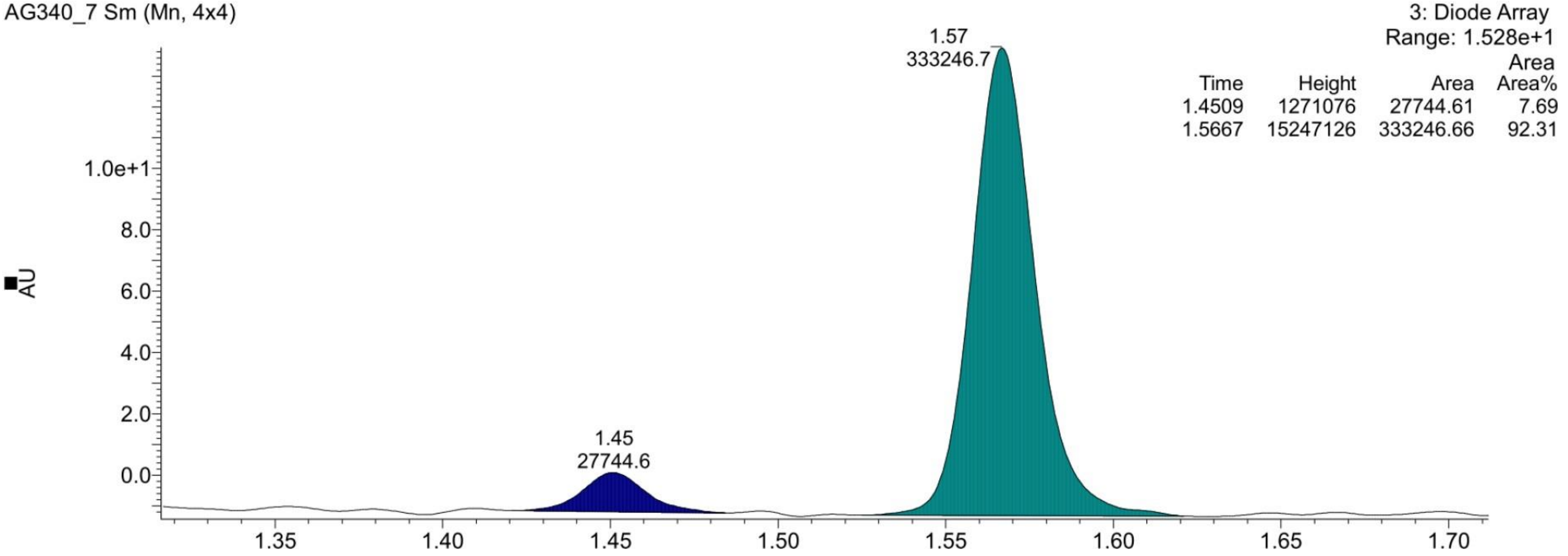

Time	Height	Area	Area Area%
1.4509	1271076	27744.61	7.69
1.5667	15247126	333246.66	92.31

Compound 3a

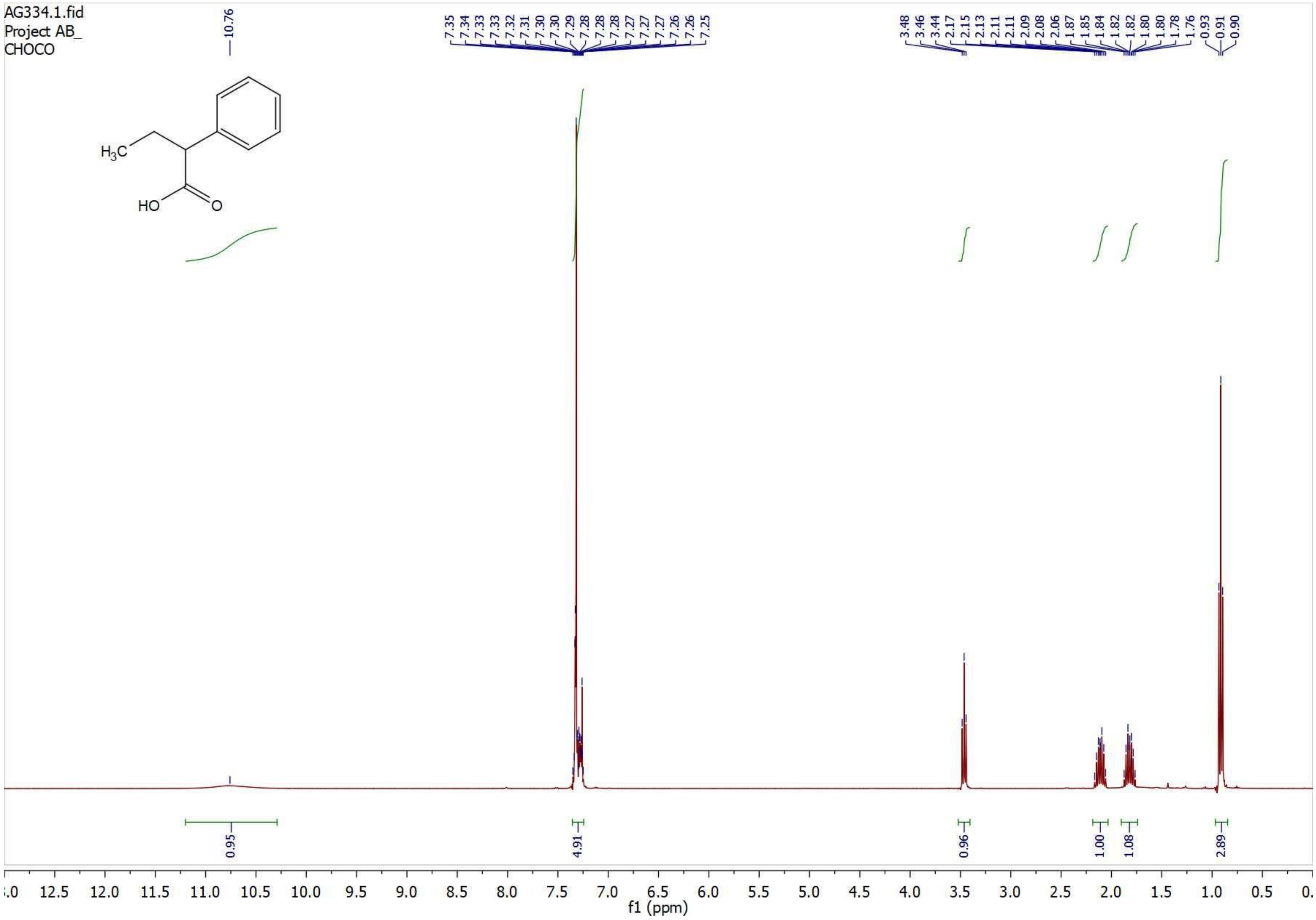
AG334.1.fid
Project AB_
CHOCO
10.76
7.35 7.34 7.33 7.33 7.32 7.31 7.30 7.30 7.29 7.28 7.28 7.28 7.27 7.27 7.27 7.26 7.26 7.25
3.48 3.46 3.44 2.17 2.15 2.13 2.11 2.11 2.09 2.08 2.06 1.87 1.85 1.84 1.82 1.82 1.80 1.80 1.78 1.76 0.93 0.91 0.90
H3C
HO
O
0.95
4.91
0.96
1.00
1.08
2.89
f1 (ppm)

Compound 3a

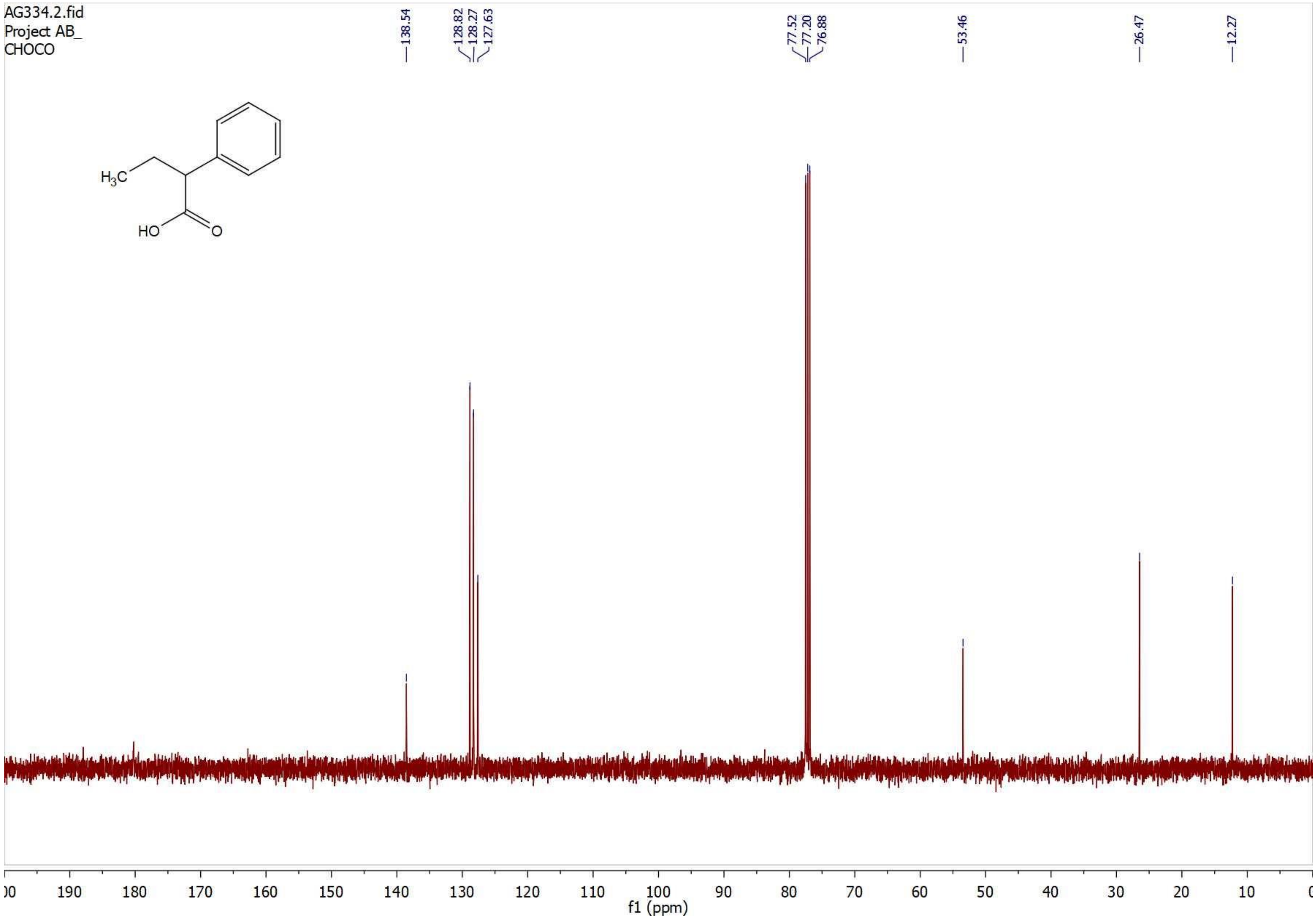
AG334.2.fid
Project AB_
CHOCO
138.54
128.82
128.27
127.63
77.52
77.20
76.88
53.46
26.47
12.27
H3C
HO
O
190
180
170
160
150
140
130
120
110
100
90
80
70
60
50
40
30
20
10
f1 (ppm)

Compound 3b

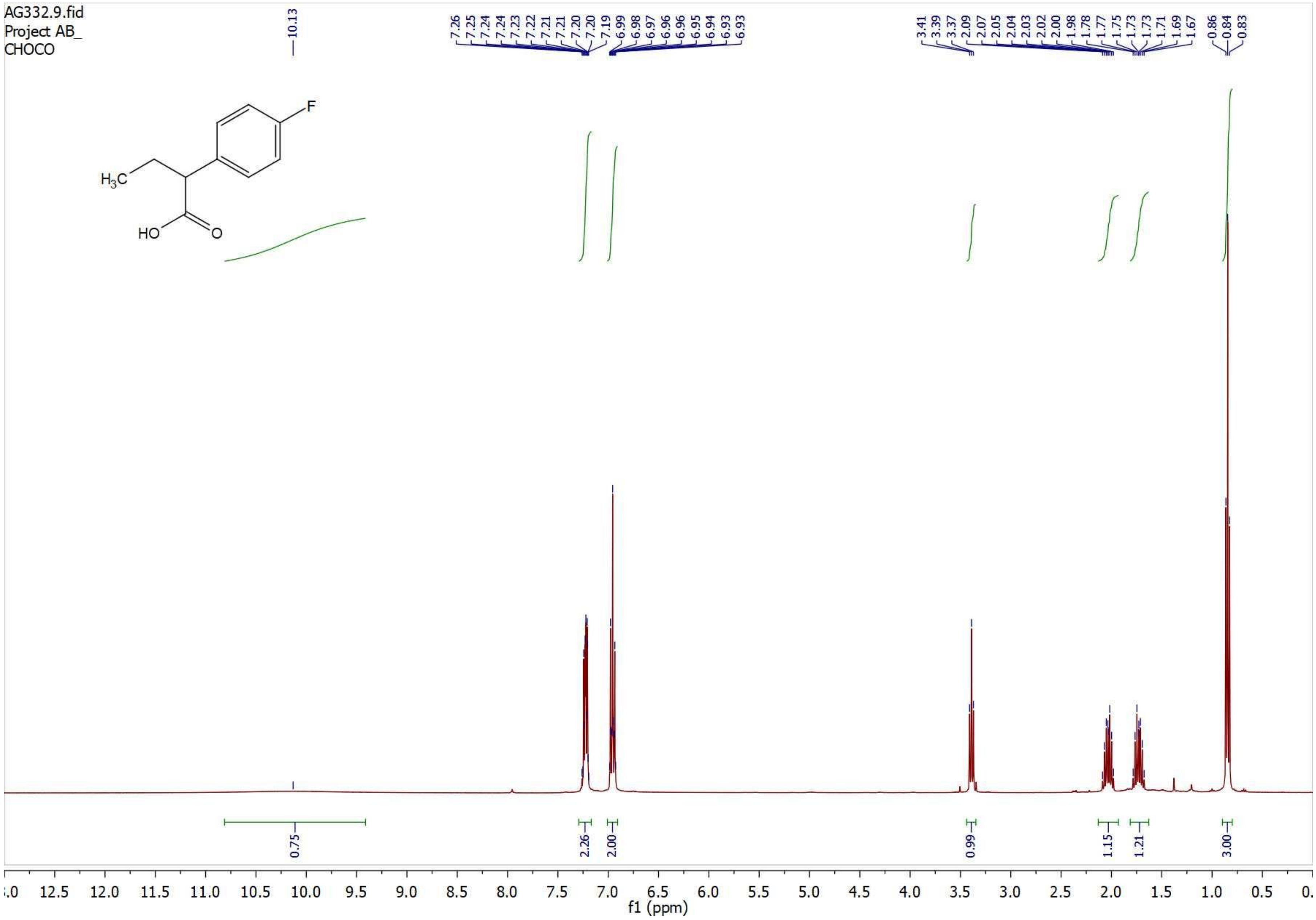

AG332.9.fid
Project AB_
CHOCO
10.13
7.26
7.25
7.24
7.24
7.23
7.22
7.21
7.21
7.20
7.20
7.19
6.99
6.98
6.97
6.96
6.96
6.95
6.94
6.93
6.93
3.41
3.39
3.37
2.09
2.07
2.05
2.04
2.03
2.02
2.00
1.98
1.78
1.77
1.75
1.73
1.73
1.71
1.69
1.67
0.86
0.84
0.83
F
H_3C
HO
O
0.75
2.26
2.00
0.99
1.15
1.21
3.00
f1 (ppm)

Compound 3b

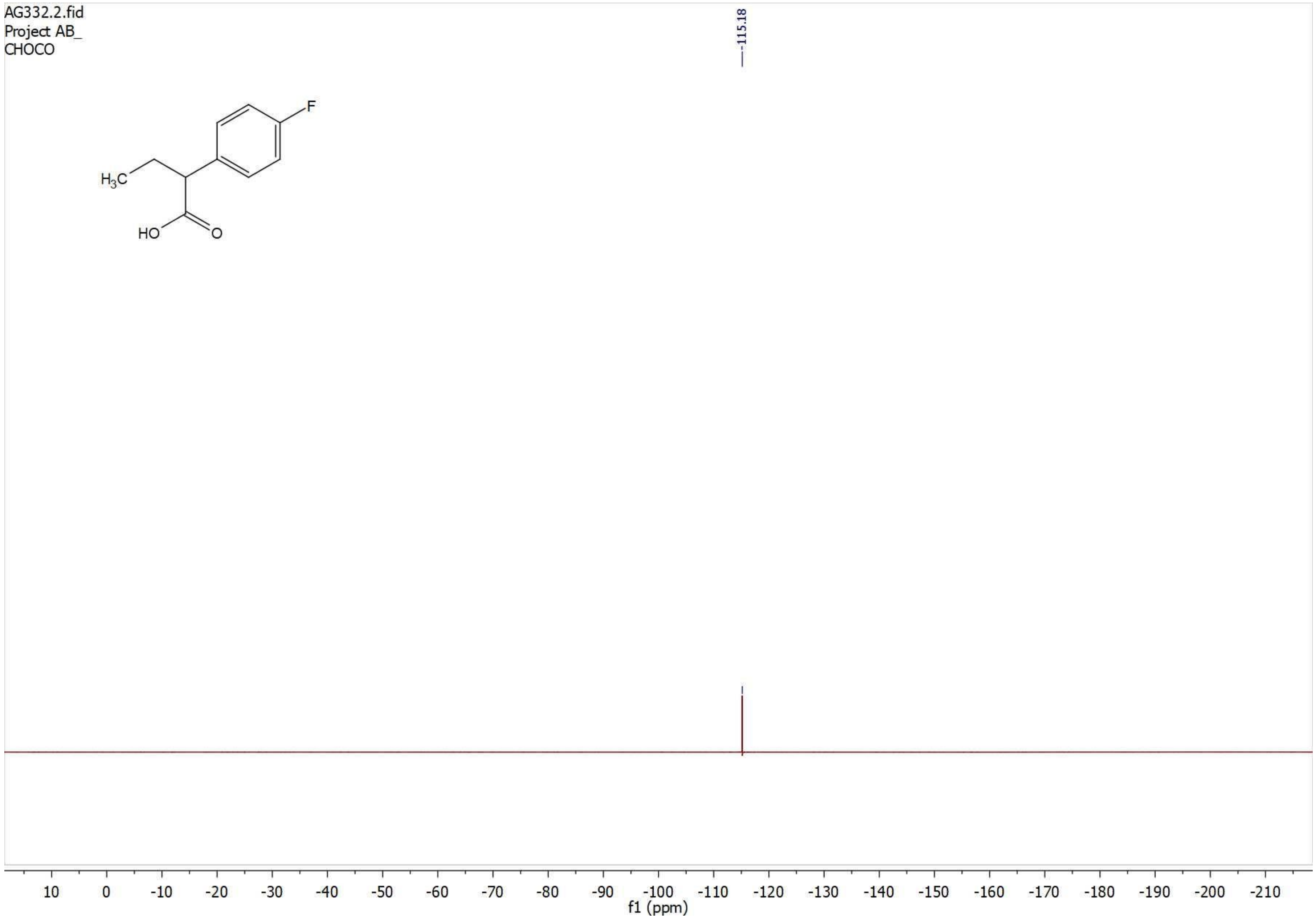
AG332.2.fid
Project AB_
CHOCO
-115.18
H3C
HO
O
F
10
0
-10
-20
-30
-40
-50
-60
-70
-80
-90
-100
-110
-120
-130
-140
-150
-160
-170
-180
-190
-200
-210
f1 (ppm)

Compound 3b

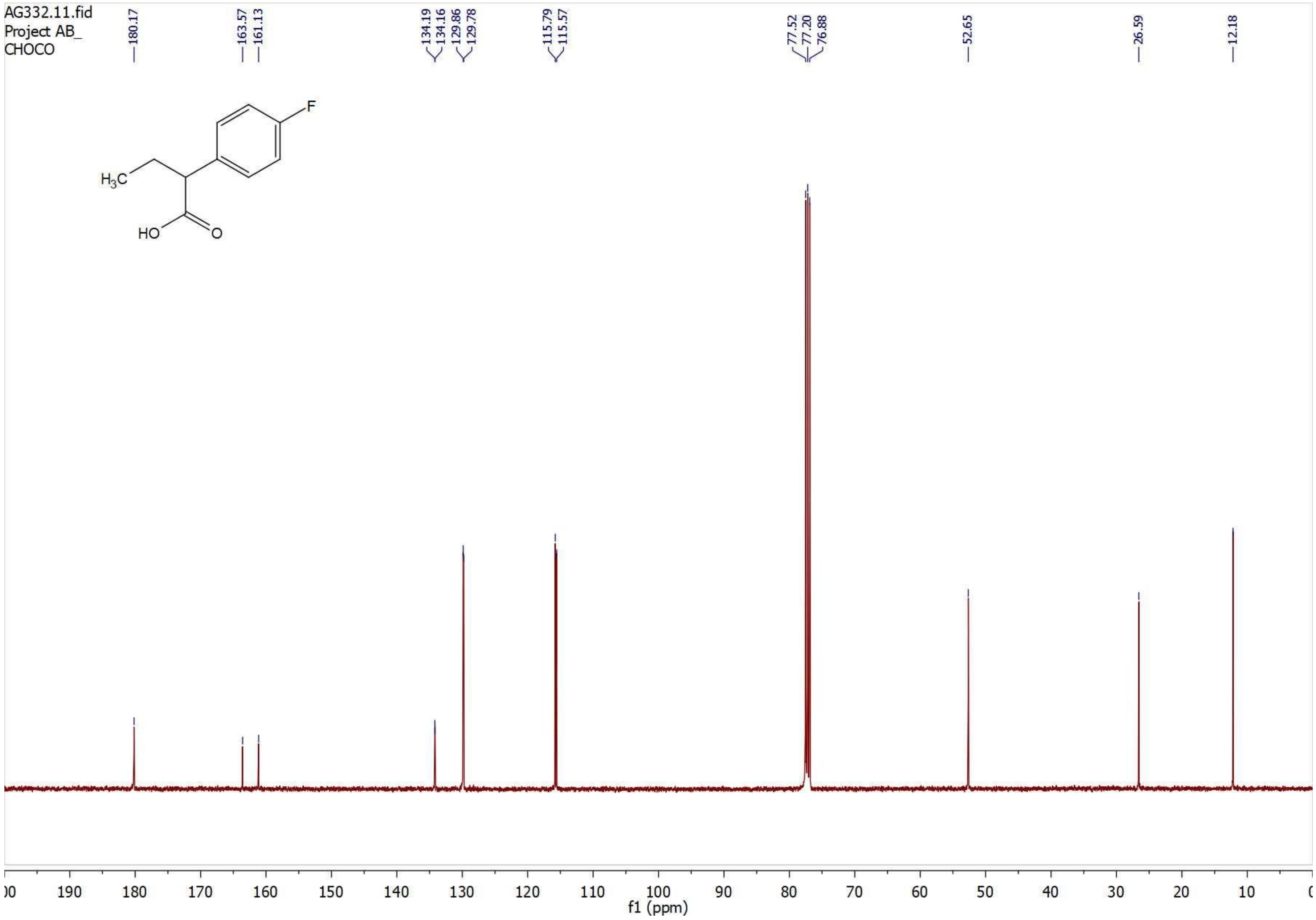

AG332.11.fid
Project AB_
CHOCO
180.17
163.57
161.13
134.19
134.16
129.86
129.78
115.79
115.57
77.52
77.20
76.88
52.65
26.59
12.18
F
H3C
HO
O
190
180
170
160
150
140
130
120
110
100
90
80
70
60
50
40
30
20
10
f1 (ppm)

Compound 3c

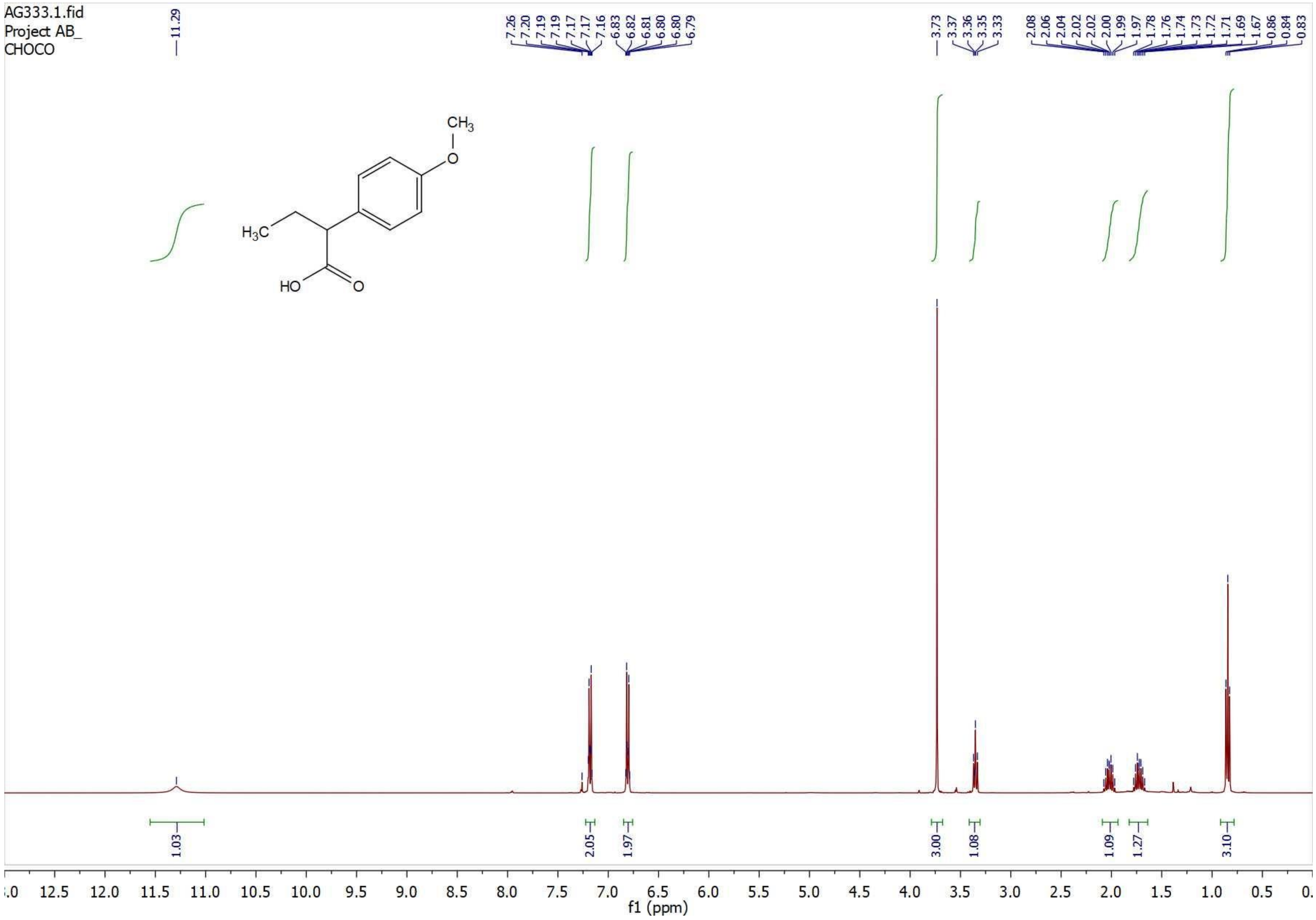
AG333.1.fid
Project AB_
CHOCO
11.29
7.26
7.20
7.19
7.19
7.17
7.17
7.16
6.83
6.82
6.81
6.80
6.80
6.79
3.73
3.37
3.36
3.35
3.33
2.08
2.06
2.04
2.02
2.02
2.00
1.99
1.97
1.78
1.76
1.74
1.73
1.72
1.71
1.69
1.67
0.86
0.84
0.83
CH3
O
H3C
HO
O
1.03
2.05
1.97
3.00
1.08
1.09
1.27
3.10
f1 (ppm)

Compound 3c

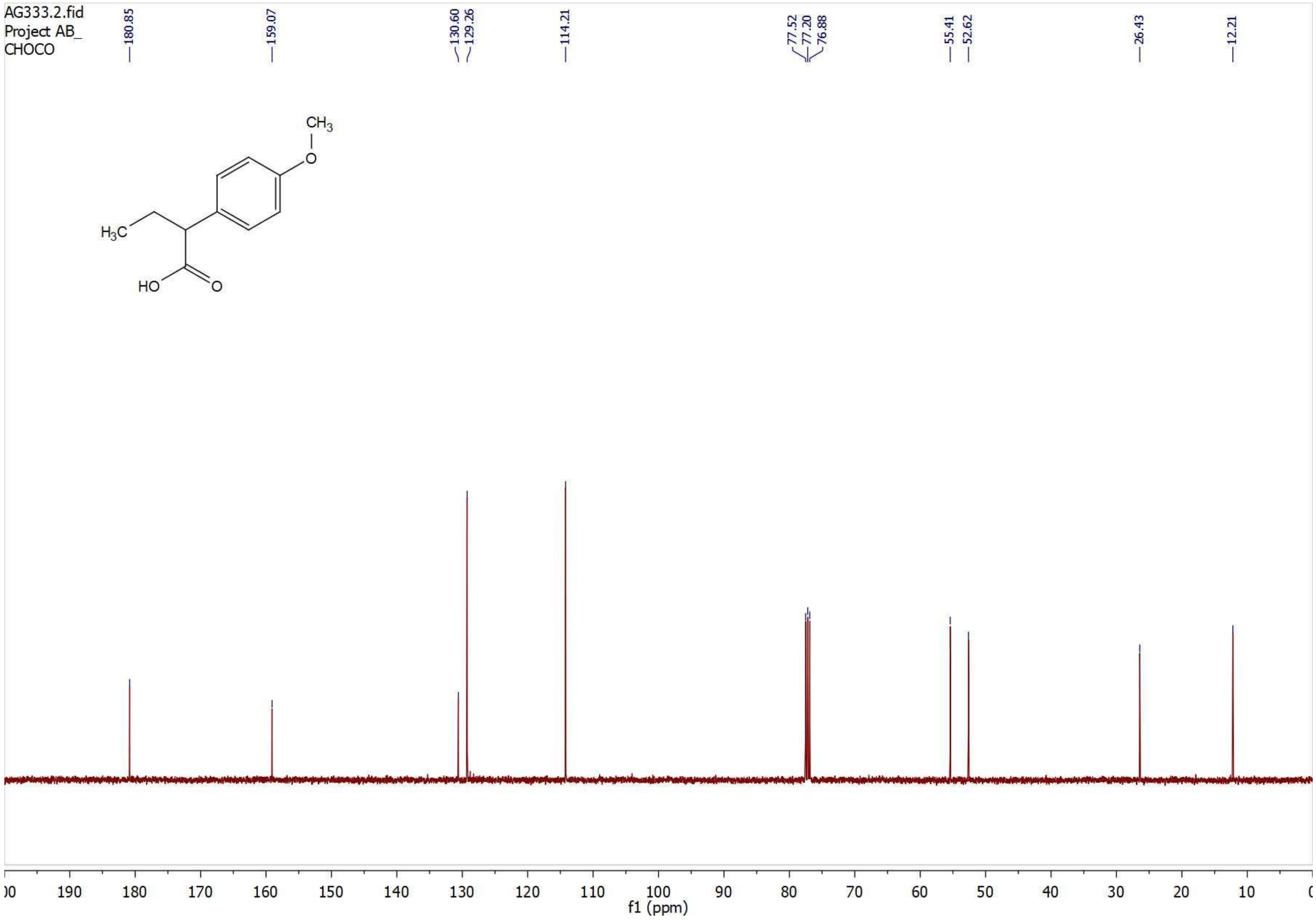

AG333.2.fid
Project AB_
CHOCO
180.85
159.07
130.60
129.26
114.21
77.52
77.20
76.88
55.41
52.62
26.43
12.21
CH3
O
H3C
HO
O
190
180
170
160
150
140
130
120
110
100
90
80
70
60
50
40
30
20
10
f1 (ppm)

Compound 3d

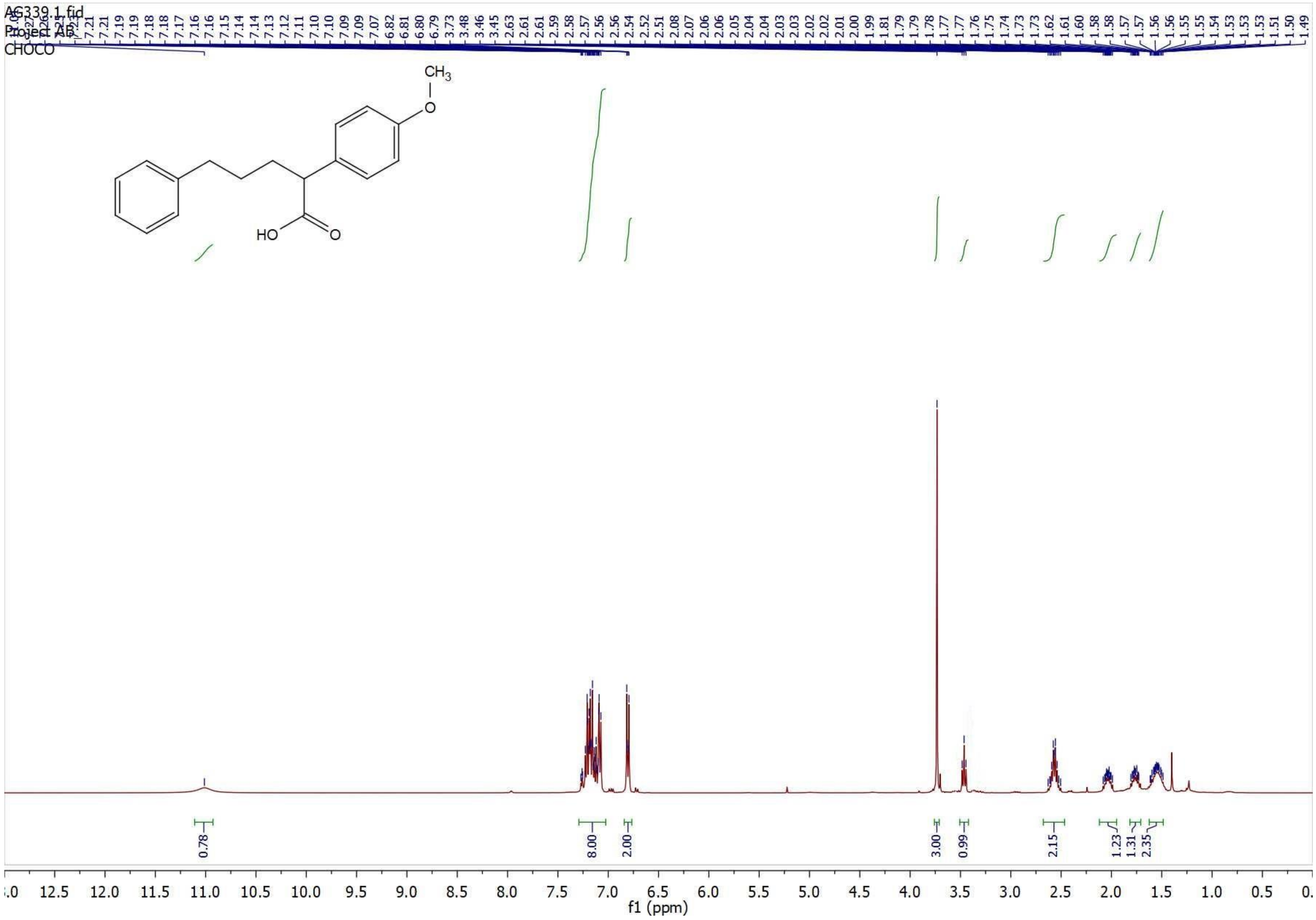

Compound 3d

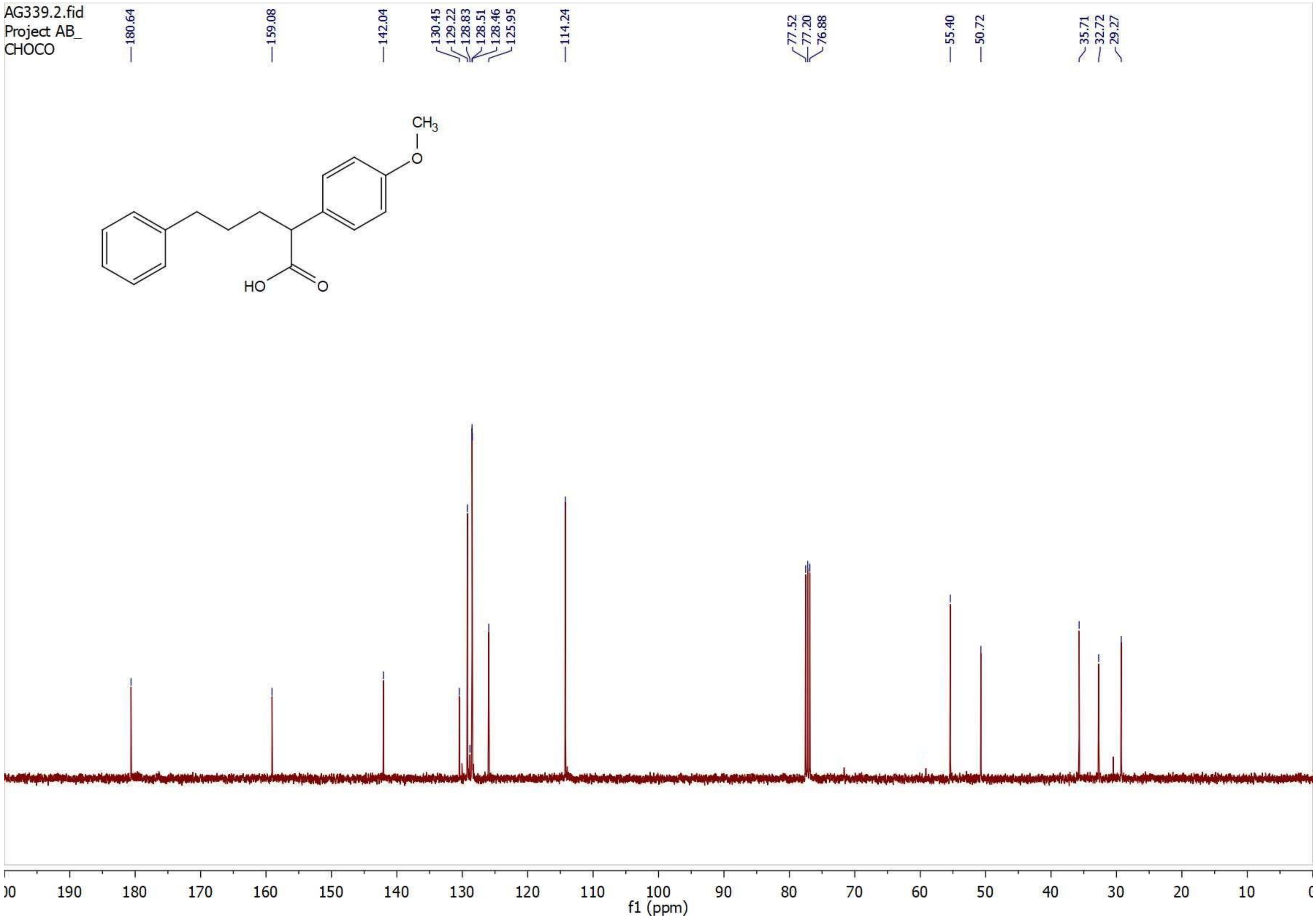
AG339.2.fid
Project AB_
CHOCO
180.64
159.08
142.04
130.45
129.22
128.83
128.51
128.46
125.95
114.24
77.52
77.20
76.88
55.40
50.72
35.71
32.72
29.27
CH3
O
HO
O
f1 (ppm)

Compound 3e

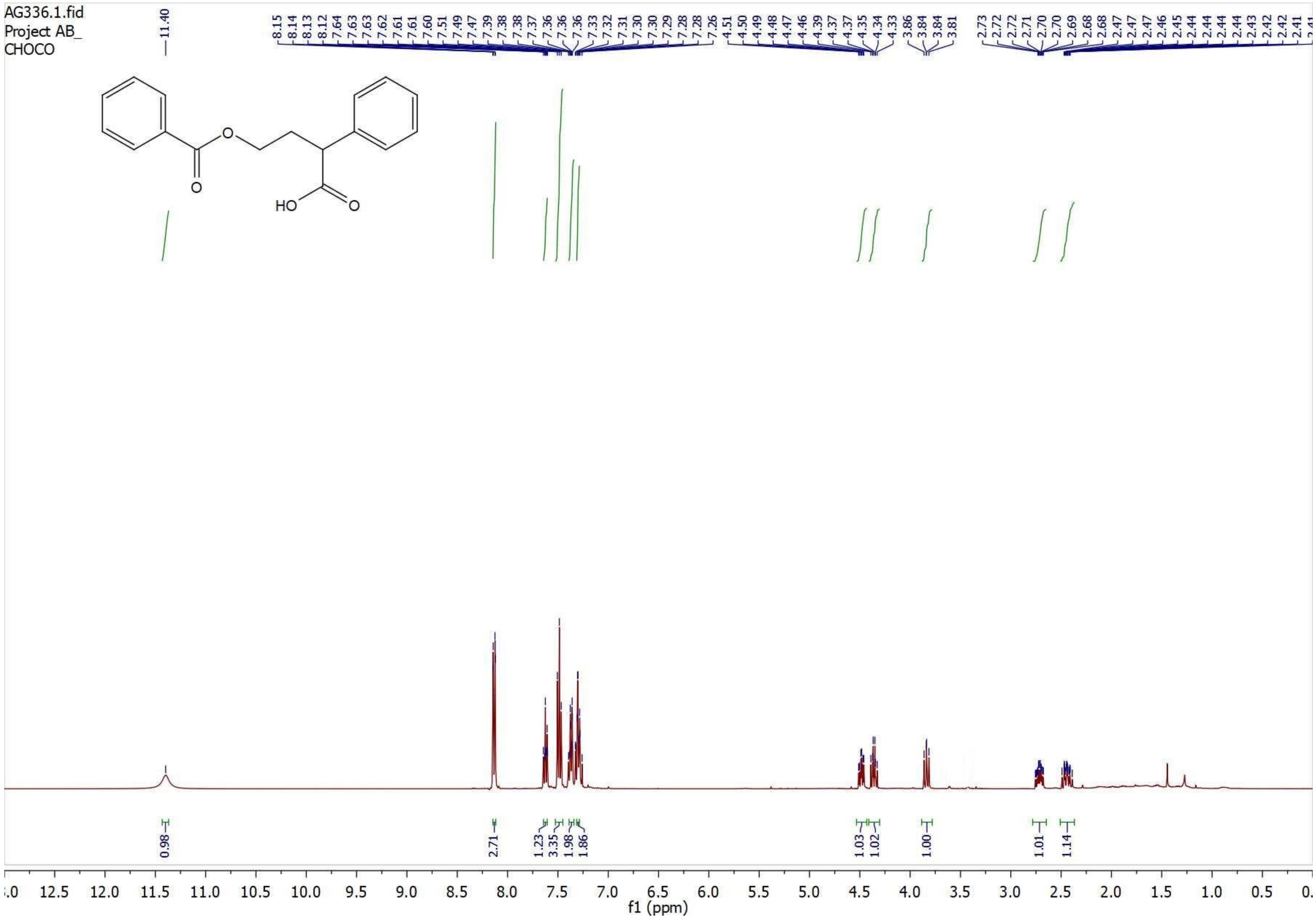

Compound 3e

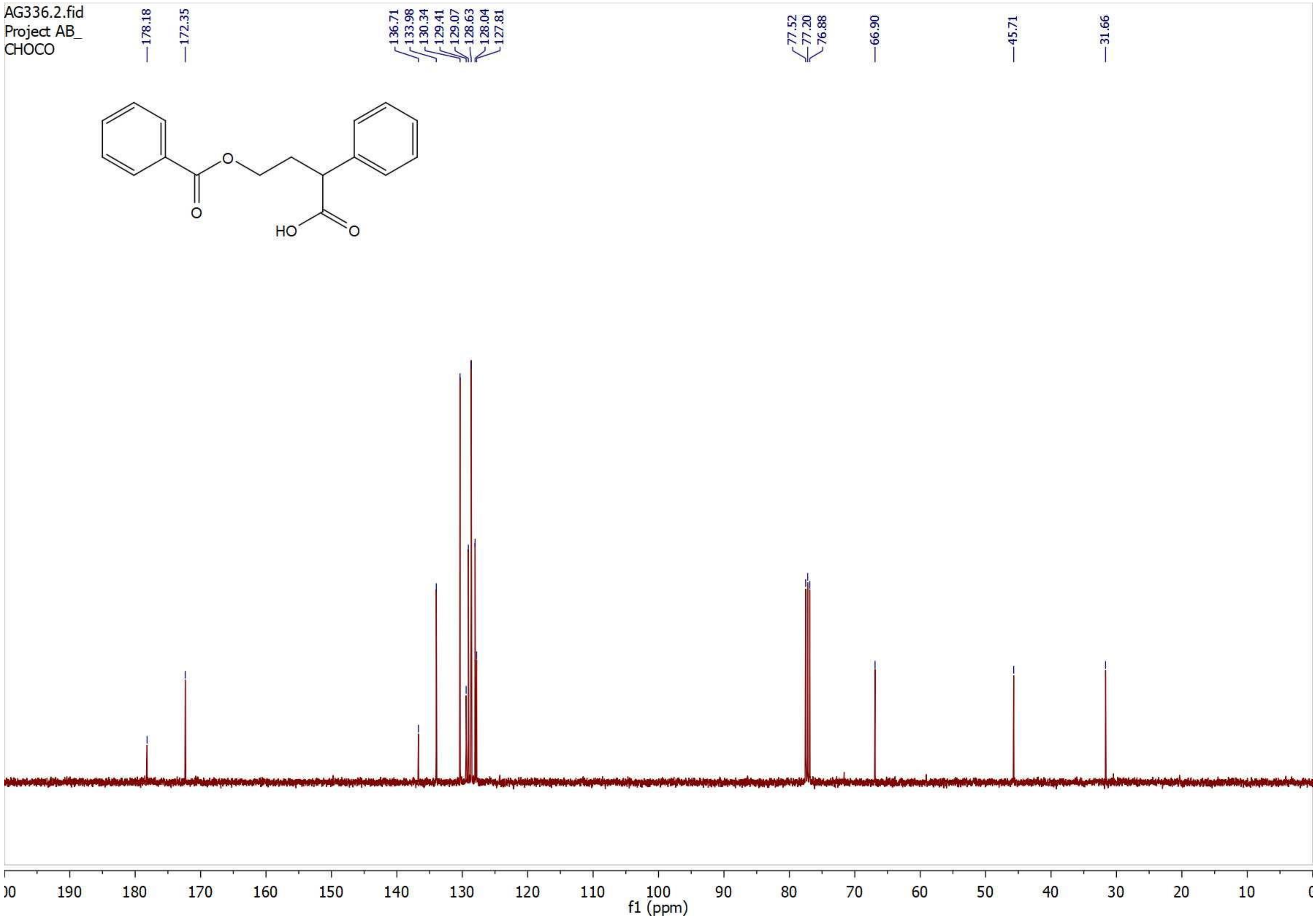
AG336.2.fid
Project AB_
CHOCO
178.18
172.35
136.71
133.98
130.34
129.41
129.07
128.63
128.04
127.81
77.52
77.20
76.88
66.90
45.71
31.66
HO
O
O
O
190
180
170
160
150
140
130
120
110
100
90
80
70
60
50
40
30
20
10
f1 (ppm)

Compound 3f

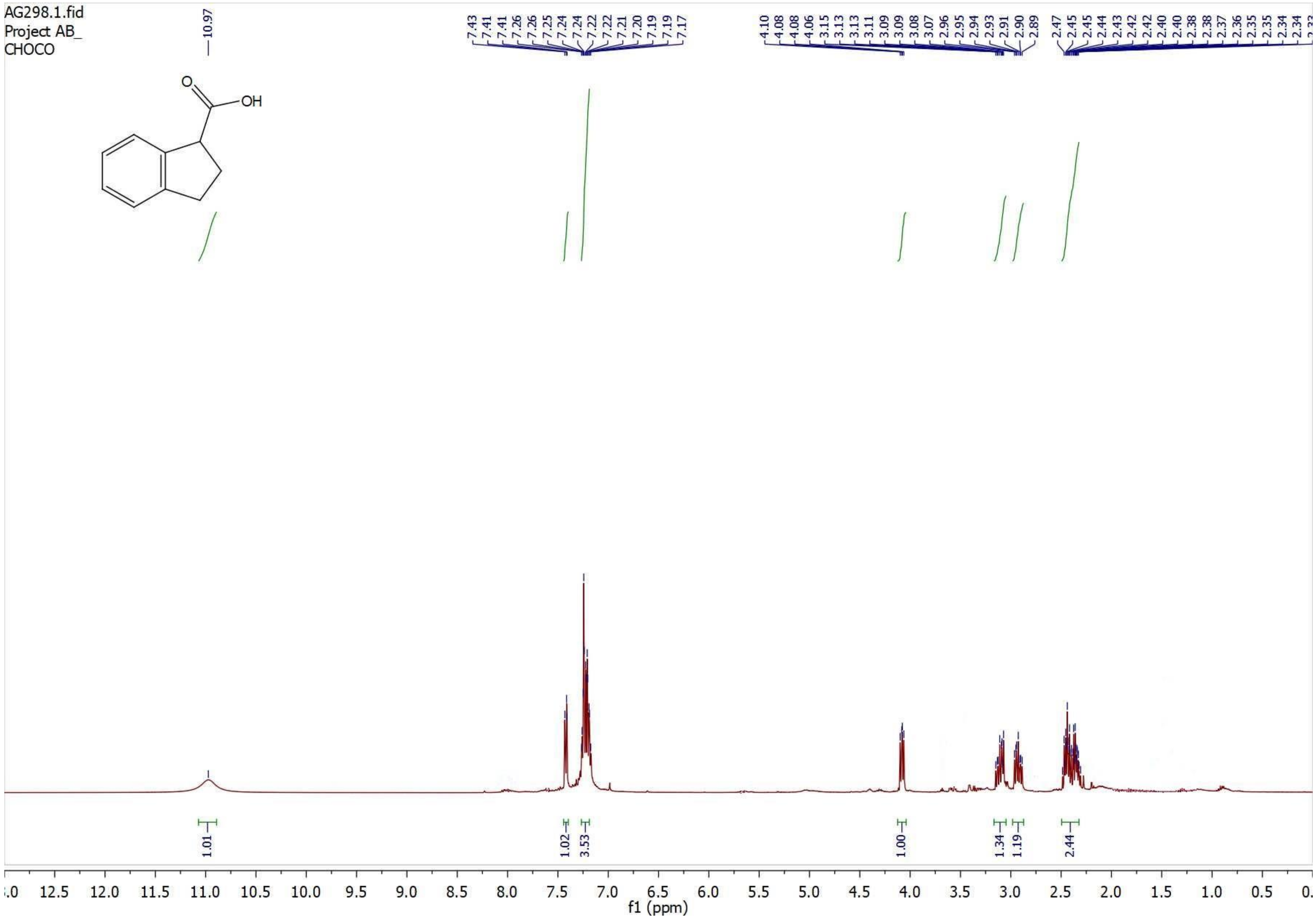
AG298.1.fid
Project AB_
CHOCO
OH
O
10.97
7.43 7.41 7.41 7.26 7.26 7.25 7.24 7.24 7.22 7.22 7.21 7.20 7.19 7.19 7.17
4.10 4.08 4.08 4.06 3.15 3.13 3.13 3.11 3.09 3.09 3.08 3.07 2.96 2.95 2.94 2.93 2.91 2.90 2.89
2.47 2.45 2.45 2.44 2.43 2.42 2.42 2.40 2.40 2.38 2.38 2.37 2.36 2.35 2.34 2.34
1.01
1.02
3.53
1.00
1.34
1.19
2.44
12.5 12.0 11.5 11.0 10.5 10.0 9.5 9.0 8.5 8.0 7.5 7.0 6.5 6.0 5.5 5.0 4.5 4.0 3.5 3.0 2.5 2.0 1.5 1.0 0.5
f1 (ppm)

Compound 3f

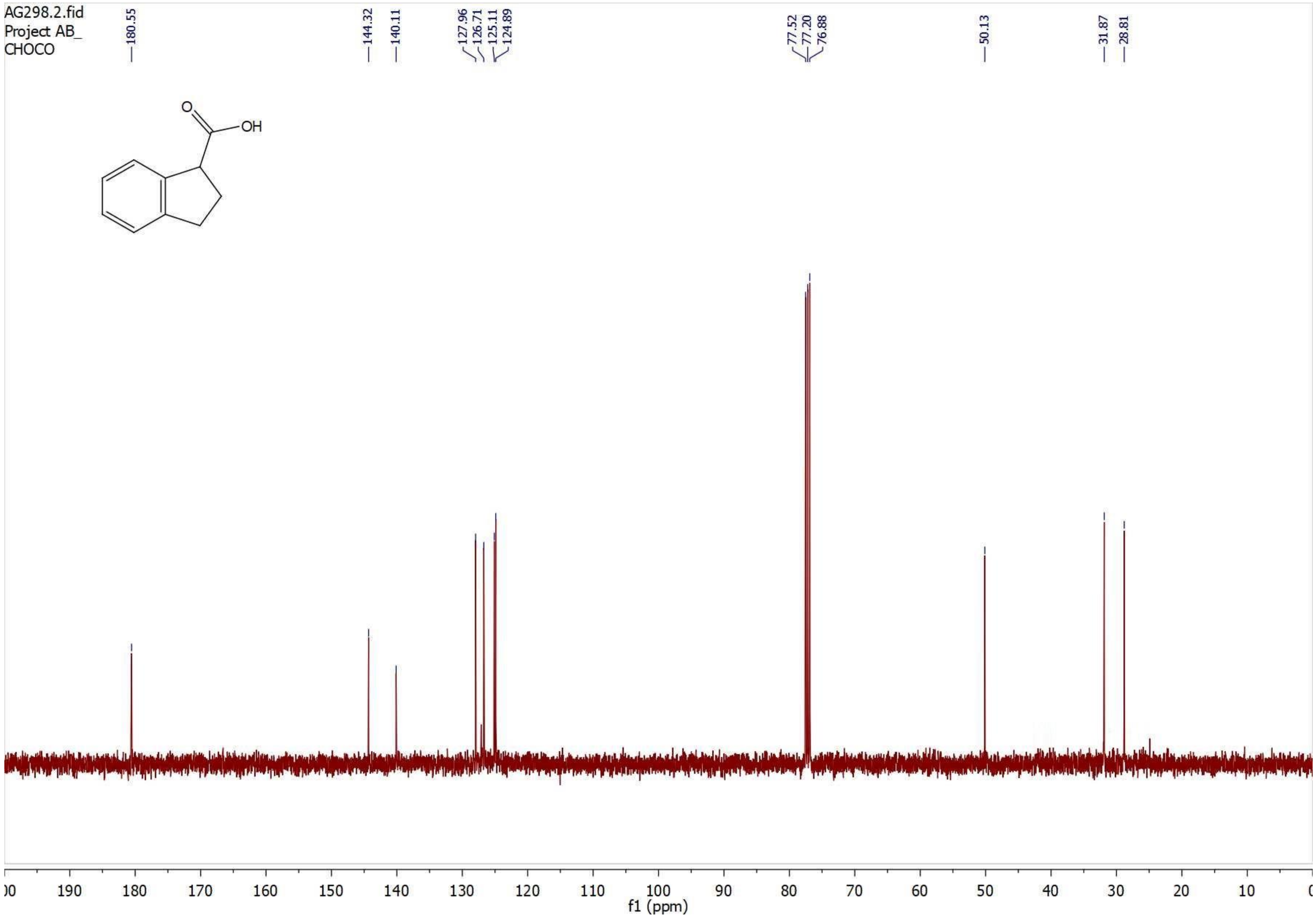
AG298.2.fid
Project AB_
CHOCO
180.55
144.32
140.11
127.96
126.71
125.11
124.89
77.52
77.20
76.88
50.13
31.87
28.81
OH
O
190
180
170
160
150
140
130
120
110
100
90
80
70
60
50
40
30
20
10
f1 (ppm)

Compound 3g

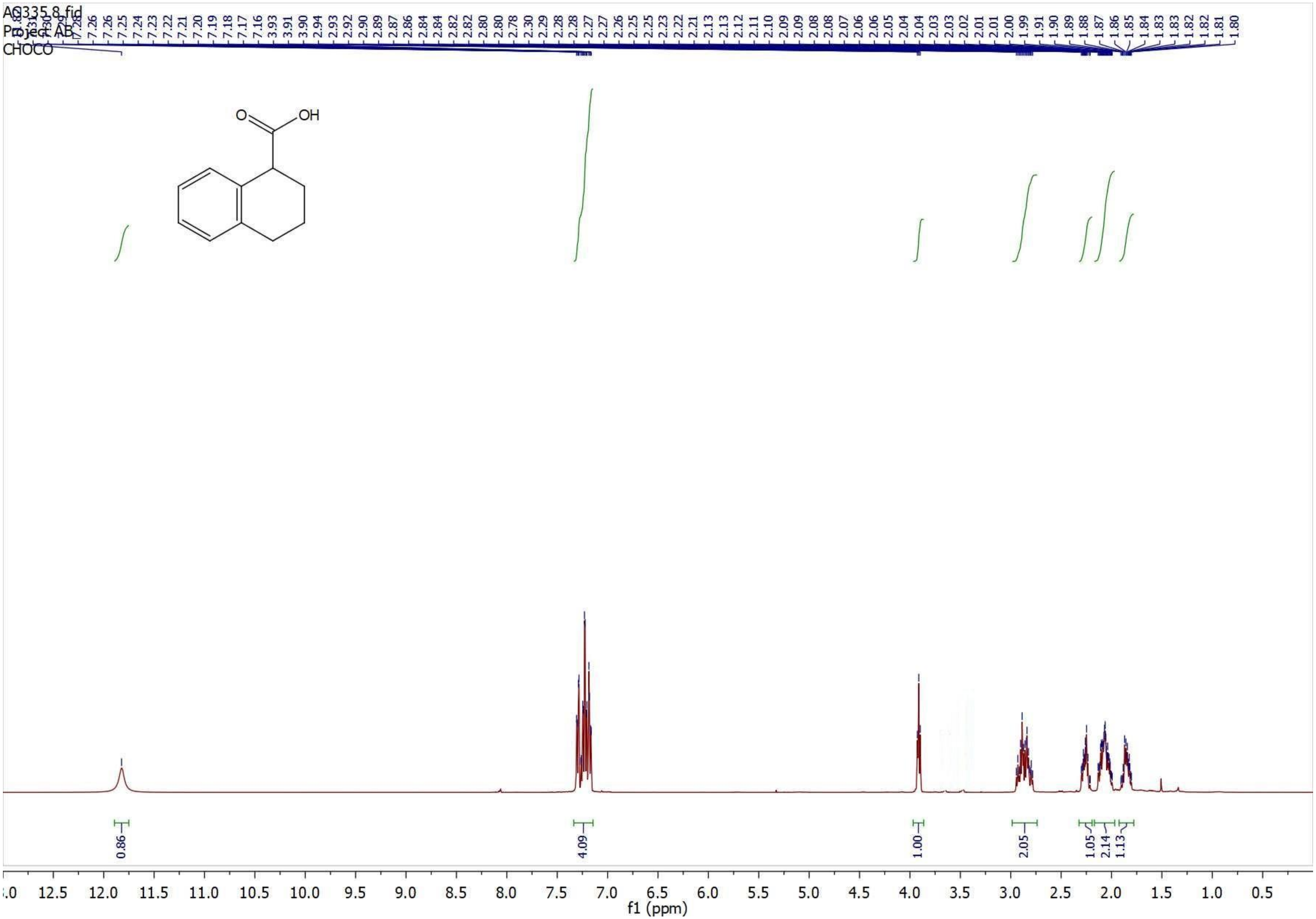
AG335.8.fid
Project AB
CHOCO
OH
O
0.86
4.09
1.00
2.05
1.05
2.14
1.13
f1 (ppm)

Compound 3g

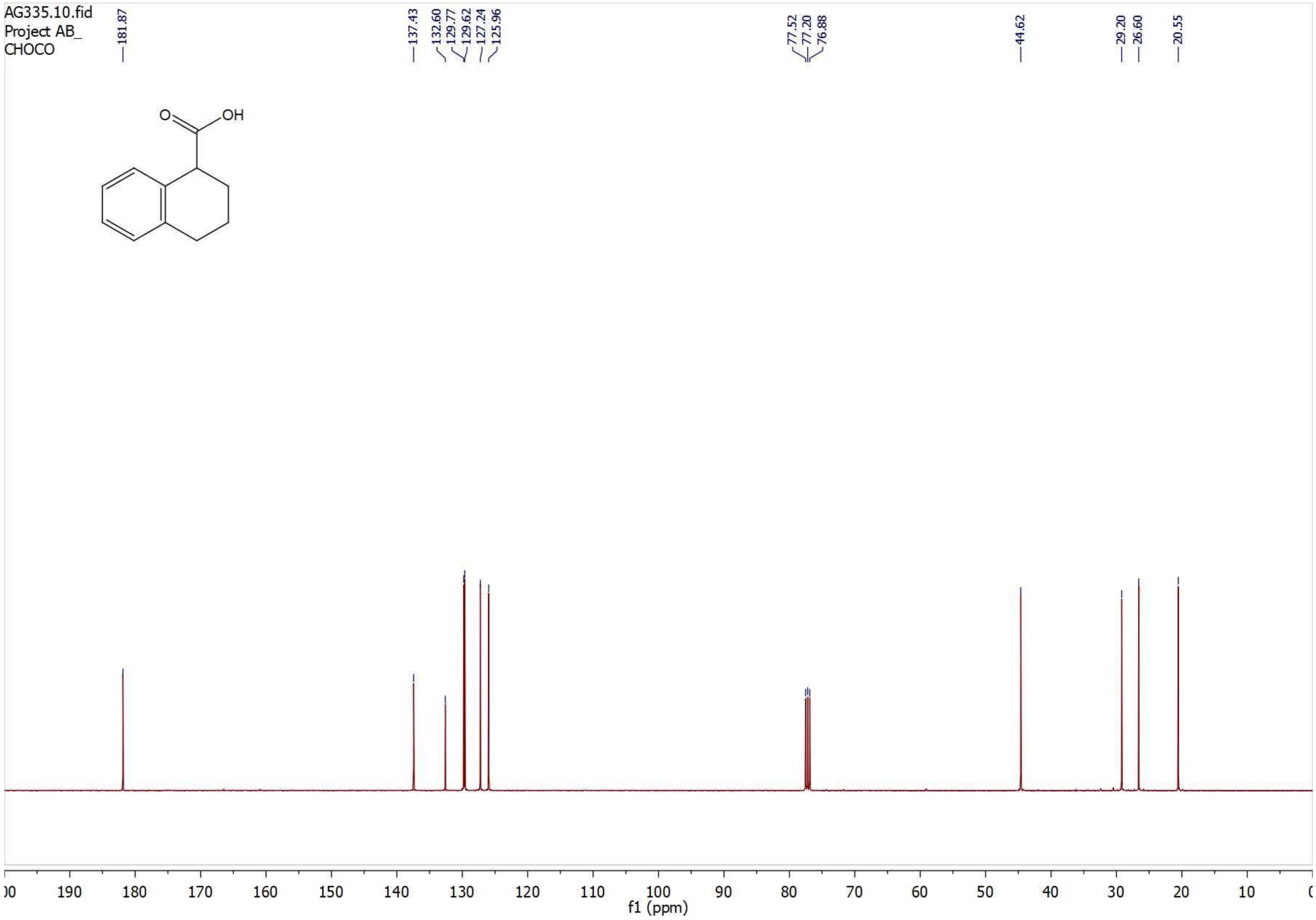

AG335.10.fid
Project AB_
CHOCO
181.87
137.43
132.60
129.77
129.62
127.24
125.96
77.52
77.20
76.88
44.62
29.20
26.60
20.55
OH
O
190
180
170
160
150
140
130
120
110
100
90
80
70
60
50
40
30
20
10
f1 (ppm)

Compound 4d

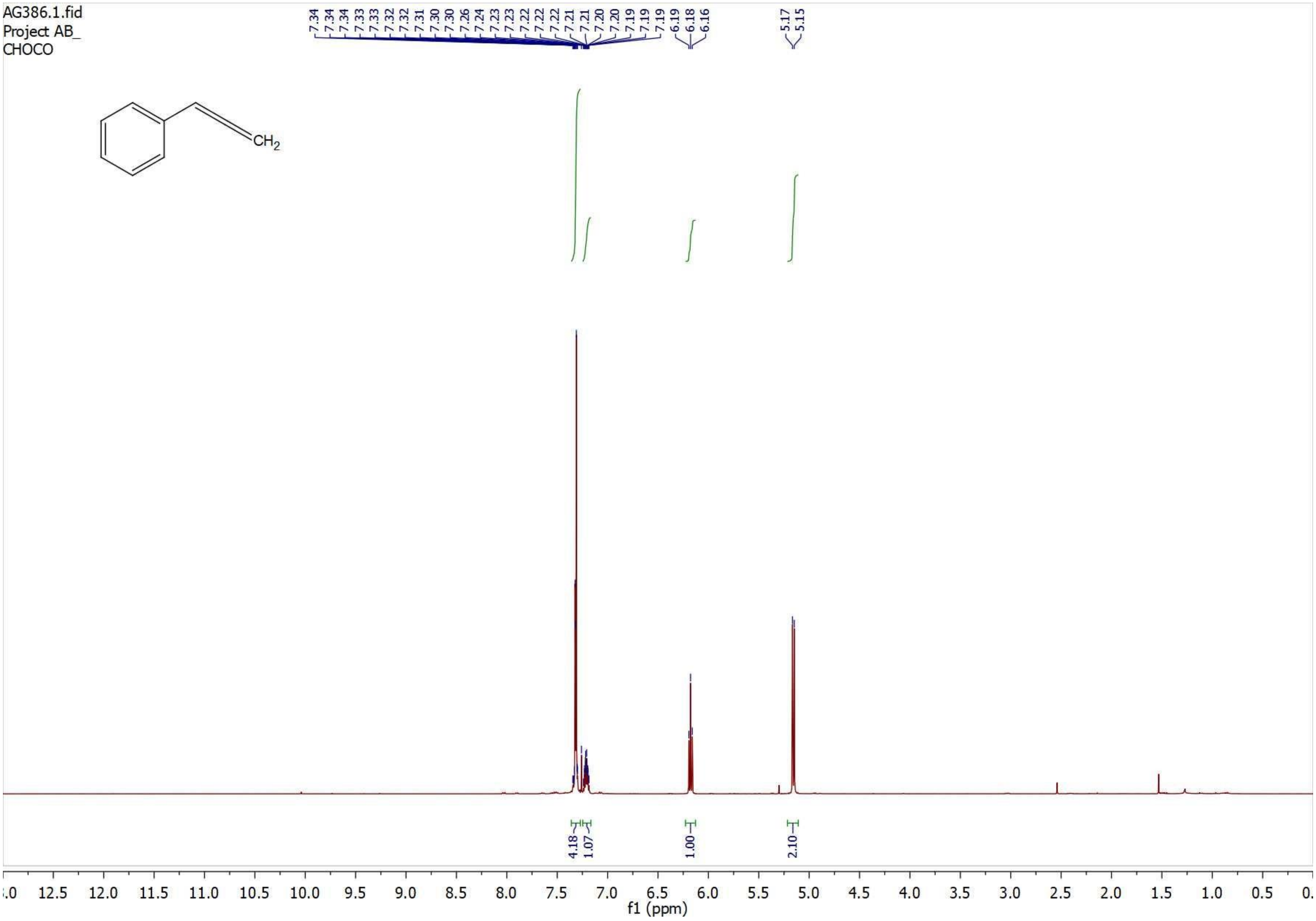
AG386.1.fid
Project AB_
CHOCO
7.34
7.34
7.34
7.33
7.33
7.32
7.32
7.31
7.30
7.30
7.26
7.24
7.23
7.23
7.22
7.22
7.22
7.21
7.21
7.20
7.20
7.19
7.19
7.19
6.19
6.18
6.16
5.17
5.15
CH_2
4.18
1.07
1.00
2.10
f1 (ppm)

Compound 4d

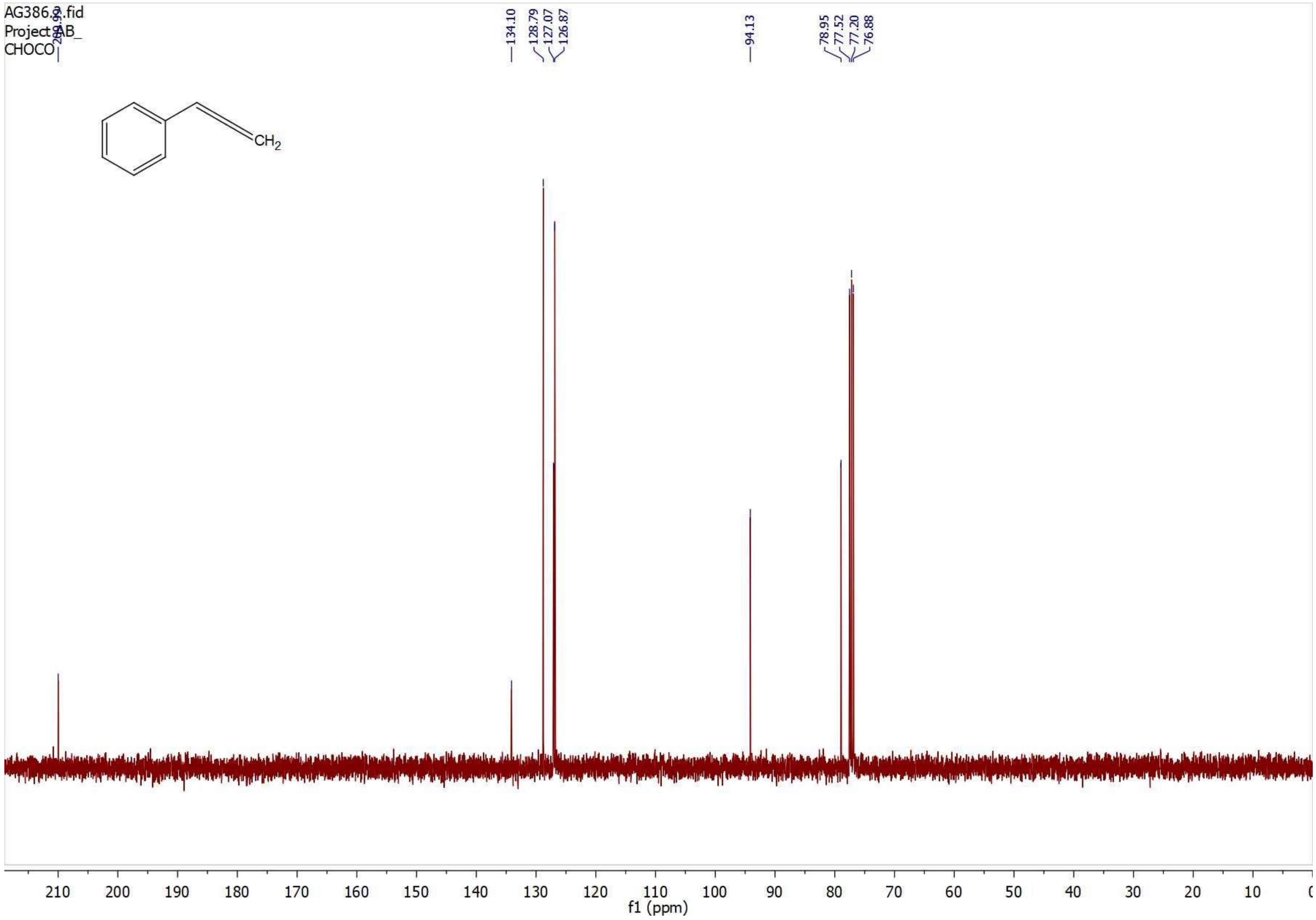
Project AB_
CHOCO
209.99
134.10
128.79
127.07
126.87
94.13
78.95
77.52
77.20
76.88
CH2
210 200 190 180 170 160 150 140 130 120 110 100 90 80 70 60 50 40 30 20 10
f1 (ppm)

Compound 4e

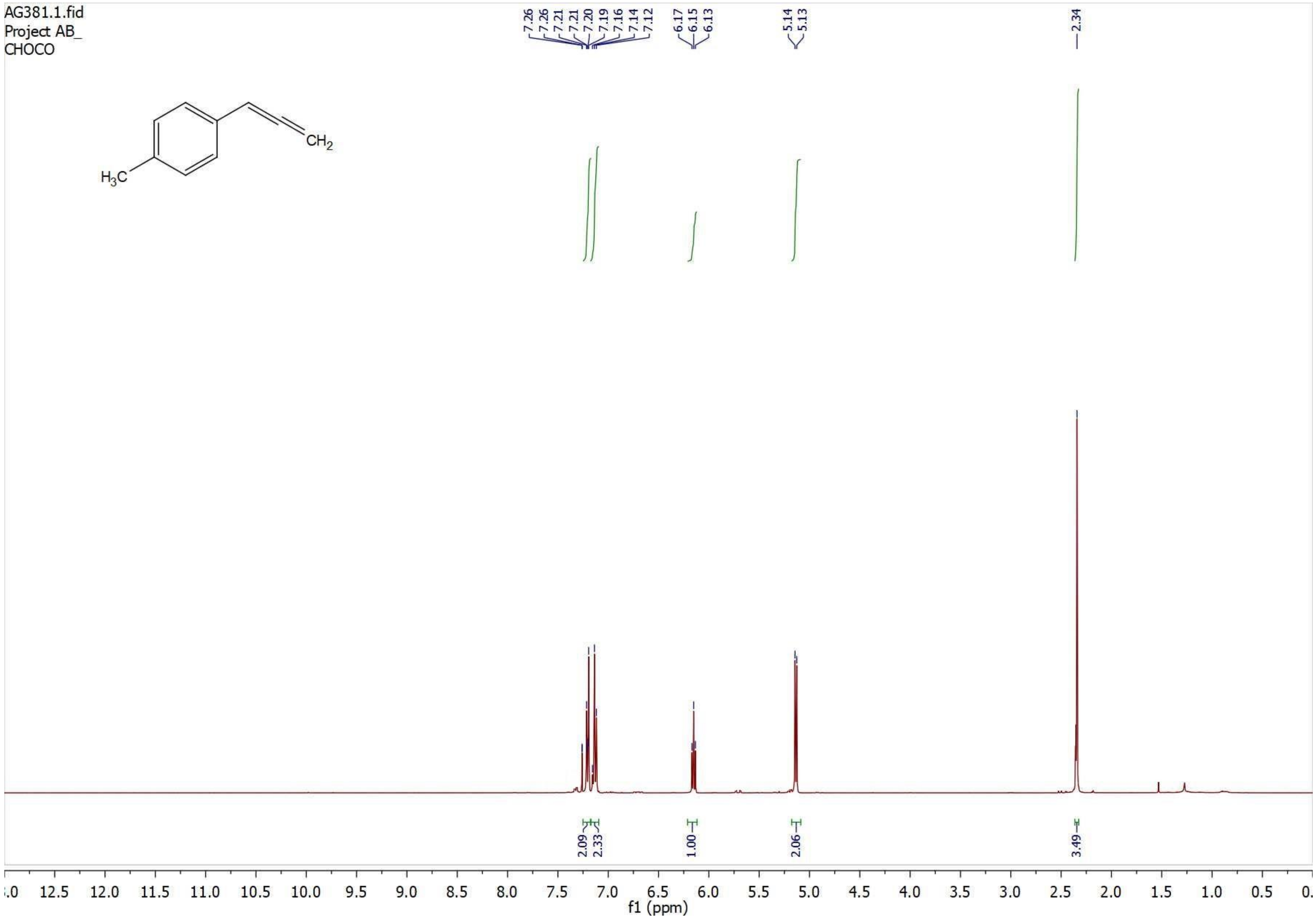
AG381.1.fid
Project AB_
CHOCO
CH2
H3C
7.26
7.26
7.21
7.21
7.20
7.19
7.16
7.14
7.12
6.17
6.15
6.13
5.14
5.13
2.34
2.09
2.33
1.00
2.06
3.49
12.5 12.0 11.5 11.0 10.5 10.0 9.5 9.0 8.5 8.0 7.5 7.0 6.5 6.0 5.5 5.0 4.5 4.0 3.5 3.0 2.5 2.0 1.5 1.0 0.5
f1 (ppm)

Compound 4e

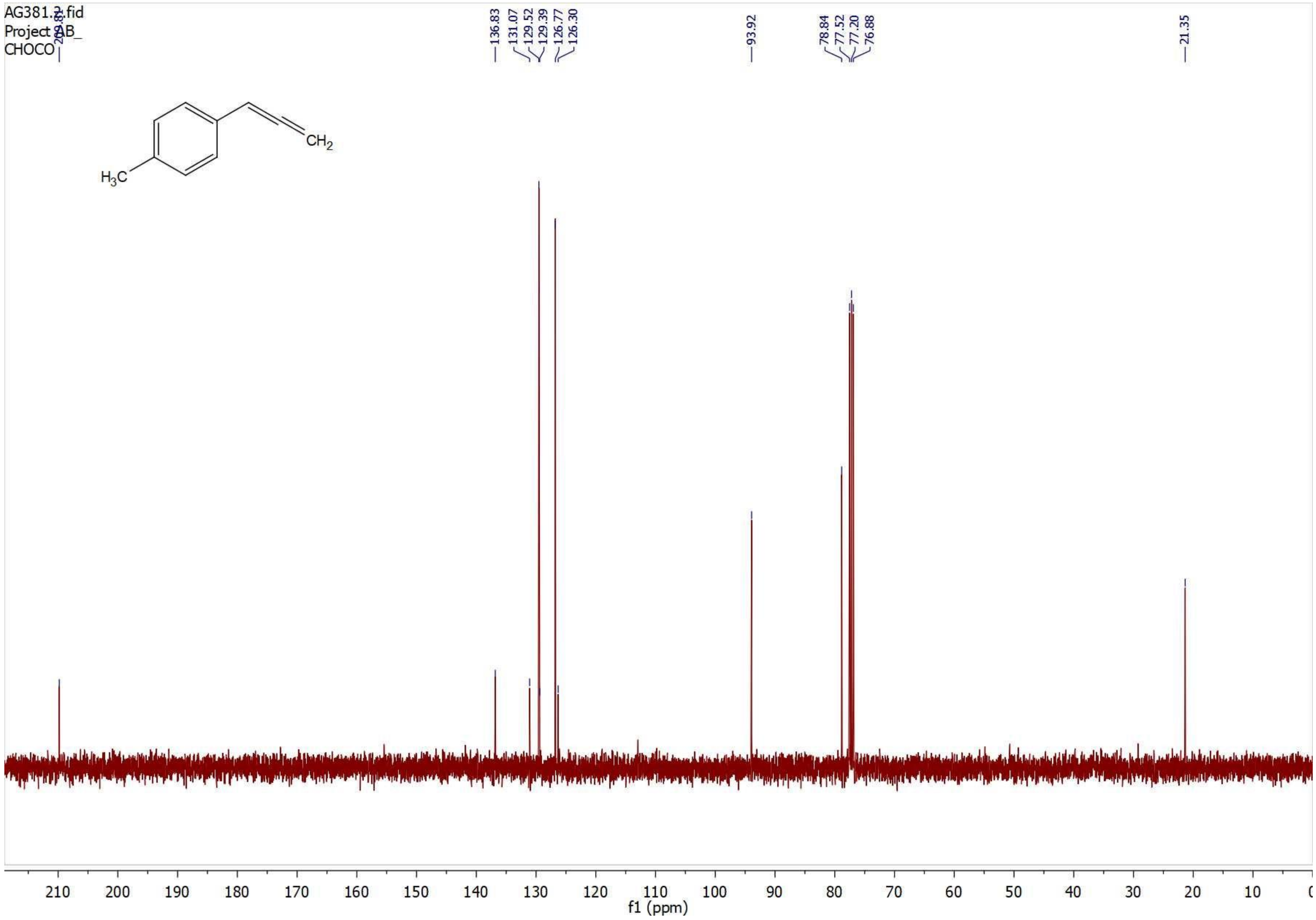
AG381.2.fid
Project AB_
CHOCO
209.81
136.83
131.07
129.52
129.39
126.77
126.30
93.92
78.84
77.52
77.20
76.88
21.35
CH2
H3C
210
200
190
180
170
160
150
140
130
120
110
100
90
80
70
60
50
40
30
20
10
f1 (ppm)

Compound 4f

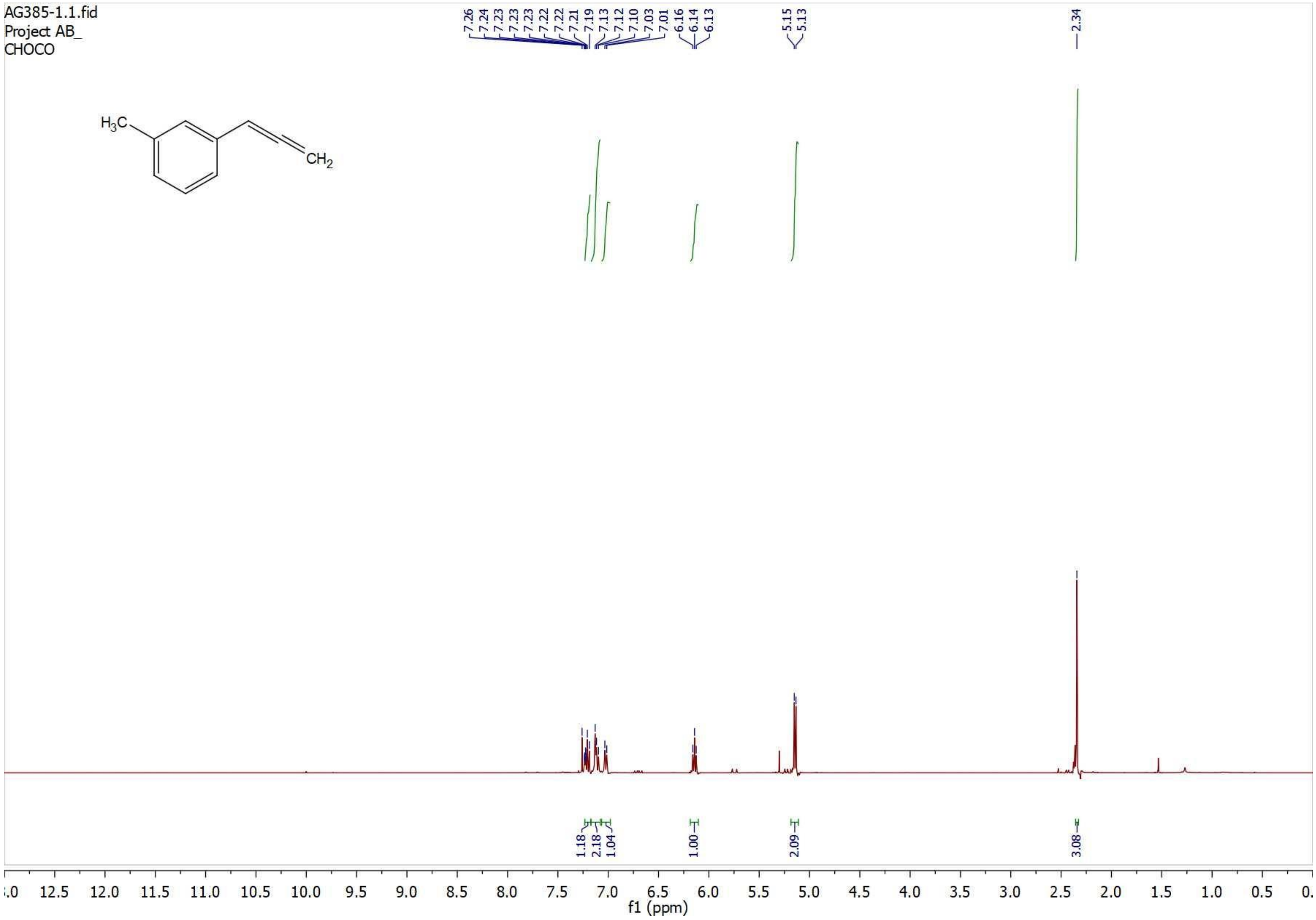
AG385-1.1.fid
Project AB_
CHOCO
H3C
CH2
7.26
7.24
7.23
7.23
7.23
7.22
7.22
7.21
7.19
7.13
7.12
7.10
7.03
7.01
6.16
6.14
6.13
5.15
5.13
2.34
1.18
2.18
1.04
1.00
2.09
3.08
f1 (ppm)

Compound 4f

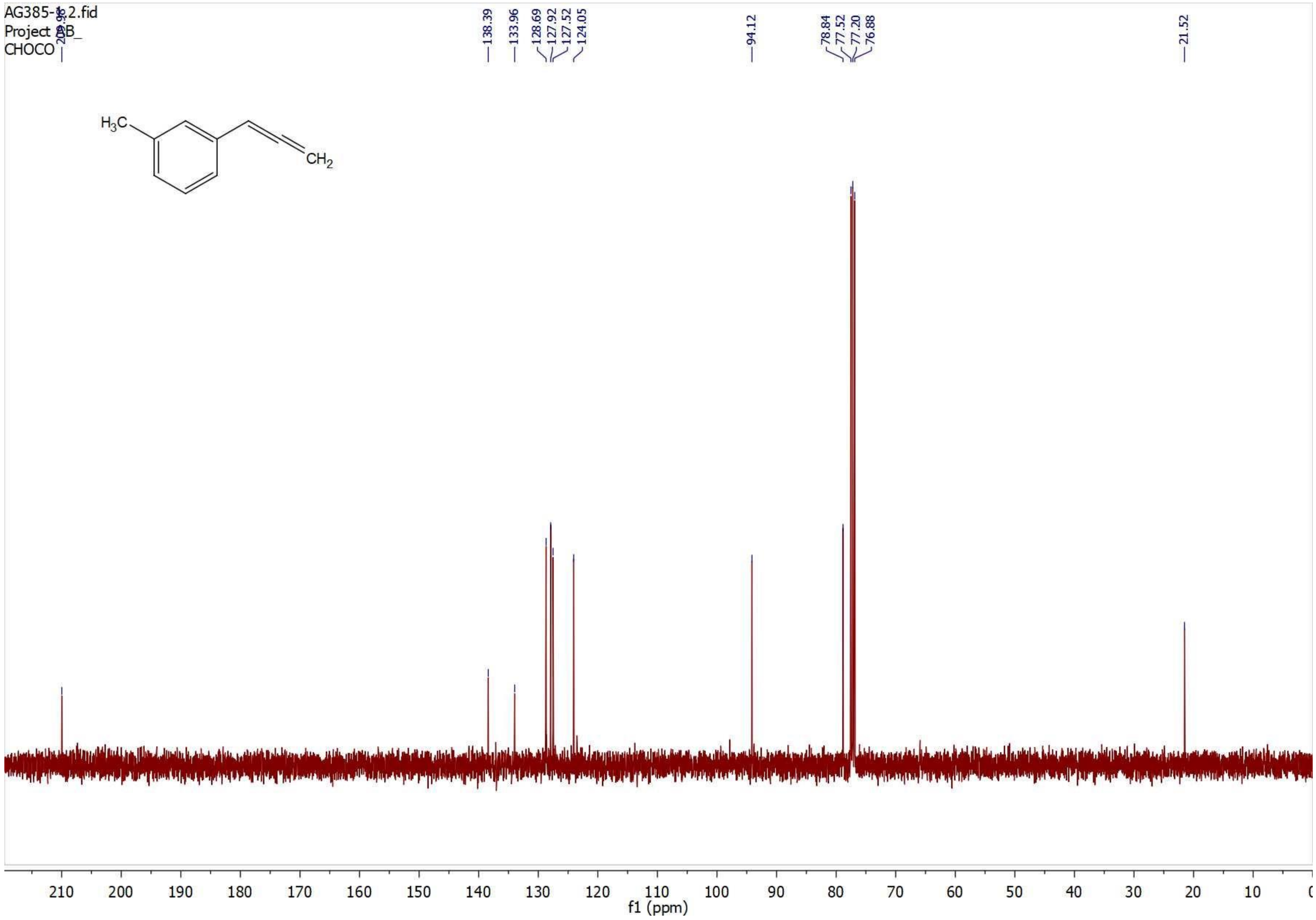
AG385-1p2.fid
Project AB_
CHOCO
209.95
138.39
133.96
128.69
127.92
127.52
124.05
94.12
78.84
77.52
77.20
76.88
21.52
H3C
CH2
210
200
190
180
170
160
150
140
130
120
110
100
90
80
70
60
50
40
30
20
10
f1 (ppm)

Compound 5a

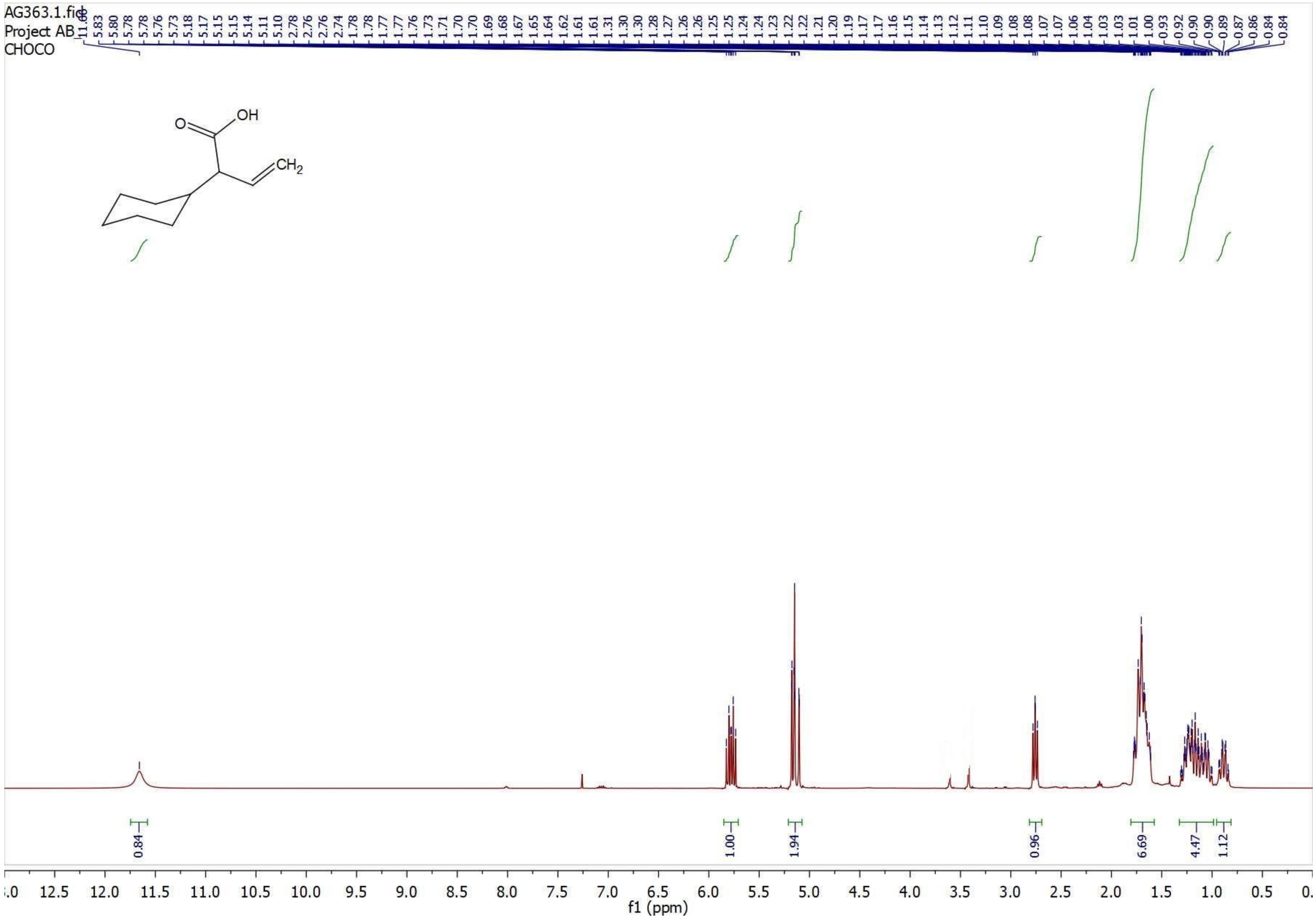

Compound 5a

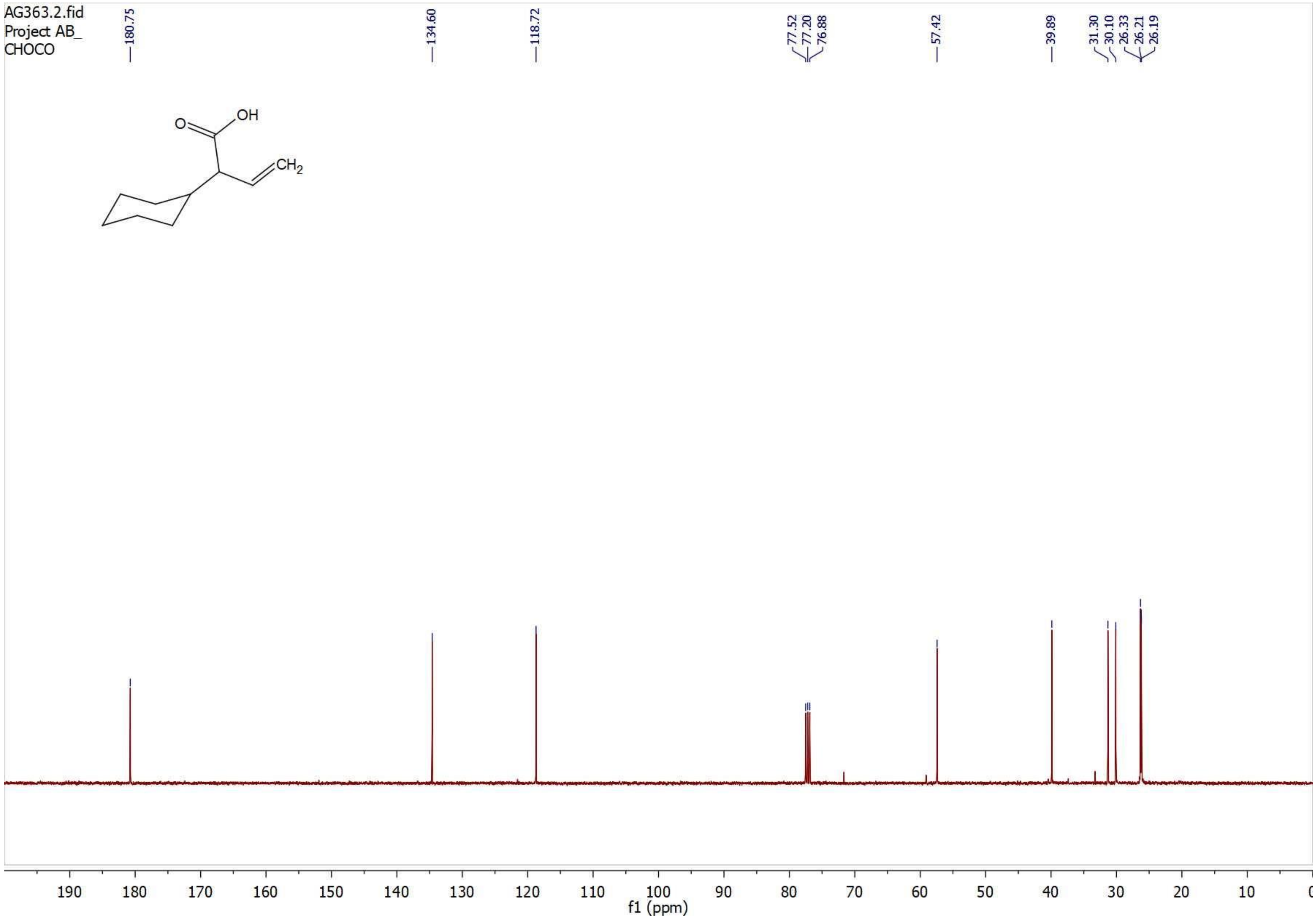
AG363.2.fid
Project AB_
CHOCO
180.75
134.60
118.72
77.52
77.20
76.88
57.42
39.89
31.30
30.10
26.33
26.21
26.19
OH
O
CH2
190 180 170 160 150 140 130 120 110 100 90 80 70 60 50 40 30 20 10
f1 (ppm)

Compound 5b

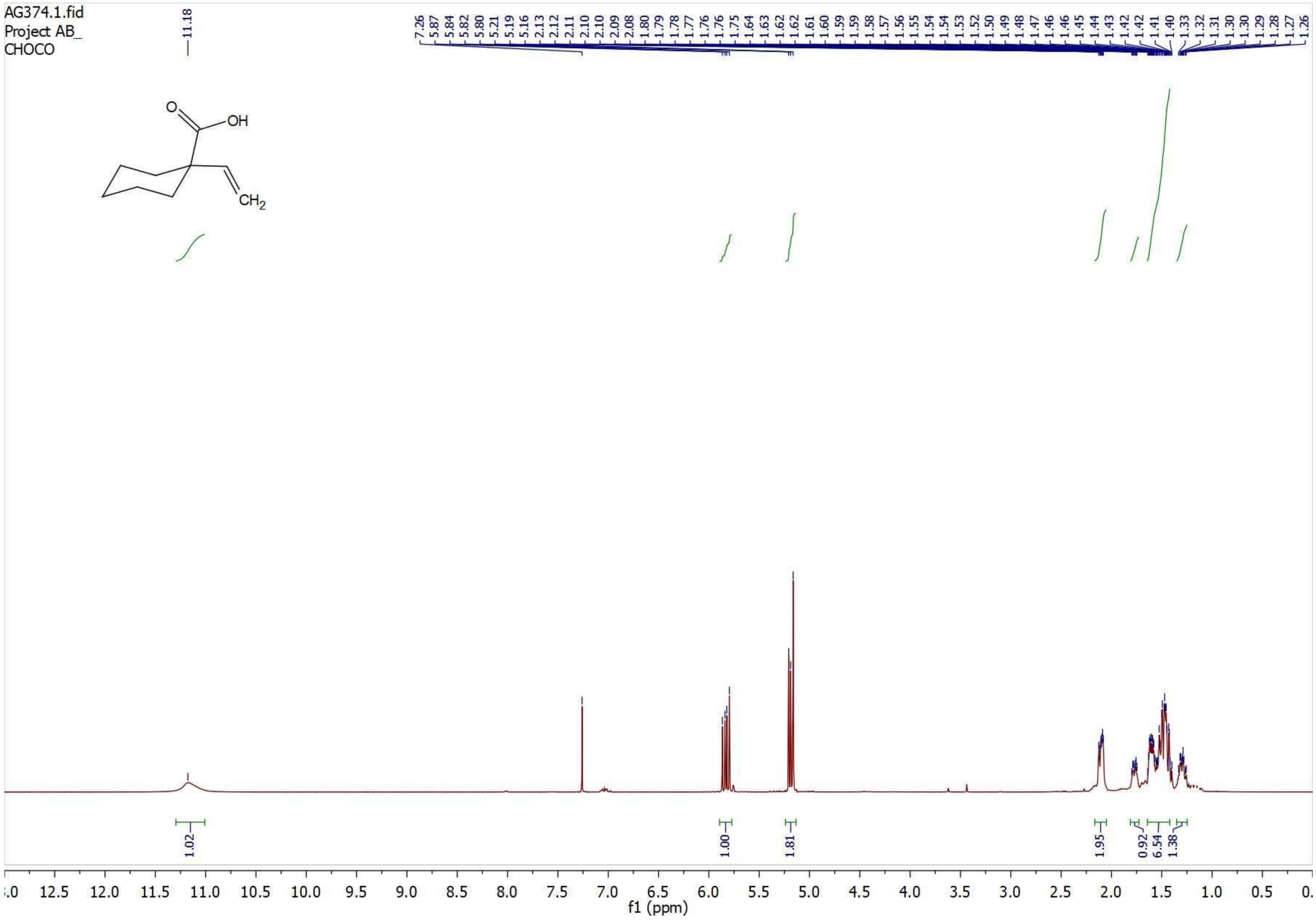

AG374.1.fid
Project AB_
CHOCO
11.18
7.26
5.87
5.84
5.82
5.80
5.21
5.19
5.16
2.13
2.12
2.11
2.10
2.10
2.09
2.08
1.80
1.79
1.78
1.77
1.76
1.76
1.75
1.64
1.63
1.62
1.62
1.61
1.60
1.59
1.59
1.58
1.57
1.56
1.55
1.54
1.54
1.53
1.52
1.50
1.49
1.48
1.47
1.46
1.46
1.45
1.44
1.43
1.42
1.42
1.41
1.40
1.33
1.32
1.31
1.30
1.30
1.29
1.28
1.27
1.26
OH
CH2
1.02
1.00
1.81
1.95
0.92
6.54
1.38
f1 (ppm)

Compound 5b

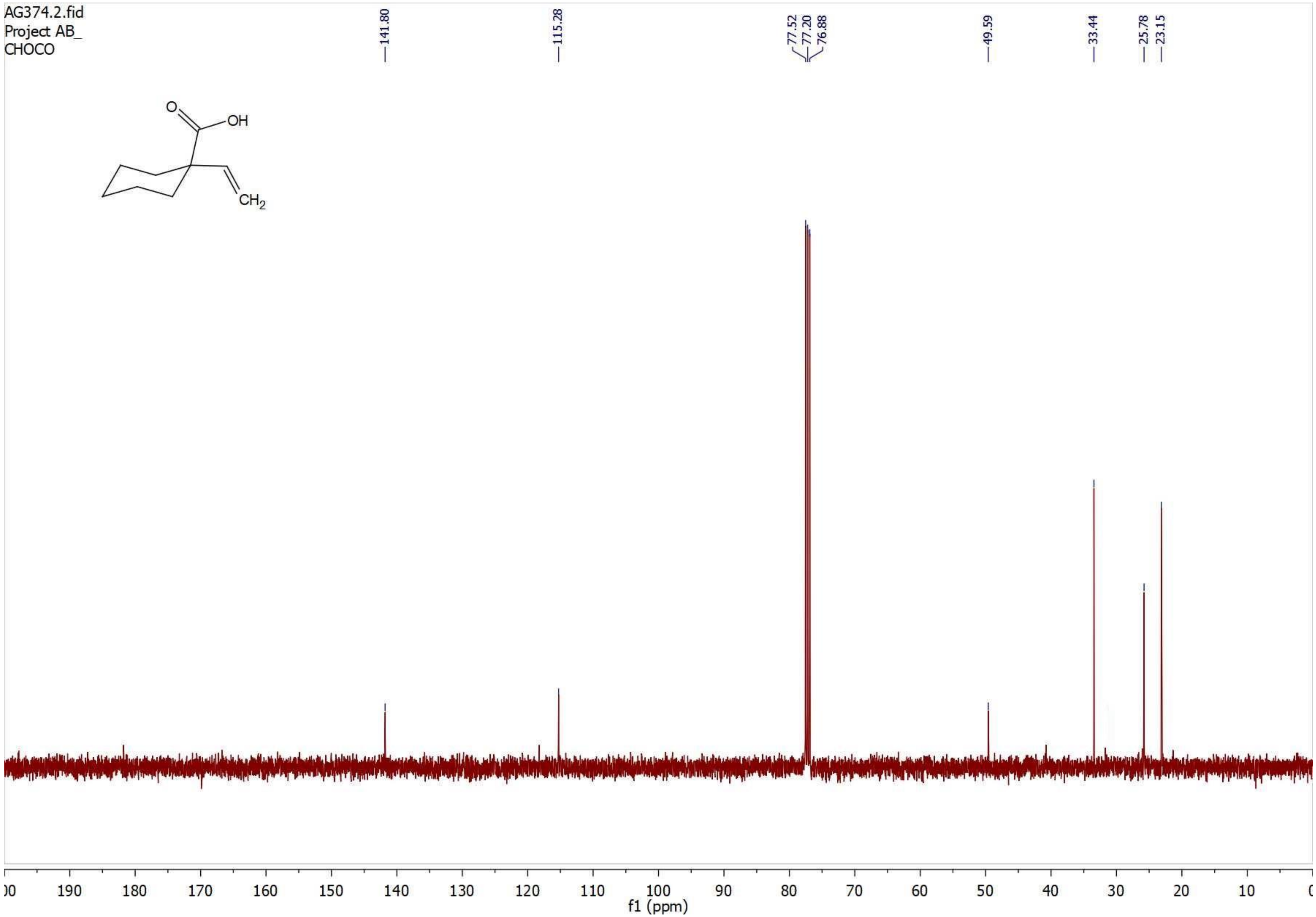
AG374.2.fid
Project AB_
CHOCO
141.80
115.28
77.52
77.20
76.88
49.59
33.44
25.78
23.15
O
OH
CH2
190
180
170
160
150
140
130
120
110
100
90
80
70
60
50
40
30
20
10
f1 (ppm)

Compound 5c

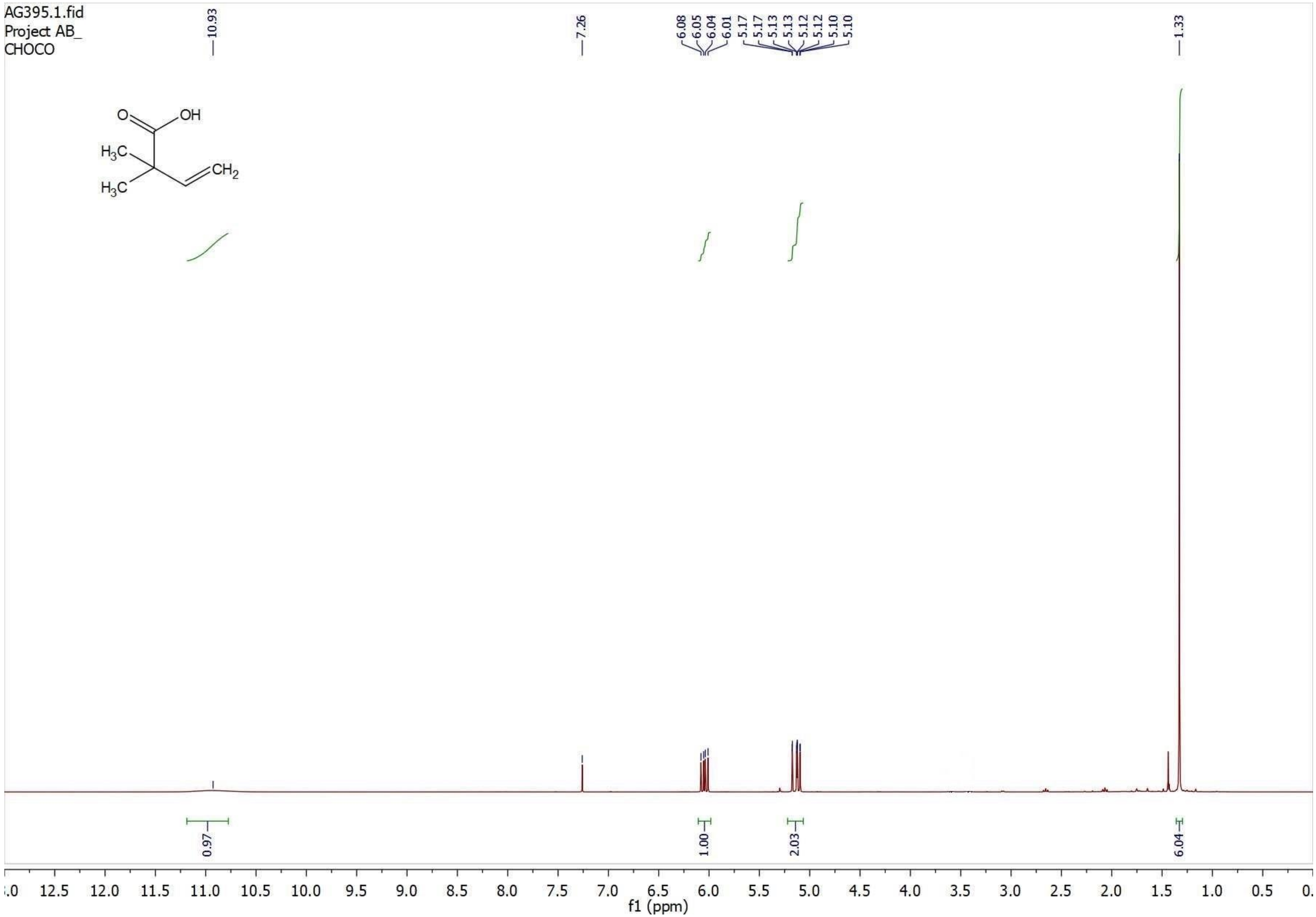
AG395.1.fid
Project AB_
CHOCO
10.93
7.26
6.08
6.05
6.04
6.01
5.17
5.17
5.13
5.13
5.12
5.12
5.10
5.10
1.33
0.97
1.00
2.03
6.04
f1 (ppm)

Compound 5c

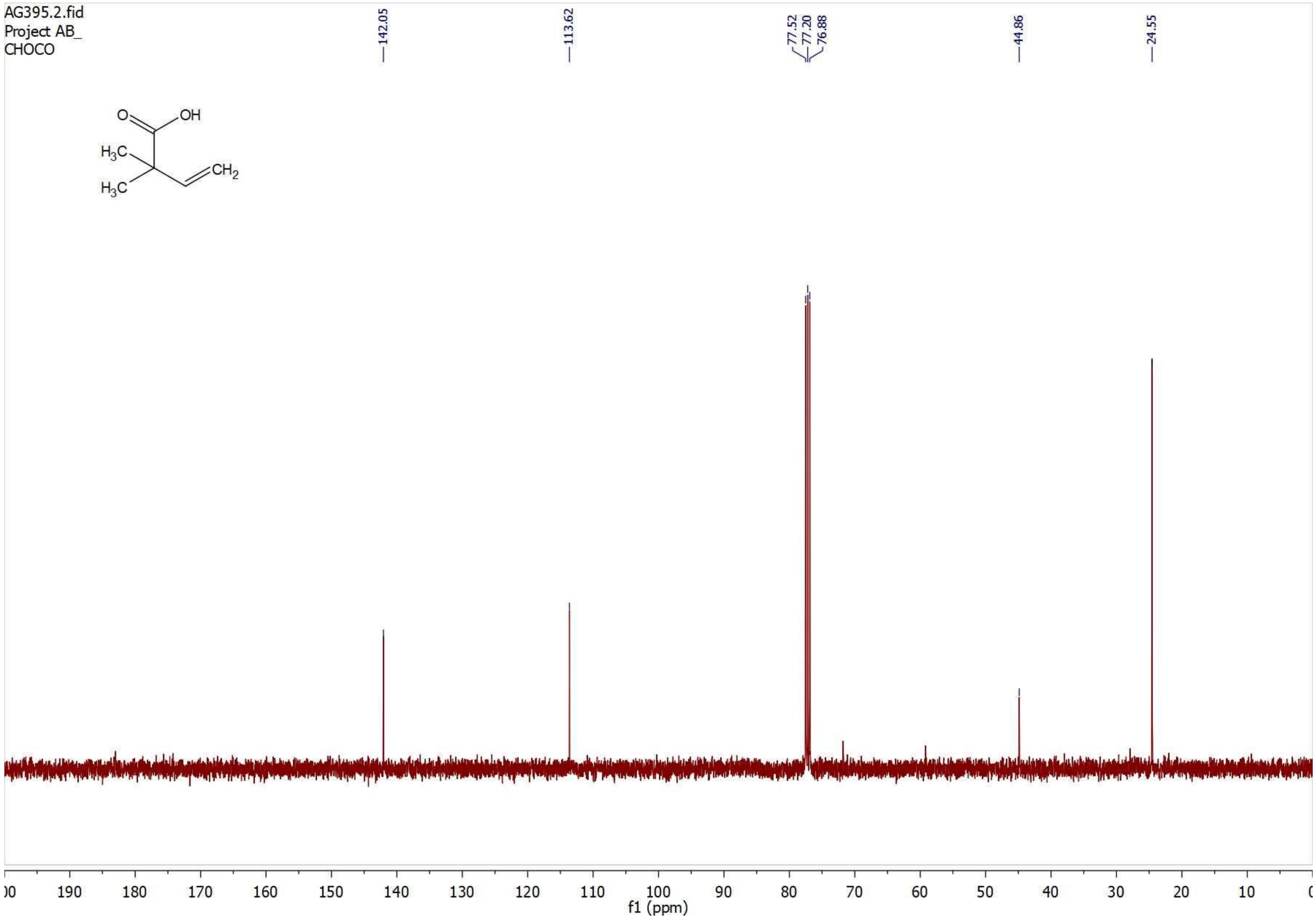
AG395.2.fid
Project AB_
CHOCO
142.05
113.62
77.52
77.20
76.88
44.86
24.55
O
OH
H_3C
H_3C
CH_2
f1 (ppm)

Compound 5da/5db/5dc

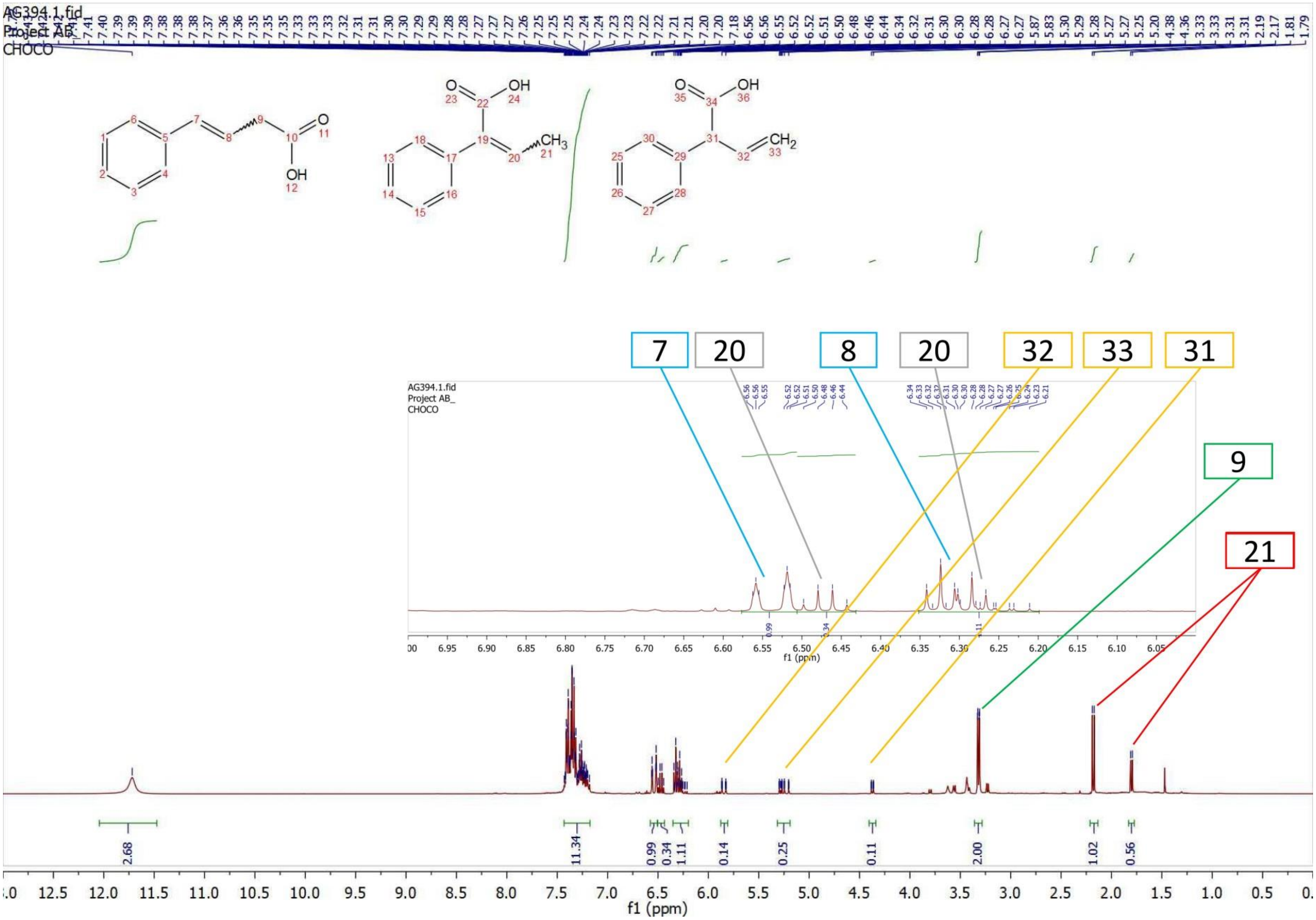

Compound 5da/5db/5dc

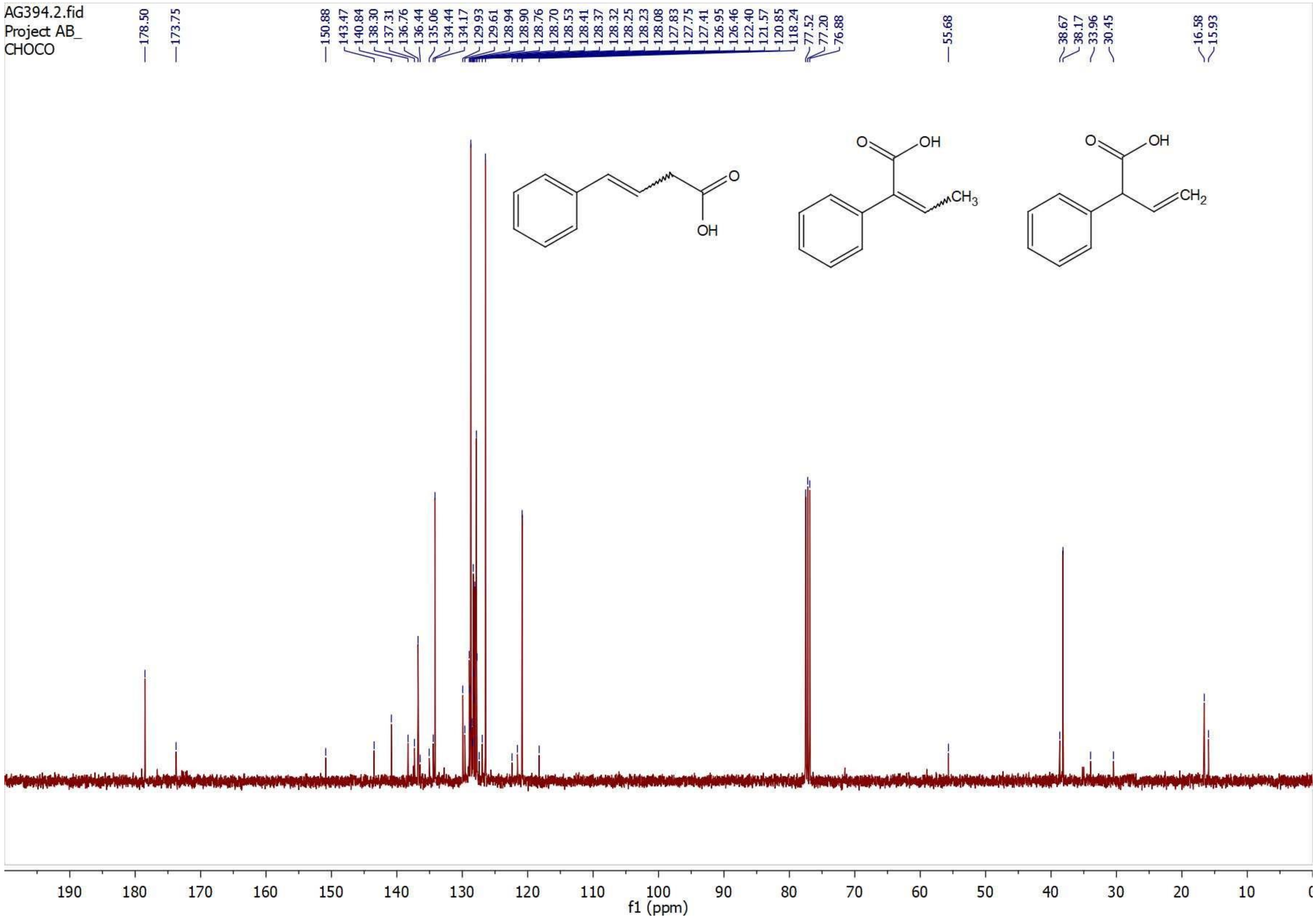

Chromatogram of compound 5da/5db/5dc

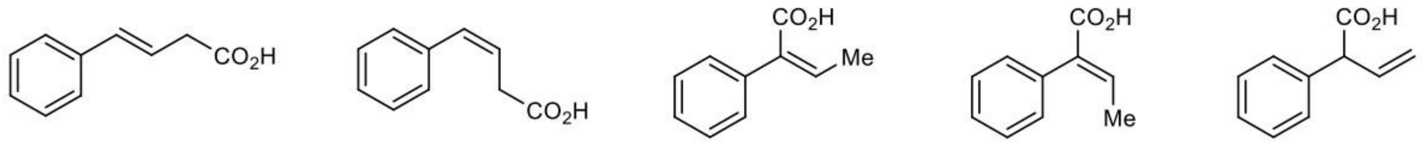

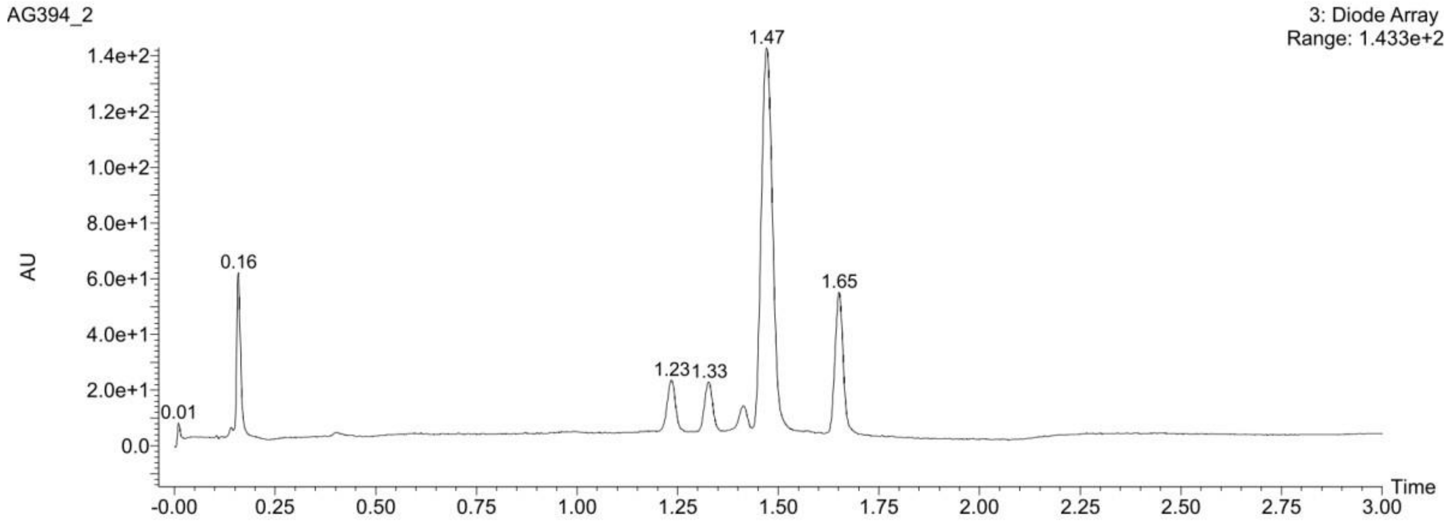

diol column
29-Apr-2019
17:28:57
UPC2

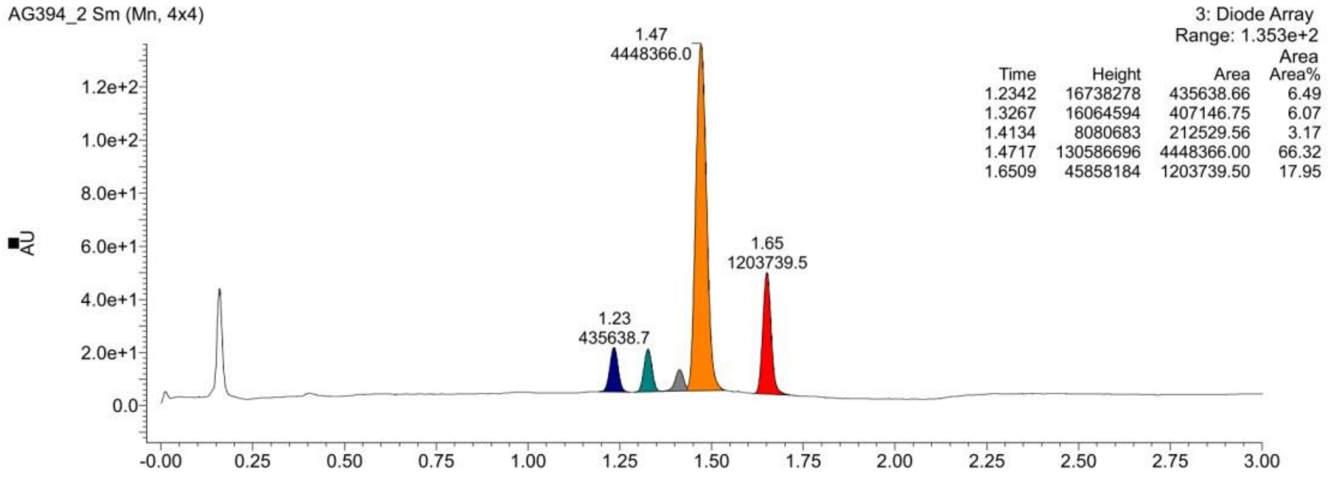

Compound 5ea/5eb/5ec

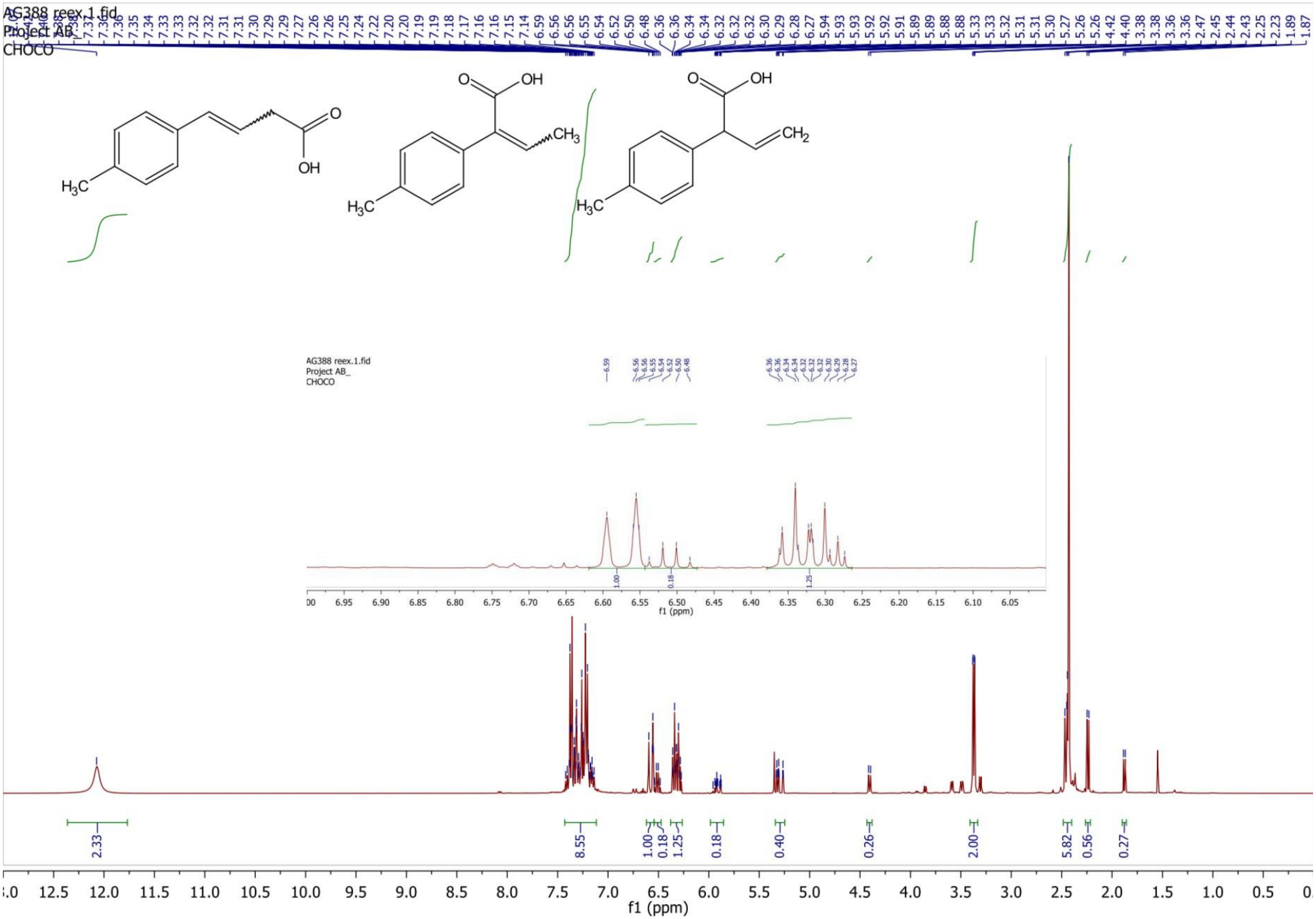

Compound 5ea/5eb/5ec

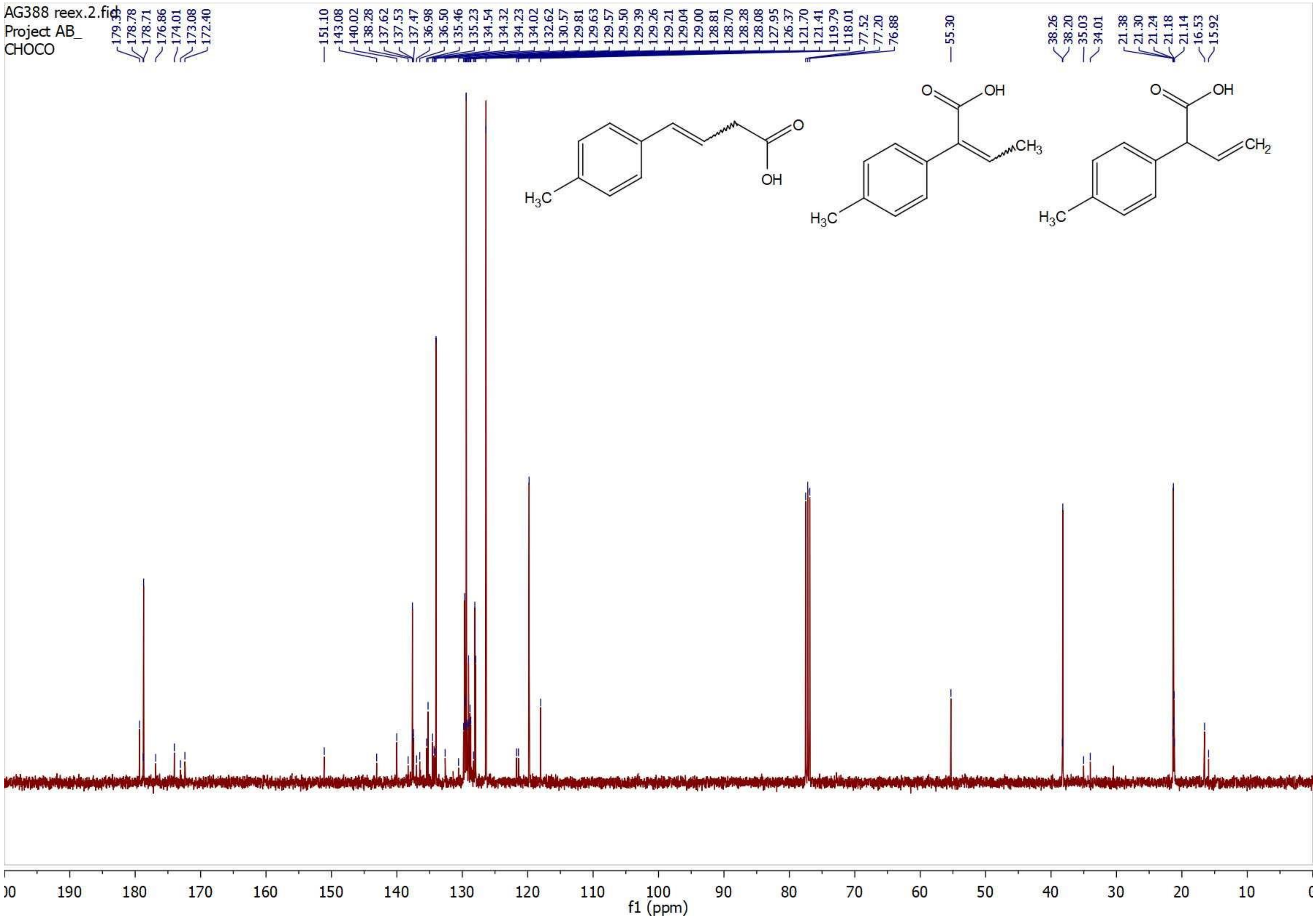

Chromatogram of compound 5ea/5eb/5ec

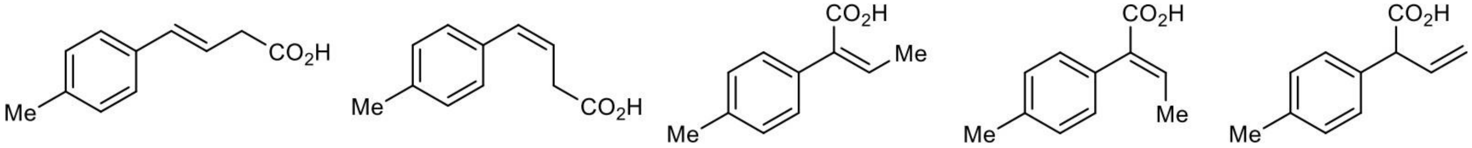

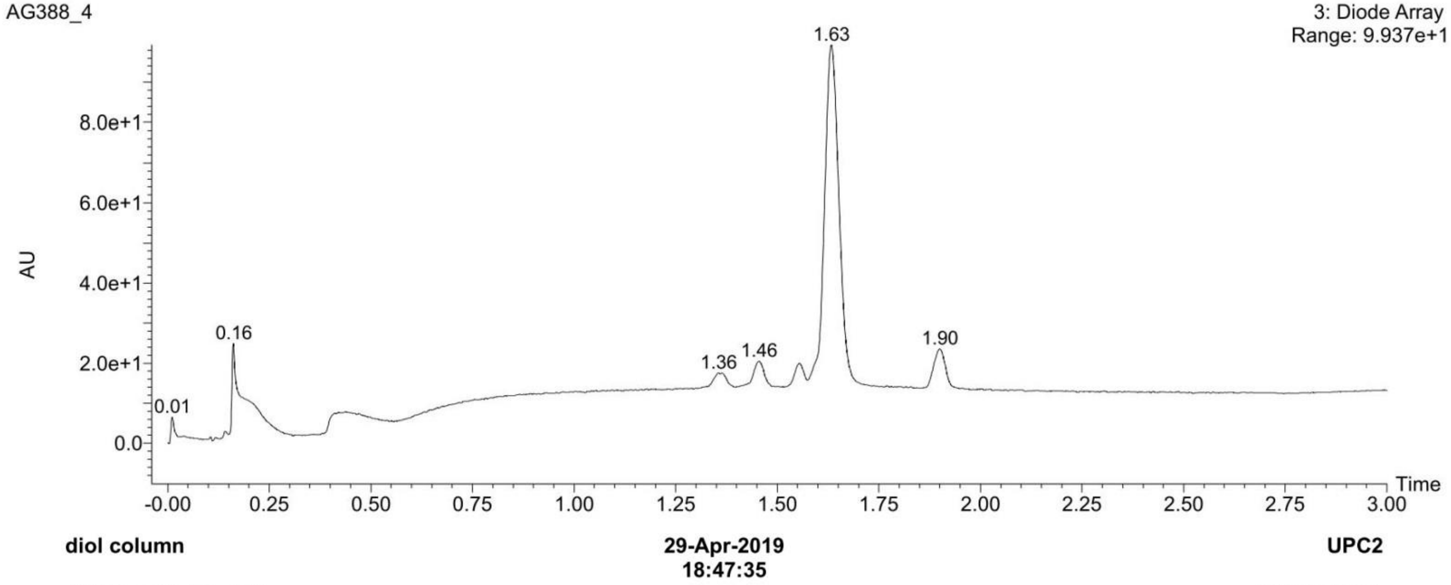

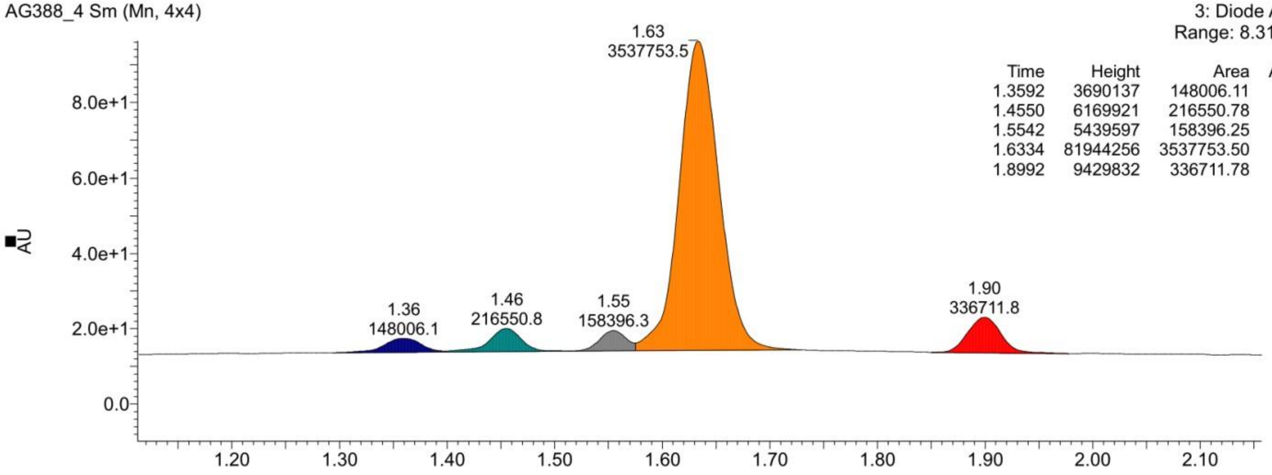

Time	Height	Area	Area Area%
1.3592	3690137	148006.11	3.37
1.4550	6169921	216550.78	4.92
1.5542	5439597	158396.25	3.60
1.6334	81944256	3537753.50	80.45
1.8992	9429832	336711.78	7.66

Compound 5fa/5fb/5fc

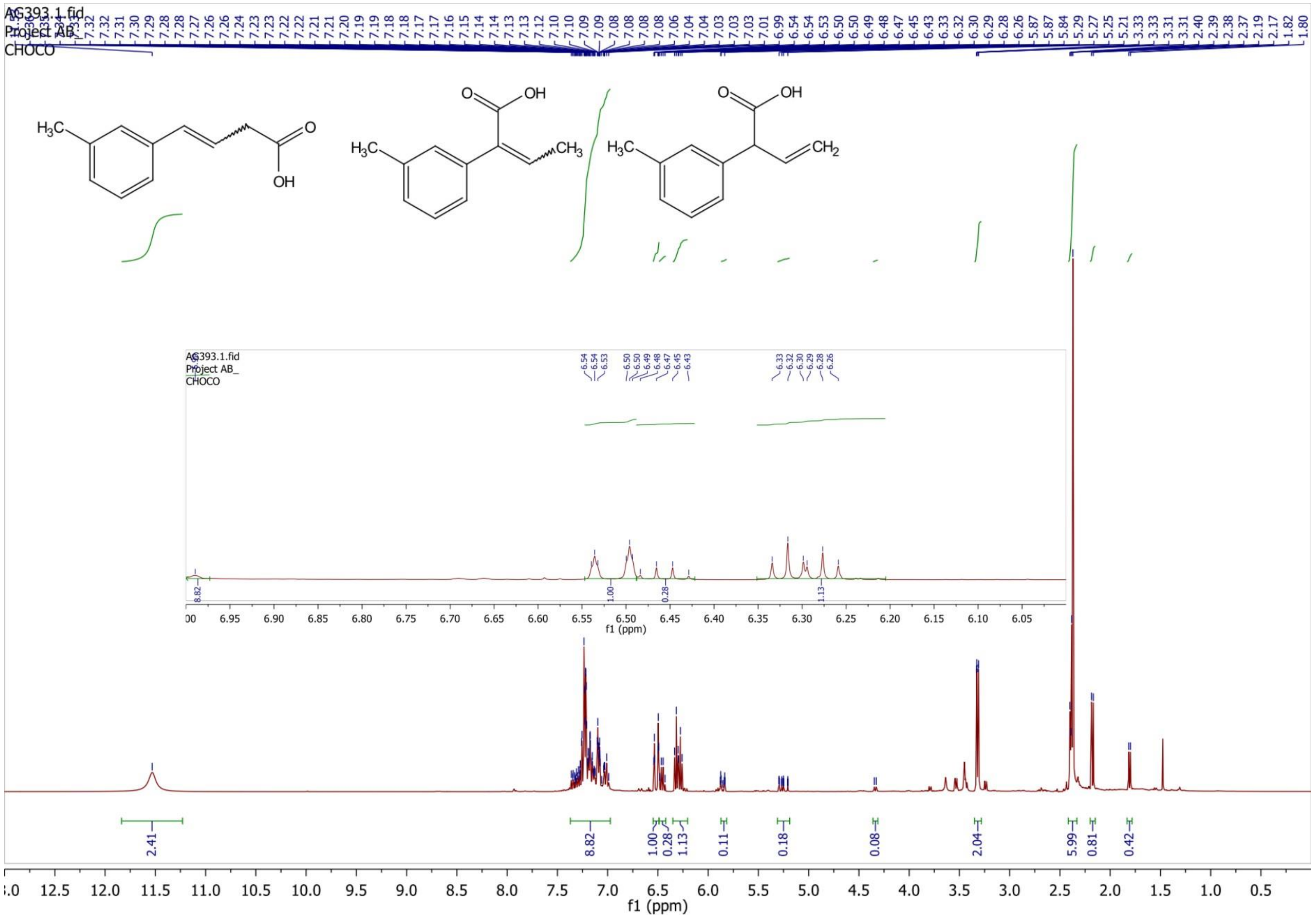

Compound 5fa/5fb/5fc

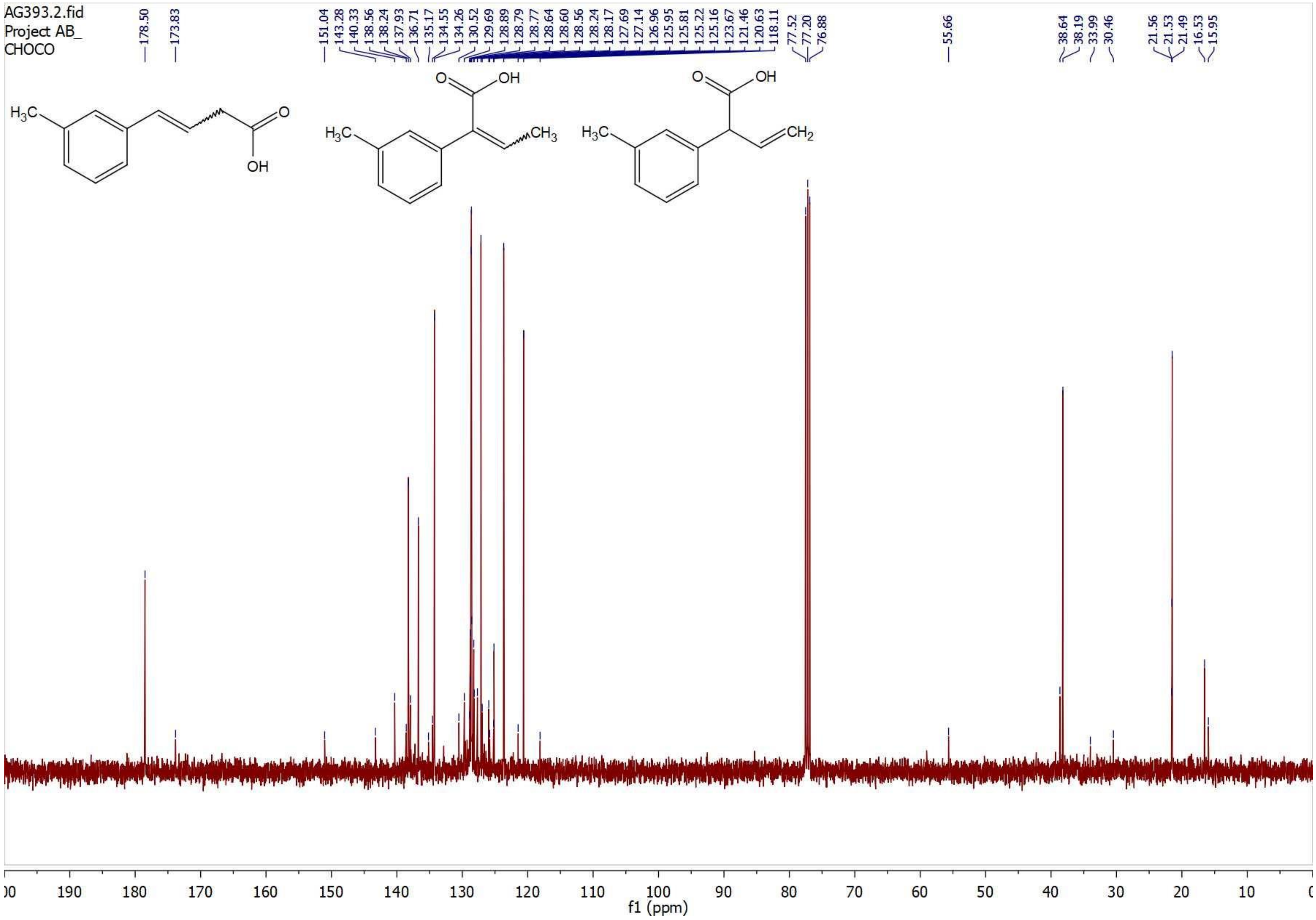

Chromatogram of compound 5fa/5fb/5fc

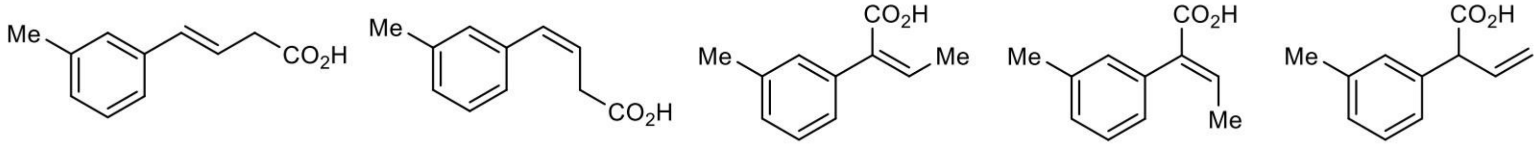

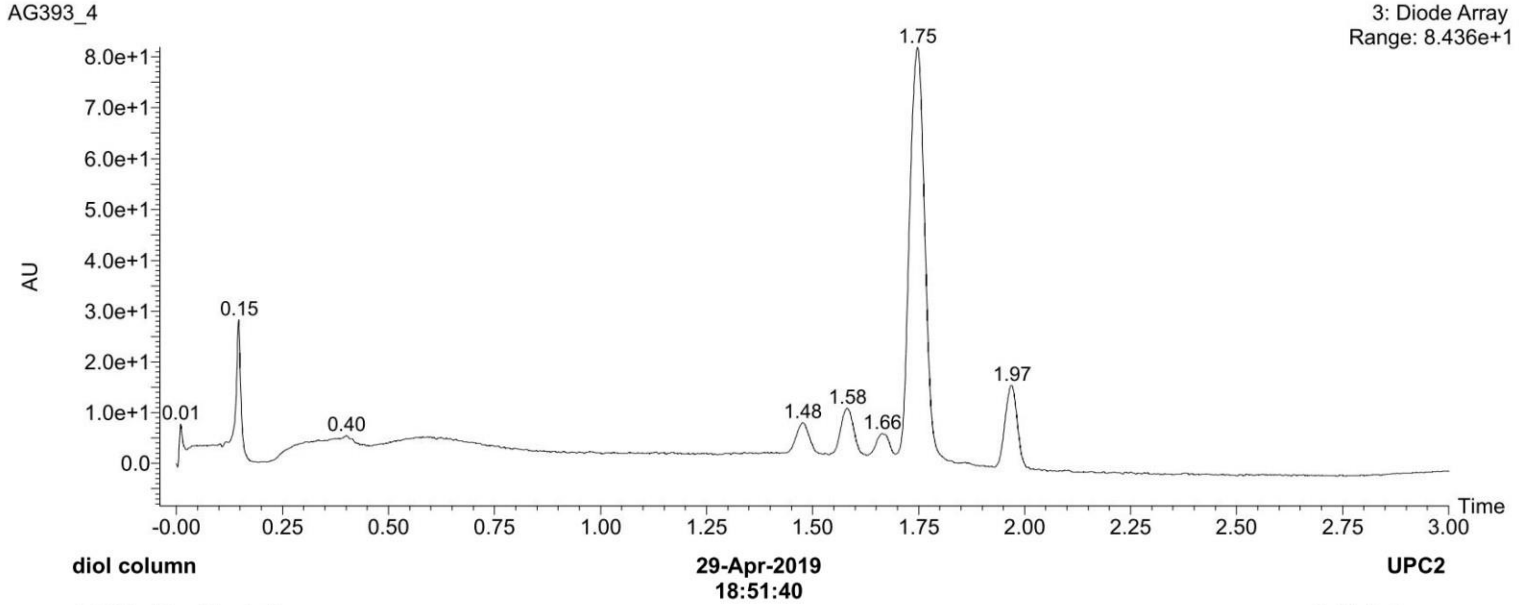

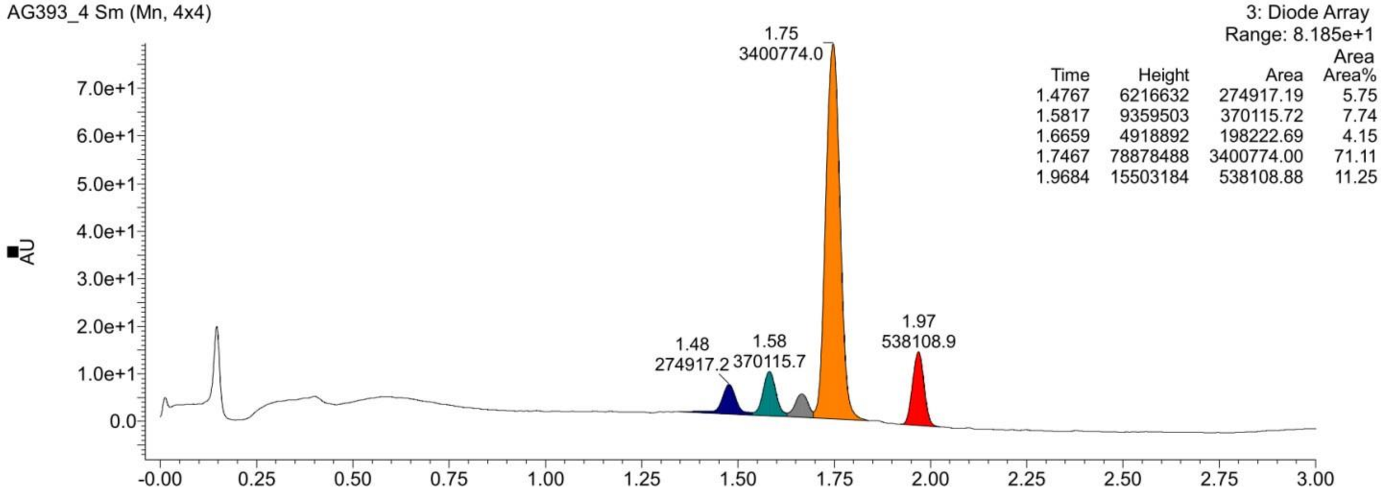

Time	Height	Area	Area Area%
1.4767	6216632	274917.19	5.75
1.5817	9359503	370115.72	7.74
1.6659	4918892	198222.69	4.15
1.7467	78878488	3400774.00	71.11
1.9684	15503184	538108.88	11.25

Compound 6a

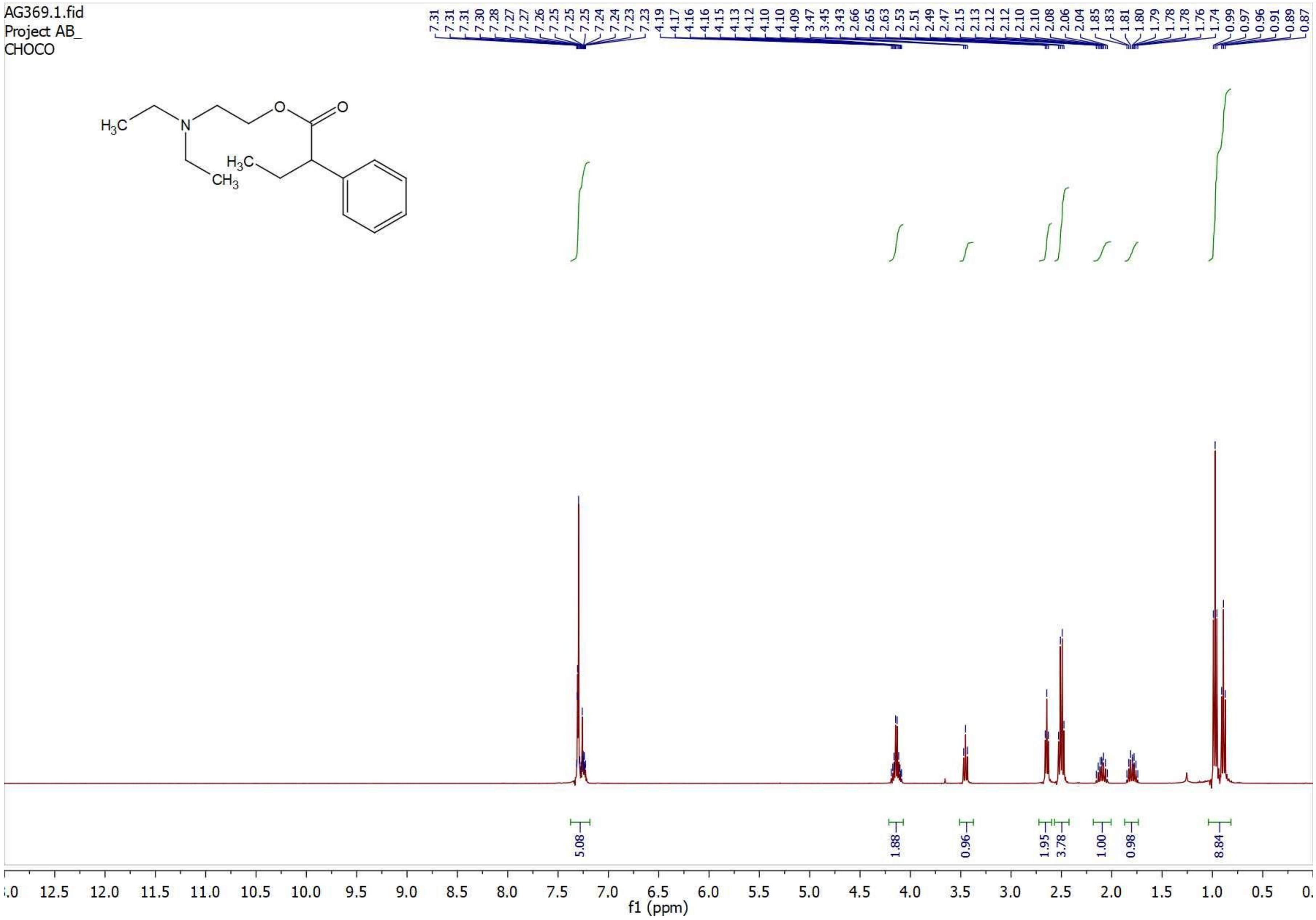
AG369.1.fid
Project AB_
CHOCO
7.31 7.31 7.31 7.30 7.28 7.27 7.27 7.26 7.25 7.25 7.25 7.24 7.24 7.23 7.23 4.19 4.17 4.16 4.16 4.15 4.13 4.12 4.10 4.10 4.09 3.47 3.45 3.43 2.66 2.65 2.63 2.53 2.51 2.49 2.47 2.15 2.13 2.12 2.12 2.10 2.10 2.08 2.06 2.04 1.85 1.83 1.81 1.80 1.79 1.78 1.78 1.76 1.74 0.99 0.97 0.96 0.91 0.89 0.87
5.08
1.88
0.96
1.95
3.78
1.00
0.98
8.84
13.0 12.5 12.0 11.5 11.0 10.5 10.0 9.5 9.0 8.5 8.0 7.5 7.0 6.5 6.0 5.5 5.0 4.5 4.0 3.5 3.0 2.5 2.0 1.5 1.0 0.5 0.
f1 (ppm)

Compound 6a

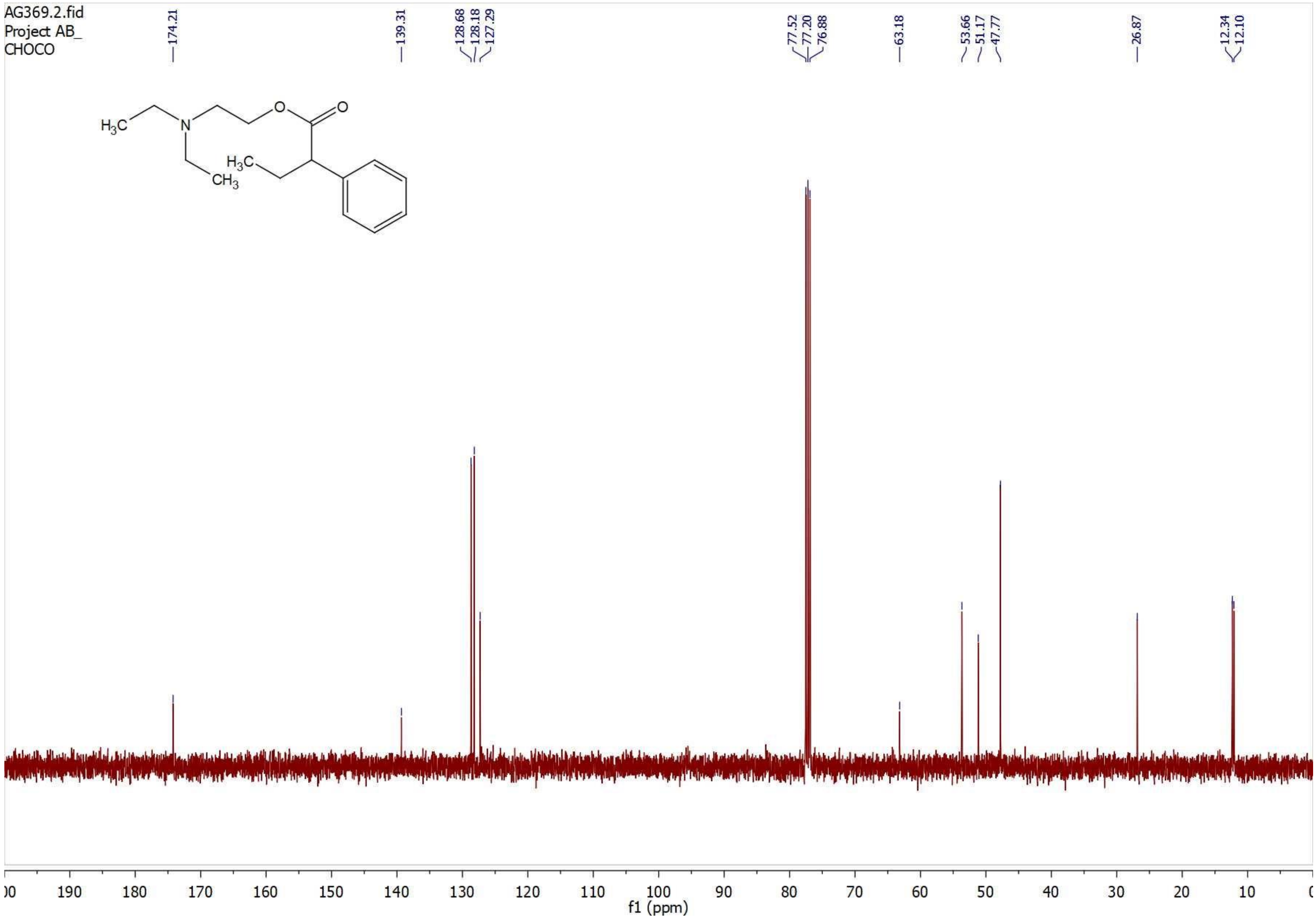
AG369.2.fid
Project AB_
CHOCO
174.21
139.31
128.68
128.18
127.29
77.52
77.20
76.88
63.18
53.66
51.17
47.77
26.87
12.34
12.10
H_3C
N
O
O
H_3C
CH_3
190
180
170
160
150
140
130
120
110
100
90
80
70
60
50
40
30
20
10
f1 (ppm)

Compound 6b

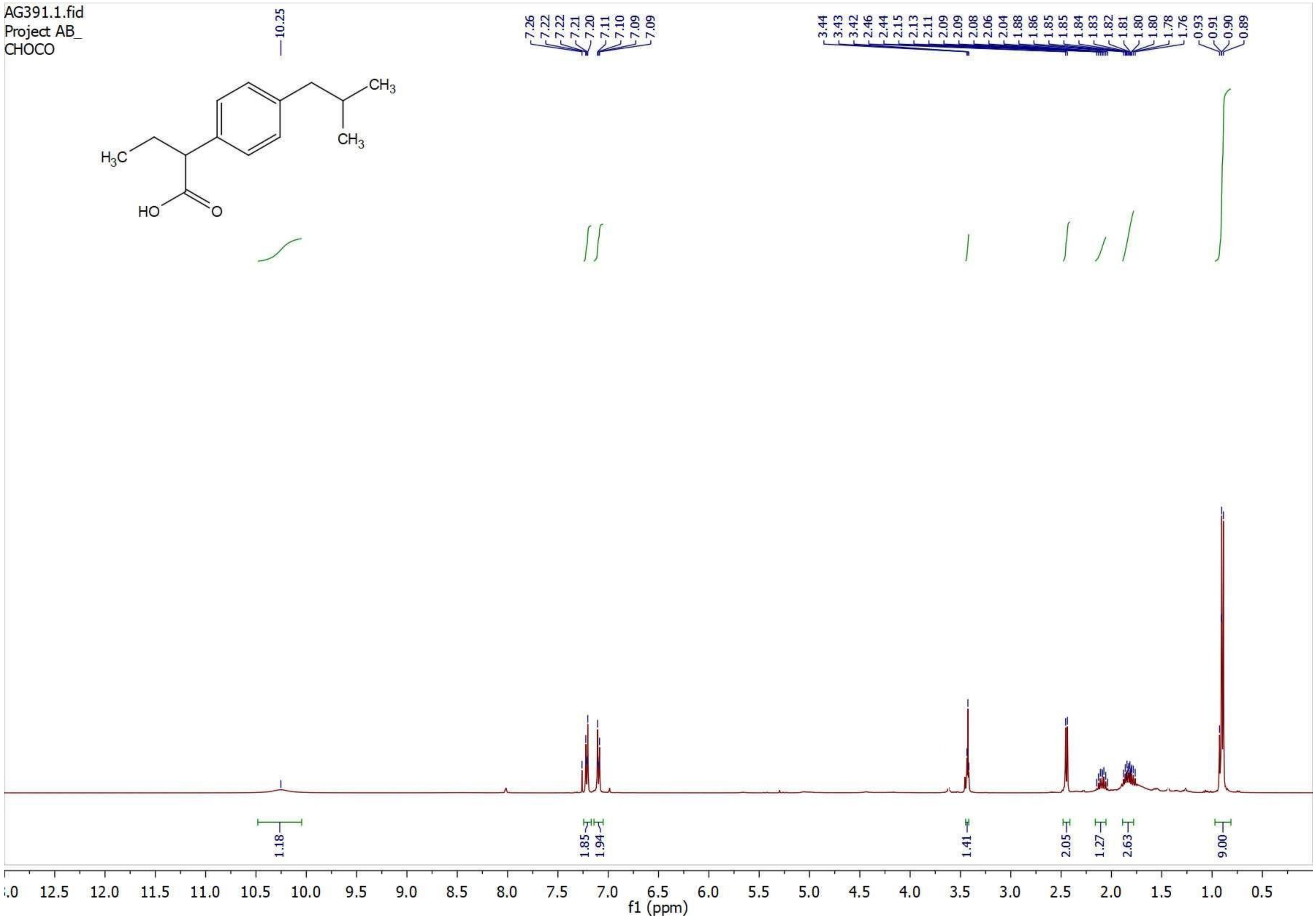
AG391.1.fid
Project AB_
CHOCO
10.25
7.26
7.22
7.22
7.21
7.20
7.11
7.10
7.09
7.09
3.44
3.43
3.42
2.46
2.44
2.15
2.13
2.11
2.09
2.09
2.08
2.06
2.04
1.88
1.86
1.85
1.85
1.84
1.83
1.82
1.81
1.80
1.80
1.78
1.76
0.93
0.91
0.90
0.89
CH3
CH3
H3C
HO
O
1.18
1.85
1.94
1.41
2.05
1.27
2.63
9.00
12.5
12.0
11.5
11.0
10.5
10.0
9.5
9.0
8.5
8.0
7.5
7.0
6.5
6.0
5.5
5.0
4.5
4.0
3.5
3.0
2.5
2.0
1.5
1.0
0.5
f1 (ppm)

Compound 6b

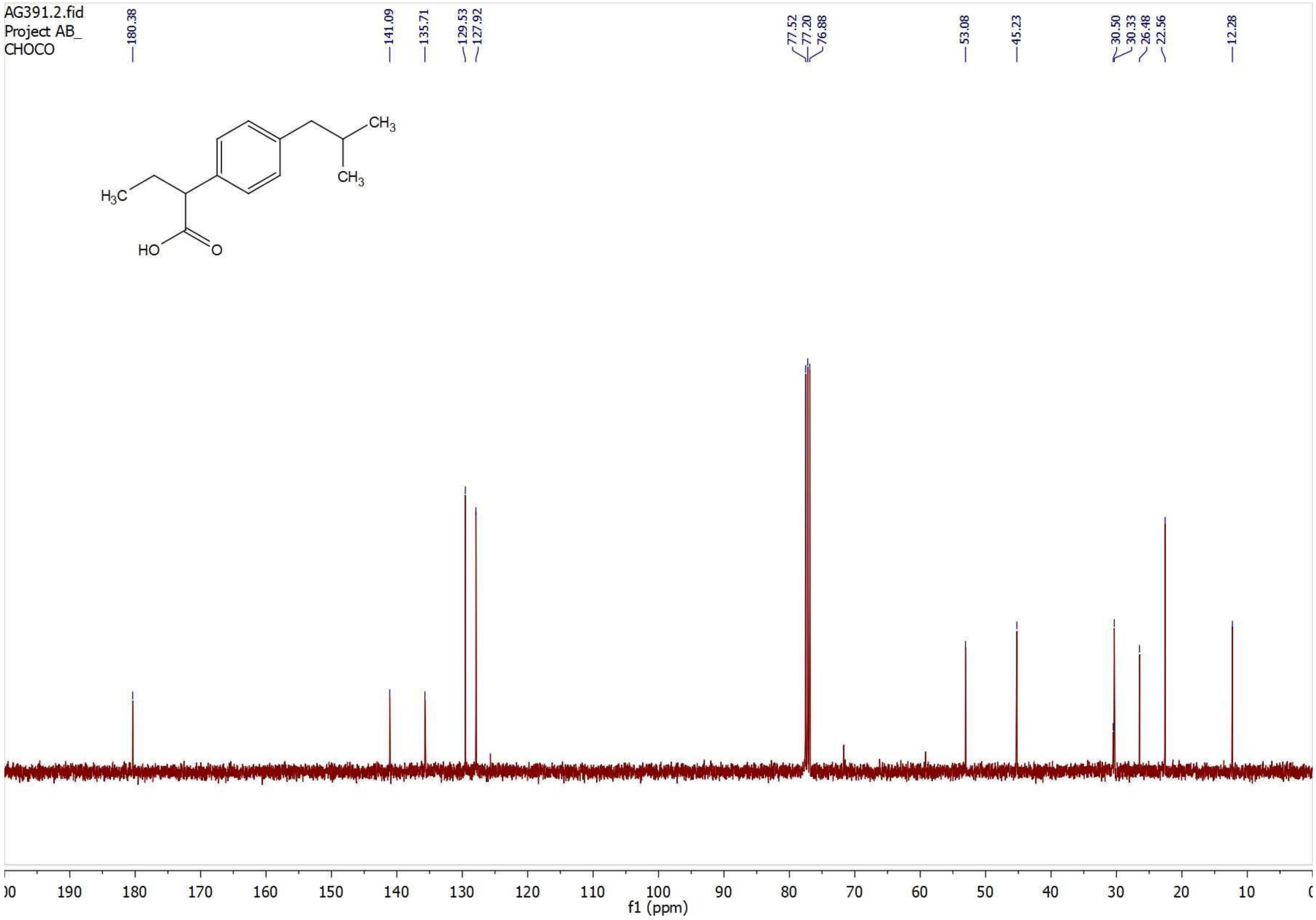
AG391.2.fid
Project AB_
CHOCO
180.38
141.09
135.71
129.53
127.92
77.52
77.20
76.88
53.08
45.23
30.50
30.33
26.48
22.56
12.28
CH3
CH3
H3C
HO
O
190
180
170
160
150
140
130
120
110
100
90
80
70
60
50
40
30
20
10
f1 (ppm)

Cu-catalyzed hydrocarboxylation of indene

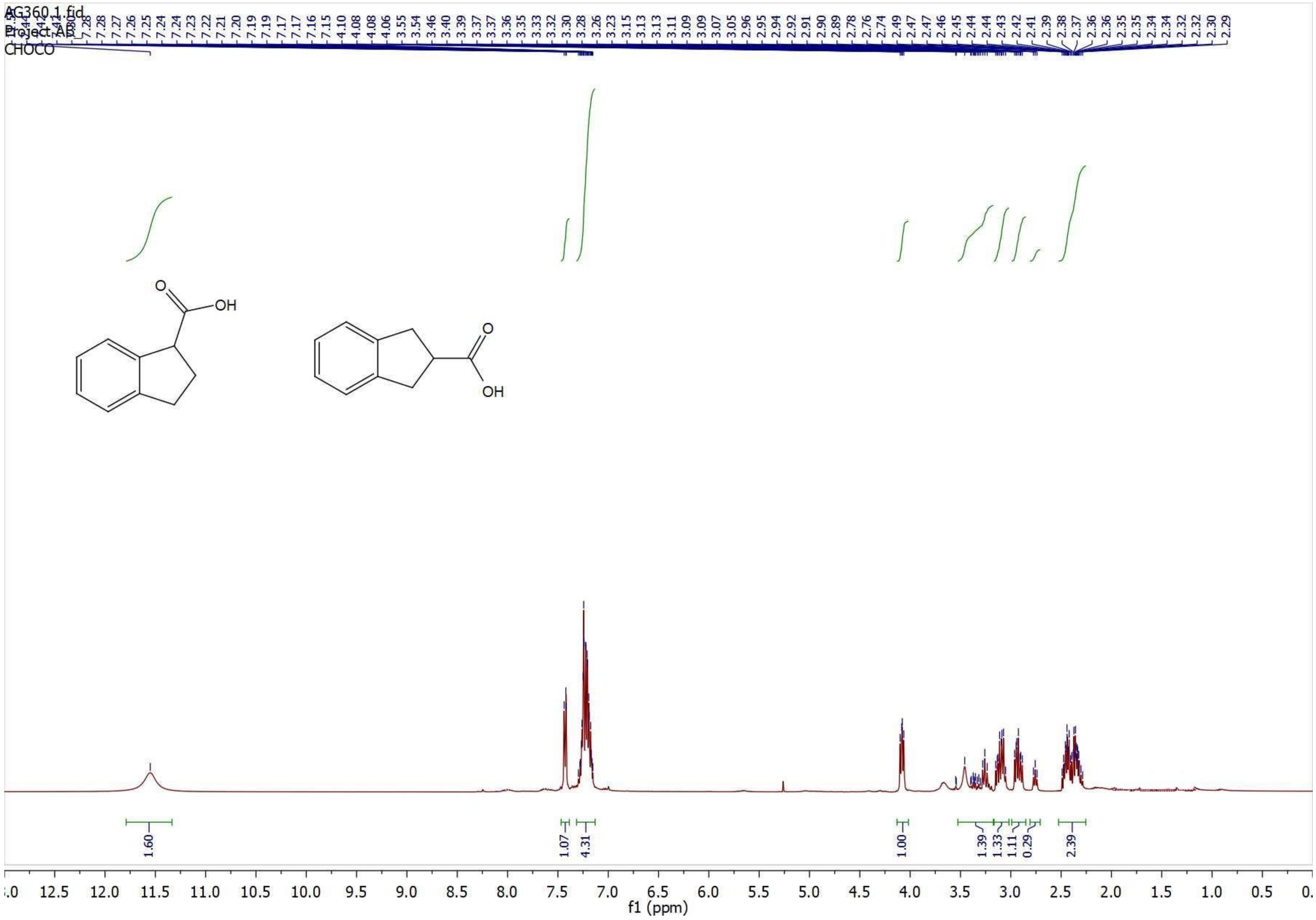

Cu-catalyzed hydrocarboxylation of indene

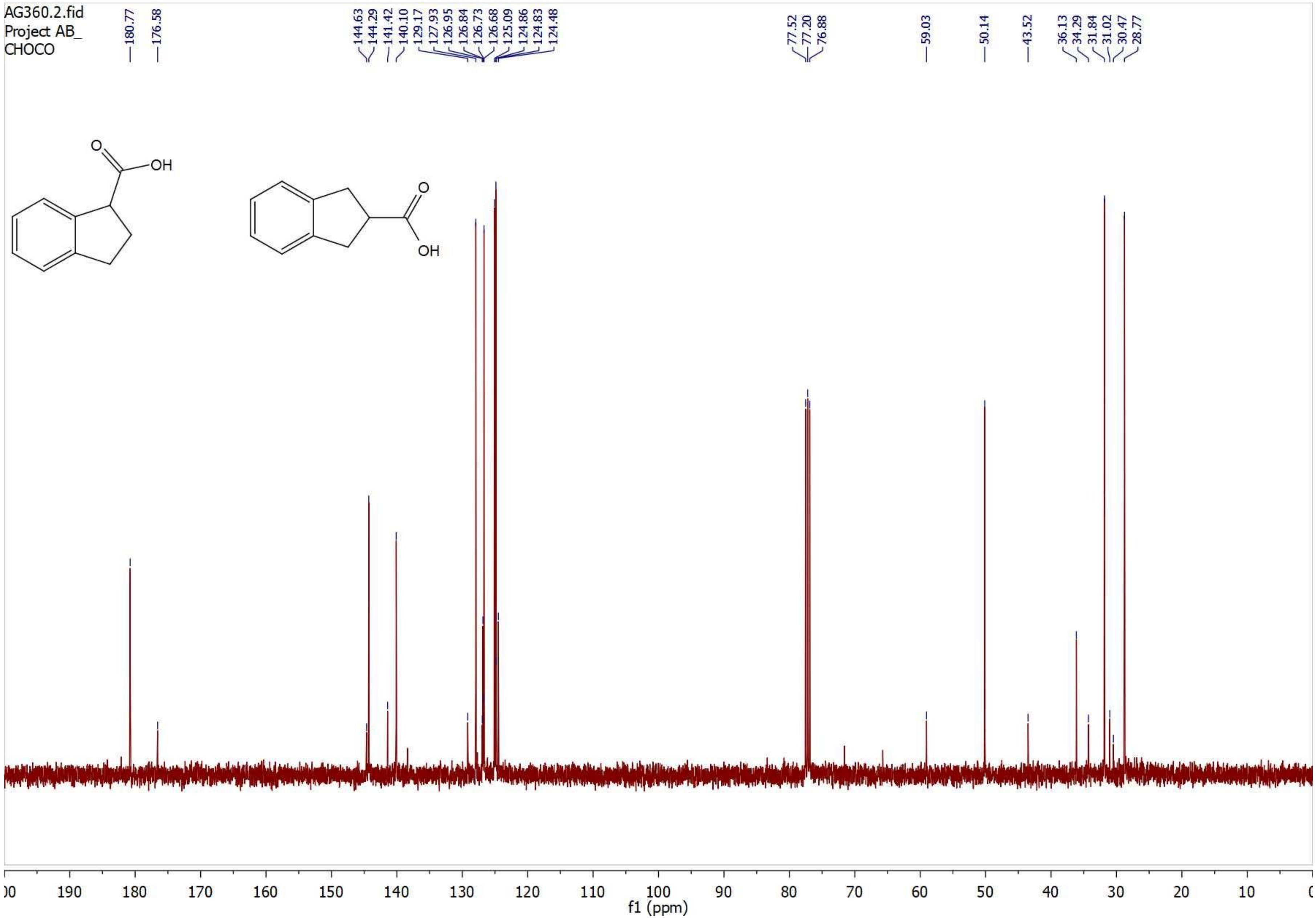

Chromatogram of Cu-catalyzed hydrocarboxylation of indene

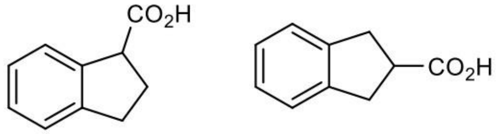

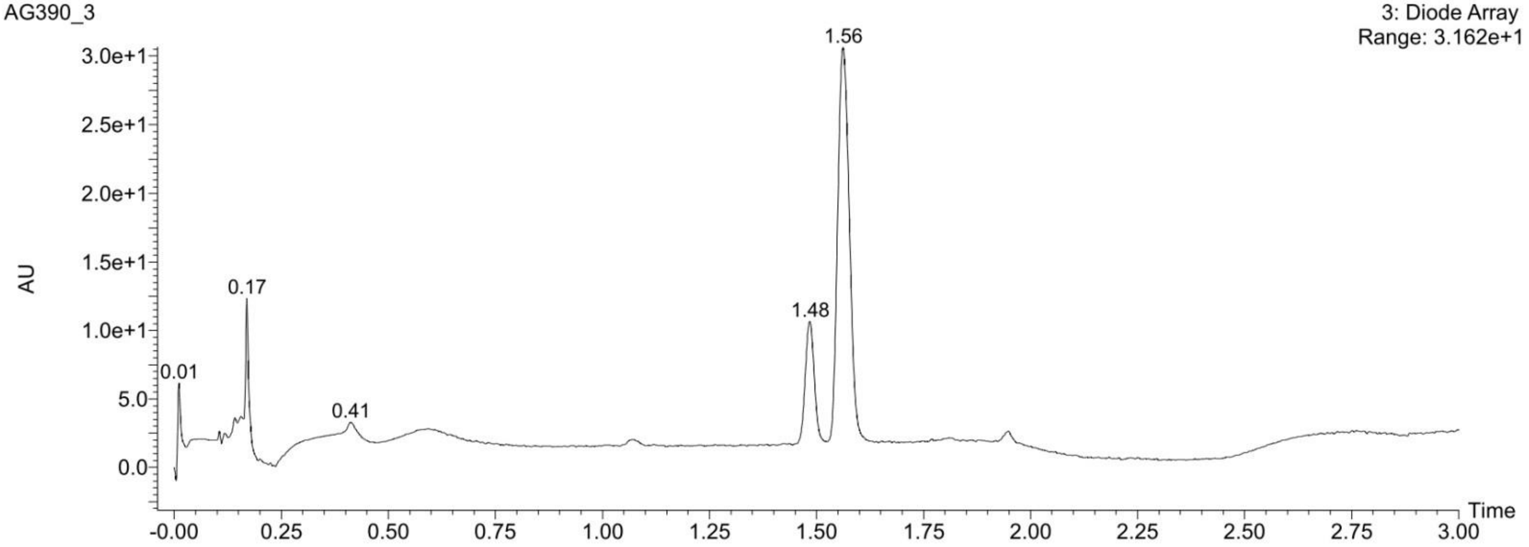

diol column **26-Apr-2019 09:55:24** **UPC2**

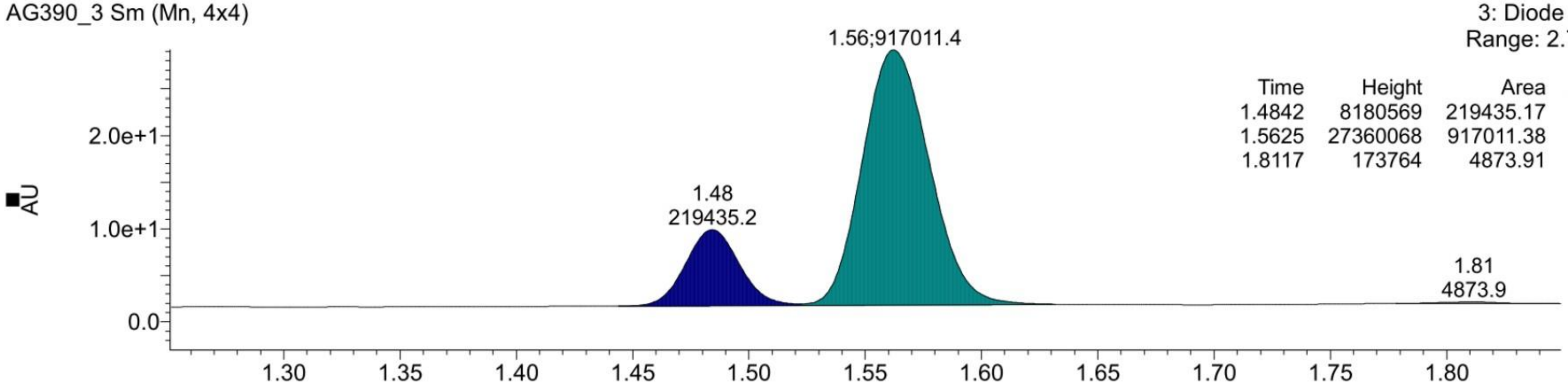

Cu-catalyzed hydrocarboxylation of 1,2-dihydronaphthalene

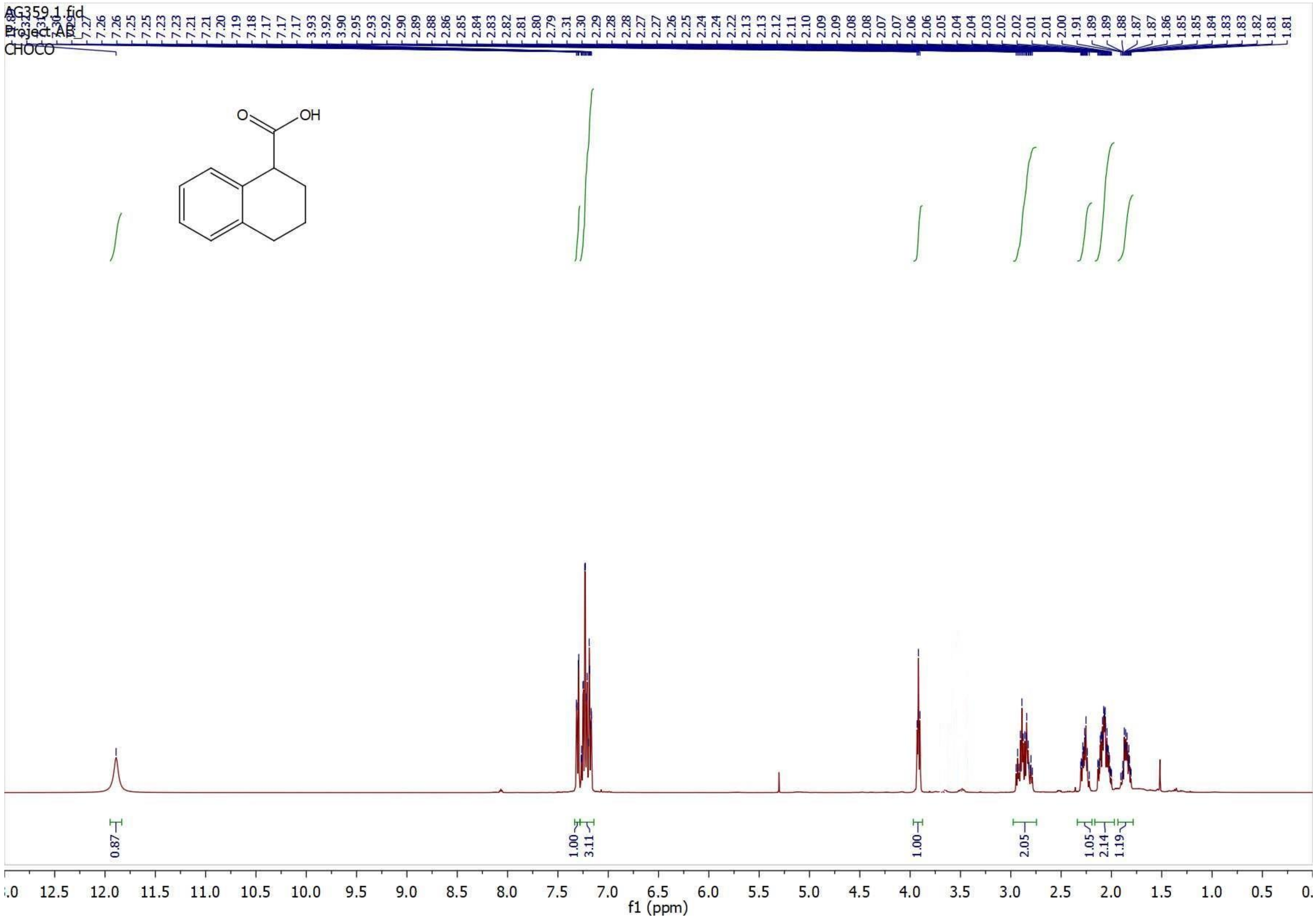

Cu-catalyzed hydrocarboxylation of 1,2-dihydronaphthalene

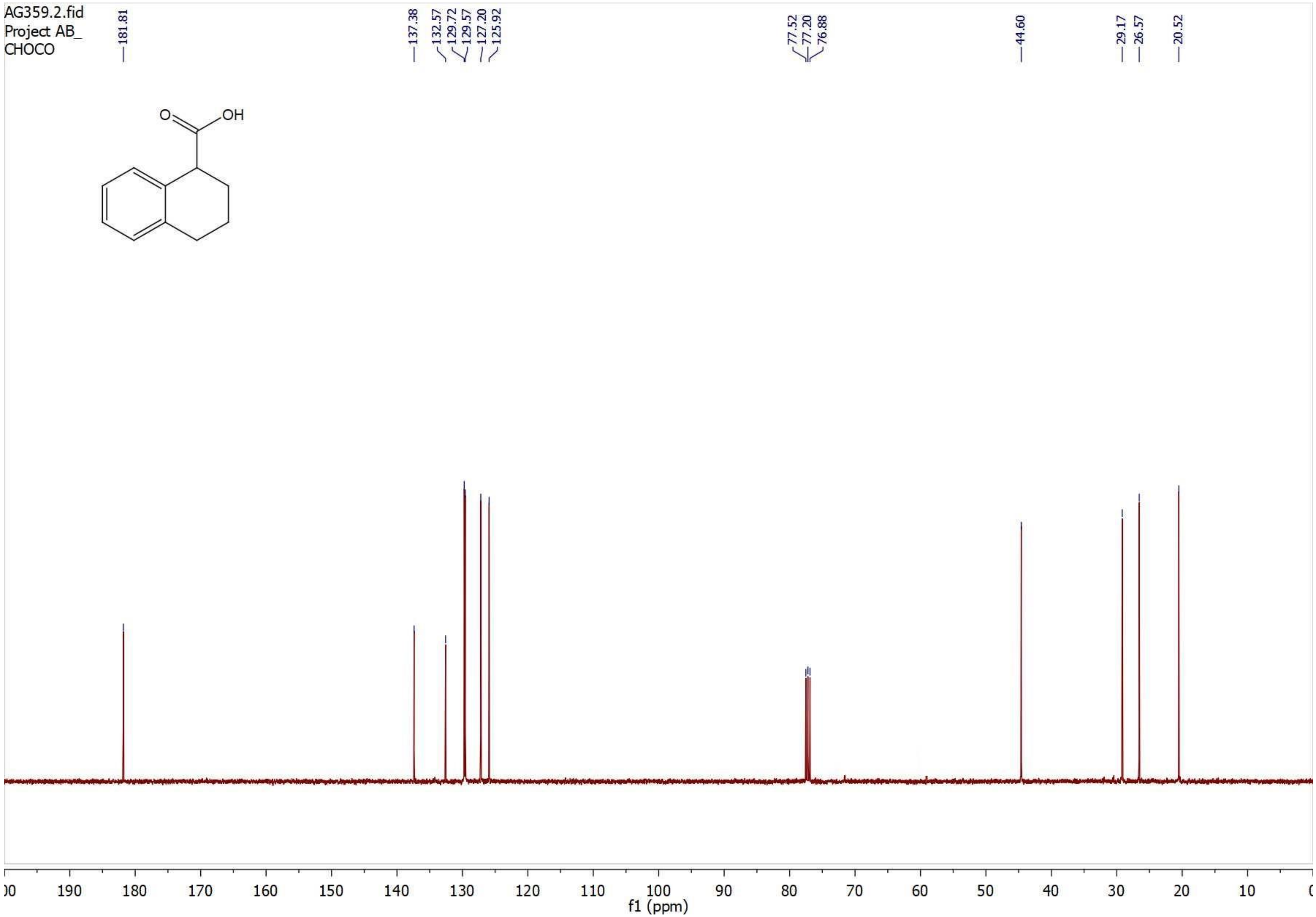

Chromatogram of Cu-catalyzed hydrocarboxylation of 1,2-dihydronaphthalene *vs* 3g

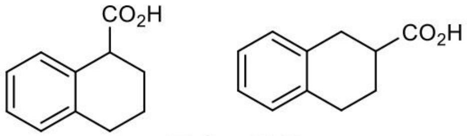

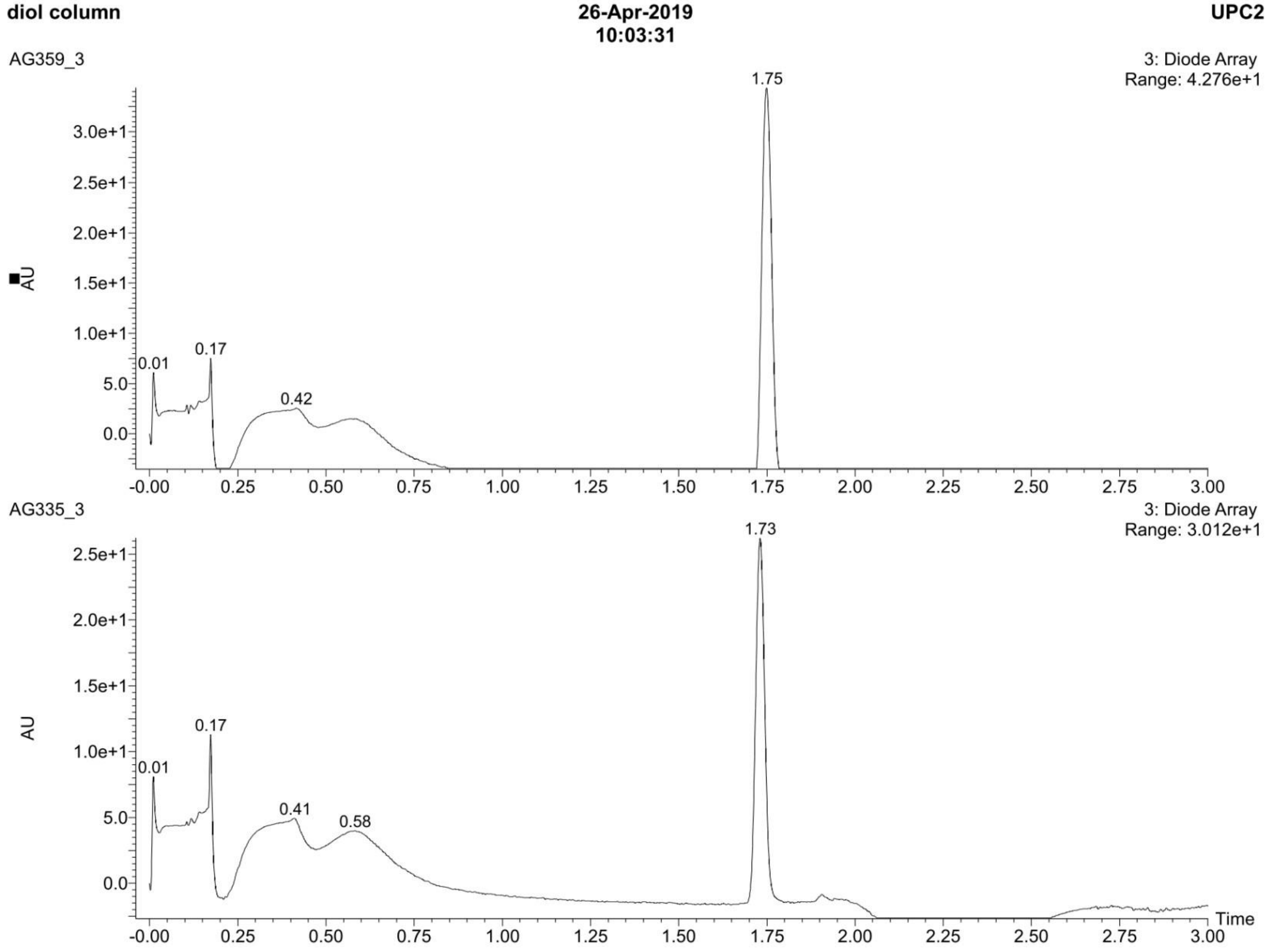

Compound 9a

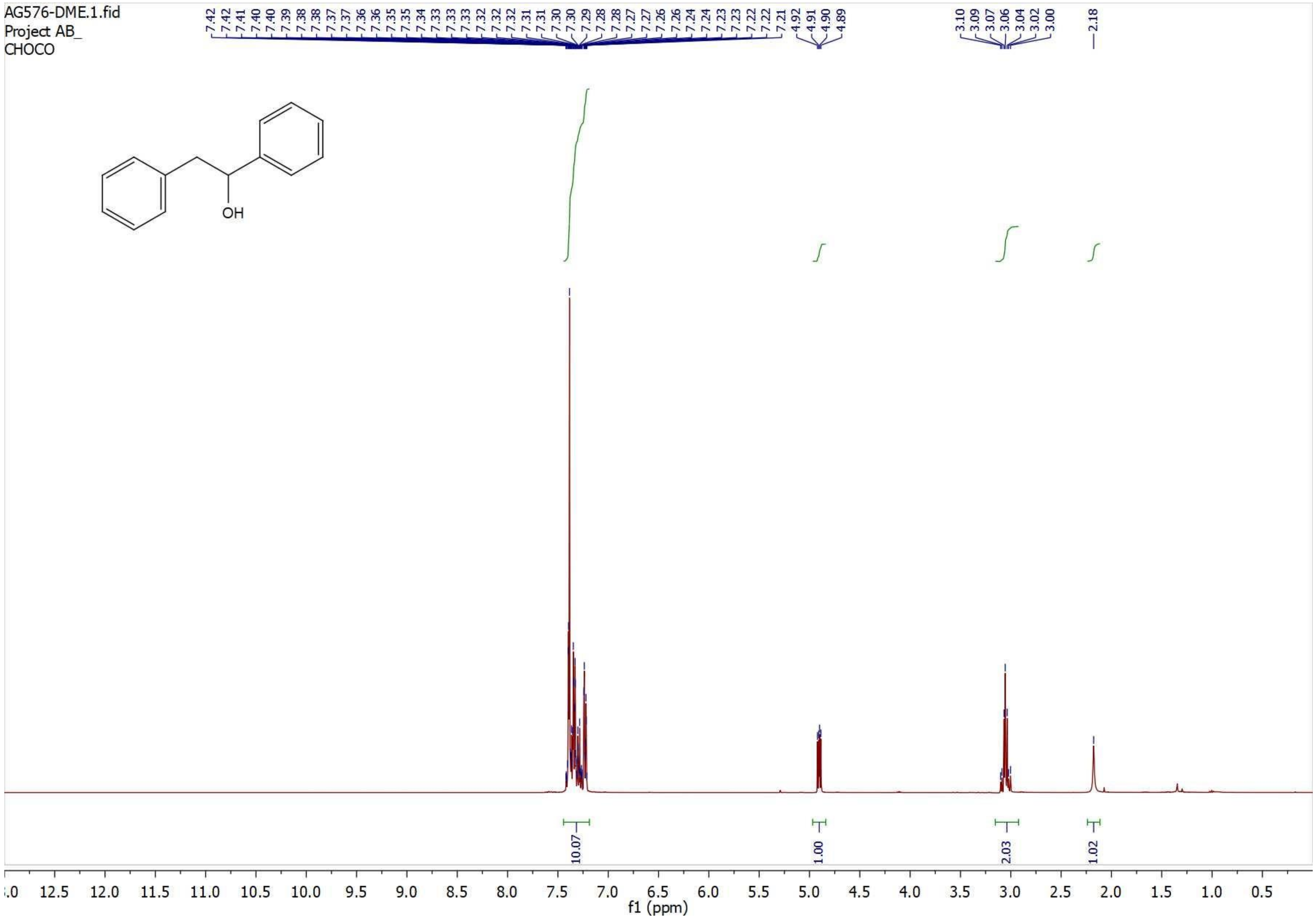

AG576-DME.1.fid
Project AB_
CHOCO
7.42 7.42 7.41 7.40 7.40 7.39 7.38 7.38 7.37 7.37 7.36 7.36 7.35 7.35 7.34 7.33 7.33 7.33 7.32 7.32 7.32 7.31 7.31 7.30 7.30 7.29 7.28 7.28 7.27 7.27 7.26 7.26 7.24 7.24 7.23 7.23 7.22 7.22 7.21
4.92 4.91 4.90 4.89
3.10 3.09 3.07 3.06 3.04 3.02 3.00
2.18
OH
10.07
1.00
2.03
1.02
12.5 12.0 11.5 11.0 10.5 10.0 9.5 9.0 8.5 8.0 7.5 7.0 6.5 6.0 5.5 5.0 4.5 4.0 3.5 3.0 2.5 2.0 1.5 1.0 0.5
f1 (ppm)

Compound 9a

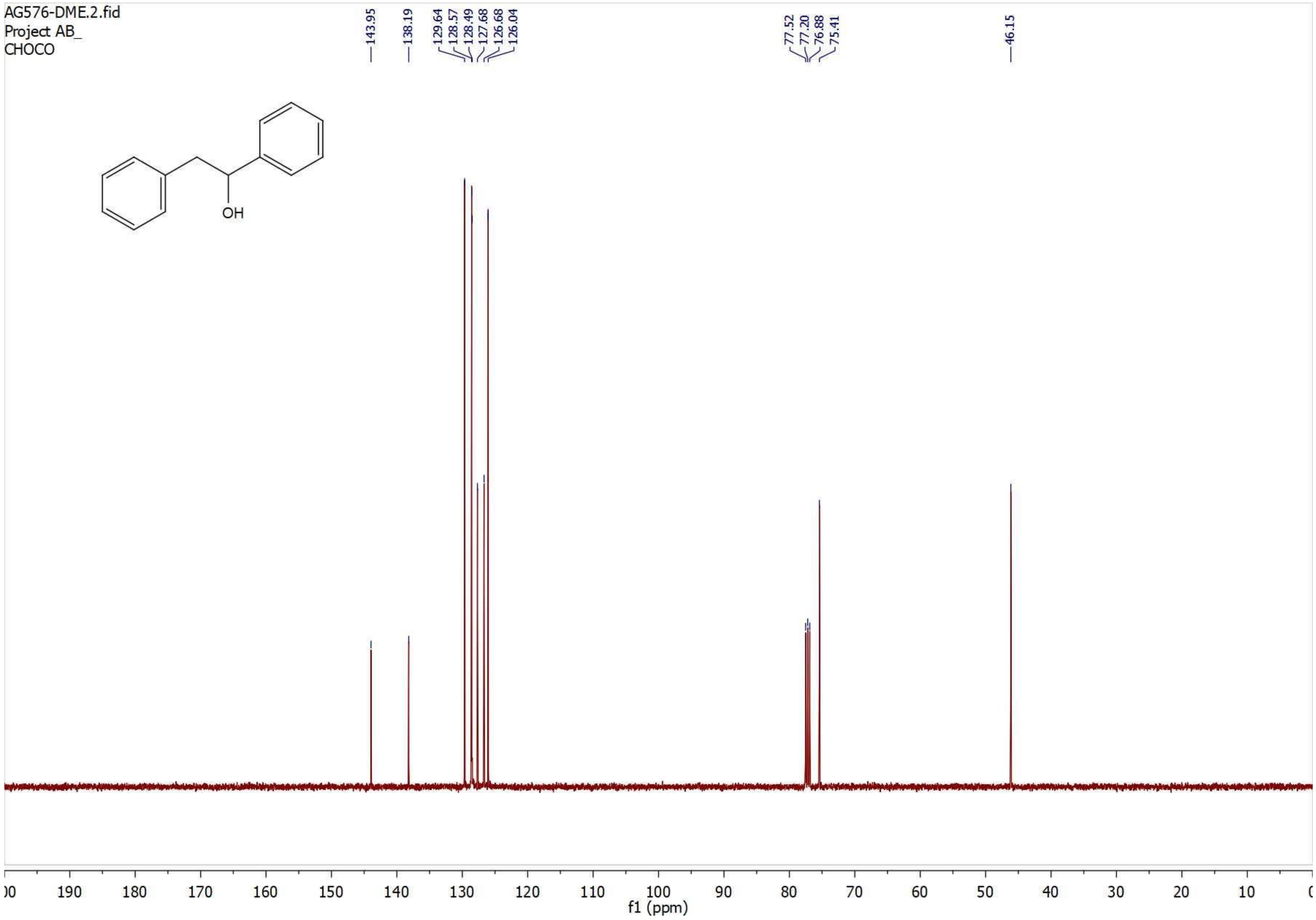
AG576-DME.2.fid
Project AB_
CHOCO
143.95
138.19
129.64
128.57
128.49
127.68
126.68
126.04
77.52
77.20
76.88
75.41
46.15
OH
190
180
170
160
150
140
130
120
110
100
90
80
70
60
50
40
30
20
10
f1 (ppm)

Asymmetric hydroboration-oxidation of *trans*-ß-methylstyrene

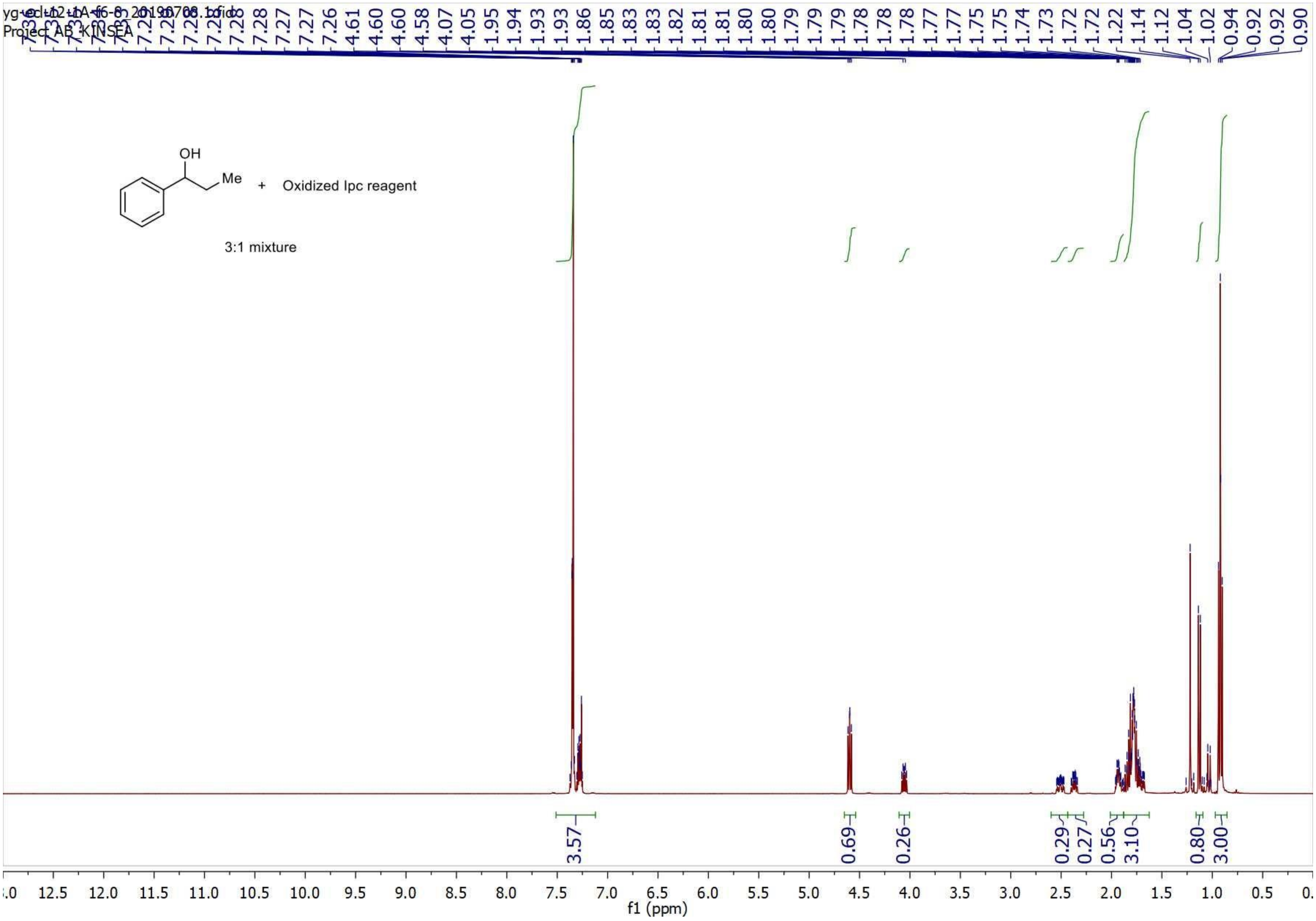

Asymmetric hydroboration-oxidation of *trans*-ß-methylstyrene

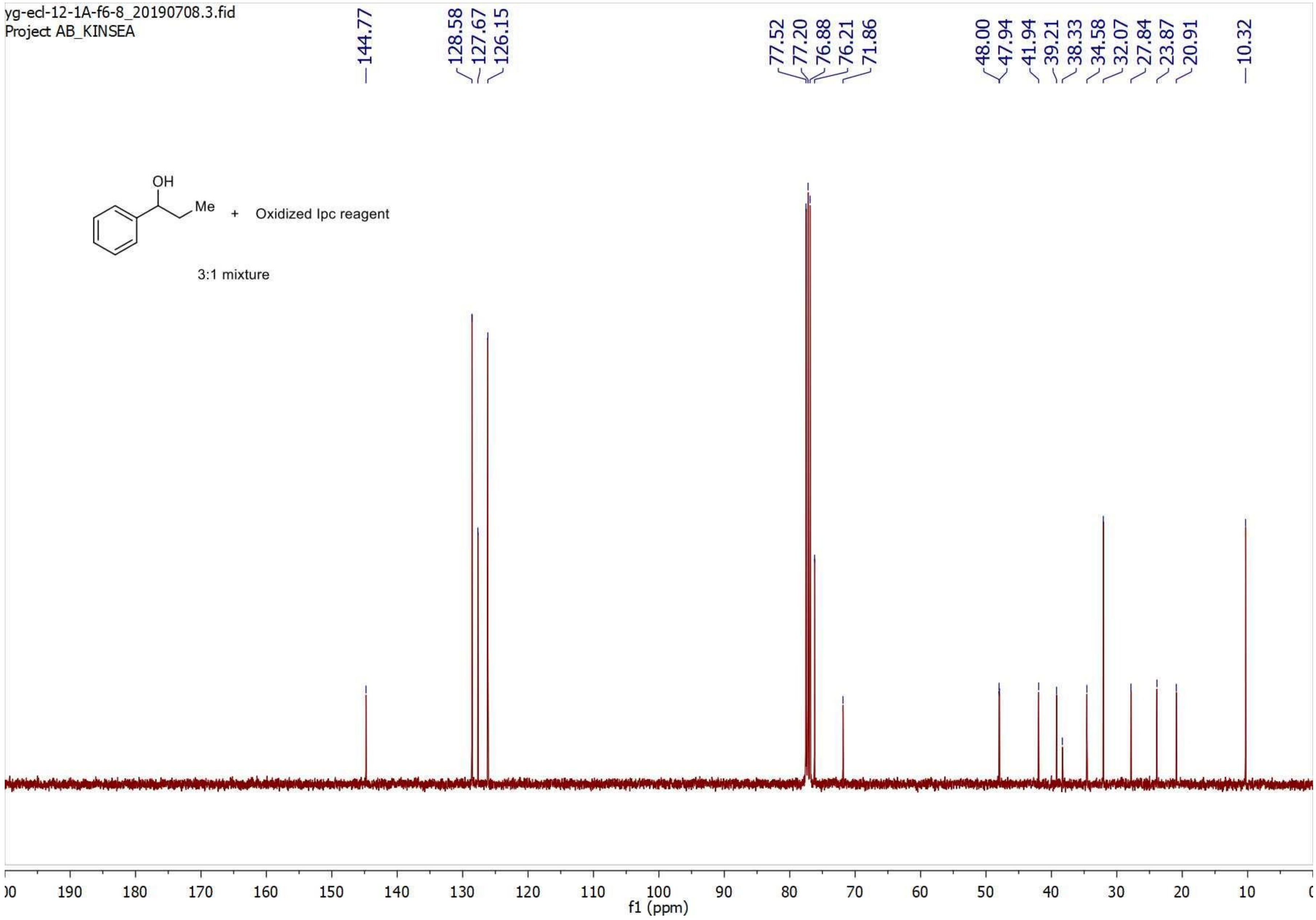

Asymmetric hydroboration-oxidation of *trans*-ß-methylstyrene

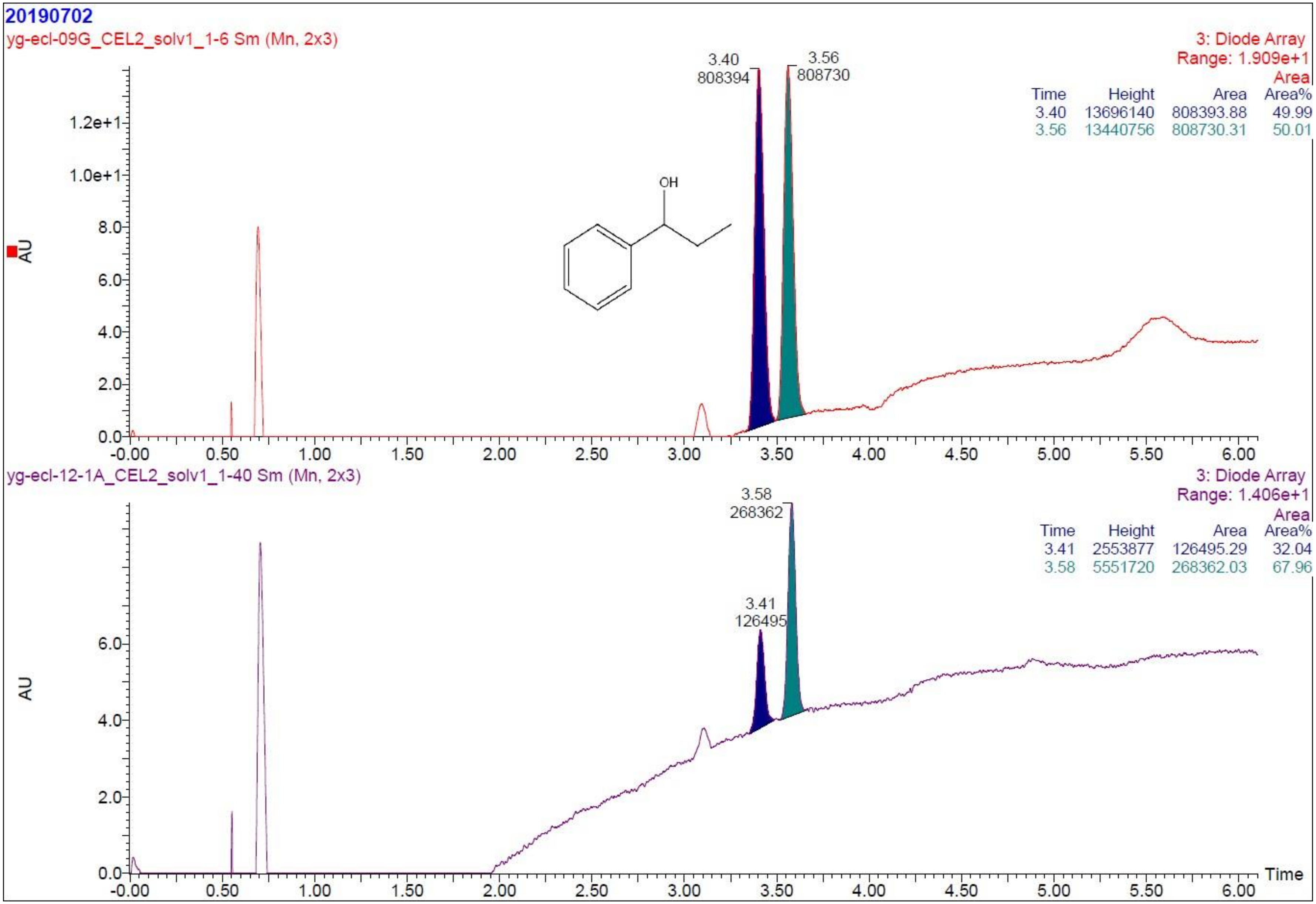

Carboxylation of enantiomerically enriched organoborane intermediate

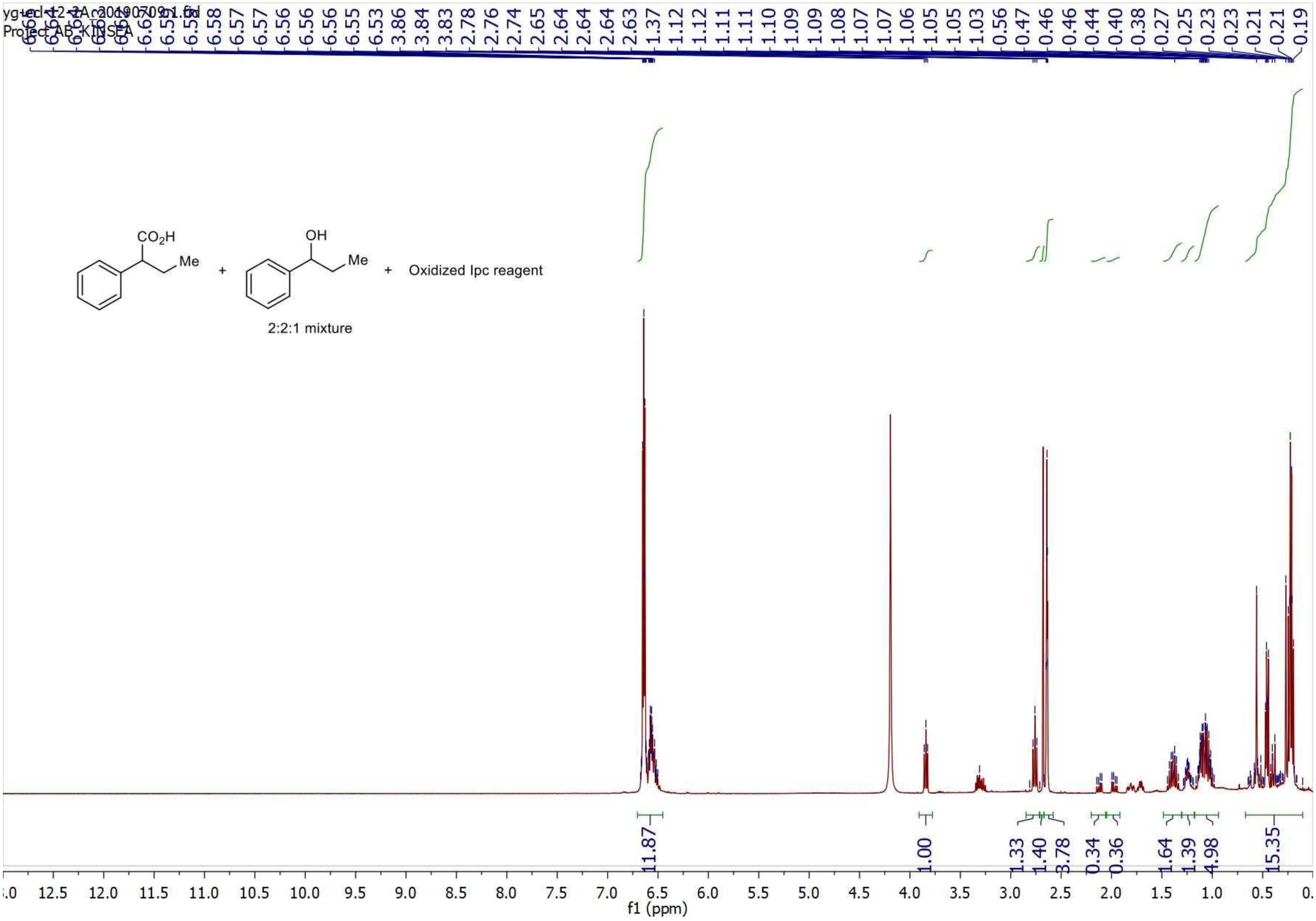

Carboxylation of enantiomerically enriched organoborane intermediate

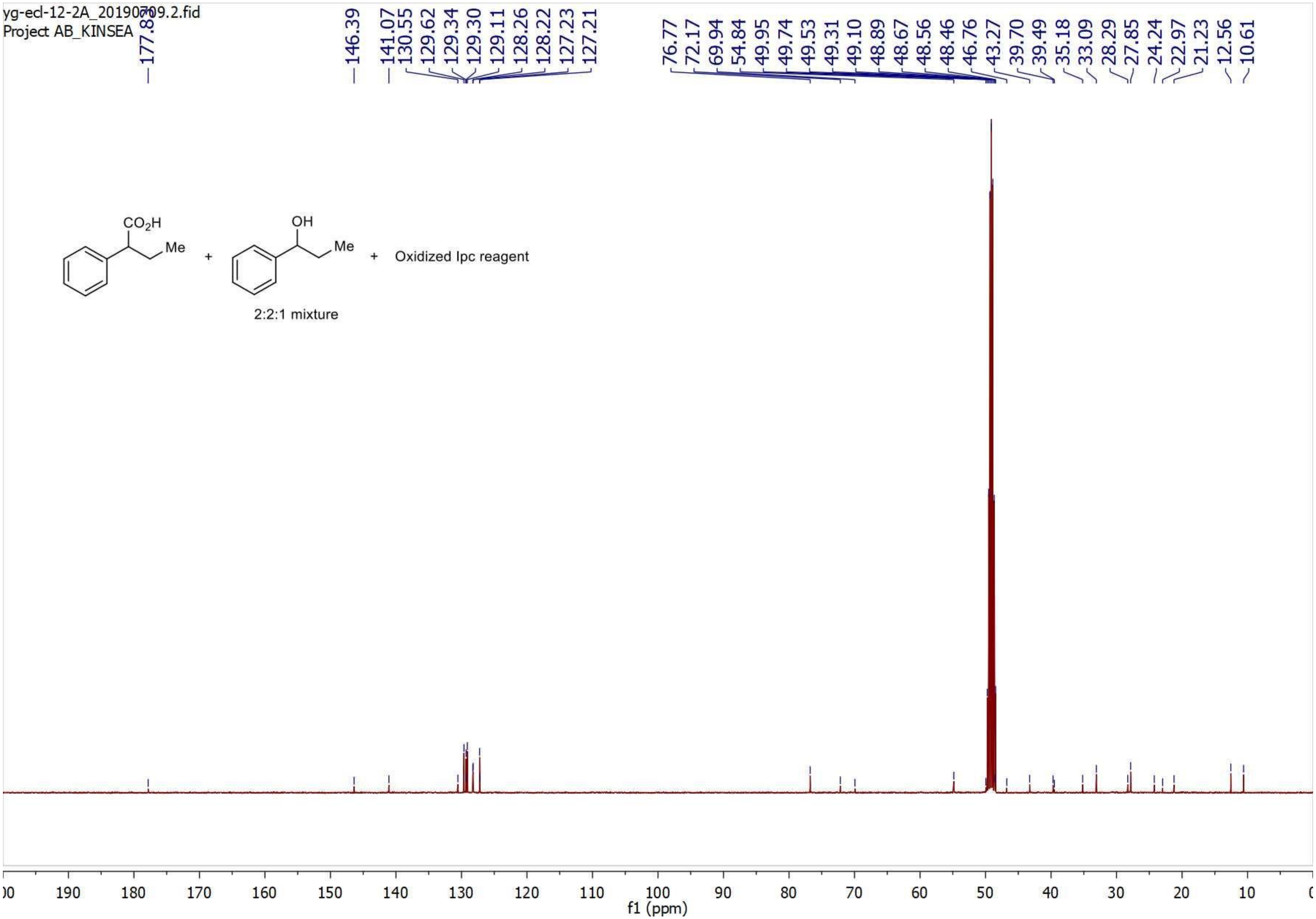

Carboxylation of enantiomerically enriched organoborane intermediate

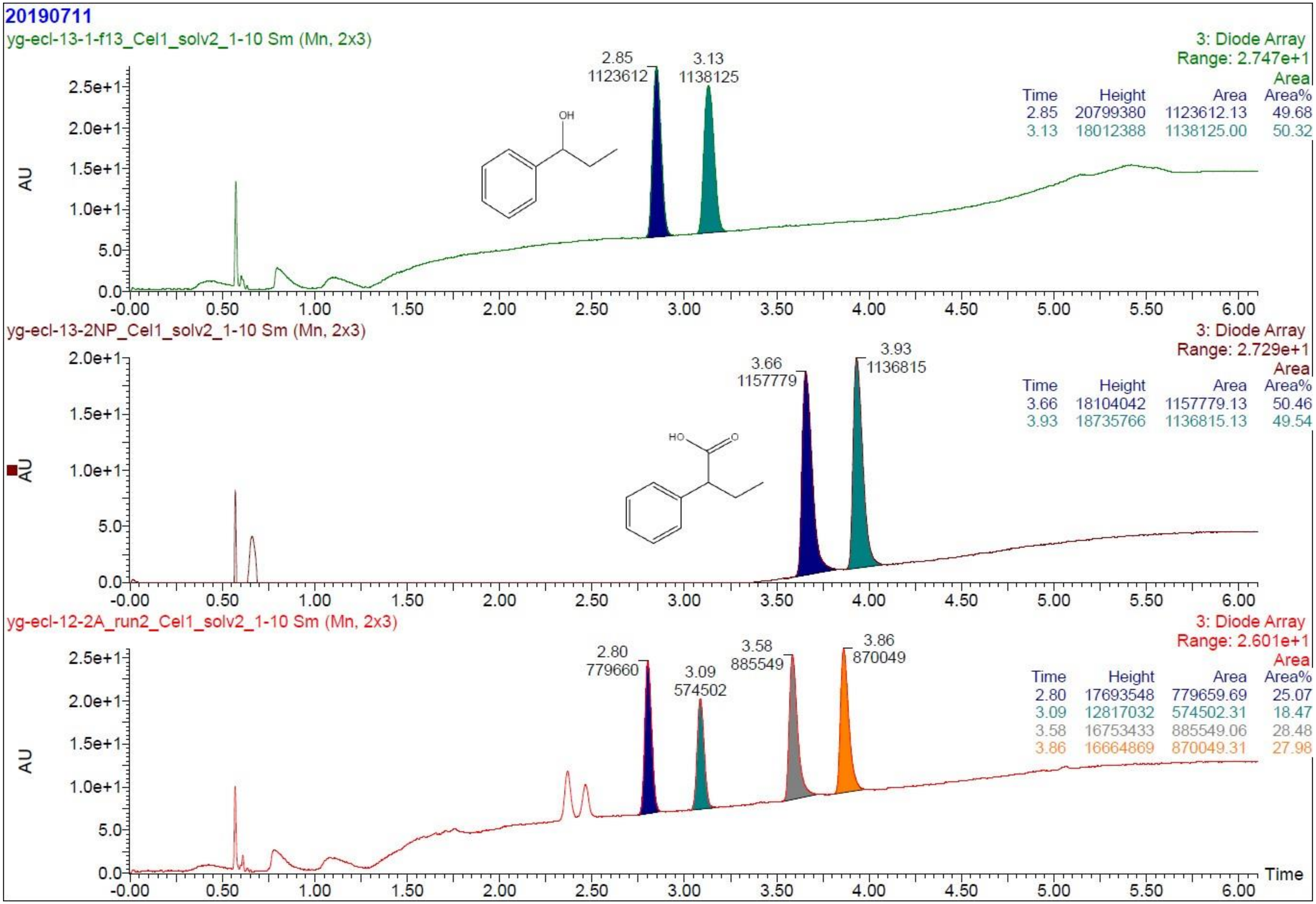

Computational details

Models: Full molecular models of **b1, b2** and **b3** were computed. For CsF, we observed in computations that a CsF dimer is much more stable than 2 CsF monomers. The CsF dimer was therefore used as a reference point, implying that addition of 1 molecule of CsF was computed as addition of ½ $(CsF)_2$. This reference point affects the resting state (**i1**) of the preferred reaction mechanisms **B** and **C** and therefore the overall barriers of the reaction.

Geometry optimizations with Gaussian: All DFT calculations were performed using the Gaussian16 Revision B.01 software package.[19] The DFT functional employed was ω-B97XD.[20] Other DFT functionals (PBE+GD3BJ,[21] PBE0+GD3BJ,[22] B3LYP+GD3[23] and CAM-B3LYP+GD3BJ[24]) were tested but with these we were unable to identify the TSs for reaction mechanism **C**. This might be a results of the missing description of long-range interaction for most of the functionals, which are important for mechanism **C**. Geometry optimizations utilized basis set BS1, consisting of the SDD basis set for Cs and 6-31+G* for all other elements. Symmetry was not used. Solvation effects were included via the model IEFPCM (1,4-dioxane). Obtained minima and transition states (TS) geometries were controlled via vibrational mode analysis to show zero (minimum) or only one (TS) imaginary frequency. Gibbs energies were computed at 393.15 K (120 ºC) at the BS1 level.

GRRM calculations: A number of initial structures for the TS optimization were generate by using the MC-AFIR[25] (Multi-Component Artificial Force Induced Reaction) method from the GGRM14 software.[26] The software was interfaced with Gaussian09 Revision D.01[27] and

[19] Gaussian 16, Revision B.01, M. J. Frisch, G. W. Trucks, H. B. Schlegel, G. E. Scuseria, M. A. Robb, J. R. Cheeseman, G. Scalmani, V. Barone, G. A. Petersson, H. Nakatsuji, X. Li, M. Caricato, A. V. Marenich, J. Bloino, B. G. Janesko, R. Gomperts, B. Mennucci, H. P. Hratchian, J. V. Ortiz, A. F. Izmaylov, J. L. Sonnenberg, D. Williams-Young, F. Ding, F. Lipparini, F. Egidi, J. Goings, B. Peng, A. Petrone, T. Henderson, D. Ranasinghe, V. G. Zakrzewski, J. Gao, N. Rega, G. Zheng, W. Liang, M. Hada, M. Ehara, K. Toyota, R. Fukuda, J. Hasegawa, M. Ishida, T. Nakajima, Y. Honda, O. Kitao, H. Nakai, T. Vreven, K. Throssell, J. A. Montgomery, Jr., J. E. Peralta, F. Ogliaro, M. J. Bearpark, J. J. Heyd, E. N. Brothers, K. N. Kudin, V. N. Staroverov, T. A. Keith, R. Kobayashi, J. Normand, K. Raghavachari, A. P. Rendell, J. C. Burant, S. S. Iyengar, J. Tomasi, M. Cossi, J. M. Millam, M. Klene, C. Adamo, R. Cammi, J. W. Ochterski, R. L. Martin, K. Morokuma, O. Farkas, J. B. Foresman, and D. J. Fox, Gaussian, Inc., Wallingford CT, 2016.
[20] J.-D. Chai, M. Head-Gordon. Phys.Chem.Chem.Phys. **2008**, 10, 6615-6620
[21] J. P. Perdew, K. Burke, and M. Ernzerhof. Phys. Rev. Lett. **1996**, 77, 3865-3868
[22] C. Adamo and V. Barone. J. Chem. Phys. **1999**, 110, 6158-69
[23] A. D. Becke. J. Chem. Phys. **1993**, 98, 5648-52
[24] T. Yanai, D. Tew, and N. Handy, Chem. Phys. Lett. **2004**, 393, 51-570
[25] S. Maeda, Y. Harabuchi, M. Takagi, K. Saita, K. Suzuki, T. Ichino, Y. Sumiya, K. Sugiyama, Y. Ono, Chem. Rec. **2016**, 16, 2232-2248
[26] S. Maeda, K. Ohno, K. Morokuma, Phys. Chem. Chem. Phys. **2013**, 15, 3683-3701.
[27] Gaussian 09, Revision D.01, M. J. Frisch, G. W. Trucks, H. B. Schlegel, G. E. Scuseria, M. A. Robb, J. R. Cheeseman, G. Scalmani, V. Barone, B. Mennucci, G. A. Petersson, H. Nakatsuji, M. Caricato, X. Li, H. P. Hratchian, A. F. Izmaylov, J. Bloino, G. Zheng, J. L. Sonnenberg, M. Hada, M. Ehara, K. Toyota, R. Fukuda, J. Hasegawa, M. Ishida, T. Nakajima, Y. Honda, O. Kitao, H. Nakai, T. Vreven, J. A. Montgomery, Jr., J. E. Peralta, F. Ogliaro, M. Bearpark, J. J. Heyd, E. Brothers, K. N. Kudin, V. N. Staroverov, T. Keith, R. Kobayashi, J. Normand, K. Raghavachari, A. Rendell, J. C. Burant, S. S. Iyengar, J. Tomasi, M. Cossi, N. Rega, J. M. Millam, M. Klene, J. E. Knox, J. B. Cross, V. Bakken, C. Adamo, J. Jaramillo, R. Gomperts, R. E. Stratmann, O. Yazyev, A. J. Austin, R. Cammi, C. Pomelli, J. W. Ochterski,

the same level of theory as described above was used. The termination criterion for the calculation was set to 20 runs without new results (NFault = 20).

Coupled-cluster single point calculations: SP electronic energies on the coupled-cluster level were obtained with the ORCA 4.1.1[28] software using the geometries optimized with w-B97XD. The used method was DLPNO-CCSD(T)[29] together with the scalar-relativistic ZORA Hamiltonian.[30] The basis set was SARC-ZORA-TZVPP for Cs and ZORA-def2-QZVPP for all other elements. Calculations were accelerated by using the RIJCOSX approximation[31] with auxiliary basis set SARC/J and def2-TZVP/C. An example of the ORCA input can be found below.

Energies: Standard state conversions (SS) were computed at 393.15 K. Two sets of energies are compared below: DFT-based Gibbs free energies ($\Delta G_{\omega\text{-B97XD}}$) and DPLNO-CCSD(T)–based Gibbs free energies ($\Delta G_{\text{DPLNO-CCSD(T)//}\omega\text{-B97XD}}$).
The *DFT Gibbs free energies* were obtained by computing single point electronic energies (including IEFPCM) with basis set 2 (BS2), which corresponds to def2-TZVP level for all elements (with SDD ECP for Cs). Counterpoise (CP_{BS2}) corrections were computed at the same level of theory, providing the following final DFT energies:

$$\Delta G_{\omega\text{-B97XD}} = \Delta G_{\omega\text{-B97XD/IEFPCM/BS1}} - \Delta E_{\omega\text{-B97XD/IEFPCM/BS1}} + \Delta E_{\omega\text{-B97XD/IEFPCM/BS2}} + CP_{BS2} + SS.$$

The *DPLNO-CCSD(T)–based Gibbs free energies* were computed by combining the DPLNO-CCSD(T) single point energies with the DFT-based vibrational, entropic and temperature corrections, and the standard state corrections (SS, 393.15 K). Counter poise corrections were not included. The final DPLNO-CCSD(T)–based energies (which are those discussed in the main text) are:

$$\Delta G_{\text{DPLNO-CCSD(T)//}\omega\text{-B97XD}} = \Delta G_{\omega\text{-B97XD/IEFPCM/BS1}} - \Delta E_{\omega\text{-B97XD/IEFPCM/BS1}} + \Delta E_{\text{DPLNO-CCSD(T)}} + SS.$$

We further tested if addition of DFT solvent corrections (computed as the difference between vacuum and IEFPCM single points, $\Delta E_{\text{sol\_}\omega\text{-B97XD/BS1}} = \Delta E_{\omega\text{-B97XD/IEFPCM/BS1}} - \Delta E_{\omega\text{-B97XD/VACUUM/BS1}}$) affected the DPLNO-CCSD(T)–based Gibbs free energies. As shown in Table S2-S4 (energies in brackets), the solvent correction does not affect the overall barriers significantly and it was therefore not included in the energies given in the main text.

R. L. Martin, K. Morokuma, V. G. Zakrzewski, G. A. Voth, P. Salvador, J. J. Dannenberg, S. Dapprich, A. D. Daniels, O. Farkas, J. B. Foresman, J. V. Ortiz, J. Cioslowski, and D. J. Fox, Gaussian, Inc., Wallingford CT, 2013.

[28] (a) F. Neese, The ORCA program system, Wiley Interdisciplinary Reviews: Computational Molecular Science, 2012, Vol. 2, Issue 1, 73-78 (b) F. Neese, Software update: the ORCA program system, version 4.0, Wiley Interdisciplinary Reviews: Computational Molecular Science, **2017**, Vol. 8, Issue 1, 1327.

[29] Y. Guo, C. Riplinger, U. Becker, D. Liakos, Y. Minenkov, L. Cavallo, F. Neese, J. Chem. Phys. **2018**, 148, 011101

[30] C. van Wüllen, J. Chem. Phys. **1998**,109, 392-399.

[31] F. Neese, F. Wennmohs, A. Hansen, U. Becker, J. Chem. Phys. **2009**, 356, 98.

EXAMPLE INPUT DPLNO-CCSD(T):

```
###
!DLPNO-CCSD(T) ZORA TightSCF SlowConv RIJCOSX
!Printbasis
!Smallprint
%maxcore 5000
%PAL
NProcs 16
END
%BASIS
Basis "ZORA-def2-QZVPP"
Aux "SARC/J"
AuxC "def2-TZVP/C"
NewGTO Cs "SARC-ZORA-TZVPP" end
END
%SCF
Convergence Tight
MaxIter 1000
END
* xyz 0 1
“Cartesian coordinates”
*
###
```

DFT and DLPNO-CCSD(T) results

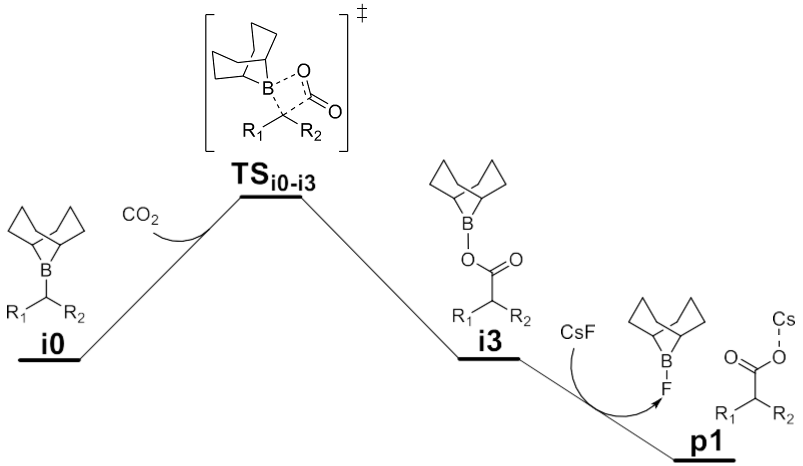

Figure S6. Reaction mechanism A.

Table S2: Computed energies (kcal/mol) for mechanism **A** for substrates **b1, b2** and **b3**. Numbers in bold are $\Delta G_{DPLNO\text{-}CCSD(T)//\omega\text{-}B97XD}$ energies (given in the main text), numbers in brackets are $\Delta G_{DPLNO\text{-}CCSD(T)//\omega\text{-}B97XD}$ energies with solvent corrections added ($\Delta E_{sol\_\omega\text{-}B97XD/BS1}$), whereas energies in parentheses are pure DFT $\Delta G_{\omega\text{-}B97XD}$ energies.

Substrate	**TS_{i0-i3}**	**i3**	**p1**	**Overall barrier**
**b1**	**57.4**<br>[58.1]<br>(62.5)	**-2.5**<br>[-2.8]<br>(-3.6)	-17.7<br>[-15.3]<br>(-12.9)	72.9<br>[68.4]<br>(75.4)
**b2**	**52.9**<br>[53.4]<br>(56.8)	-4.1<br>[-4.5]<br>(-6.1)	-14.5<br>[-13.6]<br>(-10.7)	67.5<br>[63.0]<br>(68.5)
**b3**	**57.7**<br>[58.2]<br>(62.1)	**-6.1**<br>[-6.4]<br>(-10.3)	-17.2<br>[-16.9]<br>(-10.0)	57.7<br>[58.2]<br>(62.1)

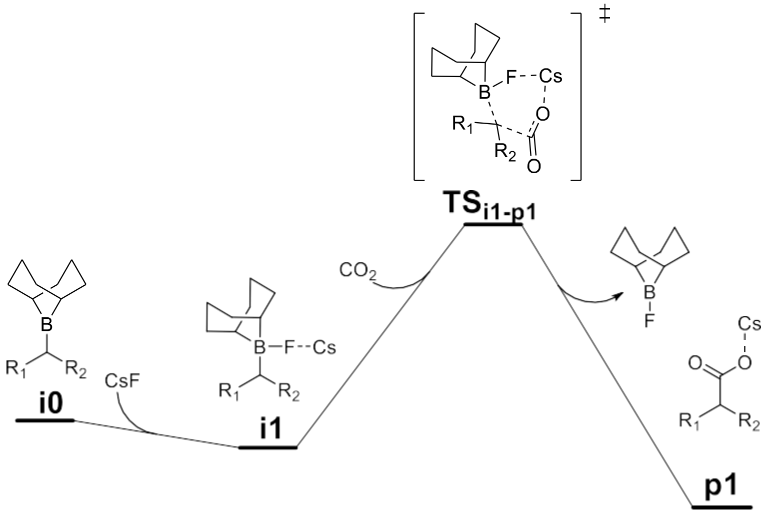

Figure S7. Reaction mechanism B.

Table S3: Computed energies (kcal/mol) for mechanism **B** for substrates **b1, b2** and **b3**. Numbers in bold are $\Delta G_{DPLNO\text{-}CCSD(T)//\omega\text{-}B97XD}$ energies (given in the main text), numbers in brackets are $\Delta G_{DPLNO\text{-}CCSD(T)//\omega\text{-}B97XD}$ energies with solvent corrections added ($\Delta E_{sol\_\omega\text{-}B97XD/BS1}$), whereas energies in parentheses are pure DFT $\Delta G_{\omega\text{-}B97XD}$ energies.

Substrate	**i1**	**TS_{i1-p1}**	**p1**	**Overall barrier**
**b1**	-15.5<br>[-10.3]<br>(-12.8)	36.3<br>[39.6]<br>(34.7)	-17.2<br>[-15.3]<br>(-12.9)	51.9<br>[49.9]<br>(47.6)
**b2**	-14.6<br>[-9.5]<br>(-11.7)	37.7<br>[40.6]<br>(36.6)	-14.5<br>[-13.6]<br>(-10.7)	52.3<br>[50.1]<br>(48.3)
**b3**	1.0<br>[3.1]<br>(6.2)	44.4<br>[47.6]<br>(47.6)	-17.2<br>[-16.9]<br>(-10.0)	44.4<br>[47.6]<br>(47.6)

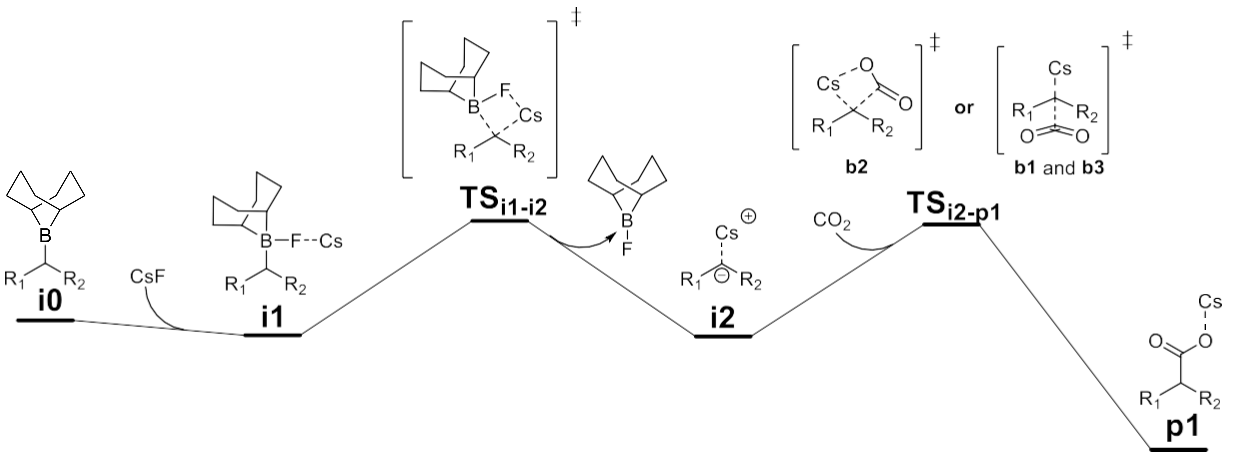

Figure S8. Reaction mechanism C.

**Table S4:** Computed energies (kcal/mol) for mechanism **C** for substrates **b1, b2** and **b3**. Numbers in bold are $\Delta G_{DPLNO\text{-}CCSD(T)//\omega\text{-}B97XD}$ energies (given in the main text), numbers in brackets are $\Delta G_{DPLNO\text{-}CCSD(T)//\omega\text{-}B97XD}$ energies with solvent corrections added ($\Delta E_{sol\_\omega\text{-}B97XD/BS1}$), whereas energies in parentheses are pure DFT $\Delta G_{\omega\text{-}B97XD}$ energies.						
Substrate	i1	TS$_{i1\text{-}i2}$	i2	TS$_{i2\text{-}p1}$	p1	**Overall barrier**
**b1**	-15.5<br>[-10.3]<br>(-12.8)	18.6<br>[24.4]<br>(19.6)	7.4<br>[9.9]<br>(8.6)	14.2<br>[19.2]<br>(16.1)	-17.2<br>[-15.3]<br>(-12.9)	34.0<br>[34.7]<br>(32.5)
**b2**	-14.6<br>[-9.5]<br>(-11.7)	22.1<br>[27.4]<br>(22.4)	12.7<br>[12.3]<br>(15.3)	19.9<br>[21.1]<br>(24.3)	-14.5<br>[-13.6]<br>(-10.7)	36.7<br>[36.9]<br>(34.0)
**b3**	1.0<br>[3.1]<br>(6.2)	44.8<br>[49.5]<br>(49.2)	37.2<br>[36.8]<br>(43.8)	51.1<br>[52.3]<br>(57.1)	-17.2<br>[-16.9]<br>(-10.0)	51.1<br>[52.3]<br>(57.1)

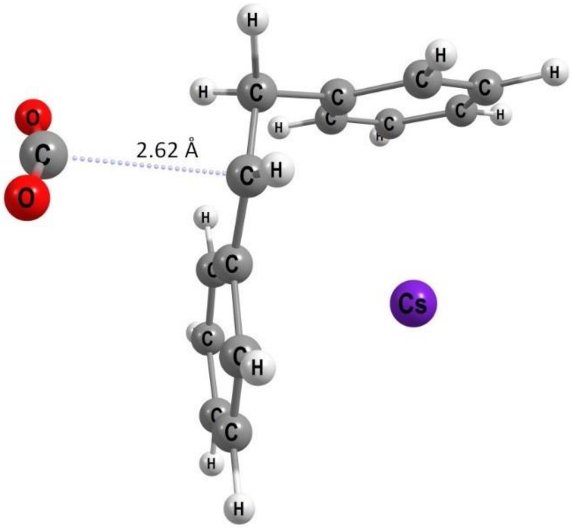

TS$_{i2\text{-}p1}$I (b1): 14.2 kcal/mol

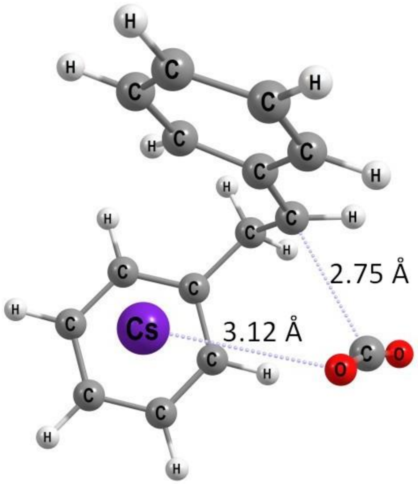

TS$_{i2\text{-}p1}$II (b1):14.9 kcal/mol

Figure S9. Comparison of optimized TS structures for C-CO_2 bond formation with substrate **b1** in Mechanism **C**. The outer/acyclic TS [**TS$_{i2\text{-}p1}$I (b1)**, left] is preferred by 0.7 kcal/mol ($\Delta G_{DPLNO\text{-}CCSD(T)//\omega\text{-}B97XD}$) in comparison to the inner/cyclic TS [**TS$_{i2\text{-}p1}$II (b1)**, right].

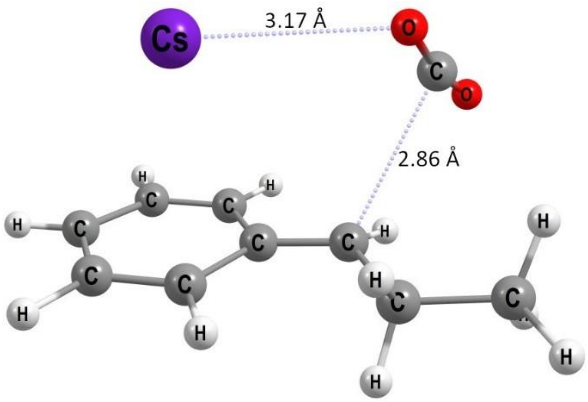

TS$_{i2\text{-}p1}$I (b2): 19.9 kcal/mol

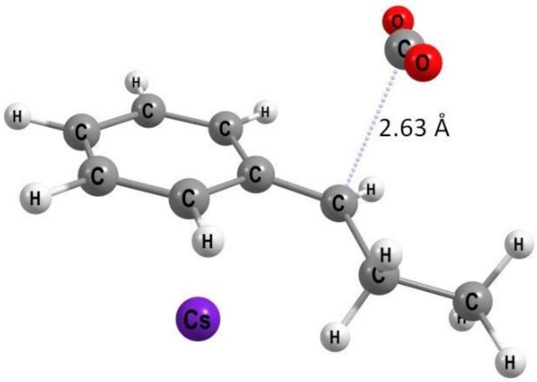

TS$_{i2\text{-}p11}$II (b2): 21.5 kcal/mol

Figure S10. Comparison of optimized TS structures for C-CO_2 bond formation with substrate **b2** in Mechanism **C**. The inner/cyclic TS [**TS$_{i2\text{-}p1}$I (b2)**, left] is preferred by 1.4 kcal/mol ($\Delta G_{DPLNO\text{-}CCSD(T)//\omega\text{-}B97XD}$) in comparison to the outer/acyclic TS [**TS$_{i2\text{-}p1}$II (b2)**, right].
